# Genomic diversity of Campylobacter jejuni and Campylobacter coli isolates recovered from human and poultry in Australia and New Zealand, 2017 to 2019

**DOI:** 10.1099/mgen.0.001319

**Published:** 2024-11-05

**Authors:** Danielle M. Cribb, Patrick J. Biggs, Angus T. McLure, Rhiannon L. Wallace, Nigel P. French, Kathryn Glass, Martyn D. Kirk

**Affiliations:** 1 Australian National University, National Centre for Epidemiology and Population Health, Canberra, Australia; 2 Massey University, Tāwharau Ora|School of Veterinary Science, Palmerston North, New Zealand; 3 Massey University, School of Natural Sciences, Palmerston North, New Zealand; 4 Massey University, New Zealand Food Safety Science and Research Centre, Palmerston North, New Zealand; 5 Agriculture and Agri-Food Canada, Agassiz Research and Development Centre, Agassiz, British Columbia, Canada

**Keywords:** Australia, *Campylobacter*, genomics, New Zealand, poultry, public health

## Abstract

We used genomic and epidemiological data to assess and compare the population structure and origins of *Campylobacter,* a major foodborne pathogen, in two neighbouring countries with strong trade and cultural links, similar poultry production systems and frequent movement of people and food products. The most common sequence types (STs) differed between Australia and New Zealand, with many unique to each country. Over half of all STs were represented by a single isolate. Multidrug-resistant (MDR) genotypes were detected in 0.8% of all samples, with no MDR isolates detected in poultry. Quinolone and tetracycline resistant ST6964 was prevalent in New Zealand (10.6% of *C. jejuni*). Closely related isolates suggested some similar food sources or contacts. We have shown that there is little genetic overlap in human and poultry STs of *Campylobacter* between the countries, which highlights that this common foodborne pathogen has domestic origins in Australia and New Zealand.

## Data Summary

We used previously deposited short-read WGS data for all isolates to the NCBI Sequence Read Archive under the BioProjects PRJNA560409, PRJNA591966, PRJNA592186 and PRJNA675916 (https://www.ncbi.nlm.nih.gov/sra) (Table S1, available in the online Supplementary Material) as the source material for this study. All other supporting data have been provided within the article or through supplementary data files.

**Impact Statement**

This study contributes to the field by examining the genetic relatedness of *Campylobacter* isolates from Australia and New Zealand using large, contemporaneous genomic datasets. It integrates epidemiological and genomic data to identify closely related isolates within poultry systems and between humans and poultry. The research highlights the utility of combining epidemiological data with whole-genome sequencing to detect outbreaks from sporadic cases and retail meat sampling and to link human cases to potential sources. This research is highly relevant for enhancing the understanding of *Campylobacter* diversity and source pathways among practitioners, researchers and policymakers, informing infection reduction strategies. The detection of multidrug resistance in a small percentage of human isolates and the ongoing presence of strains of public health concern in New Zealand emphasise the need for continued surveillance and antimicrobial resistance management.

## Introduction

*Campylobacter* spp. are common bacterial causes of gastroenteritis in humans [1]. There are 45 known species and 13 subspecies of *Campylobacter*, with *C. jejuni* and *C. coli* well-established in poultry and humans [1,2]. Among high-income countries, Australia (160 cases per 100  000 population) and New Zealand (115 cases per 100 000 population) have some of the highest global notification rates for campylobacteriosis circa 2022 [3,4].

*Campylobacter* spp. are normal intestinal flora in wild and domesticated bird species, with broiler flocks showing a high carriage rate of *Campylobacter* [5]. Human *Campylobacter* infections are frequently linked to the consumption of poultry, especially from cross-contamination and handling of undercooked chicken [6,7]. Recent source attribution studies in Australia and New Zealand have confirmed that chicken remains a prominent source of both *C. jejuni* and *C. coli* infections in humans, accounting for ≥80% cases [7,8]. Australia and New Zealand have similar poultry production systems and a large market share from a few large poultry companies, including one common producer [9,12]. The countries have strong cultural, political and economic ties, including the frequent travel of people and inspection-free trade of low risk food through the Trans-Tasman Mutual Recognition Agreement [13]. In general, poultry supply is restricted to each country in vertically integrated, farm-to-fork, domestic production systems [9,12].

Detection of *Campylobacter* spp. includes culture-based and non-culture methods, including polymerase chain reaction and immunological assays [14]. Whole-genome sequencing (WGS) is a highly precise and discriminatory method for identifying and characterizing pathogens from cultured isolates [15]. It allows accurate species and sequence type (ST) identification while providing additional insights into virulence and antimicrobial resistance (AMR) genes and mutations and high-resolution data on the genetic makeup of the organism to the single nucleotide level [15]. WGS data can be used to track the transmission of specific STs and analyse the evolution of *Campylobacter* populations [16]. This detailed genomic information significantly enhances source attribution studies and improves our understanding of *Campylobacter*’s epidemiology and ecology.

Genomic surveillance, reporting and characterisation of isolates can differ across countries, with comparative genomics often focused on isolates from a single country or community. The prevalence of different STs may vary between countries [17], with some STs associated with unique or important characteristics, including high pathogenicity and genetic resistance determinants [18]. In this study, we compared human and poultry *C. jejuni* and *C. coli* isolates collected from Australia and New Zealand between 2018 and 2019 to provide a snapshot of the distribution and relatedness of *Campylobacter* in the region. We aimed to (i) identify predominant STs within Australia and New Zealand, (ii) identify related isolates by core genome single nucleotide polymorphisms (SNP) paired with epidemiological and sampling data and (iii) compare the prevalence of AMR genes and mutations between countries and sources.

## Methods

### Human isolate collection

The present study used human and poultry isolate sequence data and metadata from previous studies, with detailed methods for both studies published elsewhere [6,7, 19, 20]. These studies had consistent methodologies and aimed to identify key sources of *Campylobacter* in Australia and New Zealand. Briefly, *Campylobacter* spp. isolates from Australia were collected from faecal samples of individuals with notified campylobacteriosis from the Australian Capital Territory (ACT), New South Wales (NSW) and Queensland (QLD) between February 2018 and October 2019 [6]. We included additional isolates from human cases across the Northern Territory, South Australia, Tasmania (TAS, Victoria (VIC) and Western Australia during a snapshot study period from October 2018 to February 2019 [19]. *Campylobacter* spp. isolates from New Zealand were collected from faecal samples of individuals with notified campylobacteriosis from Auckland or Manawatū/Whanganui regions between March 2018 and March 2019 [7]. Individuals with suspected campylobacteriosis had stool samples submitted to local pathology laboratories in Australia and regional clinical diagnostic laboratories in New Zealand for pathogen identification. Isolates were sent to reference laboratories where they were prepared for WGS.

### Poultry meat sample collection

Raw chicken meat and offal were collected in Australia from retail stores in VIC, NSW and QLD between March 2017 and March 2019, and from the ACT between May and September 2018 [21]. Offal samples included liver, giblets and heart. Poultry samples were collected in New Zealand directly from rinses of chicken carcasses sampled at processing plants within the same region as notified cases and from laboratories conducting statutory monitoring programmes for the poultry industry between March 2018 and March 2019 [7].

### WGS and genomic analysis

All *Campylobacter* isolates had genomic DNA extracted and WGS performed using an Illumina platform (Illumina, San Diego, California, USA; NextSeq 500 for Australia and HiSeq X Ten for New Zealand) [7,20]. Libraries were prepared using the Nextera XT Library Prep Kit, as detailed in previous studies [7,20]. Paired read sets were analysed using the Nullarbor pipeline (v. 2.0; https://github.com/tseemann/nullarbor). Read output per isolate was evaluated to ensure a minimum of 50 × depth using an average genome size of 1.7 Mb. We used centrifuge (v. 1.0.4) to confirm taxonomic classification and isolate purity (https://github.com/DaehwanKimLab/centrifuge). We performed *de novo* assembly of sequences into contigs with SKESA (v. 2.4.0; https://github.com/ncbi/SKESA). Isolates with >200 contigs were excluded.

### Multi-locus sequence type and *C. coli* clade analysis

The multi-locus sequence type (MLST) was determined for each isolate using mlst software (v. 2.23.0; https://github.com/tseemann/mlst) and the PubMLST *Campylobacter jejuni/coli* database [22]. We used GrapeTree to visualise minimum spanning trees based on the seven gene MLST profiles. As some of the Australian *C. coli* isolates did not belong to clade 1a (ST828 clonal complex; CC), we performed additional bioinformatic analysis (as per methods above) comparing this subset with isolates from a well-cited paper from the United Kingdom (UK) on *C. coli* introgression to define their clade and sub-clade (for example, clade 1b [ST1150 CC], 1c, 2 or 3) [23].

### Core genome sequence comparison

For the core genome comparison, reads were aligned to reference genome strains NZ_CP046317.1 and NZ_CP046318.1 for *C. coli* and NC_003912 for *C. jejuni* using Snippy (v. 4.6). Maximum likelihood phylogenetic trees were inferred from SNPs within the core genome using the general time reversible model with Gamma distance with IQTree (v. 2.2.2.3) [24]. We used Interactive Tree of Life v6 and GrapeTree for visualisation [25,26].

### Genetic determinants of resistance

We screened assembled contigs for known AMR genes using NCBI’s AMRFinderPlus (v. 3.10.1; https://www.ncbi.nlm.nih.gov/pathogens/antimicrobial-resistance/AMRFinder/) and Abricate (v. 1.01.1; https://github.com/tseemann/abricate) with ≥90% gene coverage and >95% sequence identity (Table S2). We investigated two known mutations in housekeeping genes *gyrA* (T→I at amino acid position 86) associated with quinolone resistance and 23S rRNA (nucleotides at positions 2074 and 2075) associated with macrolide resistance using PointFinder [27,29]. We also investigated a mutation in the promotor region of the *bla_OXA-61_* gene (G→T at position 57) that inactivates *bla_OXA-61_* gene expression and results in sensitivity to ampicillin [30]. The present study did not perform phenotypic AMR testing. However, our previous study using CampySource human isolates found moderate to high concordance between phenotypic and genotypic resistance for all antimicrobial classes tested [19]. We classified isolates as resistant if they had one or more of resistance genes *aadE-Cc, aad9, aph(3')-Illa, bla_OXA-184_, bla_OXA-185_, lnu*(C), *tet*(O), *erm*(B) or mutations *bla_OXA-61_* G57T, *gyrA* T86I or 23S rRNA A2074T or A2075G.

### Statistical analysis

We performed data analyses and visualisation using R statistical software (v. 4.3.0) [31] and PAST (v. 4.03) [32]. We calculated rarefaction curves using the vegan package (v. 2.6-4) and Simpson’s and Shannon’s diversity indices and their 95% bootstrap confidence intervals with 1000 iterations using PAST [32,33]. We used ggplot2 (v. 3.4.4) and ComplexUpset (v. 1.3.3) to visualize data [34,35]. We considered *p* values of <0.05 as significant for all statistical tests.

## Results

### Isolate summary

We included 2022 *C*. *jejuni* or *C. coli* isolates from poultry and humans in our analyses (Table 1). There was no significant difference between Australian and New Zealand *C. jejuni* human (47.2% Australia; 556 vs 623) and chicken (50.8% Australia; 191 vs 185) isolates (*P*=0.242). However, there was a significant difference between Australian and New Zealand *C. coli* human (81.6% Australia; 120 vs 27) and chicken (89.4% Australia; 286 vs 34) isolates (*P*=0.031), with fewer total *C. coli* isolates from New Zealand (13.1%; 61/467).

**Table 1.** Number of isolates collected in Australia and New Zealand for *Campylobacter jejuni* and *Campylobacter coli* by source type, 2017 to 2019

	Australia		Subtotal	New Zealand		Subtotal	Total
**Species/source**	Human	Chicken		Human	Chicken		
*C. jejuni*	556	191	747	623	185	808	**1555**
*C. coli*	120	286	406	27	34	61	**467**
Subtotal	676	477		650	219		
**Total**	**1153**			**869**			**2022**

### Species summary and MLSTs

The dominant species was *C. jejuni,* accounting for 76.9%(1555/2022) of isolates and was most frequently isolated in all studied sources. We detected 150 *C*. *jejuni* STs, with 24 (16.0%) detected in both countries. These 24 STs accounted for 58.1% (903/1555) of isolates. We detected 69 singleton STs (46.0%, Table S3). The most common ST detected in Australia was ST50 (15.9% of isolates, 119/747), while the most common in New Zealand was ST45 (17.6% of isolates, 142/808). In Australia, 87.7%(620/707) of isolates were found in STs that were detected in both chicken and human samples, while 83.3%(673/808) of isolates in New Zealand were found in STs from both sources. Two novel STs were detected in New Zealand and five were detected in Australia.

**Fig. 1.**
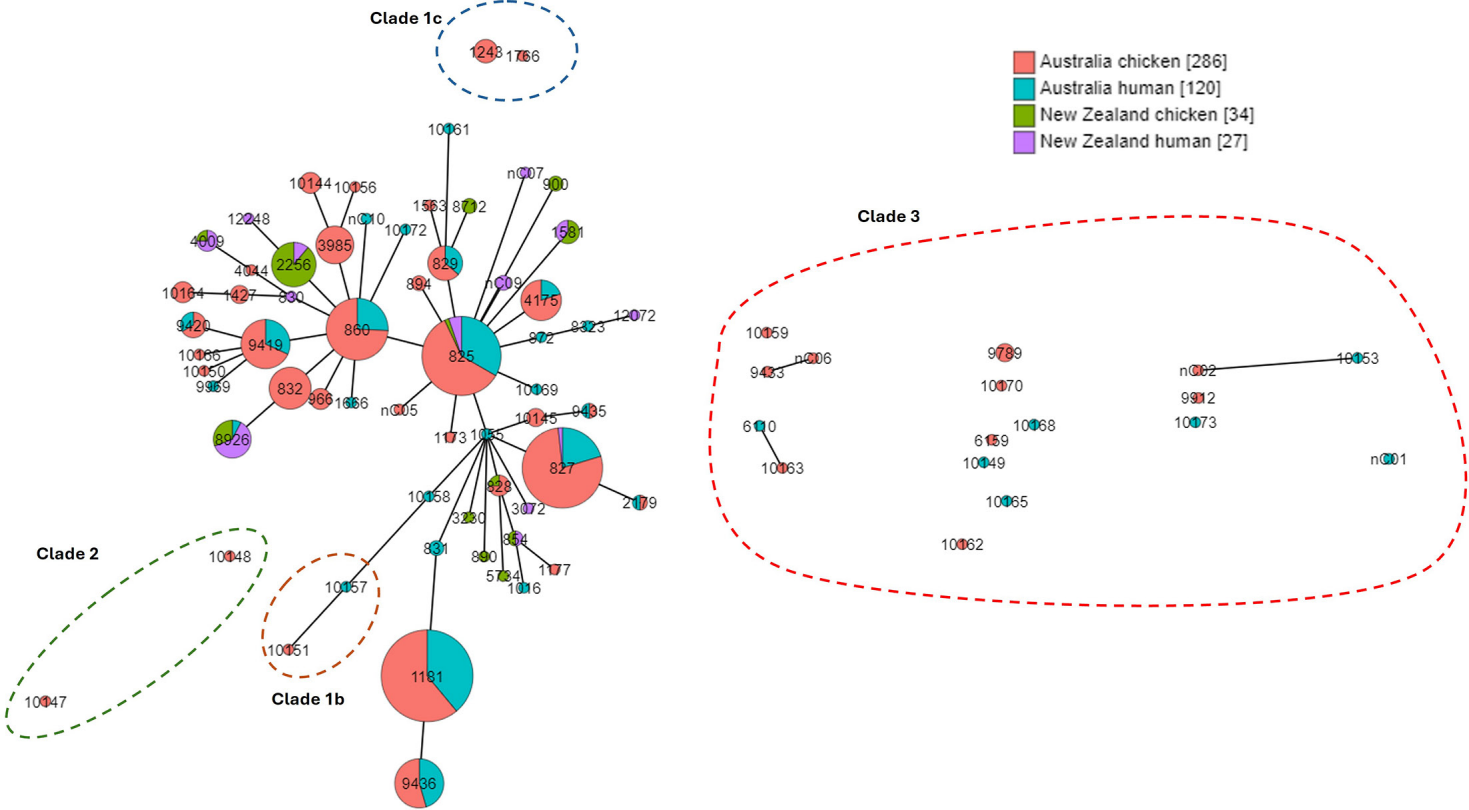
Minimum spanning tree of the distribution of *Campylobacter coli* seven-gene MLSTs in Australian chicken (pink) and human (teal) and New Zealand chicken (green) and human (purple) isolates. ST is indicated by the number on the node. Increasing node size indicates a larger number of isolates in the respective ST. Solid connecting lines infer phylogenetic relatedness and represent STs with four or more loci in common. The length of the line increases as the number of different alleles increases. Isolates without a balloon or brace are clade 1a. Square brackets in the legend indicate the number of isolates per source/country grouping.

We detected 79 *C*. *coli* STs, with only four (5.1%) detected in both Australia and New Zealand. However, these accounted for 28.5% of isolates (133/467). We detected 48 STs (60.8%) represented by a single isolate (Table S3). Fifty-six *C. coli* STs were either classified as ST828 CC (clade 1a) on PubMLST or clustered with isolates from this CC, capturing all New Zealand *C. coli* isolates. ST10151 and ST10157 isolates clustered with clade 1b isolates from the UK, and ST1243 and ST1766 clustered with clade 1c UK isolates (Fig. S1 and Table S4). ST10147 and ST10148 clustered with clade 2 UK isolates and the remaining 17 STs clustered with clade 3 UK isolates. The most common *C. coli* ST detected in Australia was ST1181 (19.0%, 77/406) while the most common in New Zealand was ST2256 (29.5%, 18/61). In Australia, 74.6% of *C. coli* isolates were from STs that were identified in both human and chicken sources (303/406), while 73.8% of isolates in New Zealand were in STs found in both sources (45/61). Nine novel STs were detected in New Zealand, while none were detected in Australia. The population structures of STs for both *C. jejuni* and *C. coli* are presented in minimum spanning trees in Figs1  2 and in maximum likelihood trees in Figs S2 and S3.

**Fig. 2.**
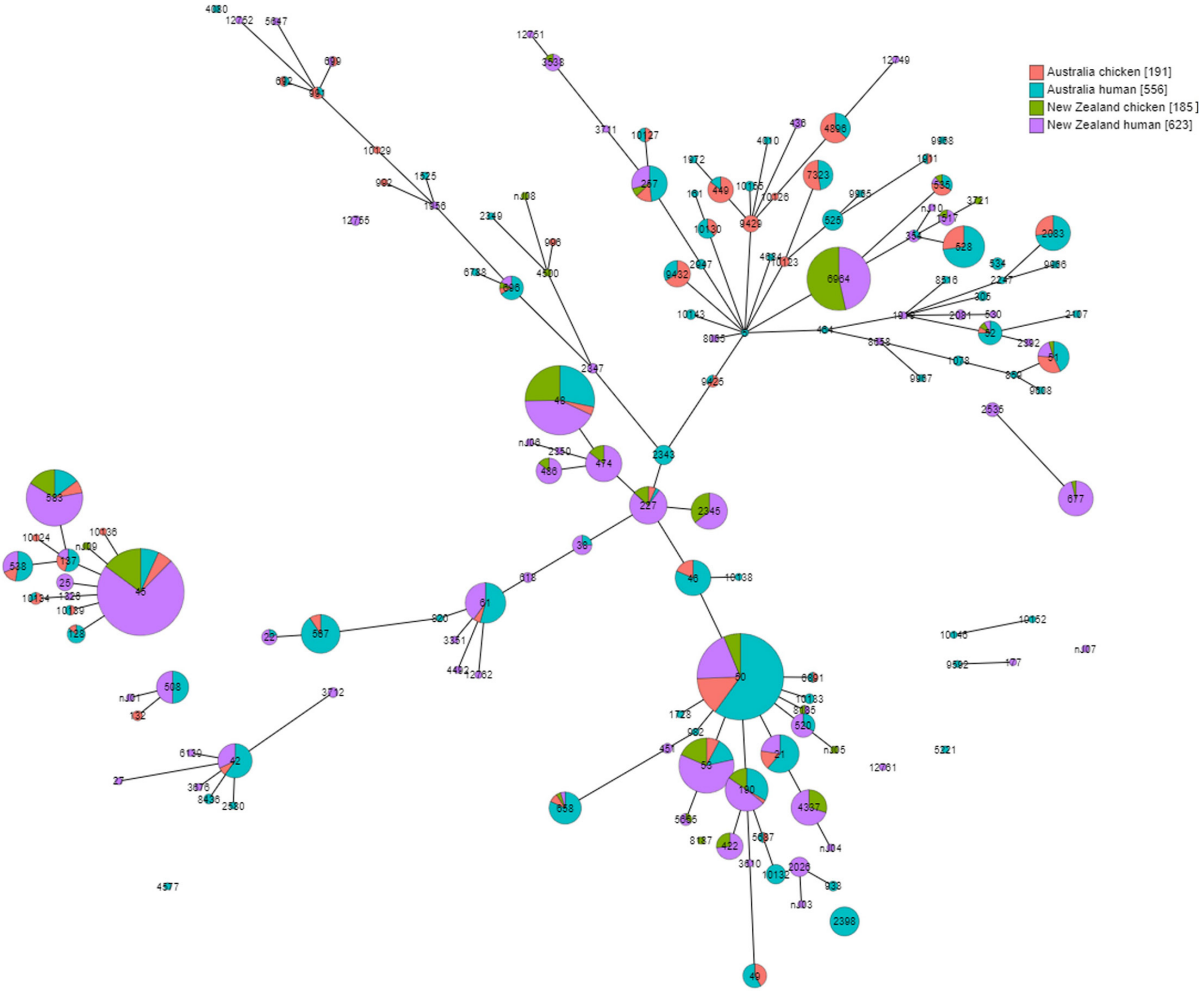
Minimum spanning tree of the distribution of *Campylobacter jejuni* seven-gene MLSTs in Australian chicken (pink) and human (teal) and New Zealand chicken (green) and human (purple) isolates. Increasing circle size indicates a larger number of isolates of the respective ST. Solid connecting lines infer phylogenetic relatedness and represent STs with four or more loci in common. The length of the line increases as the number of different alleles increases. Square brackets in the legend indicate the number of isolates per source/country grouping.

### MLST diversity

Simpson’s and Shannon’s indices of diversity and rarefaction curves for *C. jejuni* and *C. coli* are presented in Figs S4 and S5. Diversity was generally higher in Australian *C. jejuni* isolates compared to New Zealand, with New Zealand chicken isolates showing the lowest diversity. *C. coli* chicken isolates from Australia were more diverse than those from New Zealand, whereas *C. coli* isolates from humans showed similar diversity between the two countries. We note there is a substantial difference in * C. coli* sample numbers between Australia and New Zealand and between *C. jejuni* chicken and human samples. Diversity indices and figures were largely in agreement.

### SNP density

**Fig. 3.**
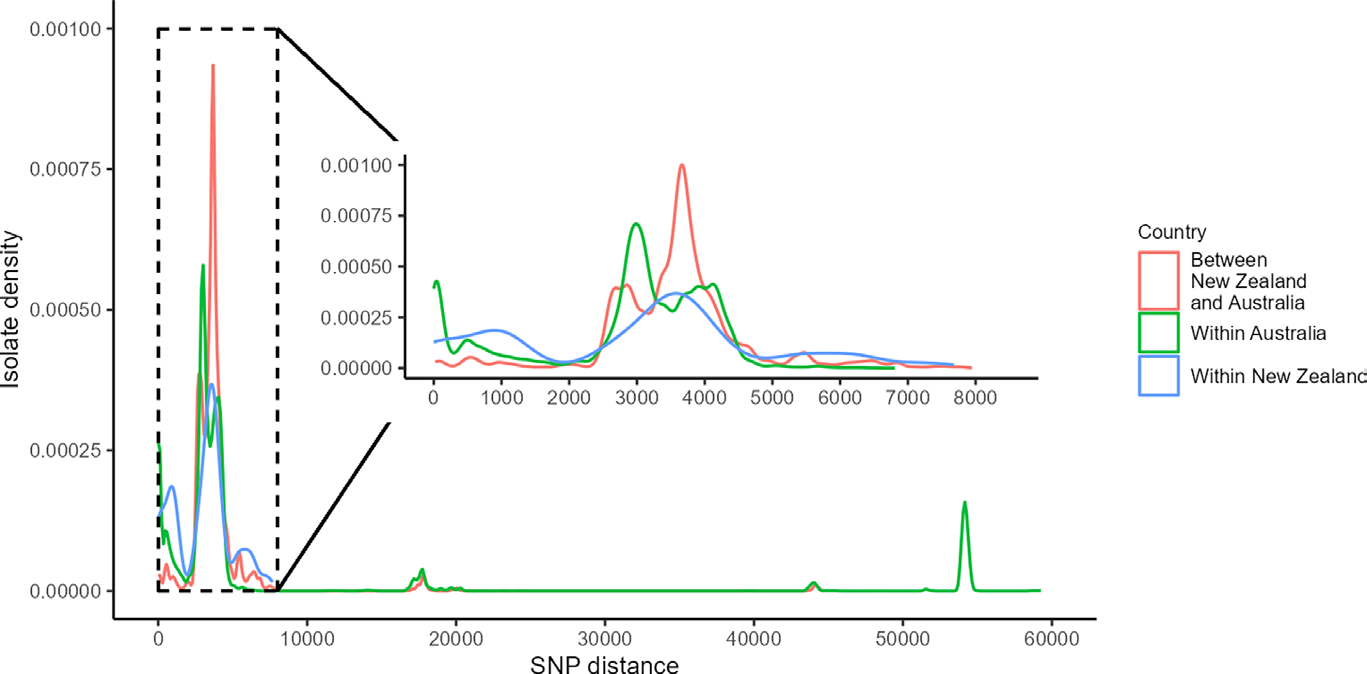
*Campylobacter coli* core genome SNP distance kernel-smoothed density graph for isolates within Australia (*n* = 406), New Zealand (*n* = 61) and comparison between the two countries (*n* = 467). Graph represents 82  621 pairwise SNPs for Australian isolates, 1891 pairwise SNPs for New Zealand isolates and 23  546 pairwise SNPs between countries. Inset graph highlights clade 1a isolates within Australia (*n* = 377), New Zealand (*n* = 61) and comparison between countries (*n* = 438). Inset graph represents 71  253 pairwise SNPs for Australian isolates, 1891 pairwise SNPs for New Zealand isolates and 22  997 pairwise SNPs between countries.

We compared core genome SNP density within and between countries for both *Campylobacter* species. For *C. coli*, the SNP distance in Australian isolates was driven by the outlier isolates from clades 1b, 1c, 2 and 3 (Fig. 3), ranging up to 59 262 SNPs. When restricted to clade 1a isolates, SNP distances ranged from 0 to 6806 SNPs for Australian isolates and 0 to 7678 SNPs for New Zealand isolates, with 23 to 7945 SNPs between the two countries (Fig. 3 inset). Australian pairwise distances peaked at ~3000 SNPs at a density of 7.09e−04. New Zealand pairwise distances peaked at ~3600 SNPs at a density of 3.68e−04.

**Fig. 4.**
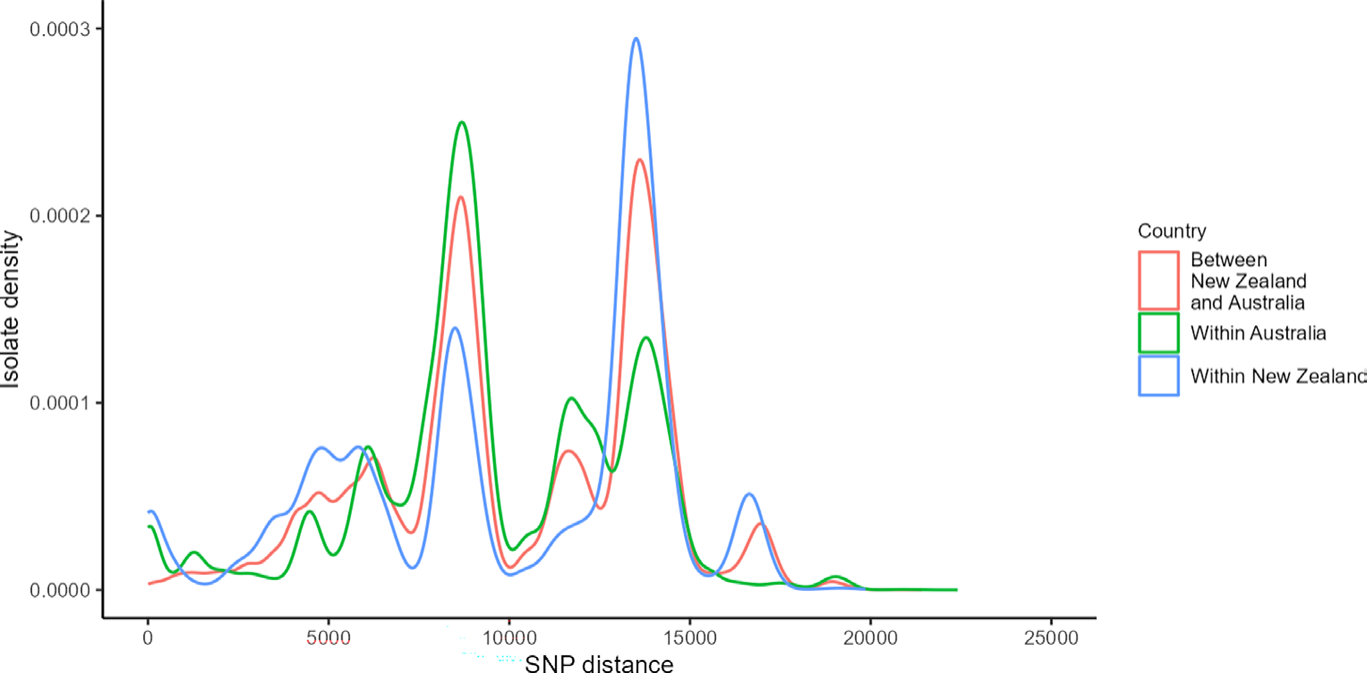
*Campylobacter jejuni* core genome SNP distance density graph for isolates within Australia (*n* = 747), New Zealand (*n* = 808) and comparison between the two countries (*n* = 1555). Graph represents 279  378 pairwise SNPs for Australia, 326  836 pairwise SNPs for New Zealand and 603  576 pairwise SNPs between countries.

For *C. jejuni,* Australian isolates ranged from 0 to 22404 SNPs, New Zealand isolates from 0 to 19 856 SNPs, with 7 to 21  424 SNPs between both countries (Fig. 4). Australian pairwise distances peaked at ~8700 SNPs at a density of 2.5e−04, driven by the high density of SNPs for ST658 CC, ST581 CC and ST49 CC isolates. A secondary peak at ~13 800 SNPs was driven by SNPs in isolates from multiple CCs, including ST1332 CC, ST45 CC, ST403 CC and ST508 CC. New Zealand pairwise distances peaked at ~13  500 SNPs at a density of 2.94e−04, driven by SNPs for isolates assigned to ST403 CC, ST1332 CC, ST508 CC, ST362 CC, ST22 CC and ST42 CC. A second peak at ~8500 SNPs was driven by SNPs for ST658 CC, ST354 CC and ST257 CC isolates.

### SNP clustering

We explored STs that included Australian and New Zealand isolates within 100 core genome SNPs to understand possible common sources for isolates within and between countries. Within *C. coli* STs that included isolates from both countries, both ST825 and ST827 contained isolates from Australia and New Zealand between 20 and 50 SNPs and included isolates from humans and chickens (Table S5). *C. jejuni* also contained isolates between 20 and 50 SNPs from Australian and New Zealand humans and chickens for ST45, ST50, ST538, ST696 and ST38 (human only), with a further two clusters between 5 and 20 SNPs for ST508 (human only) and ST137 (Table S5).

**Fig. 5.**
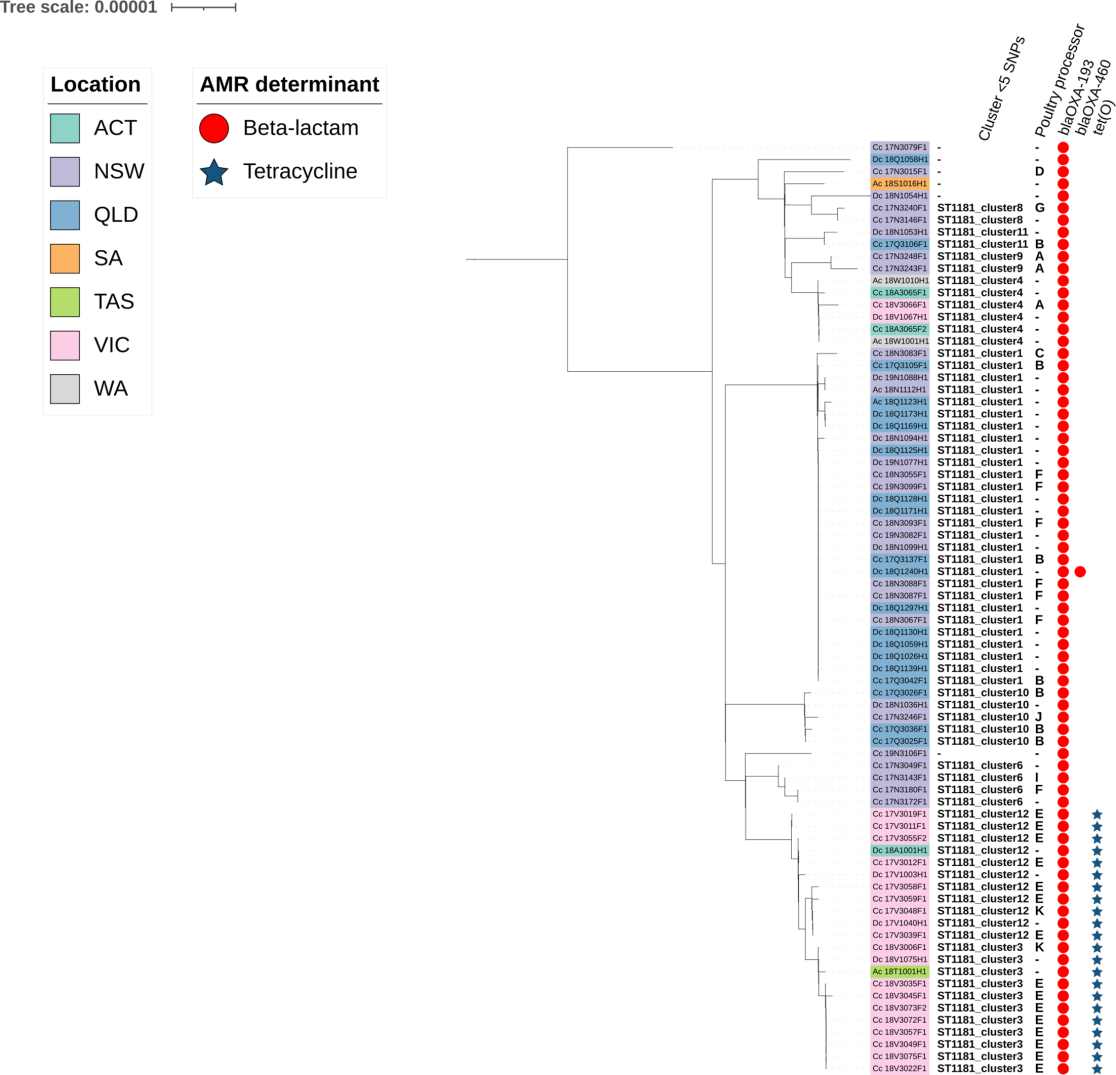
Maximum likelihood phylogenetic tree from core genome pairwise SNP for *Campylobacter coli* ST1181 from human and poultry sources in Australia. Metadata indicate the cluster ID at the <5 SNPs threshold, the poultry processor, where information was available, and the presence or absence of detected AMR genes *bla_OXA-193_*, *bla_OXA-460_* and *tet(O*).

We paired metadata from food sampling with isolate genomic data for closely related chicken isolates in Australia (<5 SNPs). We detected two ST1181 clusters from 2017 (cluster 12) and 2018 (cluster 3) samples that could both be traced back to the same poultry processor (Fig. 5). Additionally, isolates in both clusters possessed the *bla_OXA-193_* and *tet*(O) AMR genes. This could indicate one prolonged cluster from this processor. Three human isolates are included in these clusters (one from the same state, VIC, and two from the ACT and TAS) and may be linked to consuming chicken from this processor.

### AMR genotypes

We detected a high prevalence of genes associated with β-lactam resistance, with *bla_OXA-193_* and *bla_oxa-61_* detected in 59.0%(917/1555) and 65.7%(1022/1555) of *C. jejuni* and 60.4%(282/467) and 59.7%(279/467) of *C. coli* isolates (Table S6). We examined the G → T mutation at position 57 in the *bla_OXA-61_* promoter region and discovered that only 7.1%(92/1301) of these isolates had an active promoter. The next most detected resistance determinants were tetracycline gene *tet*(O) (12.8% *C*. *jejuni* and 10.7% *C*. *coli*) and fluoroquinolone gene mutation *gyrA* T86I (12.8% *C*. *jejuni* and 0.2% *C*. *coli*). Many resistance genes and mutations were present in both chicken and human isolates from Australia and New Zealand, with some determinants detected in a single *C. jejuni* or *C. coli* isolate, for example, *lnu*(C) and *erm*(B). Resistance determinant profiles were largely conserved within STs for *C. coli* and *C. jejuni*; however, some novel resistance genes or mutations were detected in single isolates (for example, in *C. coli* ST832 and ST4175 and *C. jejuni* ST61) and greater variation in predominant STs like *C. jejuni* ST45 (Figs S2 and S3).

**Fig. 6.**
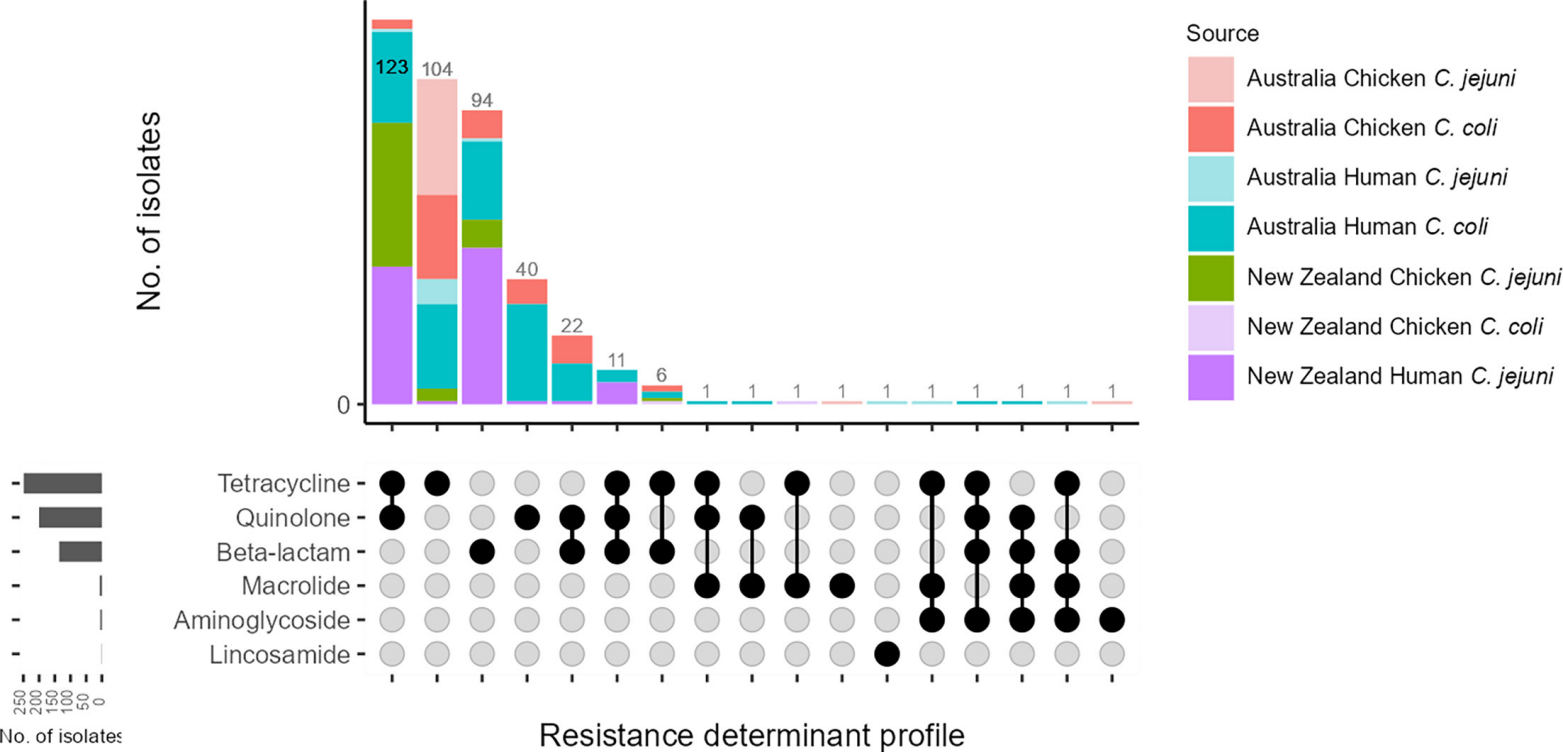
AMR determinants (side) and profiles (top) for Australia and New Zealand chicken and human *Campylobacter* spp. isolates.

We classified isolates as multidrug resistant (MDR) if they possessed genes and/or mutations to three or more drug classes. We only had evidence of phenotypic resistance to ampicillin (β-lactam) from previous work for *bla_OXA-61_* G57T*, bla_OXA-184_* and * bla_OXA-185_*, and as such, we only considered these determinants when categorising isolates as MDR. MDR genotypes were detected in 9 Australian (7 *C*. *jejuni* and 2 *C*. *coli*) and 7 New Zealand (7 *C*. *jejuni* and no *C. coli*) isolates (0.8% overall), with the most common profile of *bla_OXA-61_* G57T+*gyrA* T86I+*tet*(O) in 11 isolates (4 Australia, 7 New Zealand) (Fig. 6 and Table S7). The dual quinolone and tetracycline resistant genotype was the most detected profile in New Zealand isolates, found in all 86 *C*. *jejuni* ST6964 with seemingly sporadic resistance in other STs (Figs 6 and S2, Table S7).

## Discussion

Our study highlights the genetic diversity of *C. jejuni* and *C. coli* STs infecting humans and chickens in Australia and New Zealand. We analysed a large dataset of isolates that were mostly assigned to dominant STs (particularly for *C. jejuni*), with several unique STs found only in either Australia or New Zealand. Importantly, we found low prevalence of MDR determinants compared to human *C. jejuni* and *C. coli* isolates from other countries, including Italy (4.0%, 26/645) and China (22.3%, 99/443) [36,37]. However, the high prevalence of β-lactam resistance genes alongside determinants for quinolone and macrolide resistance – recommended for severe campylobacteriosis treatment – emphasises the need for ongoing AMR surveillance in poultry and humans to detect and monitor emerging resistance [38]. We show that for both geographically close countries, campylobacteriosis from poultry remains an important public health issue.

The genetic heterogeneity observed within and between countries highlights the need for ongoing large-scale surveillance to track the emergence of novel STs and spread of the pathogen. For both *C. jejuni* and *C. coli,* a small number of STs captured a large proportion of human and chicken isolates. This apparent dominance of a few highly populated STs has previously been reported in chicken flocks [39]. The high proportion of STs common to both humans and chickens reflects the ability of these STs to colonise animal reservoirs and subsequently infect humans through food consumption [40]. Conversely, a large proportion of isolates were assigned to singleton and newly defined STs, particularly in *C. coli*, showing that while human and poultry isolates appear in large numbers in dominant STs, emerging STs are present in the population. The large crossover between chicken and human isolates necessitates targeted interventions in poultry production and processing to reduce human campylobacteriosis.

Rarefaction curves and diversity indices were largely in agreement and indicated that *C. jejuni* isolates from humans were more diverse than chicken; Australian *C. coli* chicken samples showed greater diversity than New Zealand, while *C. coli* human isolates had similar diversity. This may reflect differences in geographical sampling methods (i.e. from small regions in New Zealand vs whole-of-country sampling in Australia) or the smaller vertically integrated poultry industry in New Zealand. This difference in species richness has previously been noted in a comparative study of New Zealand and UK isolates [41]. Within New Zealand isolates, human *C. coli* was marginally more diverse than chicken. A previous study in New Zealand found that human *C. coli* was more diverse than other sources individually, but diversity did not differ when all other sources were combined [42]. This is attributed to humans being infected with *Campylobacter* spp. from other sources in which other STs are present or more common, including pigs, cattle, sheep and other unsampled sources [8]. Due to the small number of *C. coli* isolates collected in New Zealand, samples collected from elsewhere within New Zealand may be required to reflect the prevalence and diversity of *C. coli* more accurately.

Approximately 40% of *C. jejuni* isolates and 70% of *C. coli* isolates belonged to STs that were unique to each country. Cases who had travelled internationally were excluded from the study and domestic poultry production chains within each country are vertically integrated with no import or export of raw chicken meat. Poultry production in Australia is controlled by two main companies, while production in New Zealand is controlled by four that each supply approximately 70% of their country’s chicken meat [9,10]. These suppliers own and control most of the production from farms through processing and distribution. This suggests that despite the frequent movement of people and food items between Australia and New Zealand, *Campylobacter* are evolving independently with limited import from the neighbouring countries.

In this study, we were able to detect possible prolonged clusters of *C. coli* ST1181 in Australia linked to a single processor. This finding, alongside previous studies in New Zealand (63% of human cases attributed to a single processor) and Denmark (single year-long cluster representing 12% of cases), demonstrate the value of including WGS in surveillance programmes to identify and track *Campylobacter* clusters over time [43,44]. Due to current technology, cost and resource limitations and the substantially higher case numbers in Australia circa 2022 (*n*=41999) compared to Denmark (*n*=5143) and New Zealand (*n*=5878), countries implementing these analyses into more routine surveillance practices, it is not feasible for all Australian isolates to be sequenced as part of routine surveillance [3,4, 45]. Nevertheless, using WGS routinely for characterisation of strains from notified cases and periodic sampling in farms, processors and retail sites, coupled with collaboration among industry stakeholders, regulators and public health officers, would facilitate the identification of sources of otherwise sporadic illnesses. This approach would enable targeted interventions within facilities along the entire farm-to-fork production system.

We detected low prevalence of MDR genotypes across Australia and New Zealand. We have previously reported low MDR among *Campylobacter* in Australia, which is consistent with a previous study in NSW [19,46]. The most recent Australian chicken meat survey found no phenotypic MDR in *C. jejuni* nor *C. coli* isolates, noting a reduction from previous surveys [47]. Within New Zealand, dual resistance to fluoroquinolone (ciprofloxacin) and tetracycline in *C. jejuni* ST6964 was prevalent in human and chicken isolates. This ST emerged circa 2014 and has remained a major cause of human campylobacteriosis in New Zealand [48]. While this ST does not appear within Australia, ST10130, ST2398 and ST1078 isolates in this study possess this resistance genotype. These STs do not appear to be related to each other nor ST6964. These STs were identified in the chicken meat survey among others known within Australia with this resistance profile [47]. Both Australia and New Zealand do not approve the use of fluoroquinolones in livestock, with resistance levels to this antimicrobial in Australia similar to those of other countries that do not approve fluoroquinolone use in chicken [49,50]. It is hypothesised that these isolates were introduced through human–chicken transmission, but this requires additional longitudinal and genomic studies for validation.

A strength of our study is the comparative analysis of *Campylobacter* from both human and food sources from two countries using studies with similar methods and timeframe. Other multi-country comparative studies of *Campylobacter* exist, but they are limited. These include a study of *C. lari* between Australia and Europe and an ST50 poultry isolate comparison among Australia, Europe and North America [17,51]. These studies help to define population structures and understand regional differences in *Campylobacter*. Another strength is the large sample size of *Campylobacter* isolates from human and poultry sources in both Australia and New Zealand. However, a limitation is the small sample size of *C. coli* isolates from New Zealand. A study of *C. coli* in New Zealand previously estimated that approximately 3% of campylobacteriosis cases could be attributed to *C. coli* infection. They noted that further studies may need to assess the generalisability of regional sampling to represent all of New Zealand and that sampling outside of the study area and from a wider variety of sources may increase representation of clade 2 and clade 3 isolates [42]. Furthermore, we were unable to include the same level of metadata for New Zealand isolates as Australia due to data access limitations. Finally, our study chose to only include chicken and human isolates from Australia and New Zealand. Our rationale was that poultry remains the predominant source of human campylobacteriosis in Australia and New Zealand, with local source attribution studies linking over 80% or more of cases to chickens [7,8]. Both poultry consumption and notified campylobacteriosis cases have also increased in recent years in Australia and New Zealand [3,4, 52]. Isolates from additional sources, including ruminants, pigs and environmental sources, would provide a more comprehensive picture of *Campylobacter* spp. diversity.

*C. jejuni* and *C. coli* from chicken and human samples are genetically diverse within and between Australia and New Zealand. We identified dominant STs that largely differed between countries, along with a high proportion of singleton STs. We were able to incorporate epidemiological and sampling data with core genome SNP data to identify possible prolonged clusters from a single processor in Australian *C. coli* isolates. Furthermore, we confirmed the low presence of MDR isolates in Australian and New Zealand *Campylobacter* spp. but highlighted the need to continue to monitor dual ciprofloxacin and tetracycline resistant STs including those that are emerging. Our study highlighted how integrating WGS into collaborative surveillance programmes among industry, government regulators and public health officials could provide critical insights into *Campylobacter* diversity and the ability to identify possible sources of otherwise sporadic infections.

## supplementary material

10.1099/mgen.0.001319 Uncited Supplementary Material 1.

## Abbreviations

ACT: Australian Capital Territory
AMR: antimicrobial resistance
CC: clonal complex
MDR: multidrug resistant
MLST: multi-locus sequence type
NSW: New South Wales
PCR: polymerase chain reaction
Qld: Queensland
SNP: single nucleotide polymorphism
ST: sequence type
Tas: Tasmania
UK: United Kingdom
Vic: Victoria
WGS: whole genome sequencing
