# Supplementary material for: Genomic diversity of Campylobacter jejuni and Campylobacter coli isolates recovered from human and poultry in Australia and New Zealand, 2017 to 2019
Source: Microb Genom. 2024 Nov 5;10(11):001319. doi: 10.1099/mgen.0.001319 (PMC11893275; doi:10.1099/mgen.0.001319)
Supplement: Uncited Supplementary Material 1. [file mgen-10-01319-s001.pdf]

S1 Fig**.** Maximum likelihood tree of *Campylobacter coli* isolates from Australia and the United Kingdom. Isolates are coloured according to clade or sub-clade. Square brackets indicate the number of isolates included within each sub-clade. The tree is drawn to scale, with branch length measured in the number of substitutions per site. The scale bar represents a genetic distance of 0.05 (i.e., 5.0% of the nucleotides differ). A key is provided for isolate identification in Table S4.

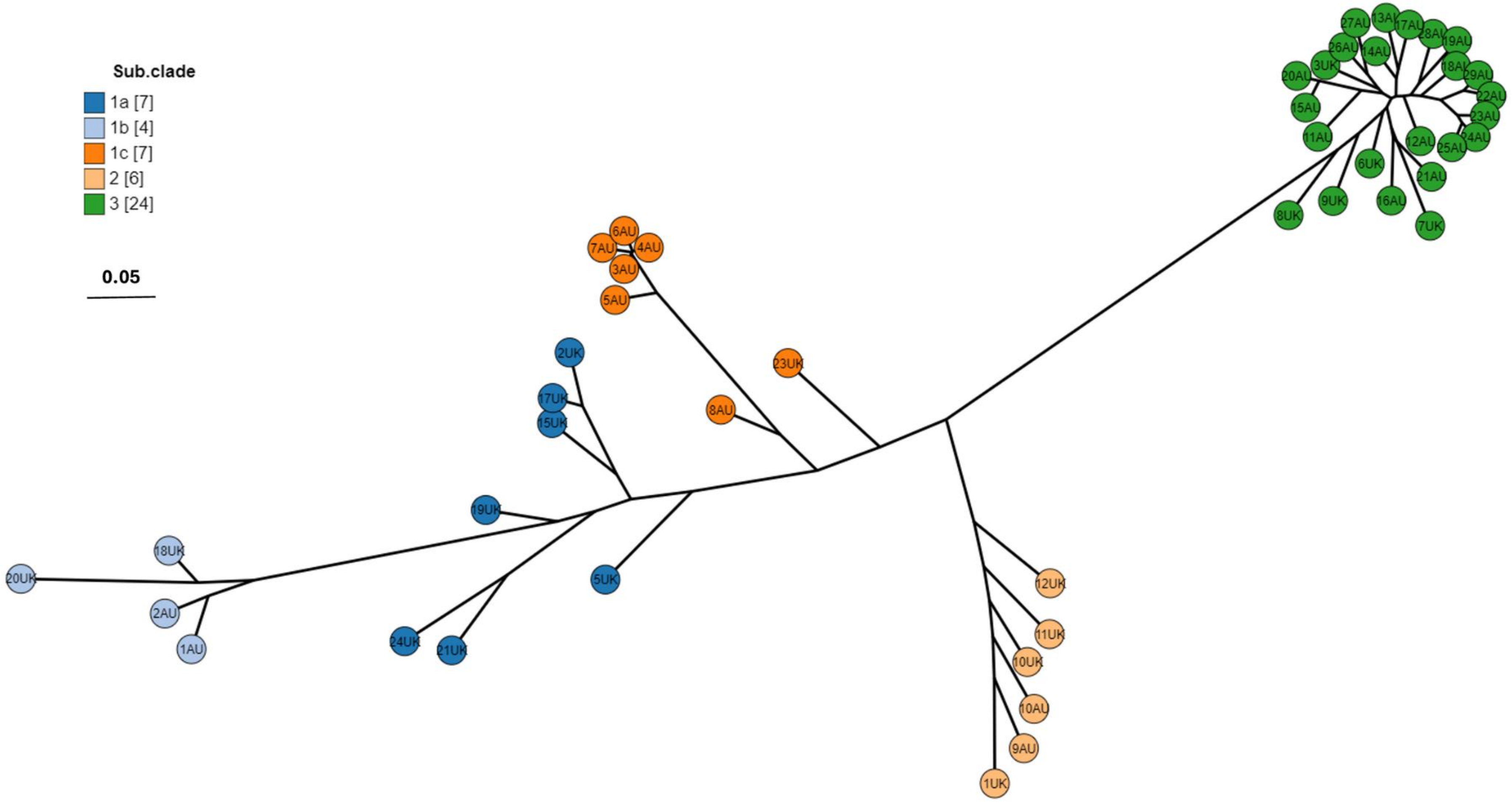

S2 Fig. Maximum likelihood phylogenetic tree showing the core genome relationship between human and chicken Campylobacter jejuni isolates (n=1,555) from Australia and New Zealand, 2017–2019. The circle lanes from inner to outer indicate the state, territory or country, source, multilocus sequence type, and the antimicrobial resistance gene or mutation detected.

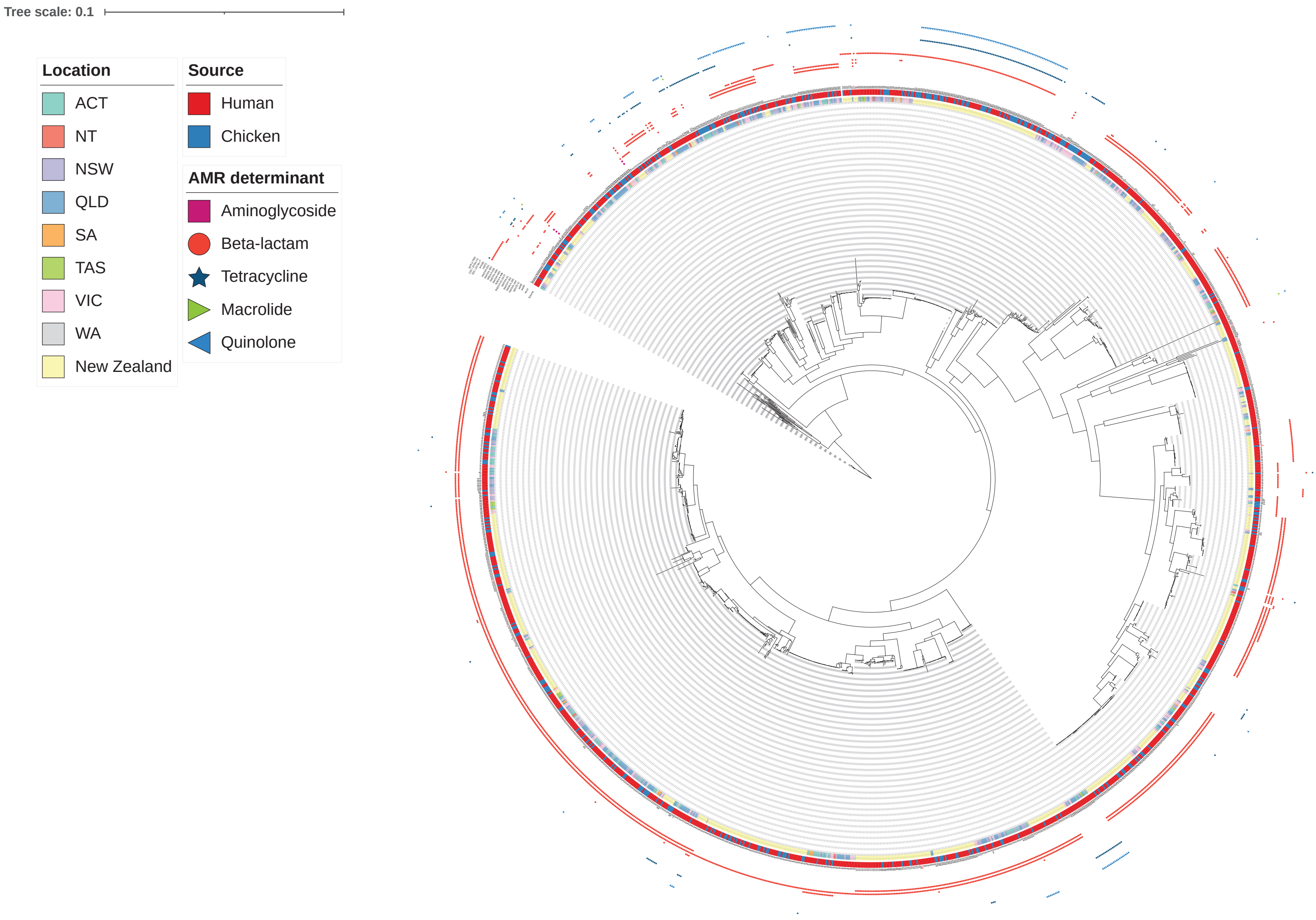

S3 Fig. (A) Maximum likelihood phylogenetic tree showing the core genome relationship between human and chicken Campylobacter coli isolates (n=467) from Australia and New Zealand, 2017–2019. (B) Maximum likelihood phylogenetic tree showing the core genome relationship between human and chicken Campylobacter coli clade 1a isolates (n=438) from Australia and New Zealand, 2017–2019. The circle lanes from inner to outer indicate the state, territory or country, clade, source, multilocus sequence type, and the antimicrobial resistance gene or mutation detected.

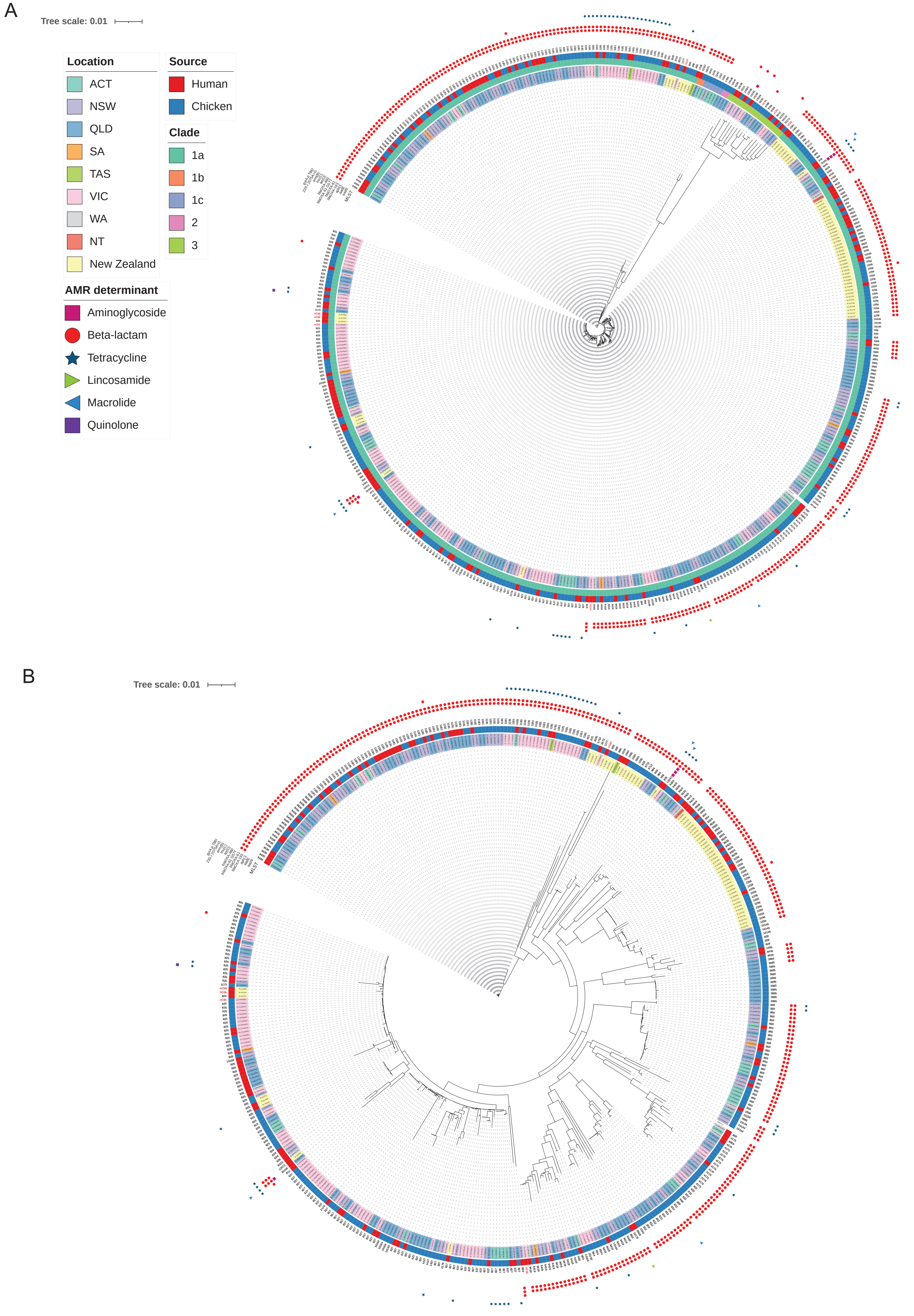

S4 Fig. Rarefaction curves and diversity indices of human and chicken *Campylobacter coli* sequence types from Australia and New Zealand, 2017–2019.

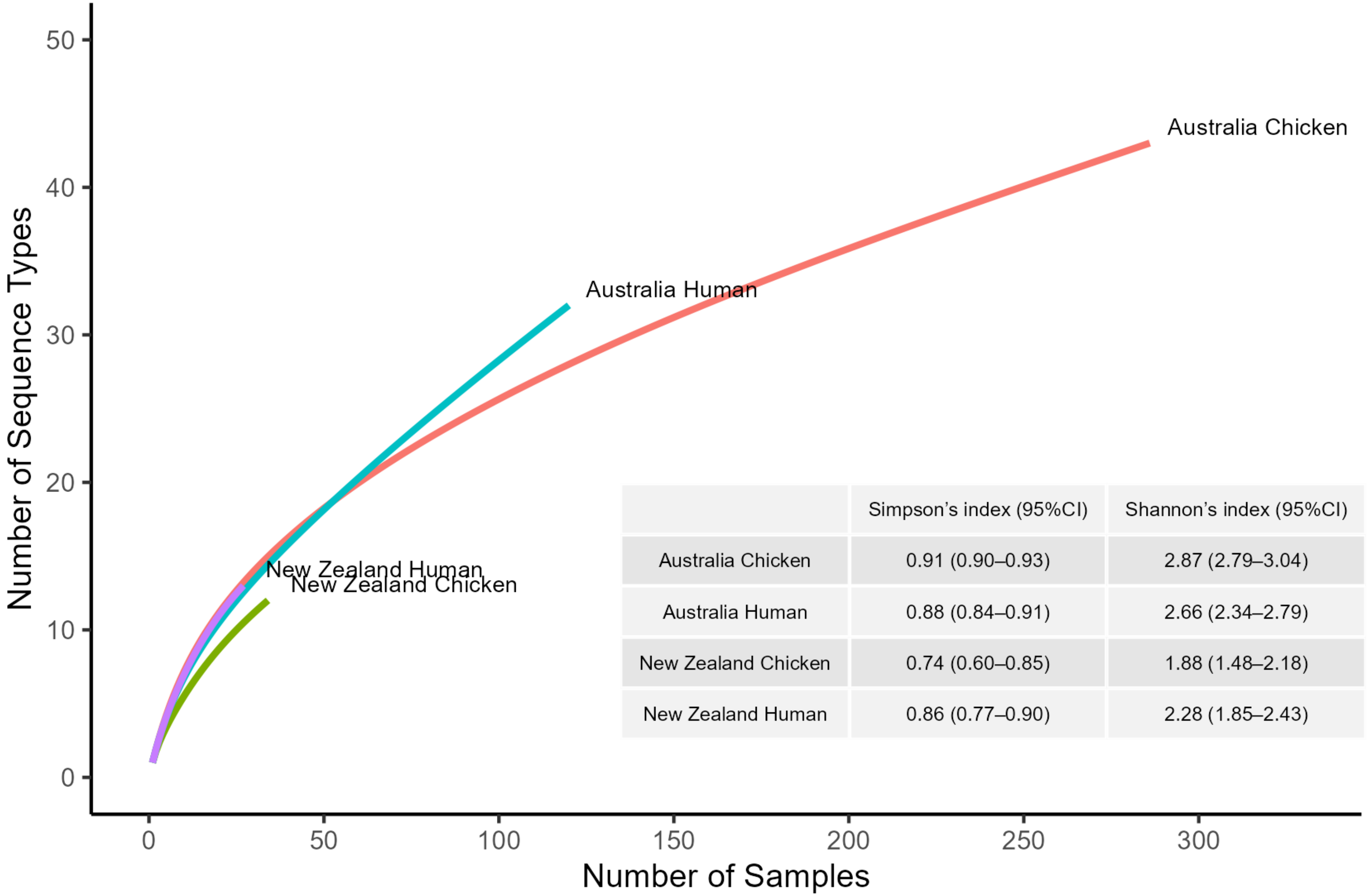

	Simpson's index (95%CI)	Shannon's index (95%CI)
Australia Chicken	0.91 (0.90–0.93)	2.87 (2.79–3.04)
Australia Human	0.88 (0.84–0.91)	2.66 (2.34–2.79)
New Zealand Chicken	0.74 (0.60–0.85)	1.88 (1.48–2.18)
New Zealand Human	0.86 (0.77–0.90)	2.28 (1.85–2.43)

S5 Fig. Rarefaction curves and diversity indices of human and chicken *Campylobacter jejuni* sequence types from Australia and New Zealand, 2017–2019.

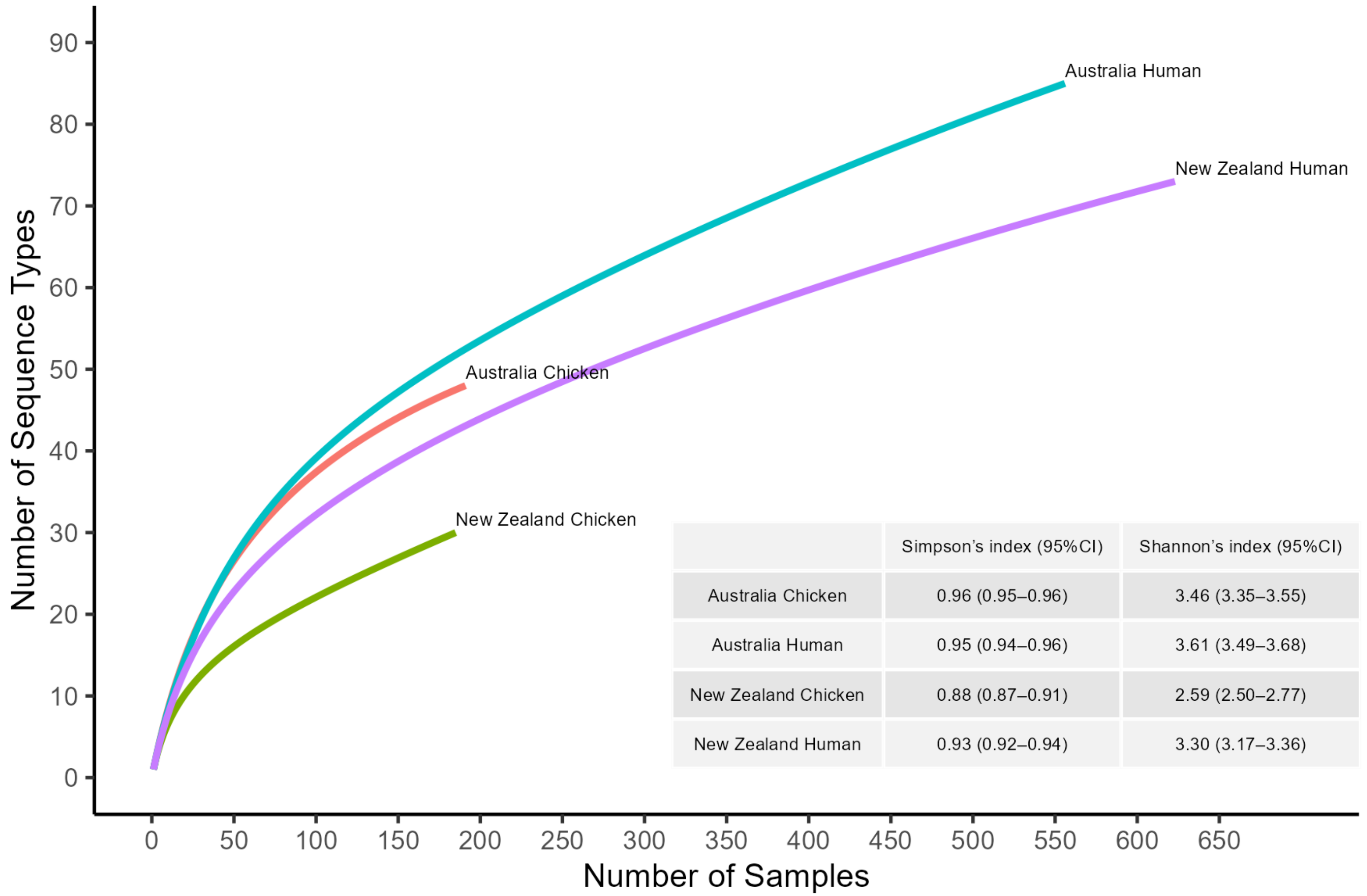

	Simpson's index (95%CI)	Shannon's index (95%CI)
Australia Chicken	0.96 (0.95–0.96)	3.46 (3.35–3.55)
Australia Human	0.95 (0.94–0.96)	3.61 (3.49–3.68)
New Zealand Chicken	0.88 (0.87–0.91)	2.59 (2.50–2.77)
New Zealand Human	0.93 (0.92–0.94)	3.30 (3.17–3.36)

Table S**1.** Summary of *Campylobacter* isolates used in this study.

**Isolate no.**	**Year**	**Accession no.**	**BioProject no.**	**Source**	**Country**	**Species**	**Sequence type**	**Clonal complex**	**SRA hyperlink**
Ac_18A1046H1	2018	SRR9974817	PRJNA560409	Human	Australia	Campylobacter coli	9436		https://www.ncbi.nlm.nih.gov/sra/SRR9974817
Ac_18A1048H1	2018	SRR9974816	PRJNA560409	Human	Australia	Campylobacter coli	4175	ST-828 complex	https://www.ncbi.nlm.nih.gov/sra/SRR9974816
Ac_18D1001H1	2018	SRR9974746	PRJNA560409	Human	Australia	Campylobacter coli	9419		https://www.ncbi.nlm.nih.gov/sra/SRR9974746
Ac_18N1112H1	2018	SRR9974740	PRJNA560409	Human	Australia	Campylobacter coli	1181	ST-828 complex	https://www.ncbi.nlm.nih.gov/sra/SRR9974740
Ac_18N1122H1	2018	SRR9974716	PRJNA560409	Human	Australia	Campylobacter coli	8926	ST-828 complex	https://www.ncbi.nlm.nih.gov/sra/SRR9974716
Ac_18N1130H1	2019	SRR9974710	PRJNA560409	Human	Australia	Campylobacter coli	829	ST-828 complex	https://www.ncbi.nlm.nih.gov/sra/SRR9974710
Ac_18Q1102H1	2018	SRR9974773	PRJNA560409	Human	Australia	Campylobacter coli	825	ST-828 complex	https://www.ncbi.nlm.nih.gov/sra/SRR9974773
Ac_18Q1111H1	2018	SRR9974829	PRJNA560409	Human	Australia	Campylobacter coli	9436		https://www.ncbi.nlm.nih.gov/sra/SRR9974829
Ac_18Q1123H1	2018	SRR9974826	PRJNA560409	Human	Australia	Campylobacter coli	1181	ST-828 complex	https://www.ncbi.nlm.nih.gov/sra/SRR9974826
Ac_18Q1131H1	2018	SRR9974822	PRJNA560409	Human	Australia	Campylobacter coli	860	ST-828 complex	https://www.ncbi.nlm.nih.gov/sra/SRR9974822
Ac_18S1005H1	2018	SRR9974730	PRJNA560409	Human	Australia	Campylobacter coli	860	ST-828 complex	https://www.ncbi.nlm.nih.gov/sra/SRR9974730
Ac_18S1011H1	2018	SRR9974764	PRJNA560409	Human	Australia	Campylobacter coli	9419		https://www.ncbi.nlm.nih.gov/sra/SRR9974764
Ac_18S1015H1	2018	SRR9974768	PRJNA560409	Human	Australia	Campylobacter coli	825	ST-828 complex	https://www.ncbi.nlm.nih.gov/sra/SRR9974768
Ac_18S1016H1	2018	SRR9974769	PRJNA560409	Human	Australia	Campylobacter coli	1181	ST-828 complex	https://www.ncbi.nlm.nih.gov/sra/SRR9974769
Ac_18T1001H1	2018	SRR9974722	PRJNA560409	Human	Australia	Campylobacter coli	1181	ST-828 complex	https://www.ncbi.nlm.nih.gov/sra/SRR9974722
Ac_18T1017H1	2018	SRR9974757	PRJNA560409	Human	Australia	Campylobacter coli	1016	ST-828 complex	https://www.ncbi.nlm.nih.gov/sra/SRR9974757
Ac_18V1081H1	2018	SRR9974690	PRJNA560409	Human	Australia	Campylobacter coli	827	ST-828 complex	https://www.ncbi.nlm.nih.gov/sra/SRR9974690
Ac_18W1001H1	2018	SRR9974683	PRJNA560409	Human	Australia	Campylobacter coli	1181	ST-828 complex	https://www.ncbi.nlm.nih.gov/sra/SRR9974683
Ac_18W1005H1	2018	SRR9974687	PRJNA560409	Human	Australia	Campylobacter coli	825	ST-828 complex	https://www.ncbi.nlm.nih.gov/sra/SRR9974687
Ac_18W1010H1	2018	SRR9974679	PRJNA560409	Human	Australia	Campylobacter coli	1181	ST-828 complex	https://www.ncbi.nlm.nih.gov/sra/SRR9974679
Ac_18W1013H1	2018	SRR9974705	PRJNA560409	Human	Australia	Campylobacter coli	9419		https://www.ncbi.nlm.nih.gov/sra/SRR9974705
Ac_18W1017H1	2018	SRR9974701	PRJNA560409	Human	Australia	Campylobacter coli	9419		https://www.ncbi.nlm.nih.gov/sra/SRR9974701
Ac_18W1021H1	2018	SRR9974707	PRJNA560409	Human	Australia	Campylobacter coli	1666	ST-828 complex	https://www.ncbi.nlm.nih.gov/sra/SRR9974707
Ac_18W1026H1	2018	SRR9974667	PRJNA560409	Human	Australia	Campylobacter coli	860	ST-828 complex	https://www.ncbi.nlm.nih.gov/sra/SRR9974667
Ac_18W1027H1	2018	SRR9974776	PRJNA560409	Human	Australia	Campylobacter coli	8323	ST-828 complex	https://www.ncbi.nlm.nih.gov/sra/SRR9974776
Ac_19W1005H1	2019	SRR9974745	PRJNA560409	Human	Australia	Campylobacter coli	9969		https://www.ncbi.nlm.nih.gov/sra/SRR9974745
Aj_18A1037H1	2018	SRR9974819	PRJNA560409	Human	Australia	Campylobacter jejuni	6788	ST-1332 complex	https://www.ncbi.nlm.nih.gov/sra/SRR9974819
Aj_18A1038H1	2018	SRR9974818	PRJNA560409	Human	Australia	Campylobacter jejuni	2398		https://www.ncbi.nlm.nih.gov/sra/SRR9974818
Aj_18A1039H1	2018	SRR9974811	PRJNA560409	Human	Australia	Campylobacter jejuni	50	ST-21 complex	https://www.ncbi.nlm.nih.gov/sra/SRR9974811
Aj_18A1040H1	2018	SRR9974810	PRJNA560409	Human	Australia	Campylobacter jejuni	50	ST-21 complex	https://www.ncbi.nlm.nih.gov/sra/SRR9974810
Aj_18A1041H1	2018	SRR9974802	PRJNA560409	Human	Australia	Campylobacter jejuni	2398		https://www.ncbi.nlm.nih.gov/sra/SRR9974802
Aj_18A1043H1	2018	SRR9974803	PRJNA560409	Human	Australia	Campylobacter jejuni	50	ST-21 complex	https://www.ncbi.nlm.nih.gov/sra/SRR9974803
Aj_18A1044H1	2018	SRR9974800	PRJNA560409	Human	Australia	Campylobacter jejuni	48	ST-48 complex	https://www.ncbi.nlm.nih.gov/sra/SRR9974800
Aj_18A1045H1	2018	SRR9974801	PRJNA560409	Human	Australia	Campylobacter jejuni	658	ST-658 complex	https://www.ncbi.nlm.nih.gov/sra/SRR9974801
Aj_18A1047H1	2018	SRR9974806	PRJNA560409	Human	Australia	Campylobacter jejuni	2398		https://www.ncbi.nlm.nih.gov/sra/SRR9974806
Aj_18A1049H1	2018	SRR9974807	PRJNA560409	Human	Australia	Campylobacter jejuni	583	ST-45 complex	https://www.ncbi.nlm.nih.gov/sra/SRR9974807
Aj_18A1050H1	2018	SRR9974804	PRJNA560409	Human	Australia	Campylobacter jejuni	257	ST-257 complex	https://www.ncbi.nlm.nih.gov/sra/SRR9974804
Aj_18A1052H1	2018	SRR9974805	PRJNA560409	Human	Australia	Campylobacter jejuni	50	ST-21 complex	https://www.ncbi.nlm.nih.gov/sra/SRR9974805
Aj_18A1053H1	2018	SRR9974794	PRJNA560409	Human	Australia	Campylobacter jejuni	21	ST-21 complex	https://www.ncbi.nlm.nih.gov/sra/SRR9974794
Aj_18A1056H1	2018	SRR9974795	PRJNA560409	Human	Australia	Campylobacter jejuni	128	ST-45 complex	https://www.ncbi.nlm.nih.gov/sra/SRR9974795
Aj_18A1057H1	2018	SRR9974786	PRJNA560409	Human	Australia	Campylobacter jejuni	658	ST-658 complex	https://www.ncbi.nlm.nih.gov/sra/SRR9974786
Aj_18A1058H1	2018	SRR9974785	PRJNA560409	Human	Australia	Campylobacter jejuni	46	ST-206 complex	https://www.ncbi.nlm.nih.gov/sra/SRR9974785
Aj_18D1002H1	2018	SRR9974747	PRJNA560409	Human	Australia	Campylobacter jejuni	48	ST-48 complex	https://www.ncbi.nlm.nih.gov/sra/SRR9974747
Aj_18N1111H1	2018	SRR9974741	PRJNA560409	Human	Australia	Campylobacter jejuni	4010		https://www.ncbi.nlm.nih.gov/sra/SRR9974741
Aj_18N1113H1	2018	SRR9974743	PRJNA560409	Human	Australia	Campylobacter jejuni	46	ST-206 complex	https://www.ncbi.nlm.nih.gov/sra/SRR9974743
Aj_18N1114H1	2018	SRR9974742	PRJNA560409	Human	Australia	Campylobacter jejuni	257	ST-257 complex	https://www.ncbi.nlm.nih.gov/sra/SRR9974742
Aj_18N1115H1	2018	SRR9974749	PRJNA560409	Human	Australia	Campylobacter jejuni	528	ST-354 complex	https://www.ncbi.nlm.nih.gov/sra/SRR9974749
Aj_18N1116H1	2018	SRR9974748	PRJNA560409	Human	Australia	Campylobacter jejuni	257	ST-257 complex	https://www.ncbi.nlm.nih.gov/sra/SRR9974748
Aj_18N1117H1	2018	SRR9974715	PRJNA560409	Human	Australia	Campylobacter jejuni	50	ST-21 complex	https://www.ncbi.nlm.nih.gov/sra/SRR9974715
Aj_18N1125H1	2019	SRR9974713	PRJNA560409	Human	Australia	Campylobacter jejuni	528	ST-354 complex	https://www.ncbi.nlm.nih.gov/sra/SRR9974713
Aj_18N1126H1	2019	SRR9974714	PRJNA560409	Human	Australia	Campylobacter jejuni	42	ST-42 complex	https://www.ncbi.nlm.nih.gov/sra/SRR9974714
Aj_18N1127H1	2019	SRR9974711	PRJNA560409	Human	Australia	Campylobacter jejuni	50	ST-21 complex	https://www.ncbi.nlm.nih.gov/sra/SRR9974711
Aj_18N1128H1	2019	SRR9974712	PRJNA560409	Human	Australia	Campylobacter jejuni	696	ST-1332 complex	https://www.ncbi.nlm.nih.gov/sra/SRR9974712
Aj_18N1129H1	2019	SRR9974709	PRJNA560409	Human	Australia	Campylobacter jejuni	2343	ST-48 complex	https://www.ncbi.nlm.nih.gov/sra/SRR9974709
Aj_18N1131H1	2019	SRR9974717	PRJNA560409	Human	Australia	Campylobacter jejuni	658	ST-658 complex	https://www.ncbi.nlm.nih.gov/sra/SRR9974717
Aj_18N1132H1	2019	SRR9974718	PRJNA560409	Human	Australia	Campylobacter jejuni	50	ST-21 complex	https://www.ncbi.nlm.nih.gov/sra/SRR9974718
Aj_18N1133H1	2019	SRR9974694	PRJNA560409	Human	Australia	Campylobacter jejuni	50	ST-21 complex	https://www.ncbi.nlm.nih.gov/sra/SRR9974694
Aj_18N1134H1	2019	SRR9974693	PRJNA560409	Human	Australia	Campylobacter jejuni	4684		https://www.ncbi.nlm.nih.gov/sra/SRR9974693
Aj_18N1135H1	2019	SRR9974692	PRJNA560409	Human	Australia	Campylobacter jejuni	51	ST-443 complex	https://www.ncbi.nlm.nih.gov/sra/SRR9974692
Aj_18N1138H1	2019	SRR9974691	PRJNA560409	Human	Australia	Campylobacter jejuni	21	ST-21 complex	https://www.ncbi.nlm.nih.gov/sra/SRR9974691
Aj_18Q1092H1	2018	SRR9974787	PRJNA560409	Human	Australia	Campylobacter jejuni	933	ST-403 complex	https://www.ncbi.nlm.nih.gov/sra/SRR9974787
Aj_18Q1095H1	2018	SRR9974775	PRJNA560409	Human	Australia	Campylobacter jejuni	2083		https://www.ncbi.nlm.nih.gov/sra/SRR9974775
Aj_18Q1104H1	2018	SRR9974774	PRJNA560409	Human	Australia	Campylobacter jejuni	583	ST-45 complex	https://www.ncbi.nlm.nih.gov/sra/SRR9974774
Aj_18Q1112H1	2018	SRR9974830	PRJNA560409	Human	Australia	Campylobacter jejuni	128	ST-45 complex	https://www.ncbi.nlm.nih.gov/sra/SRR9974830
Aj_18Q1122H1	2018	SRR9974825	PRJNA560409	Human	Australia	Campylobacter jejuni	692	ST-692 complex	https://www.ncbi.nlm.nih.gov/sra/SRR9974825
Aj_18Q1124H1	2018	SRR9974823	PRJNA560409	Human	Australia	Campylobacter jejuni	50	ST-21 complex	https://www.ncbi.nlm.nih.gov/sra/SRR9974823
Aj_18Q1126H1	2018	SRR9974824	PRJNA560409	Human	Australia	Campylobacter jejuni	2398		https://www.ncbi.nlm.nih.gov/sra/SRR9974824
Aj_18Q1127H1	2018	SRR9974821	PRJNA560409	Human	Australia	Campylobacter jejuni	50	ST-21 complex	https://www.ncbi.nlm.nih.gov/sra/SRR9974821
Aj_18Q1138H1	2018	SRR9974736	PRJNA560409	Human	Australia	Campylobacter jejuni	2083		https://www.ncbi.nlm.nih.gov/sra/SRR9974736
Aj_18Q1140H1	2018	SRR9974735	PRJNA560409	Human	Australia	Campylobacter jejuni	2343	ST-48 complex	https://www.ncbi.nlm.nih.gov/sra/SRR9974735
Aj_18S1001H1	2018	SRR9974734	PRJNA560409	Human	Australia	Campylobacter jejuni	528	ST-354 complex	https://www.ncbi.nlm.nih.gov/sra/SRR9974734
Aj_18S1002H1	2018	SRR9974733	PRJNA560409	Human	Australia	Campylobacter jejuni	48	ST-48 complex	https://www.ncbi.nlm.nih.gov/sra/SRR9974733
Aj_18S1003H1	2018	SRR9974732	PRJNA560409	Human	Australia	Campylobacter jejuni	48	ST-48 complex	https://www.ncbi.nlm.nih.gov/sra/SRR9974732
Aj_18S1004H1	2018	SRR9974731	PRJNA560409	Human	Australia	Campylobacter jejuni	48	ST-48 complex	https://www.ncbi.nlm.nih.gov/sra/SRR9974731
Aj_18S1006H1	2018	SRR9974729	PRJNA560409	Human	Australia	Campylobacter jejuni	50	ST-21 complex	https://www.ncbi.nlm.nih.gov/sra/SRR9974729
Aj_18S1007H1	2018	SRR9974738	PRJNA560409	Human	Australia	Campylobacter jejuni	658	ST-658 complex	https://www.ncbi.nlm.nih.gov/sra/SRR9974738
Aj_18S1008H1	2018	SRR9974737	PRJNA560409	Human	Australia	Campylobacter jejuni	5221		https://www.ncbi.nlm.nih.gov/sra/SRR9974737
Aj_18S1009H1	2018	SRR9974762	PRJNA560409	Human	Australia	Campylobacter jejuni	9965		https://www.ncbi.nlm.nih.gov/sra/SRR9974762
Aj_18S1010H1	2018	SRR9974763	PRJNA560409	Human	Australia	Campylobacter jejuni	528	ST-354 complex	https://www.ncbi.nlm.nih.gov/sra/SRR9974763
Aj_18S1012H1	2018	SRR9974765	PRJNA560409	Human	Australia	Campylobacter jejuni	538	ST-45 complex	https://www.ncbi.nlm.nih.gov/sra/SRR9974765
Aj_18S1013H1	2018	SRR9974766	PRJNA560409	Human	Australia	Campylobacter jejuni	50	ST-21 complex	https://www.ncbi.nlm.nih.gov/sra/SRR9974766
Aj_18S1014H1	2018	SRR9974767	PRJNA560409	Human	Australia	Campylobacter jejuni	50	ST-21 complex	https://www.ncbi.nlm.nih.gov/sra/SRR9974767
Aj_18S1017H1	2018	SRR9974770	PRJNA560409	Human	Australia	Campylobacter jejuni	4896	ST-353 complex	https://www.ncbi.nlm.nih.gov/sra/SRR9974770
Aj_18S1018H1	2018	SRR9974771	PRJNA560409	Human	Australia	Campylobacter jejuni	128	ST-45 complex	https://www.ncbi.nlm.nih.gov/sra/SRR9974771
Aj_18S1019H1	2018	SRR9974720	PRJNA560409	Human	Australia	Campylobacter jejuni	50	ST-21 complex	https://www.ncbi.nlm.nih.gov/sra/SRR9974720
Aj_18S1020H1	2018	SRR9974719	PRJNA560409	Human	Australia	Campylobacter jejuni	567	ST-22 complex	https://www.ncbi.nlm.nih.gov/sra/SRR9974719

Isolate no.	Year	Accession no.	BioProject no.	Source	Country	Species	Sequence type	Clonal complex	SRA hyperlink
Aj_18T1002H1	2018	SRR9974721	PRJNA560409	Human	Australia	Campylobacter jejuni	528	ST-354 complex	https://www.ncbi.nlm.nih.gov/sra/SRR9974721
Aj_18T1003H1	2018	SRR9974724	PRJNA560409	Human	Australia	Campylobacter jejuni	190	ST-21 complex	https://www.ncbi.nlm.nih.gov/sra/SRR9974724
Aj_18T1004H1	2018	SRR9974723	PRJNA560409	Human	Australia	Campylobacter jejuni	50	ST-21 complex	https://www.ncbi.nlm.nih.gov/sra/SRR9974723
Aj_18T1005H1	2018	SRR9974726	PRJNA560409	Human	Australia	Campylobacter jejuni	2398		https://www.ncbi.nlm.nih.gov/sra/SRR9974726
Aj_18T1006H1	2018	SRR9974725	PRJNA560409	Human	Australia	Campylobacter jejuni	567	ST-22 complex	https://www.ncbi.nlm.nih.gov/sra/SRR9974725
Aj_18T1007H1	2018	SRR9974728	PRJNA560409	Human	Australia	Campylobacter jejuni	45	ST-45 complex	https://www.ncbi.nlm.nih.gov/sra/SRR9974728
Aj_18T1008H1	2018	SRR9974727	PRJNA560409	Human	Australia	Campylobacter jejuni	50	ST-21 complex	https://www.ncbi.nlm.nih.gov/sra/SRR9974727
Aj_18T1009H1	2018	SRR9974754	PRJNA560409	Human	Australia	Campylobacter jejuni	42	ST-42 complex	https://www.ncbi.nlm.nih.gov/sra/SRR9974754
Aj_18T1011H1	2018	SRR9974755	PRJNA560409	Human	Australia	Campylobacter jejuni	991	ST-692 complex	https://www.ncbi.nlm.nih.gov/sra/SRR9974755
Aj_18T1012H1	2018	SRR9974752	PRJNA560409	Human	Australia	Campylobacter jejuni	528	ST-354 complex	https://www.ncbi.nlm.nih.gov/sra/SRR9974752
Aj_18T1013H1	2018	SRR9974753	PRJNA560409	Human	Australia	Campylobacter jejuni	190	ST-21 complex	https://www.ncbi.nlm.nih.gov/sra/SRR9974753
Aj_18T1014H1	2018	SRR9974758	PRJNA560409	Human	Australia	Campylobacter jejuni	528	ST-354 complex	https://www.ncbi.nlm.nih.gov/sra/SRR9974758
Aj_18T1015H1	2018	SRR9974759	PRJNA560409	Human	Australia	Campylobacter jejuni	535	ST-460 complex	https://www.ncbi.nlm.nih.gov/sra/SRR9974759
Aj_18T1016H1	2018	SRR9974756	PRJNA560409	Human	Australia	Campylobacter jejuni	50	ST-21 complex	https://www.ncbi.nlm.nih.gov/sra/SRR9974756
Aj_18T1018H1	2018	SRR9974760	PRJNA560409	Human	Australia	Campylobacter jejuni	190	ST-21 complex	https://www.ncbi.nlm.nih.gov/sra/SRR9974760
Aj_18T1019H1	2018	SRR9974761	PRJNA560409	Human	Australia	Campylobacter jejuni	538	ST-45 complex	https://www.ncbi.nlm.nih.gov/sra/SRR9974761
Aj_18T1020H1	2018	SRR9974684	PRJNA560409	Human	Australia	Campylobacter jejuni	190	ST-21 complex	https://www.ncbi.nlm.nih.gov/sra/SRR9974684
Aj_18V1077H1	2018	SRR9974698	PRJNA560409	Human	Australia	Campylobacter jejuni	9966		https://www.ncbi.nlm.nih.gov/sra/SRR9974698
Aj_18V1078H1	2018	SRR9974697	PRJNA560409	Human	Australia	Campylobacter jejuni	464	ST-464 complex	https://www.ncbi.nlm.nih.gov/sra/SRR9974697
Aj_18V1079H1	2018	SRR9974696	PRJNA560409	Human	Australia	Campylobacter jejuni	567	ST-22 complex	https://www.ncbi.nlm.nih.gov/sra/SRR9974696
Aj_18V1080H1	2018	SRR9974695	PRJNA560409	Human	Australia	Campylobacter jejuni	520	ST-21 complex	https://www.ncbi.nlm.nih.gov/sra/SRR9974695
Aj_18V1082H1	2018	SRR9974689	PRJNA560409	Human	Australia	Campylobacter jejuni	257	ST-257 complex	https://www.ncbi.nlm.nih.gov/sra/SRR9974689
Aj_18V1083H1	2018	SRR9974675	PRJNA560409	Human	Australia	Campylobacter jejuni	567	ST-22 complex	https://www.ncbi.nlm.nih.gov/sra/SRR9974675
Aj_18V1084H1	2018	SRR9974676	PRJNA560409	Human	Australia	Campylobacter jejuni	567	ST-22 complex	https://www.ncbi.nlm.nih.gov/sra/SRR9974676
Aj_18V1085H1	2018	SRR9974677	PRJNA560409	Human	Australia	Campylobacter jejuni	48	ST-48 complex	https://www.ncbi.nlm.nih.gov/sra/SRR9974677
Aj_18V1086H1	2018	SRR9974678	PRJNA560409	Human	Australia	Campylobacter jejuni	257	ST-257 complex	https://www.ncbi.nlm.nih.gov/sra/SRR9974678
Aj_18V1087H1	2018	SRR9974671	PRJNA560409	Human	Australia	Campylobacter jejuni	2083		https://www.ncbi.nlm.nih.gov/sra/SRR9974671
Aj_18V1088H1	2018	SRR9974672	PRJNA560409	Human	Australia	Campylobacter jejuni	538	ST-45 complex	https://www.ncbi.nlm.nih.gov/sra/SRR9974672
Aj_18V1089H1	2018	SRR9974673	PRJNA560409	Human	Australia	Campylobacter jejuni	61	ST-61 complex	https://www.ncbi.nlm.nih.gov/sra/SRR9974673
Aj_18V1090H1	2018	SRR9974674	PRJNA560409	Human	Australia	Campylobacter jejuni	50	ST-21 complex	https://www.ncbi.nlm.nih.gov/sra/SRR9974674
Aj_18V1091H1	2018	SRR9974669	PRJNA560409	Human	Australia	Campylobacter jejuni	137	ST-45 complex	https://www.ncbi.nlm.nih.gov/sra/SRR9974669
Aj_18V1092H1	2018	SRR9974670	PRJNA560409	Human	Australia	Campylobacter jejuni	50	ST-21 complex	https://www.ncbi.nlm.nih.gov/sra/SRR9974670
Aj_18V1093H1	2018	SRR9974813	PRJNA560409	Human	Australia	Campylobacter jejuni	567	ST-22 complex	https://www.ncbi.nlm.nih.gov/sra/SRR9974813
Aj_18V1094H1	2018	SRR9974812	PRJNA560409	Human	Australia	Campylobacter jejuni	2398		https://www.ncbi.nlm.nih.gov/sra/SRR9974812
Aj_18V1095H1	2018	SRR9974815	PRJNA560409	Human	Australia	Campylobacter jejuni	50	ST-21 complex	https://www.ncbi.nlm.nih.gov/sra/SRR9974815
Aj_18V1096H1	2018	SRR9974814	PRJNA560409	Human	Australia	Campylobacter jejuni	21	ST-21 complex	https://www.ncbi.nlm.nih.gov/sra/SRR9974814
Aj_18W1002H1	2018	SRR9974682	PRJNA560409	Human	Australia	Campylobacter jejuni	528	ST-354 complex	https://www.ncbi.nlm.nih.gov/sra/SRR9974682
Aj_18W1003H1	2018	SRR9974681	PRJNA560409	Human	Australia	Campylobacter jejuni	190	ST-21 complex	https://www.ncbi.nlm.nih.gov/sra/SRR9974681
Aj_18W1004H1	2018	SRR9974688	PRJNA560409	Human	Australia	Campylobacter jejuni	528	ST-354 complex	https://www.ncbi.nlm.nih.gov/sra/SRR9974688
Aj_18W1007H1	2018	SRR9974686	PRJNA560409	Human	Australia	Campylobacter jejuni	51	ST-443 complex	https://www.ncbi.nlm.nih.gov/sra/SRR9974686
Aj_18W1008H1	2018	SRR9974685	PRJNA560409	Human	Australia	Campylobacter jejuni	2947	ST-257 complex	https://www.ncbi.nlm.nih.gov/sra/SRR9974685
Aj_18W1009H1	2018	SRR9974680	PRJNA560409	Human	Australia	Campylobacter jejuni	50	ST-21 complex	https://www.ncbi.nlm.nih.gov/sra/SRR9974680
Aj_18W1011H1	2018	SRR9974703	PRJNA560409	Human	Australia	Campylobacter jejuni	9967	ST-464 complex	https://www.ncbi.nlm.nih.gov/sra/SRR9974703
Aj_18W1012H1	2018	SRR9974704	PRJNA560409	Human	Australia	Campylobacter jejuni	528	ST-354 complex	https://www.ncbi.nlm.nih.gov/sra/SRR9974704
Aj_18W1014H1	2018	SRR9974706	PRJNA560409	Human	Australia	Campylobacter jejuni	48	ST-48 complex	https://www.ncbi.nlm.nih.gov/sra/SRR9974706
Aj_18W1015H1	2018	SRR9974699	PRJNA560409	Human	Australia	Campylobacter jejuni	52	ST-52 complex	https://www.ncbi.nlm.nih.gov/sra/SRR9974699
Aj_18W1016H1	2018	SRR9974700	PRJNA560409	Human	Australia	Campylobacter jejuni	528	ST-354 complex	https://www.ncbi.nlm.nih.gov/sra/SRR9974700
Aj_18W1018H1	2018	SRR9974702	PRJNA560409	Human	Australia	Campylobacter jejuni	50	ST-21 complex	https://www.ncbi.nlm.nih.gov/sra/SRR9974702
Aj_18W1022H1	2018	SRR9974708	PRJNA560409	Human	Australia	Campylobacter jejuni	50	ST-21 complex	https://www.ncbi.nlm.nih.gov/sra/SRR9974708
Aj_18W1023H1	2018	SRR9974780	PRJNA560409	Human	Australia	Campylobacter jejuni	7323		https://www.ncbi.nlm.nih.gov/sra/SRR9974780
Aj_18W1024H1	2018	SRR9974779	PRJNA560409	Human	Australia	Campylobacter jejuni	2247		https://www.ncbi.nlm.nih.gov/sra/SRR9974779
Aj_18W1025H1	2018	SRR9974782	PRJNA560409	Human	Australia	Campylobacter jejuni	305	ST-574 complex	https://www.ncbi.nlm.nih.gov/sra/SRR9974782
Aj_18W1027H2	2018	SRR9974820	PRJNA560409	Human	Australia	Campylobacter jejuni	859	ST-443 complex	https://www.ncbi.nlm.nih.gov/sra/SRR9974820
Aj_18W1028H1	2018	SRR9974778	PRJNA560409	Human	Australia	Campylobacter jejuni	61	ST-61 complex	https://www.ncbi.nlm.nih.gov/sra/SRR9974778
Aj_18W1029H1	2018	SRR9974777	PRJNA560409	Human	Australia	Campylobacter jejuni	7323		https://www.ncbi.nlm.nih.gov/sra/SRR9974777
Aj_18W1030H1	2018	SRR9974739	PRJNA560409	Human	Australia	Campylobacter jejuni	305	ST-574 complex	https://www.ncbi.nlm.nih.gov/sra/SRR9974739
Aj_18W1031H1	2018	SRR9974668	PRJNA560409	Human	Australia	Campylobacter jejuni	7323		https://www.ncbi.nlm.nih.gov/sra/SRR9974668
Aj_19A1001H1	2018	SRR9974784	PRJNA560409	Human	Australia	Campylobacter jejuni	48	ST-48 complex	https://www.ncbi.nlm.nih.gov/sra/SRR9974784
Aj_19A1002H1	2018	SRR9974783	PRJNA560409	Human	Australia	Campylobacter jejuni	9608	ST-443 complex	https://www.ncbi.nlm.nih.gov/sra/SRR9974783
Aj_19D1001H1	2019	SRR9974798	PRJNA560409	Human	Australia	Campylobacter jejuni	528	ST-354 complex	https://www.ncbi.nlm.nih.gov/sra/SRR9974798
Aj_19D1002H1	2018	SRR9974799	PRJNA560409	Human	Australia	Campylobacter jejuni	45	ST-45 complex	https://www.ncbi.nlm.nih.gov/sra/SRR9974799
Aj_19D1003H1	2018	SRR9974796	PRJNA560409	Human	Australia	Campylobacter jejuni	2107	ST-52 complex	https://www.ncbi.nlm.nih.gov/sra/SRR9974796
Aj_19D1004H1	2019	SRR9974797	PRJNA560409	Human	Australia	Campylobacter jejuni	1078	ST-443 complex	https://www.ncbi.nlm.nih.gov/sra/SRR9974797
Aj_19D1005H1	2019	SRR9974790	PRJNA560409	Human	Australia	Campylobacter jejuni	528	ST-354 complex	https://www.ncbi.nlm.nih.gov/sra/SRR9974790
Aj_19D1006H1	2019	SRR9974791	PRJNA560409	Human	Australia	Campylobacter jejuni	50	ST-21 complex	https://www.ncbi.nlm.nih.gov/sra/SRR9974791
Aj_19W1001H1	2019	SRR9974792	PRJNA560409	Human	Australia	Campylobacter jejuni	9968		https://www.ncbi.nlm.nih.gov/sra/SRR9974792
Aj_19W1002H1	2019	SRR9974793	PRJNA560409	Human	Australia	Campylobacter jejuni	50	ST-21 complex	https://www.ncbi.nlm.nih.gov/sra/SRR9974793
Aj_19W1003H1	2019	SRR9974808	PRJNA560409	Human	Australia	Campylobacter jejuni	1078	ST-443 complex	https://www.ncbi.nlm.nih.gov/sra/SRR9974808
Aj_19W1004H1	2019	SRR9974809	PRJNA560409	Human	Australia	Campylobacter jejuni	61	ST-61 complex	https://www.ncbi.nlm.nih.gov/sra/SRR9974809
Aj_19W1006H1	2019	SRR9974744	PRJNA560409	Human	Australia	Campylobacter jejuni	48	ST-48 complex	https://www.ncbi.nlm.nih.gov/sra/SRR9974744
Aj_19W1007H1	2019	SRR9974789	PRJNA560409	Human	Australia	Campylobacter jejuni	508	ST-508 complex	https://www.ncbi.nlm.nih.gov/sra/SRR9974789
Aj_19W1008H1	2019	SRR9974788	PRJNA560409	Human	Australia	Campylobacter jejuni	50	ST-21 complex	https://www.ncbi.nlm.nih.gov/sra/SRR9974788
Cc_17N3001F1	2017	SRR10537820	PRJNA591966	Chicken	Australia	Campylobacter coli	827	ST-828 complex	https://www.ncbi.nlm.nih.gov/sra/SRR10537820
Cc_17N3015F1	2017	SRR10537814	PRJNA591966	Chicken	Australia	Campylobacter coli	1181	ST-828 complex	https://www.ncbi.nlm.nih.gov/sra/SRR10537814
Cc_17N3018F1	2017	SRR10537812	PRJNA591966	Chicken	Australia	Campylobacter coli	nC06		https://www.ncbi.nlm.nih.gov/sra/SRR10537812
Cc_17N3021F1	2017	SRR10537810	PRJNA591966	Chicken	Australia	Campylobacter coli	4175	ST-828 complex	https://www.ncbi.nlm.nih.gov/sra/SRR10537810
Cc_17N3022F1	2017	SRR10537809	PRJNA591966	Chicken	Australia	Campylobacter coli	1563	ST-828 complex	https://www.ncbi.nlm.nih.gov/sra/SRR10537809
Cc_17N3024F1	2017	SRR10537806	PRJNA591966	Chicken	Australia	Campylobacter coli	10145	ST-828 complex	https://www.ncbi.nlm.nih.gov/sra/SRR10537806
Cc_17N3026F1	2017	SRR10537805	PRJNA591966	Chicken	Australia	Campylobacter coli	825	ST-828 complex	https://www.ncbi.nlm.nih.gov/sra/SRR10537805
Cc_17N3028F1	2017	SRR10537803	PRJNA591966	Chicken	Australia	Campylobacter coli	825	ST-828 complex	https://www.ncbi.nlm.nih.gov/sra/SRR10537803
Cc_17N3044F1	2017	SRR10537792	PRJNA591966	Chicken	Australia	Campylobacter coli	832	ST-828 complex	https://www.ncbi.nlm.nih.gov/sra/SRR10537792
Cc_17N3046F1	2017	SRR10537828	PRJNA591966	Chicken	Australia	Campylobacter coli	827	ST-828 complex	https://www.ncbi.nlm.nih.gov/sra/SRR10537828
Cc_17N3047F1	2017	SRR10537827	PRJNA591966	Chicken	Australia	Campylobacter coli	860	ST-828 complex	https://www.ncbi.nlm.nih.gov/sra/SRR10537827
Cc_17N3048F1	2017	SRR10537826	PRJNA591966	Chicken	Australia	Campylobacter coli	827	ST-828 complex	https://www.ncbi.nlm.nih.gov/sra/SRR10537826
Cc_17N3049F1	2017	SRR10537825	PRJNA591966	Chicken	Australia	Campylobacter coli	1181	ST-828 complex	https://www.ncbi.nlm.nih.gov/sra/SRR10537825
Cc_17N3066F1	2017	SRR10537781	PRJNA591966	Chicken	Australia	Campylobacter coli	9436		https://www.ncbi.nlm.nih.gov/sra/SRR10537781
Cc_17N3067F1	2017	SRR10537780	PRJNA591966	Chicken	Australia	Campylobacter coli	860	ST-828 complex	https://www.ncbi.nlm.nih.gov/sra/SRR10537780

Isolate no.	Year	Accession no.	BioProject no.	Source	Country	Species	Sequence type	Clonal complex	SRA hyperlink
Cc_17N3068F1	2017	SRR10537779	PRJNA591966	Chicken	Australia	Campylobacter coli	4175	ST-828 complex	https://www.ncbi.nlm.nih.gov/sra/SRR10537779
Cc_17N3071F1	2017	SRR10537776	PRJNA591966	Chicken	Australia	Campylobacter coli	10144		https://www.ncbi.nlm.nih.gov/sra/SRR10537776
Cc_17N3079F1	2017	SRR10537771	PRJNA591966	Chicken	Australia	Campylobacter coli	1181	ST-828 complex	https://www.ncbi.nlm.nih.gov/sra/SRR10537771
Cc_17N3084F1	2017	SRR10537769	PRJNA591966	Chicken	Australia	Campylobacter coli	4175	ST-828 complex	https://www.ncbi.nlm.nih.gov/sra/SRR10537769
Cc_17N3092F1	2017	SRR10537764	PRJNA591966	Chicken	Australia	Campylobacter coli	4175	ST-828 complex	https://www.ncbi.nlm.nih.gov/sra/SRR10537764
Cc_17N3093F1	2017	SRR10537763	PRJNA591966	Chicken	Australia	Campylobacter coli	966	ST-828 complex	https://www.ncbi.nlm.nih.gov/sra/SRR10537763
Cc_17N3110F1	2017	SRR10537761	PRJNA591966	Chicken	Australia	Campylobacter coli	966	ST-828 complex	https://www.ncbi.nlm.nih.gov/sra/SRR10537761
Cc_17N3119F1	2017	SRR10537760	PRJNA591966	Chicken	Australia	Campylobacter coli	9436		https://www.ncbi.nlm.nih.gov/sra/SRR10537760
Cc_17N3143F1	2017	SRR10537754	PRJNA591966	Chicken	Australia	Campylobacter coli	1181	ST-828 complex	https://www.ncbi.nlm.nih.gov/sra/SRR10537754
Cc_17N3146F1	2017	SRR10537753	PRJNA591966	Chicken	Australia	Campylobacter coli	1181	ST-828 complex	https://www.ncbi.nlm.nih.gov/sra/SRR10537753
Cc_17N3151F1	2017	SRR10537752	PRJNA591966	Chicken	Australia	Campylobacter coli	9419		https://www.ncbi.nlm.nih.gov/sra/SRR10537752
Cc_17N3172F1	2017	SRR10537748	PRJNA591966	Chicken	Australia	Campylobacter coli	1181	ST-828 complex	https://www.ncbi.nlm.nih.gov/sra/SRR10537748
Cc_17N3180F1	2017	SRR10537744	PRJNA591966	Chicken	Australia	Campylobacter coli	1181	ST-828 complex	https://www.ncbi.nlm.nih.gov/sra/SRR10537744
Cc_17N3181F1	2017	SRR10537743	PRJNA591966	Chicken	Australia	Campylobacter coli	10144		https://www.ncbi.nlm.nih.gov/sra/SRR10537743
Cc_17N3206F1	2017	SRR10537740	PRJNA591966	Chicken	Australia	Campylobacter coli	9435	ST-828 complex	https://www.ncbi.nlm.nih.gov/sra/SRR10537740
Cc_17N3219F1	2017	SRR10537737	PRJNA591966	Chicken	Australia	Campylobacter coli	860	ST-828 complex	https://www.ncbi.nlm.nih.gov/sra/SRR10537737
Cc_17N3227F1	2017	SRR10537735	PRJNA591966	Chicken	Australia	Campylobacter coli	832	ST-828 complex	https://www.ncbi.nlm.nih.gov/sra/SRR10537735
Cc_17N3240F1	2017	SRR10537733	PRJNA591966	Chicken	Australia	Campylobacter coli	1181	ST-828 complex	https://www.ncbi.nlm.nih.gov/sra/SRR10537733
Cc_17N3241F1	2017	SRR10537732	PRJNA591966	Chicken	Australia	Campylobacter coli	9419		https://www.ncbi.nlm.nih.gov/sra/SRR10537732
Cc_17N3243F1	2017	SRR10537730	PRJNA591966	Chicken	Australia	Campylobacter coli	1181	ST-828 complex	https://www.ncbi.nlm.nih.gov/sra/SRR10537730
Cc_17N3245F1	2017	SRR10537729	PRJNA591966	Chicken	Australia	Campylobacter coli	9419		https://www.ncbi.nlm.nih.gov/sra/SRR10537729
Cc_17N3246F1	2017	SRR10537728	PRJNA591966	Chicken	Australia	Campylobacter coli	1181	ST-828 complex	https://www.ncbi.nlm.nih.gov/sra/SRR10537728
Cc_17N3247F1	2017	SRR10537727	PRJNA591966	Chicken	Australia	Campylobacter coli	9419		https://www.ncbi.nlm.nih.gov/sra/SRR10537727
Cc_17N3248F1	2017	SRR10537726	PRJNA591966	Chicken	Australia	Campylobacter coli	1181	ST-828 complex	https://www.ncbi.nlm.nih.gov/sra/SRR10537726
Cc_17N3251F1	2017	SRR10537724	PRJNA591966	Chicken	Australia	Campylobacter coli	832	ST-828 complex	https://www.ncbi.nlm.nih.gov/sra/SRR10537724
Cc_17N3259F1	2017	SRR10537720	PRJNA591966	Chicken	Australia	Campylobacter coli	828	ST-828 complex	https://www.ncbi.nlm.nih.gov/sra/SRR10537720
Cc_17Q3022F1	2017	SRR10537564	PRJNA591966	Chicken	Australia	Campylobacter coli	3985	ST-828 complex	https://www.ncbi.nlm.nih.gov/sra/SRR10537564
Cc_17Q3023F1	2017	SRR10537563	PRJNA591966	Chicken	Australia	Campylobacter coli	1766		https://www.ncbi.nlm.nih.gov/sra/SRR10537563
Cc_17Q3024F1	2017	SRR10537562	PRJNA591966	Chicken	Australia	Campylobacter coli	4175	ST-828 complex	https://www.ncbi.nlm.nih.gov/sra/SRR10537562
Cc_17Q3025F1	2017	SRR10537561	PRJNA591966	Chicken	Australia	Campylobacter coli	1181	ST-828 complex	https://www.ncbi.nlm.nih.gov/sra/SRR10537561
Cc_17Q3026F1	2017	SRR10537560	PRJNA591966	Chicken	Australia	Campylobacter coli	1181	ST-828 complex	https://www.ncbi.nlm.nih.gov/sra/SRR10537560
Cc_17Q3027F1	2017	SRR10537559	PRJNA591966	Chicken	Australia	Campylobacter coli	9436		https://www.ncbi.nlm.nih.gov/sra/SRR10537559
Cc_17Q3028F1	2017	SRR10537558	PRJNA591966	Chicken	Australia	Campylobacter coli	827	ST-828 complex	https://www.ncbi.nlm.nih.gov/sra/SRR10537558
Cc_17Q3030F1	2017	SRR10537555	PRJNA591966	Chicken	Australia	Campylobacter coli	827	ST-828 complex	https://www.ncbi.nlm.nih.gov/sra/SRR10537555
Cc_17Q3031F1	2017	SRR10537554	PRJNA591966	Chicken	Australia	Campylobacter coli	860	ST-828 complex	https://www.ncbi.nlm.nih.gov/sra/SRR10537554
Cc_17Q3032F1	2017	SRR10537553	PRJNA591966	Chicken	Australia	Campylobacter coli	827	ST-828 complex	https://www.ncbi.nlm.nih.gov/sra/SRR10537553
Cc_17Q3033F1	2017	SRR10537552	PRJNA591966	Chicken	Australia	Campylobacter coli	3985	ST-828 complex	https://www.ncbi.nlm.nih.gov/sra/SRR10537552
Cc_17Q3034F1	2017	SRR10537551	PRJNA591966	Chicken	Australia	Campylobacter coli	9436		https://www.ncbi.nlm.nih.gov/sra/SRR10537551
Cc_17Q3035F1	2017	SRR10537550	PRJNA591966	Chicken	Australia	Campylobacter coli	10144		https://www.ncbi.nlm.nih.gov/sra/SRR10537550
Cc_17Q3036F1	2017	SRR10537549	PRJNA591966	Chicken	Australia	Campylobacter coli	1181	ST-828 complex	https://www.ncbi.nlm.nih.gov/sra/SRR10537549
Cc_17Q3037F1	2017	SRR10537548	PRJNA591966	Chicken	Australia	Campylobacter coli	832	ST-828 complex	https://www.ncbi.nlm.nih.gov/sra/SRR10537548
Cc_17Q3038F1	2017	SRR10537547	PRJNA591966	Chicken	Australia	Campylobacter coli	832	ST-828 complex	https://www.ncbi.nlm.nih.gov/sra/SRR10537547
Cc_17Q3041F1	2017	SRR10537544	PRJNA591966	Chicken	Australia	Campylobacter coli	4175	ST-828 complex	https://www.ncbi.nlm.nih.gov/sra/SRR10537544
Cc_17Q3042F1	2017	SRR10537543	PRJNA591966	Chicken	Australia	Campylobacter coli	1181	ST-828 complex	https://www.ncbi.nlm.nih.gov/sra/SRR10537543
Cc_17Q3043F1	2017	SRR10537542	PRJNA591966	Chicken	Australia	Campylobacter coli	827	ST-828 complex	https://www.ncbi.nlm.nih.gov/sra/SRR10537542
Cc_17Q3044F1	2017	SRR10537541	PRJNA591966	Chicken	Australia	Campylobacter coli	10164	ST-828 complex	https://www.ncbi.nlm.nih.gov/sra/SRR10537541
Cc_17Q3045F1	2017	SRR10537540	PRJNA591966	Chicken	Australia	Campylobacter coli	10164	ST-828 complex	https://www.ncbi.nlm.nih.gov/sra/SRR10537540
Cc_17Q3046F1	2017	SRR10537539	PRJNA591966	Chicken	Australia	Campylobacter coli	10164	ST-828 complex	https://www.ncbi.nlm.nih.gov/sra/SRR10537539
Cc_17Q3047F1	2017	SRR10537538	PRJNA591966	Chicken	Australia	Campylobacter coli	10145	ST-828 complex	https://www.ncbi.nlm.nih.gov/sra/SRR10537538
Cc_17Q3050F1	2017	SRR10537535	PRJNA591966	Chicken	Australia	Campylobacter coli	1427	ST-828 complex	https://www.ncbi.nlm.nih.gov/sra/SRR10537535
Cc_17Q3052F1	2017	SRR10537533	PRJNA591966	Chicken	Australia	Campylobacter coli	10145	ST-828 complex	https://www.ncbi.nlm.nih.gov/sra/SRR10537533
Cc_17Q3053F1	2017	SRR10537532	PRJNA591966	Chicken	Australia	Campylobacter coli	9433		https://www.ncbi.nlm.nih.gov/sra/SRR10537532
Cc_17Q3058F1	2017	SRR10537527	PRJNA591966	Chicken	Australia	Campylobacter coli	827	ST-828 complex	https://www.ncbi.nlm.nih.gov/sra/SRR10537527
Cc_17Q3059F1	2017	SRR10537526	PRJNA591966	Chicken	Australia	Campylobacter coli	9419		https://www.ncbi.nlm.nih.gov/sra/SRR10537526
Cc_17Q3063F1	2017	SRR10537521	PRJNA591966	Chicken	Australia	Campylobacter coli	825	ST-828 complex	https://www.ncbi.nlm.nih.gov/sra/SRR10537521
Cc_17Q3065F1	2017	SRR10537519	PRJNA591966	Chicken	Australia	Campylobacter coli	827	ST-828 complex	https://www.ncbi.nlm.nih.gov/sra/SRR10537519
Cc_17Q3068F1	2017	SRR10537516	PRJNA591966	Chicken	Australia	Campylobacter coli	3985	ST-828 complex	https://www.ncbi.nlm.nih.gov/sra/SRR10537516
Cc_17Q3070F1	2017	SRR10537514	PRJNA591966	Chicken	Australia	Campylobacter coli	10144		https://www.ncbi.nlm.nih.gov/sra/SRR10537514
Cc_17Q3073F1	2017	SRR10537510	PRJNA591966	Chicken	Australia	Campylobacter coli	6159		https://www.ncbi.nlm.nih.gov/sra/SRR10537510
Cc_17Q3075F1	2017	SRR10537508	PRJNA591966	Chicken	Australia	Campylobacter coli	860	ST-828 complex	https://www.ncbi.nlm.nih.gov/sra/SRR10537508
Cc_17Q3076F1	2017	SRR10537507	PRJNA591966	Chicken	Australia	Campylobacter coli	10147		https://www.ncbi.nlm.nih.gov/sra/SRR10537507
Cc_17Q3077F1	2017	SRR10537506	PRJNA591966	Chicken	Australia	Campylobacter coli	832	ST-828 complex	https://www.ncbi.nlm.nih.gov/sra/SRR10537506
Cc_17Q3078F1	2017	SRR10537505	PRJNA591966	Chicken	Australia	Campylobacter coli	3985	ST-828 complex	https://www.ncbi.nlm.nih.gov/sra/SRR10537505
Cc_17Q3079F1	2017	SRR10537504	PRJNA591966	Chicken	Australia	Campylobacter coli	3985	ST-828 complex	https://www.ncbi.nlm.nih.gov/sra/SRR10537504
Cc_17Q3081F1	2017	SRR10537502	PRJNA591966	Chicken	Australia	Campylobacter coli	1173	ST-828 complex	https://www.ncbi.nlm.nih.gov/sra/SRR10537502
Cc_17Q3083F2	2017	SRR10537498	PRJNA591966	Chicken	Australia	Campylobacter coli	860	ST-828 complex	https://www.ncbi.nlm.nih.gov/sra/SRR10537498
Cc_17Q3084F2	2017	SRR10537496	PRJNA591966	Chicken	Australia	Campylobacter coli	10148		https://www.ncbi.nlm.nih.gov/sra/SRR10537496
Cc_17Q3085F1	2017	SRR10537495	PRJNA591966	Chicken	Australia	Campylobacter coli	1427	ST-828 complex	https://www.ncbi.nlm.nih.gov/sra/SRR10537495
Cc_17Q3085F2	2017	SRR10537494	PRJNA591966	Chicken	Australia	Campylobacter coli	825	ST-828 complex	https://www.ncbi.nlm.nih.gov/sra/SRR10537494
Cc_17Q3086F2	2017	SRR10537492	PRJNA591966	Chicken	Australia	Campylobacter coli	3985	ST-828 complex	https://www.ncbi.nlm.nih.gov/sra/SRR10537492
Cc_17Q3087F2	2017	SRR10537489	PRJNA591966	Chicken	Australia	Campylobacter coli	10164	ST-828 complex	https://www.ncbi.nlm.nih.gov/sra/SRR10537489
Cc_17Q3089F1	2017	SRR10537486	PRJNA591966	Chicken	Australia	Campylobacter coli	966	ST-828 complex	https://www.ncbi.nlm.nih.gov/sra/SRR10537486
Cc_17Q3089F2	2017	SRR10537485	PRJNA591966	Chicken	Australia	Campylobacter coli	966	ST-828 complex	https://www.ncbi.nlm.nih.gov/sra/SRR10537485
Cc_17Q3096F1	2017	SRR10537478	PRJNA591966	Chicken	Australia	Campylobacter coli	9436		https://www.ncbi.nlm.nih.gov/sra/SRR10537478
Cc_17Q3099F1	2017	SRR10537475	PRJNA591966	Chicken	Australia	Campylobacter coli	9789		https://www.ncbi.nlm.nih.gov/sra/SRR10537475
Cc_17Q3103F1	2017	SRR10537471	PRJNA591966	Chicken	Australia	Campylobacter coli	9419		https://www.ncbi.nlm.nih.gov/sra/SRR10537471
Cc_17Q3104F1	2017	SRR10537470	PRJNA591966	Chicken	Australia	Campylobacter coli	9419		https://www.ncbi.nlm.nih.gov/sra/SRR10537470
Cc_17Q3105F1	2017	SRR10537469	PRJNA591966	Chicken	Australia	Campylobacter coli	1181	ST-828 complex	https://www.ncbi.nlm.nih.gov/sra/SRR10537469
Cc_17Q3106F1	2017	SRR10537467	PRJNA591966	Chicken	Australia	Campylobacter coli	1181	ST-828 complex	https://www.ncbi.nlm.nih.gov/sra/SRR10537467
Cc_17Q3110F2	2017	SRR10537464	PRJNA591966	Chicken	Australia	Campylobacter coli	9419		https://www.ncbi.nlm.nih.gov/sra/SRR10537464
Cc_17Q3112F1	2017	SRR10537462	PRJNA591966	Chicken	Australia	Campylobacter coli	860	ST-828 complex	https://www.ncbi.nlm.nih.gov/sra/SRR10537462
Cc_17Q3115F1	2017	SRR10537459	PRJNA591966	Chicken	Australia	Campylobacter coli	827	ST-828 complex	https://www.ncbi.nlm.nih.gov/sra/SRR10537459
Cc_17Q3116F1	2017	SRR10537458	PRJNA591966	Chicken	Australia	Campylobacter coli	3985	ST-828 complex	https://www.ncbi.nlm.nih.gov/sra/SRR10537458
Cc_17Q3119F1	2017	SRR10538070	PRJNA591966	Chicken	Australia	Campylobacter coli	860	ST-828 complex	https://www.ncbi.nlm.nih.gov/sra/SRR10538070
Cc_17Q3126F1	2017	SRR10538063	PRJNA591966	Chicken	Australia	Campylobacter coli	3985	ST-828 complex	https://www.ncbi.nlm.nih.gov/sra/SRR10538063
Cc_17Q3129F1	2017	SRR10538060	PRJNA591966	Chicken	Australia	Campylobacter coli	10159		https://www.ncbi.nlm.nih.gov/sra/SRR10538060
Cc_17Q3137F1	2017	SRR10538053	PRJNA591966	Chicken	Australia	Campylobacter coli	1181	ST-828 complex	https://www.ncbi.nlm.nih.gov/sra/SRR10538053

Isolate no.	Year	Accession no.	BioProject no.	Source	Country	Species	Sequence type	Clonal complex	SRA hyperlink
Cc_17Q3138F1	2017	SRR10538052	PRJNA591966	Chicken	Australia	Campylobacter coli	832	ST-828 complex	https://www.ncbi.nlm.nih.gov/sra/SRR10538052
Cc_17V3001F1	2017	SRR10537995	PRJNA591966	Chicken	Australia	Campylobacter coli	825	ST-828 complex	https://www.ncbi.nlm.nih.gov/sra/SRR10537995
Cc_17V3002F1	2017	SRR10537994	PRJNA591966	Chicken	Australia	Campylobacter coli	829	ST-828 complex	https://www.ncbi.nlm.nih.gov/sra/SRR10537994
Cc_17V3004F1	2017	SRR10537993	PRJNA591966	Chicken	Australia	Campylobacter coli	825	ST-828 complex	https://www.ncbi.nlm.nih.gov/sra/SRR10537993
Cc_17V3005F1	2017	SRR10537992	PRJNA591966	Chicken	Australia	Campylobacter coli	825	ST-828 complex	https://www.ncbi.nlm.nih.gov/sra/SRR10537992
Cc_17V3006F1	2017	SRR10537991	PRJNA591966	Chicken	Australia	Campylobacter coli	827	ST-828 complex	https://www.ncbi.nlm.nih.gov/sra/SRR10537991
Cc_17V3007F1	2017	SRR10537990	PRJNA591966	Chicken	Australia	Campylobacter coli	10150		https://www.ncbi.nlm.nih.gov/sra/SRR10537990
Cc_17V3010F1	2017	SRR10537987	PRJNA591966	Chicken	Australia	Campylobacter coli	825	ST-828 complex	https://www.ncbi.nlm.nih.gov/sra/SRR10537987
Cc_17V3011F1	2017	SRR10537986	PRJNA591966	Chicken	Australia	Campylobacter coli	1181	ST-828 complex	https://www.ncbi.nlm.nih.gov/sra/SRR10537986
Cc_17V3012F1	2017	SRR10537984	PRJNA591966	Chicken	Australia	Campylobacter coli	1181	ST-828 complex	https://www.ncbi.nlm.nih.gov/sra/SRR10537984
Cc_17V3013F1	2017	SRR10537982	PRJNA591966	Chicken	Australia	Campylobacter coli	827	ST-828 complex	https://www.ncbi.nlm.nih.gov/sra/SRR10537982
Cc_17V3014F1	2017	SRR10537981	PRJNA591966	Chicken	Australia	Campylobacter coli	829	ST-828 complex	https://www.ncbi.nlm.nih.gov/sra/SRR10537981
Cc_17V3016F1	2017	SRR10537979	PRJNA591966	Chicken	Australia	Campylobacter coli	825	ST-828 complex	https://www.ncbi.nlm.nih.gov/sra/SRR10537979
Cc_17V3017F1	2017	SRR10537978	PRJNA591966	Chicken	Australia	Campylobacter coli	825	ST-828 complex	https://www.ncbi.nlm.nih.gov/sra/SRR10537978
Cc_17V3019F1	2017	SRR10537976	PRJNA591966	Chicken	Australia	Campylobacter coli	1181	ST-828 complex	https://www.ncbi.nlm.nih.gov/sra/SRR10537976
Cc_17V3020F1	2017	SRR10537975	PRJNA591966	Chicken	Australia	Campylobacter coli	4175	ST-828 complex	https://www.ncbi.nlm.nih.gov/sra/SRR10537975
Cc_17V3021F1	2017	SRR10537973	PRJNA591966	Chicken	Australia	Campylobacter coli	9419		https://www.ncbi.nlm.nih.gov/sra/SRR10537973
Cc_17V3023F1	2017	SRR10537971	PRJNA591966	Chicken	Australia	Campylobacter coli	9912		https://www.ncbi.nlm.nih.gov/sra/SRR10537971
Cc_17V3024F1	2017	SRR10537970	PRJNA591966	Chicken	Australia	Campylobacter coli	825	ST-828 complex	https://www.ncbi.nlm.nih.gov/sra/SRR10537970
Cc_17V3031F2	2017	SRR10537965	PRJNA591966	Chicken	Australia	Campylobacter coli	10166		https://www.ncbi.nlm.nih.gov/sra/SRR10537965
Cc_17V3034F1	2017	SRR10537960	PRJNA591966	Chicken	Australia	Campylobacter coli	825	ST-828 complex	https://www.ncbi.nlm.nih.gov/sra/SRR10537960
Cc_17V3036F1	2017	SRR10537958	PRJNA591966	Chicken	Australia	Campylobacter coli	825	ST-828 complex	https://www.ncbi.nlm.nih.gov/sra/SRR10537958
Cc_17V3037F1	2017	SRR10537957	PRJNA591966	Chicken	Australia	Campylobacter coli	825	ST-828 complex	https://www.ncbi.nlm.nih.gov/sra/SRR10537957
Cc_17V3038F1	2017	SRR10537956	PRJNA591966	Chicken	Australia	Campylobacter coli	825	ST-828 complex	https://www.ncbi.nlm.nih.gov/sra/SRR10537956
Cc_17V3039F1	2017	SRR10537955	PRJNA591966	Chicken	Australia	Campylobacter coli	1181	ST-828 complex	https://www.ncbi.nlm.nih.gov/sra/SRR10537955
Cc_17V3040F1	2017	SRR10537954	PRJNA591966	Chicken	Australia	Campylobacter coli	nC05		https://www.ncbi.nlm.nih.gov/sra/SRR10537954
Cc_17V3041F1	2017	SRR10537953	PRJNA591966	Chicken	Australia	Campylobacter coli	825	ST-828 complex	https://www.ncbi.nlm.nih.gov/sra/SRR10537953
Cc_17V3044F1	2017	SRR10537950	PRJNA591966	Chicken	Australia	Campylobacter coli	825	ST-828 complex	https://www.ncbi.nlm.nih.gov/sra/SRR10537950
Cc_17V3046F1	2017	SRR10537949	PRJNA591966	Chicken	Australia	Campylobacter coli	825	ST-828 complex	https://www.ncbi.nlm.nih.gov/sra/SRR10537949
Cc_17V3047F1	2017	SRR10537948	PRJNA591966	Chicken	Australia	Campylobacter coli	825	ST-828 complex	https://www.ncbi.nlm.nih.gov/sra/SRR10537948
Cc_17V3048F1	2017	SRR10537947	PRJNA591966	Chicken	Australia	Campylobacter coli	1181	ST-828 complex	https://www.ncbi.nlm.nih.gov/sra/SRR10537947
Cc_17V3049F1	2017	SRR10537946	PRJNA591966	Chicken	Australia	Campylobacter coli	825	ST-828 complex	https://www.ncbi.nlm.nih.gov/sra/SRR10537946
Cc_17V3051F1	2017	SRR10537945	PRJNA591966	Chicken	Australia	Campylobacter coli	825	ST-828 complex	https://www.ncbi.nlm.nih.gov/sra/SRR10537945
Cc_17V3055F2	2017	SRR10537942	PRJNA591966	Chicken	Australia	Campylobacter coli	1181	ST-828 complex	https://www.ncbi.nlm.nih.gov/sra/SRR10537942
Cc_17V3056F1	2017	SRR10537941	PRJNA591966	Chicken	Australia	Campylobacter coli	825	ST-828 complex	https://www.ncbi.nlm.nih.gov/sra/SRR10537941
Cc_17V3058F1	2017	SRR10537938	PRJNA591966	Chicken	Australia	Campylobacter coli	1181	ST-828 complex	https://www.ncbi.nlm.nih.gov/sra/SRR10537938
Cc_17V3059F1	2017	SRR10537936	PRJNA591966	Chicken	Australia	Campylobacter coli	1181	ST-828 complex	https://www.ncbi.nlm.nih.gov/sra/SRR10537936
Cc_17V3060F2	2017	SRR10537934	PRJNA591966	Chicken	Australia	Campylobacter coli	825	ST-828 complex	https://www.ncbi.nlm.nih.gov/sra/SRR10537934
Cc_18A3001F1	2018	SRR10537791	PRJNA591966	Chicken	Australia	Campylobacter coli	9420		https://www.ncbi.nlm.nih.gov/sra/SRR10537791
Cc_18A3002F1	2018	SRR10537790	PRJNA591966	Chicken	Australia	Campylobacter coli	860	ST-828 complex	https://www.ncbi.nlm.nih.gov/sra/SRR10537790
Cc_18A3002F2	2018	SRR10537679	PRJNA591966	Chicken	Australia	Campylobacter coli	860	ST-828 complex	https://www.ncbi.nlm.nih.gov/sra/SRR10537679
Cc_18A3003F1	2018	SRR10537568	PRJNA591966	Chicken	Australia	Campylobacter coli	860	ST-828 complex	https://www.ncbi.nlm.nih.gov/sra/SRR10537568
Cc_18A3004F1	2018	SRR10537457	PRJNA591966	Chicken	Australia	Campylobacter coli	828	ST-828 complex	https://www.ncbi.nlm.nih.gov/sra/SRR10537457
Cc_18A3004F2	2018	SRR10537963	PRJNA591966	Chicken	Australia	Campylobacter coli	828	ST-828 complex	https://www.ncbi.nlm.nih.gov/sra/SRR10537963
Cc_18A3005F1	2018	SRR10537852	PRJNA591966	Chicken	Australia	Campylobacter coli	829	ST-828 complex	https://www.ncbi.nlm.nih.gov/sra/SRR10537852
Cc_18A3005F2	2018	SRR10537819	PRJNA591966	Chicken	Australia	Campylobacter coli	829	ST-828 complex	https://www.ncbi.nlm.nih.gov/sra/SRR10537819
Cc_18A3006F1	2018	SRR10537808	PRJNA591966	Chicken	Australia	Campylobacter coli	860	ST-828 complex	https://www.ncbi.nlm.nih.gov/sra/SRR10537808
Cc_18A3009F1	2018	SRR10537756	PRJNA591966	Chicken	Australia	Campylobacter coli	860	ST-828 complex	https://www.ncbi.nlm.nih.gov/sra/SRR10537756
Cc_18A3009F2	2018	SRR10537745	PRJNA591966	Chicken	Australia	Campylobacter coli	860	ST-828 complex	https://www.ncbi.nlm.nih.gov/sra/SRR10537745
Cc_18A3010F1	2018	SRR10537734	PRJNA591966	Chicken	Australia	Campylobacter coli	827	ST-828 complex	https://www.ncbi.nlm.nih.gov/sra/SRR10537734
Cc_18A3010F2	2018	SRR10537723	PRJNA591966	Chicken	Australia	Campylobacter coli	827	ST-828 complex	https://www.ncbi.nlm.nih.gov/sra/SRR10537723
Cc_18A3013F1	2018	SRR10537690	PRJNA591966	Chicken	Australia	Campylobacter coli	827	ST-828 complex	https://www.ncbi.nlm.nih.gov/sra/SRR10537690
Cc_18A3014F1	2018	SRR10537678	PRJNA591966	Chicken	Australia	Campylobacter coli	9436		https://www.ncbi.nlm.nih.gov/sra/SRR10537678
Cc_18A3018F1	2018	SRR10537634	PRJNA591966	Chicken	Australia	Campylobacter coli	827	ST-828 complex	https://www.ncbi.nlm.nih.gov/sra/SRR10537634
Cc_18A3019F1	2018	SRR10537623	PRJNA591966	Chicken	Australia	Campylobacter coli	827	ST-828 complex	https://www.ncbi.nlm.nih.gov/sra/SRR10537623
Cc_18A3022F1	2018	SRR10537590	PRJNA591966	Chicken	Australia	Campylobacter coli	860	ST-828 complex	https://www.ncbi.nlm.nih.gov/sra/SRR10537590
Cc_18A3022F2	2018	SRR10537579	PRJNA591966	Chicken	Australia	Campylobacter coli	860	ST-828 complex	https://www.ncbi.nlm.nih.gov/sra/SRR10537579
Cc_18A3027F1	2018	SRR10537523	PRJNA591966	Chicken	Australia	Campylobacter coli	827	ST-828 complex	https://www.ncbi.nlm.nih.gov/sra/SRR10537523
Cc_18A3046F1	2018	SRR10538051	PRJNA591966	Chicken	Australia	Campylobacter coli	3985	ST-828 complex	https://www.ncbi.nlm.nih.gov/sra/SRR10538051
Cc_18A3055F1	2018	SRR10537951	PRJNA591966	Chicken	Australia	Campylobacter coli	10151		https://www.ncbi.nlm.nih.gov/sra/SRR10537951
Cc_18A3057F1	2018	SRR10537929	PRJNA591966	Chicken	Australia	Campylobacter coli	1243		https://www.ncbi.nlm.nih.gov/sra/SRR10537929
Cc_18A3059F1	2018	SRR10537918	PRJNA591966	Chicken	Australia	Campylobacter coli	829	ST-828 complex	https://www.ncbi.nlm.nih.gov/sra/SRR10537918
Cc_18A3060F1	2018	SRR10537907	PRJNA591966	Chicken	Australia	Campylobacter coli	832	ST-828 complex	https://www.ncbi.nlm.nih.gov/sra/SRR10537907
Cc_18A3060F2	2018	SRR10537896	PRJNA591966	Chicken	Australia	Campylobacter coli	832	ST-828 complex	https://www.ncbi.nlm.nih.gov/sra/SRR10537896
Cc_18A3062F1	2018	SRR10537874	PRJNA591966	Chicken	Australia	Campylobacter coli	1243		https://www.ncbi.nlm.nih.gov/sra/SRR10537874
Cc_18A3063F1	2018	SRR10537863	PRJNA591966	Chicken	Australia	Campylobacter coli	1243		https://www.ncbi.nlm.nih.gov/sra/SRR10537863
Cc_18A3063F2	2018	SRR10537851	PRJNA591966	Chicken	Australia	Campylobacter coli	1243		https://www.ncbi.nlm.nih.gov/sra/SRR10537851
Cc_18A3065F1	2018	SRR10537832	PRJNA591966	Chicken	Australia	Campylobacter coli	1181	ST-828 complex	https://www.ncbi.nlm.nih.gov/sra/SRR10537832
Cc_18A3065F2	2018	SRR10537831	PRJNA591966	Chicken	Australia	Campylobacter coli	1181	ST-828 complex	https://www.ncbi.nlm.nih.gov/sra/SRR10537831
Cc_18N3001F1	2018	SRR10537718	PRJNA591966	Chicken	Australia	Campylobacter coli	832	ST-828 complex	https://www.ncbi.nlm.nih.gov/sra/SRR10537718
Cc_18N3006F1	2018	SRR10537714	PRJNA591966	Chicken	Australia	Campylobacter coli	nC02		https://www.ncbi.nlm.nih.gov/sra/SRR10537714
Cc_18N3012F1	2018	SRR10537709	PRJNA591966	Chicken	Australia	Campylobacter coli	827	ST-828 complex	https://www.ncbi.nlm.nih.gov/sra/SRR10537709
Cc_18N3017F1	2018	SRR10537705	PRJNA591966	Chicken	Australia	Campylobacter coli	4175	ST-828 complex	https://www.ncbi.nlm.nih.gov/sra/SRR10537705
Cc_18N3024F1	2018	SRR10537700	PRJNA591966	Chicken	Australia	Campylobacter coli	9436		https://www.ncbi.nlm.nih.gov/sra/SRR10537700
Cc_18N3027F1	2018	SRR10537697	PRJNA591966	Chicken	Australia	Campylobacter coli	10170		https://www.ncbi.nlm.nih.gov/sra/SRR10537697
Cc_18N3028F1	2018	SRR10537696	PRJNA591966	Chicken	Australia	Campylobacter coli	9419		https://www.ncbi.nlm.nih.gov/sra/SRR10537696
Cc_18N3030F1	2018	SRR10537694	PRJNA591966	Chicken	Australia	Campylobacter coli	860	ST-828 complex	https://www.ncbi.nlm.nih.gov/sra/SRR10537694
Cc_18N3032F1	2018	SRR10537692	PRJNA591966	Chicken	Australia	Campylobacter coli	9419		https://www.ncbi.nlm.nih.gov/sra/SRR10537692
Cc_18N3045F1	2018	SRR10537686	PRJNA591966	Chicken	Australia	Campylobacter coli	4175	ST-828 complex	https://www.ncbi.nlm.nih.gov/sra/SRR10537686
Cc_18N3051F1	2018	SRR10537682	PRJNA591966	Chicken	Australia	Campylobacter coli	832	ST-828 complex	https://www.ncbi.nlm.nih.gov/sra/SRR10537682
Cc_18N3052F1	2018	SRR10537681	PRJNA591966	Chicken	Australia	Campylobacter coli	4044	ST-828 complex	https://www.ncbi.nlm.nih.gov/sra/SRR10537681
Cc_18N3055F1	2018	SRR10537677	PRJNA591966	Chicken	Australia	Campylobacter coli	1181	ST-828 complex	https://www.ncbi.nlm.nih.gov/sra/SRR10537677
Cc_18N3067F1	2018	SRR10537668	PRJNA591966	Chicken	Australia	Campylobacter coli	1181	ST-828 complex	https://www.ncbi.nlm.nih.gov/sra/SRR10537668
Cc_18N3068F1	2018	SRR10537666	PRJNA591966	Chicken	Australia	Campylobacter coli	9419		https://www.ncbi.nlm.nih.gov/sra/SRR10537666
Cc_18N3072F1	2018	SRR10537662	PRJNA591966	Chicken	Australia	Campylobacter coli	9420		https://www.ncbi.nlm.nih.gov/sra/SRR10537662
Cc_18N3074F1	2018	SRR10537660	PRJNA591966	Chicken	Australia	Campylobacter coli	860	ST-828 complex	https://www.ncbi.nlm.nih.gov/sra/SRR10537660
Cc_18N3080F1	2018	SRR10537653	PRJNA591966	Chicken	Australia	Campylobacter coli	10156		https://www.ncbi.nlm.nih.gov/sra/SRR10537653

Isolate no.	Year	Accession no.	BioProject no.	Source	Country	Species	Sequence type	Clonal complex	SRA hyperlink
Cc_18N3082F1	2018	SRR10537651	PRJNA591966	Chicken	Australia	Campylobacter coli	1243		https://www.ncbi.nlm.nih.gov/sra/SRR10537651
Cc_18N3083F1	2018	SRR10537650	PRJNA591966	Chicken	Australia	Campylobacter coli	1181	ST-828 complex	https://www.ncbi.nlm.nih.gov/sra/SRR10537650
Cc_18N3084F1	2018	SRR10537649	PRJNA591966	Chicken	Australia	Campylobacter coli	860	ST-828 complex	https://www.ncbi.nlm.nih.gov/sra/SRR10537649
Cc_18N3086F1	2018	SRR10537647	PRJNA591966	Chicken	Australia	Campylobacter coli	860	ST-828 complex	https://www.ncbi.nlm.nih.gov/sra/SRR10537647
Cc_18N3087F1	2018	SRR10537646	PRJNA591966	Chicken	Australia	Campylobacter coli	1181	ST-828 complex	https://www.ncbi.nlm.nih.gov/sra/SRR10537646
Cc_18N3088F1	2018	SRR10537644	PRJNA591966	Chicken	Australia	Campylobacter coli	1181	ST-828 complex	https://www.ncbi.nlm.nih.gov/sra/SRR10537644
Cc_18N3092F1	2018	SRR10537640	PRJNA591966	Chicken	Australia	Campylobacter coli	825	ST-828 complex	https://www.ncbi.nlm.nih.gov/sra/SRR10537640
Cc_18N3093F1	2018	SRR10537639	PRJNA591966	Chicken	Australia	Campylobacter coli	1181	ST-828 complex	https://www.ncbi.nlm.nih.gov/sra/SRR10537639
Cc_18N3123F1	2018	SRR10537637	PRJNA591966	Chicken	Australia	Campylobacter coli	860	ST-828 complex	https://www.ncbi.nlm.nih.gov/sra/SRR10537637
Cc_18N3127F1	2018	SRR10537636	PRJNA591966	Chicken	Australia	Campylobacter coli	827	ST-828 complex	https://www.ncbi.nlm.nih.gov/sra/SRR10537636
Cc_18N3128F1	2018	SRR10537635	PRJNA591966	Chicken	Australia	Campylobacter coli	860	ST-828 complex	https://www.ncbi.nlm.nih.gov/sra/SRR10537635
Cc_18N3129F1	2018	SRR10537633	PRJNA591966	Chicken	Australia	Campylobacter coli	860	ST-828 complex	https://www.ncbi.nlm.nih.gov/sra/SRR10537633
Cc_18N3133F1	2018	SRR10537632	PRJNA591966	Chicken	Australia	Campylobacter coli	860	ST-828 complex	https://www.ncbi.nlm.nih.gov/sra/SRR10537632
Cc_18N3135F1	2018	SRR10537631	PRJNA591966	Chicken	Australia	Campylobacter coli	827	ST-828 complex	https://www.ncbi.nlm.nih.gov/sra/SRR10537631
Cc_18N3147F1	2018	SRR10537629	PRJNA591966	Chicken	Australia	Campylobacter coli	9436		https://www.ncbi.nlm.nih.gov/sra/SRR10537629
Cc_18Q3014F1	2018	SRR10538033	PRJNA591966	Chicken	Australia	Campylobacter coli	832	ST-828 complex	https://www.ncbi.nlm.nih.gov/sra/SRR10538033
Cc_18Q3016F1	2018	SRR10538031	PRJNA591966	Chicken	Australia	Campylobacter coli	825	ST-828 complex	https://www.ncbi.nlm.nih.gov/sra/SRR10538031
Cc_18Q3017F1	2018	SRR10538030	PRJNA591966	Chicken	Australia	Campylobacter coli	825	ST-828 complex	https://www.ncbi.nlm.nih.gov/sra/SRR10538030
Cc_18Q3018F1	2018	SRR10538028	PRJNA591966	Chicken	Australia	Campylobacter coli	9789		https://www.ncbi.nlm.nih.gov/sra/SRR10538028
Cc_18Q3019F1	2018	SRR10538027	PRJNA591966	Chicken	Australia	Campylobacter coli	9789		https://www.ncbi.nlm.nih.gov/sra/SRR10538027
Cc_18Q3023F1	2018	SRR10538024	PRJNA591966	Chicken	Australia	Campylobacter coli	832	ST-828 complex	https://www.ncbi.nlm.nih.gov/sra/SRR10538024
Cc_18Q3024F1	2018	SRR10538023	PRJNA591966	Chicken	Australia	Campylobacter coli	4175	ST-828 complex	https://www.ncbi.nlm.nih.gov/sra/SRR10538023
Cc_18Q3026F1	2018	SRR10538021	PRJNA591966	Chicken	Australia	Campylobacter coli	3985	ST-828 complex	https://www.ncbi.nlm.nih.gov/sra/SRR10538021
Cc_18Q3028F1	2018	SRR10538019	PRJNA591966	Chicken	Australia	Campylobacter coli	3985	ST-828 complex	https://www.ncbi.nlm.nih.gov/sra/SRR10538019
Cc_18Q3029F1	2018	SRR10538017	PRJNA591966	Chicken	Australia	Campylobacter coli	9419		https://www.ncbi.nlm.nih.gov/sra/SRR10538017
Cc_18Q3033F1	2018	SRR10538013	PRJNA591966	Chicken	Australia	Campylobacter coli	3985	ST-828 complex	https://www.ncbi.nlm.nih.gov/sra/SRR10538013
Cc_18Q3036F1	2018	SRR10538010	PRJNA591966	Chicken	Australia	Campylobacter coli	3985	ST-828 complex	https://www.ncbi.nlm.nih.gov/sra/SRR10538010
Cc_18Q3040F1	2018	SRR10538005	PRJNA591966	Chicken	Australia	Campylobacter coli	827	ST-828 complex	https://www.ncbi.nlm.nih.gov/sra/SRR10538005
Cc_18V3001F1	2018	SRR10537933	PRJNA591966	Chicken	Australia	Campylobacter coli	829	ST-828 complex	https://www.ncbi.nlm.nih.gov/sra/SRR10537933
Cc_18V3002F1	2018	SRR10537932	PRJNA591966	Chicken	Australia	Campylobacter coli	825	ST-828 complex	https://www.ncbi.nlm.nih.gov/sra/SRR10537932
Cc_18V3003F1	2018	SRR10537931	PRJNA591966	Chicken	Australia	Campylobacter coli	832	ST-828 complex	https://www.ncbi.nlm.nih.gov/sra/SRR10537931
Cc_18V3005F1	2018	SRR10537928	PRJNA591966	Chicken	Australia	Campylobacter coli	1177	ST-828 complex	https://www.ncbi.nlm.nih.gov/sra/SRR10537928
Cc_18V3006F1	2018	SRR10537927	PRJNA591966	Chicken	Australia	Campylobacter coli	1181	ST-828 complex	https://www.ncbi.nlm.nih.gov/sra/SRR10537927
Cc_18V3009F1	2018	SRR10537925	PRJNA591966	Chicken	Australia	Campylobacter coli	825	ST-828 complex	https://www.ncbi.nlm.nih.gov/sra/SRR10537925
Cc_18V3010F1	2018	SRR10537924	PRJNA591966	Chicken	Australia	Campylobacter coli	827	ST-828 complex	https://www.ncbi.nlm.nih.gov/sra/SRR10537924
Cc_18V3013F1	2018	SRR10537922	PRJNA591966	Chicken	Australia	Campylobacter coli	827	ST-828 complex	https://www.ncbi.nlm.nih.gov/sra/SRR10537922
Cc_18V3015F1	2018	SRR10537920	PRJNA591966	Chicken	Australia	Campylobacter coli	825	ST-828 complex	https://www.ncbi.nlm.nih.gov/sra/SRR10537920
Cc_18V3016F1	2018	SRR10537919	PRJNA591966	Chicken	Australia	Campylobacter coli	827	ST-828 complex	https://www.ncbi.nlm.nih.gov/sra/SRR10537919
Cc_18V3016F2	2018	SRR10537917	PRJNA591966	Chicken	Australia	Campylobacter coli	827	ST-828 complex	https://www.ncbi.nlm.nih.gov/sra/SRR10537917
Cc_18V3017F1	2018	SRR10537916	PRJNA591966	Chicken	Australia	Campylobacter coli	827	ST-828 complex	https://www.ncbi.nlm.nih.gov/sra/SRR10537916
Cc_18V3018F1	2018	SRR10537915	PRJNA591966	Chicken	Australia	Campylobacter coli	827	ST-828 complex	https://www.ncbi.nlm.nih.gov/sra/SRR10537915
Cc_18V3022F1	2018	SRR10537911	PRJNA591966	Chicken	Australia	Campylobacter coli	1181	ST-828 complex	https://www.ncbi.nlm.nih.gov/sra/SRR10537911
Cc_18V3023F1	2018	SRR10537910	PRJNA591966	Chicken	Australia	Campylobacter coli	827	ST-828 complex	https://www.ncbi.nlm.nih.gov/sra/SRR10537910
Cc_18V3026F1	2018	SRR10537906	PRJNA591966	Chicken	Australia	Campylobacter coli	827	ST-828 complex	https://www.ncbi.nlm.nih.gov/sra/SRR10537906
Cc_18V3027F1	2018	SRR10537905	PRJNA591966	Chicken	Australia	Campylobacter coli	894	ST-828 complex	https://www.ncbi.nlm.nih.gov/sra/SRR10537905
Cc_18V3029F1	2018	SRR10537903	PRJNA591966	Chicken	Australia	Campylobacter coli	10162		https://www.ncbi.nlm.nih.gov/sra/SRR10537903
Cc_18V3030F1	2018	SRR10537902	PRJNA591966	Chicken	Australia	Campylobacter coli	894	ST-828 complex	https://www.ncbi.nlm.nih.gov/sra/SRR10537902
Cc_18V3031F1	2018	SRR10537901	PRJNA591966	Chicken	Australia	Campylobacter coli	1427	ST-828 complex	https://www.ncbi.nlm.nih.gov/sra/SRR10537901
Cc_18V3033F1	2018	SRR10537899	PRJNA591966	Chicken	Australia	Campylobacter coli	825	ST-828 complex	https://www.ncbi.nlm.nih.gov/sra/SRR10537899
Cc_18V3034F1	2018	SRR10537898	PRJNA591966	Chicken	Australia	Campylobacter coli	10163		https://www.ncbi.nlm.nih.gov/sra/SRR10537898
Cc_18V3035F1	2018	SRR10537897	PRJNA591966	Chicken	Australia	Campylobacter coli	1181	ST-828 complex	https://www.ncbi.nlm.nih.gov/sra/SRR10537897
Cc_18V3037F1	2018	SRR10537894	PRJNA591966	Chicken	Australia	Campylobacter coli	827	ST-828 complex	https://www.ncbi.nlm.nih.gov/sra/SRR10537894
Cc_18V3038F2	2018	SRR10537892	PRJNA591966	Chicken	Australia	Campylobacter coli	827	ST-828 complex	https://www.ncbi.nlm.nih.gov/sra/SRR10537892
Cc_18V3039F1	2018	SRR10537891	PRJNA591966	Chicken	Australia	Campylobacter coli	827	ST-828 complex	https://www.ncbi.nlm.nih.gov/sra/SRR10537891
Cc_18V3041F1	2018	SRR10537889	PRJNA591966	Chicken	Australia	Campylobacter coli	827	ST-828 complex	https://www.ncbi.nlm.nih.gov/sra/SRR10537889
Cc_18V3042F1	2018	SRR10537888	PRJNA591966	Chicken	Australia	Campylobacter coli	2179	ST-828 complex	https://www.ncbi.nlm.nih.gov/sra/SRR10537888
Cc_18V3044F1	2018	SRR10537886	PRJNA591966	Chicken	Australia	Campylobacter coli	832	ST-828 complex	https://www.ncbi.nlm.nih.gov/sra/SRR10537886
Cc_18V3045F1	2018	SRR10537884	PRJNA591966	Chicken	Australia	Campylobacter coli	1181	ST-828 complex	https://www.ncbi.nlm.nih.gov/sra/SRR10537884
Cc_18V3049F1	2018	SRR10537880	PRJNA591966	Chicken	Australia	Campylobacter coli	1181	ST-828 complex	https://www.ncbi.nlm.nih.gov/sra/SRR10537880
Cc_18V3053F1	2018	SRR10537876	PRJNA591966	Chicken	Australia	Campylobacter coli	827	ST-828 complex	https://www.ncbi.nlm.nih.gov/sra/SRR10537876
Cc_18V3056F1	2018	SRR10537872	PRJNA591966	Chicken	Australia	Campylobacter coli	827	ST-828 complex	https://www.ncbi.nlm.nih.gov/sra/SRR10537872
Cc_18V3057F1	2018	SRR10537871	PRJNA591966	Chicken	Australia	Campylobacter coli	1181	ST-828 complex	https://www.ncbi.nlm.nih.gov/sra/SRR10537871
Cc_18V3058F1	2018	SRR10537870	PRJNA591966	Chicken	Australia	Campylobacter coli	827	ST-828 complex	https://www.ncbi.nlm.nih.gov/sra/SRR10537870
Cc_18V3060F1	2018	SRR10537868	PRJNA591966	Chicken	Australia	Campylobacter coli	827	ST-828 complex	https://www.ncbi.nlm.nih.gov/sra/SRR10537868
Cc_18V3064F1	2018	SRR10537864	PRJNA591966	Chicken	Australia	Campylobacter coli	825	ST-828 complex	https://www.ncbi.nlm.nih.gov/sra/SRR10537864
Cc_18V3066F1	2018	SRR10537862	PRJNA591966	Chicken	Australia	Campylobacter coli	1181	ST-828 complex	https://www.ncbi.nlm.nih.gov/sra/SRR10537862
Cc_18V3067F1	2018	SRR10537860	PRJNA591966	Chicken	Australia	Campylobacter coli	827	ST-828 complex	https://www.ncbi.nlm.nih.gov/sra/SRR10537860
Cc_18V3068F1	2018	SRR10537859	PRJNA591966	Chicken	Australia	Campylobacter coli	829	ST-828 complex	https://www.ncbi.nlm.nih.gov/sra/SRR10537859
Cc_18V3071F2	2018	SRR10537854	PRJNA591966	Chicken	Australia	Campylobacter coli	827	ST-828 complex	https://www.ncbi.nlm.nih.gov/sra/SRR10537854
Cc_18V3072F1	2018	SRR10537853	PRJNA591966	Chicken	Australia	Campylobacter coli	1181	ST-828 complex	https://www.ncbi.nlm.nih.gov/sra/SRR10537853
Cc_18V3073F2	2018	SRR10537849	PRJNA591966	Chicken	Australia	Campylobacter coli	1181	ST-828 complex	https://www.ncbi.nlm.nih.gov/sra/SRR10537849
Cc_18V3075F1	2018	SRR10537847	PRJNA591966	Chicken	Australia	Campylobacter coli	1181	ST-828 complex	https://www.ncbi.nlm.nih.gov/sra/SRR10537847
Cc_18V3076F1	2018	SRR10537846	PRJNA591966	Chicken	Australia	Campylobacter coli	827	ST-828 complex	https://www.ncbi.nlm.nih.gov/sra/SRR10537846
Cc_18V3077F1	2018	SRR10537845	PRJNA591966	Chicken	Australia	Campylobacter coli	827	ST-828 complex	https://www.ncbi.nlm.nih.gov/sra/SRR10537845
Cc_18V3080F1	2018	SRR10537842	PRJNA591966	Chicken	Australia	Campylobacter coli	827	ST-828 complex	https://www.ncbi.nlm.nih.gov/sra/SRR10537842
Cc_18V3081F1	2018	SRR10537841	PRJNA591966	Chicken	Australia	Campylobacter coli	825	ST-828 complex	https://www.ncbi.nlm.nih.gov/sra/SRR10537841
Cc_18V3082F1	2018	SRR10537839	PRJNA591966	Chicken	Australia	Campylobacter coli	827	ST-828 complex	https://www.ncbi.nlm.nih.gov/sra/SRR10537839
Cc_18V3085F1	2018	SRR10537837	PRJNA591966	Chicken	Australia	Campylobacter coli	4175	ST-828 complex	https://www.ncbi.nlm.nih.gov/sra/SRR10537837
Cc_18V3086F1	2018	SRR10537835	PRJNA591966	Chicken	Australia	Campylobacter coli	827	ST-828 complex	https://www.ncbi.nlm.nih.gov/sra/SRR10537835
Cc_19N3014F1	2019	SRR10537626	PRJNA591966	Chicken	Australia	Campylobacter coli	9419		https://www.ncbi.nlm.nih.gov/sra/SRR10537626
Cc_19N3017F1	2019	SRR10537625	PRJNA591966	Chicken	Australia	Campylobacter coli	9436		https://www.ncbi.nlm.nih.gov/sra/SRR10537625
Cc_19N3021F1	2019	SRR10537624	PRJNA591966	Chicken	Australia	Campylobacter coli	9436		https://www.ncbi.nlm.nih.gov/sra/SRR10537624
Cc_19N3022F1	2019	SRR10537622	PRJNA591966	Chicken	Australia	Campylobacter coli	860	ST-828 complex	https://www.ncbi.nlm.nih.gov/sra/SRR10537622
Cc_19N3035F1	2019	SRR10537620	PRJNA591966	Chicken	Australia	Campylobacter coli	825	ST-828 complex	https://www.ncbi.nlm.nih.gov/sra/SRR10537620
Cc_19N3057F1	2019	SRR10537618	PRJNA591966	Chicken	Australia	Campylobacter coli	9436		https://www.ncbi.nlm.nih.gov/sra/SRR10537618
Cc_19N3082F1	2019	SRR10537617	PRJNA591966	Chicken	Australia	Campylobacter coli	1181	ST-828 complex	https://www.ncbi.nlm.nih.gov/sra/SRR10537617
Cc_19N3099F1	2019	SRR10537615	PRJNA591966	Chicken	Australia	Campylobacter coli	1181	ST-828 complex	https://www.ncbi.nlm.nih.gov/sra/SRR10537615

Isolate no.	Year	Accession no.	BioProject no.	Source	Country	Species	Sequence type	Clonal complex	SRA hyperlink
Cc_19N3106F1	2019	SRR10537611	PRJNA591966	Chicken	Australia	Campylobacter coli	1181	ST-828 complex	https://www.ncbi.nlm.nih.gov/sra/SRR10537611
Cc_19N3114F1	2019	SRR10537605	PRJNA591966	Chicken	Australia	Campylobacter coli	9420		https://www.ncbi.nlm.nih.gov/sra/SRR10537605
Cc_19N3115F1	2019	SRR10537604	PRJNA591966	Chicken	Australia	Campylobacter coli	860	ST-828 complex	https://www.ncbi.nlm.nih.gov/sra/SRR10537604
Cc_19N3119F1	2019	SRR10537602	PRJNA591966	Chicken	Australia	Campylobacter coli	827	ST-828 complex	https://www.ncbi.nlm.nih.gov/sra/SRR10537602
Cc_19N3121F1	2019	SRR10537600	PRJNA591966	Chicken	Australia	Campylobacter coli	9436		https://www.ncbi.nlm.nih.gov/sra/SRR10537600
Cc_19N3126F1	2019	SRR10537599	PRJNA591966	Chicken	Australia	Campylobacter coli	825	ST-828 complex	https://www.ncbi.nlm.nih.gov/sra/SRR10537599
Cc_19N3149F1	2019	SRR10537597	PRJNA591966	Chicken	Australia	Campylobacter coli	4175	ST-828 complex	https://www.ncbi.nlm.nih.gov/sra/SRR10537597
Cc_19N3152F1	2019	SRR10537596	PRJNA591966	Chicken	Australia	Campylobacter coli	9420		https://www.ncbi.nlm.nih.gov/sra/SRR10537596
Cc_19N3188F1	2019	SRR10537594	PRJNA591966	Chicken	Australia	Campylobacter coli	9419		https://www.ncbi.nlm.nih.gov/sra/SRR10537594
Cc_19N3195F1	2019	SRR10537589	PRJNA591966	Chicken	Australia	Campylobacter coli	832	ST-828 complex	https://www.ncbi.nlm.nih.gov/sra/SRR10537589
Cj_17N3013F1	2017	SRR10537816	PRJNA591966	Chicken	Australia	Campylobacter jejuni	528	ST-354 complex	https://www.ncbi.nlm.nih.gov/sra/SRR10537816
Cj_17N3014F1	2017	SRR10537815	PRJNA591966	Chicken	Australia	Campylobacter jejuni	449	ST-581 complex	https://www.ncbi.nlm.nih.gov/sra/SRR10537815
Cj_17N3020F1	2017	SRR10537811	PRJNA591966	Chicken	Australia	Campylobacter jejuni	10123	ST-607 complex	https://www.ncbi.nlm.nih.gov/sra/SRR10537811
Cj_17N3023F1	2017	SRR10537807	PRJNA591966	Chicken	Australia	Campylobacter jejuni	10124	ST-45 complex	https://www.ncbi.nlm.nih.gov/sra/SRR10537807
Cj_17N3027F1	2017	SRR10537804	PRJNA591966	Chicken	Australia	Campylobacter jejuni	49	ST-49 complex	https://www.ncbi.nlm.nih.gov/sra/SRR10537804
Cj_17N3055F1	2017	SRR10537786	PRJNA591966	Chicken	Australia	Campylobacter jejuni	51	ST-443 complex	https://www.ncbi.nlm.nih.gov/sra/SRR10537786
Cj_17N3057F1	2017	SRR10537785	PRJNA591966	Chicken	Australia	Campylobacter jejuni	7323		https://www.ncbi.nlm.nih.gov/sra/SRR10537785
Cj_17N3061F1	2017	SRR10537784	PRJNA591966	Chicken	Australia	Campylobacter jejuni	658	ST-658 complex	https://www.ncbi.nlm.nih.gov/sra/SRR10537784
Cj_17N3065F1	2017	SRR10537782	PRJNA591966	Chicken	Australia	Campylobacter jejuni	46	ST-206 complex	https://www.ncbi.nlm.nih.gov/sra/SRR10537782
Cj_17N3072F1	2017	SRR10537775	PRJNA591966	Chicken	Australia	Campylobacter jejuni	51	ST-443 complex	https://www.ncbi.nlm.nih.gov/sra/SRR10537775
Cj_17N3073F1	2017	SRR10537774	PRJNA591966	Chicken	Australia	Campylobacter jejuni	658	ST-658 complex	https://www.ncbi.nlm.nih.gov/sra/SRR10537774
Cj_17N3076F1	2017	SRR10537773	PRJNA591966	Chicken	Australia	Campylobacter jejuni	528	ST-354 complex	https://www.ncbi.nlm.nih.gov/sra/SRR10537773
Cj_17N3078F1	2017	SRR10537772	PRJNA591966	Chicken	Australia	Campylobacter jejuni	10126		https://www.ncbi.nlm.nih.gov/sra/SRR10537772
Cj_17N3087F1	2017	SRR10537768	PRJNA591966	Chicken	Australia	Campylobacter jejuni	449	ST-581 complex	https://www.ncbi.nlm.nih.gov/sra/SRR10537768
Cj_17N3090F1	2017	SRR10537766	PRJNA591966	Chicken	Australia	Campylobacter jejuni	61	ST-61 complex	https://www.ncbi.nlm.nih.gov/sra/SRR10537766
Cj_17N3105F1	2017	SRR10537762	PRJNA591966	Chicken	Australia	Campylobacter jejuni	48	ST-48 complex	https://www.ncbi.nlm.nih.gov/sra/SRR10537762
Cj_17N3125F1	2017	SRR10537759	PRJNA591966	Chicken	Australia	Campylobacter jejuni	5687	ST-21 complex	https://www.ncbi.nlm.nih.gov/sra/SRR10537759
Cj_17N3131F1	2017	SRR10537758	PRJNA591966	Chicken	Australia	Campylobacter jejuni	449	ST-581 complex	https://www.ncbi.nlm.nih.gov/sra/SRR10537758
Cj_17N3175F1	2017	SRR10537747	PRJNA591966	Chicken	Australia	Campylobacter jejuni	50	ST-21 complex	https://www.ncbi.nlm.nih.gov/sra/SRR10537747
Cj_17N3177F1	2017	SRR10537746	PRJNA591966	Chicken	Australia	Campylobacter jejuni	528	ST-354 complex	https://www.ncbi.nlm.nih.gov/sra/SRR10537746
Cj_17N3205F1	2017	SRR10537741	PRJNA591966	Chicken	Australia	Campylobacter jejuni	50	ST-21 complex	https://www.ncbi.nlm.nih.gov/sra/SRR10537741
Cj_17N3221F1	2017	SRR10537736	PRJNA591966	Chicken	Australia	Campylobacter jejuni	9425	ST-353 complex	https://www.ncbi.nlm.nih.gov/sra/SRR10537736
Cj_17N3242F1	2017	SRR10537731	PRJNA591966	Chicken	Australia	Campylobacter jejuni	528	ST-354 complex	https://www.ncbi.nlm.nih.gov/sra/SRR10537731
Cj_17N3250F1	2017	SRR10537725	PRJNA591966	Chicken	Australia	Campylobacter jejuni	10127	ST-257 complex	https://www.ncbi.nlm.nih.gov/sra/SRR10537725
Cj_17N3258F1	2017	SRR10537721	PRJNA591966	Chicken	Australia	Campylobacter jejuni	2083		https://www.ncbi.nlm.nih.gov/sra/SRR10537721
Cj_17Q3029F1	2017	SRR10537557	PRJNA591966	Chicken	Australia	Campylobacter jejuni	7323		https://www.ncbi.nlm.nih.gov/sra/SRR10537557
Cj_17Q3039F1	2017	SRR10537546	PRJNA591966	Chicken	Australia	Campylobacter jejuni	61	ST-61 complex	https://www.ncbi.nlm.nih.gov/sra/SRR10537546
Cj_17Q3048F1	2017	SRR10537537	PRJNA591966	Chicken	Australia	Campylobacter jejuni	449	ST-581 complex	https://www.ncbi.nlm.nih.gov/sra/SRR10537537
Cj_17Q3049F1	2017	SRR10537536	PRJNA591966	Chicken	Australia	Campylobacter jejuni	45	ST-45 complex	https://www.ncbi.nlm.nih.gov/sra/SRR10537536
Cj_17Q3054F1	2017	SRR10537531	PRJNA591966	Chicken	Australia	Campylobacter jejuni	10123	ST-607 complex	https://www.ncbi.nlm.nih.gov/sra/SRR10537531
Cj_17Q3055F1	2017	SRR10537530	PRJNA591966	Chicken	Australia	Campylobacter jejuni	449	ST-581 complex	https://www.ncbi.nlm.nih.gov/sra/SRR10537530
Cj_17Q3056F1	2017	SRR10537529	PRJNA591966	Chicken	Australia	Campylobacter jejuni	50	ST-21 complex	https://www.ncbi.nlm.nih.gov/sra/SRR10537529
Cj_17Q3057F1	2017	SRR10537528	PRJNA591966	Chicken	Australia	Campylobacter jejuni	53	ST-21 complex	https://www.ncbi.nlm.nih.gov/sra/SRR10537528
Cj_17Q3060F1	2017	SRR10537525	PRJNA591966	Chicken	Australia	Campylobacter jejuni	46	ST-206 complex	https://www.ncbi.nlm.nih.gov/sra/SRR10537525
Cj_17Q3061F1	2017	SRR10537524	PRJNA591966	Chicken	Australia	Campylobacter jejuni	449	ST-581 complex	https://www.ncbi.nlm.nih.gov/sra/SRR10537524
Cj_17Q3062F1	2017	SRR10537522	PRJNA591966	Chicken	Australia	Campylobacter jejuni	50	ST-21 complex	https://www.ncbi.nlm.nih.gov/sra/SRR10537522
Cj_17Q3064F1	2017	SRR10537520	PRJNA591966	Chicken	Australia	Campylobacter jejuni	9432	ST-353 complex	https://www.ncbi.nlm.nih.gov/sra/SRR10537520
Cj_17Q3067F1	2017	SRR10537517	PRJNA591966	Chicken	Australia	Campylobacter jejuni	699	ST-692 complex	https://www.ncbi.nlm.nih.gov/sra/SRR10537517
Cj_17Q3069F1	2017	SRR10537515	PRJNA591966	Chicken	Australia	Campylobacter jejuni	7323		https://www.ncbi.nlm.nih.gov/sra/SRR10537515
Cj_17Q3071F1	2017	SRR10537513	PRJNA591966	Chicken	Australia	Campylobacter jejuni	50	ST-21 complex	https://www.ncbi.nlm.nih.gov/sra/SRR10537513
Cj_17Q3072F1	2017	SRR10537511	PRJNA591966	Chicken	Australia	Campylobacter jejuni	45	ST-45 complex	https://www.ncbi.nlm.nih.gov/sra/SRR10537511
Cj_17Q3074F1	2017	SRR10537509	PRJNA591966	Chicken	Australia	Campylobacter jejuni	50	ST-21 complex	https://www.ncbi.nlm.nih.gov/sra/SRR10537509
Cj_17Q3080F1	2017	SRR10537503	PRJNA591966	Chicken	Australia	Campylobacter jejuni	449	ST-581 complex	https://www.ncbi.nlm.nih.gov/sra/SRR10537503
Cj_17Q3082F1	2017	SRR10537500	PRJNA591966	Chicken	Australia	Campylobacter jejuni	50	ST-21 complex	https://www.ncbi.nlm.nih.gov/sra/SRR10537500
Cj_17Q3084F1	2017	SRR10537497	PRJNA591966	Chicken	Australia	Campylobacter jejuni	991	ST-692 complex	https://www.ncbi.nlm.nih.gov/sra/SRR10537497
Cj_17Q3086F1	2017	SRR10537493	PRJNA591966	Chicken	Australia	Campylobacter jejuni	7323		https://www.ncbi.nlm.nih.gov/sra/SRR10537493
Cj_17Q3087F1	2017	SRR10537491	PRJNA591966	Chicken	Australia	Campylobacter jejuni	257	ST-257 complex	https://www.ncbi.nlm.nih.gov/sra/SRR10537491
Cj_17Q3088F1	2017	SRR10537488	PRJNA591966	Chicken	Australia	Campylobacter jejuni	4896	ST-353 complex	https://www.ncbi.nlm.nih.gov/sra/SRR10537488
Cj_17Q3088F2	2017	SRR10537487	PRJNA591966	Chicken	Australia	Campylobacter jejuni	4896	ST-353 complex	https://www.ncbi.nlm.nih.gov/sra/SRR10537487
Cj_17Q3095F1	2017	SRR10537480	PRJNA591966	Chicken	Australia	Campylobacter jejuni	4896	ST-353 complex	https://www.ncbi.nlm.nih.gov/sra/SRR10537480
Cj_17Q3097F1	2017	SRR10537477	PRJNA591966	Chicken	Australia	Campylobacter jejuni	50	ST-21 complex	https://www.ncbi.nlm.nih.gov/sra/SRR10537477
Cj_17Q3098F1	2017	SRR10537476	PRJNA591966	Chicken	Australia	Campylobacter jejuni	583	ST-45 complex	https://www.ncbi.nlm.nih.gov/sra/SRR10537476
Cj_17Q3100F1	2017	SRR10537474	PRJNA591966	Chicken	Australia	Campylobacter jejuni	538	ST-45 complex	https://www.ncbi.nlm.nih.gov/sra/SRR10537474
Cj_17Q3101F1	2017	SRR10537473	PRJNA591966	Chicken	Australia	Campylobacter jejuni	257	ST-257 complex	https://www.ncbi.nlm.nih.gov/sra/SRR10537473
Cj_17Q3102F1	2017	SRR10537472	PRJNA591966	Chicken	Australia	Campylobacter jejuni	50	ST-21 complex	https://www.ncbi.nlm.nih.gov/sra/SRR10537472
Cj_17Q3108F1	2017	SRR10537466	PRJNA591966	Chicken	Australia	Campylobacter jejuni	50	ST-21 complex	https://www.ncbi.nlm.nih.gov/sra/SRR10537466
Cj_17Q3109F1	2017	SRR10537465	PRJNA591966	Chicken	Australia	Campylobacter jejuni	528	ST-354 complex	https://www.ncbi.nlm.nih.gov/sra/SRR10537465
Cj_17Q3111F1	2017	SRR10537463	PRJNA591966	Chicken	Australia	Campylobacter jejuni	49	ST-49 complex	https://www.ncbi.nlm.nih.gov/sra/SRR10537463
Cj_17Q3113F1	2017	SRR10537461	PRJNA591966	Chicken	Australia	Campylobacter jejuni	50	ST-21 complex	https://www.ncbi.nlm.nih.gov/sra/SRR10537461
Cj_17Q3117F1	2017	SRR10538072	PRJNA591966	Chicken	Australia	Campylobacter jejuni	10129		https://www.ncbi.nlm.nih.gov/sra/SRR10538072
Cj_17Q3118F1	2017	SRR10538071	PRJNA591966	Chicken	Australia	Campylobacter jejuni	10130	ST-52 complex	https://www.ncbi.nlm.nih.gov/sra/SRR10538071
Cj_17Q3120F1	2017	SRR10538069	PRJNA591966	Chicken	Australia	Campylobacter jejuni	449	ST-581 complex	https://www.ncbi.nlm.nih.gov/sra/SRR10538069
Cj_17Q3121F1	2017	SRR10538068	PRJNA591966	Chicken	Australia	Campylobacter jejuni	257	ST-257 complex	https://www.ncbi.nlm.nih.gov/sra/SRR10538068
Cj_17Q3122F1	2017	SRR10538067	PRJNA591966	Chicken	Australia	Campylobacter jejuni	45	ST-45 complex	https://www.ncbi.nlm.nih.gov/sra/SRR10538067
Cj_17Q3123F1	2017	SRR10538066	PRJNA591966	Chicken	Australia	Campylobacter jejuni	10127	ST-257 complex	https://www.ncbi.nlm.nih.gov/sra/SRR10538066
Cj_17Q3124F1	2017	SRR10538065	PRJNA591966	Chicken	Australia	Campylobacter jejuni	9425	ST-353 complex	https://www.ncbi.nlm.nih.gov/sra/SRR10538065
Cj_17Q3125F1	2017	SRR10538064	PRJNA591966	Chicken	Australia	Campylobacter jejuni	7323		https://www.ncbi.nlm.nih.gov/sra/SRR10538064
Cj_17Q3127F1	2017	SRR10538061	PRJNA591966	Chicken	Australia	Campylobacter jejuni	49	ST-49 complex	https://www.ncbi.nlm.nih.gov/sra/SRR10538061
Cj_17Q3136F1	2017	SRR10538054	PRJNA591966	Chicken	Australia	Campylobacter jejuni	50	ST-21 complex	https://www.ncbi.nlm.nih.gov/sra/SRR10538054
Cj_17Q3139F1	2017	SRR10538050	PRJNA591966	Chicken	Australia	Campylobacter jejuni	4896	ST-353 complex	https://www.ncbi.nlm.nih.gov/sra/SRR10538050
Cj_17Q3140F1	2017	SRR10538049	PRJNA591966	Chicken	Australia	Campylobacter jejuni	7323		https://www.ncbi.nlm.nih.gov/sra/SRR10538049
Cj_17Q3141F1	2017	SRR10538048	PRJNA591966	Chicken	Australia	Campylobacter jejuni	10130	ST-52 complex	https://www.ncbi.nlm.nih.gov/sra/SRR10538048
Cj_17Q3142F1	2017	SRR10538047	PRJNA591966	Chicken	Australia	Campylobacter jejuni	6891	ST-21 complex	https://www.ncbi.nlm.nih.gov/sra/SRR10538047
Cj_17V3008F1	2017	SRR10537989	PRJNA591966	Chicken	Australia	Campylobacter jejuni	4896	ST-353 complex	https://www.ncbi.nlm.nih.gov/sra/SRR10537989
Cj_17V3009F1	2017	SRR10537988	PRJNA591966	Chicken	Australia	Campylobacter jejuni	2083		https://www.ncbi.nlm.nih.gov/sra/SRR10537988
Cj_17V3012F2	2017	SRR10537983	PRJNA591966	Chicken	Australia	Campylobacter jejuni	49	ST-49 complex	https://www.ncbi.nlm.nih.gov/sra/SRR10537983
Cj_17V3015F1	2017	SRR10537980	PRJNA591966	Chicken	Australia	Campylobacter jejuni	583	ST-45 complex	https://www.ncbi.nlm.nih.gov/sra/SRR10537980

Isolate no.	Year	Accession no.	BioProject no.	Source	Country	Species	Sequence type	Clonal complex	SRA hyperlink
Cj_17V3018F1	2017	SRR10537977	PRJNA591966	Chicken	Australia	Campylobacter jejuni	4896	ST-353 complex	https://www.ncbi.nlm.nih.gov/sra/SRR10537977
Cj_17V3022F1	2017	SRR10537972	PRJNA591966	Chicken	Australia	Campylobacter jejuni	449	ST-581 complex	https://www.ncbi.nlm.nih.gov/sra/SRR10537972
Cj_17V3028F1	2017	SRR10537969	PRJNA591966	Chicken	Australia	Campylobacter jejuni	51	ST-443 complex	https://www.ncbi.nlm.nih.gov/sra/SRR10537969
Cj_17V3029F1	2017	SRR10537968	PRJNA591966	Chicken	Australia	Campylobacter jejuni	48	ST-48 complex	https://www.ncbi.nlm.nih.gov/sra/SRR10537968
Cj_17V3030F1	2017	SRR10537967	PRJNA591966	Chicken	Australia	Campylobacter jejuni	45	ST-45 complex	https://www.ncbi.nlm.nih.gov/sra/SRR10537967
Cj_17V3031F1	2017	SRR10537966	PRJNA591966	Chicken	Australia	Campylobacter jejuni	583	ST-45 complex	https://www.ncbi.nlm.nih.gov/sra/SRR10537966
Cj_17V3032F1	2017	SRR10537964	PRJNA591966	Chicken	Australia	Campylobacter jejuni	2083		https://www.ncbi.nlm.nih.gov/sra/SRR10537964
Cj_17V3033F1	2017	SRR10537961	PRJNA591966	Chicken	Australia	Campylobacter jejuni	49	ST-49 complex	https://www.ncbi.nlm.nih.gov/sra/SRR10537961
Cj_17V3035F1	2017	SRR10537959	PRJNA591966	Chicken	Australia	Campylobacter jejuni	227	ST-206 complex	https://www.ncbi.nlm.nih.gov/sra/SRR10537959
Cj_17V3042F1	2017	SRR10537952	PRJNA591966	Chicken	Australia	Campylobacter jejuni	567	ST-22 complex	https://www.ncbi.nlm.nih.gov/sra/SRR10537952
Cj_17V3052F1	2017	SRR10537944	PRJNA591966	Chicken	Australia	Campylobacter jejuni	535	ST-460 complex	https://www.ncbi.nlm.nih.gov/sra/SRR10537944
Cj_17V3055F1	2017	SRR10537943	PRJNA591966	Chicken	Australia	Campylobacter jejuni	45	ST-45 complex	https://www.ncbi.nlm.nih.gov/sra/SRR10537943
Cj_17V3057F1	2017	SRR10537939	PRJNA591966	Chicken	Australia	Campylobacter jejuni	4896	ST-353 complex	https://www.ncbi.nlm.nih.gov/sra/SRR10537939
Cj_17V3058F2	2017	SRR10537937	PRJNA591966	Chicken	Australia	Campylobacter jejuni	9429	ST-581 complex	https://www.ncbi.nlm.nih.gov/sra/SRR10537937
Cj_17V3060F1	2017	SRR10537935	PRJNA591966	Chicken	Australia	Campylobacter jejuni	45	ST-45 complex	https://www.ncbi.nlm.nih.gov/sra/SRR10537935
Cj_18A3007F1	2018	SRR10537797	PRJNA591966	Chicken	Australia	Campylobacter jejuni	51	ST-443 complex	https://www.ncbi.nlm.nih.gov/sra/SRR10537797
Cj_18A3007F2	2018	SRR10537789	PRJNA591966	Chicken	Australia	Campylobacter jejuni	51	ST-443 complex	https://www.ncbi.nlm.nih.gov/sra/SRR10537789
Cj_18A3008F1	2018	SRR10537778	PRJNA591966	Chicken	Australia	Campylobacter jejuni	9432	ST-353 complex	https://www.ncbi.nlm.nih.gov/sra/SRR10537778
Cj_18A3008F2	2018	SRR10537767	PRJNA591966	Chicken	Australia	Campylobacter jejuni	9432	ST-353 complex	https://www.ncbi.nlm.nih.gov/sra/SRR10537767
Cj_18A3011F1	2018	SRR10537712	PRJNA591966	Chicken	Australia	Campylobacter jejuni	48	ST-48 complex	https://www.ncbi.nlm.nih.gov/sra/SRR10537712
Cj_18A3017F1	2018	SRR10537645	PRJNA591966	Chicken	Australia	Campylobacter jejuni	21	ST-21 complex	https://www.ncbi.nlm.nih.gov/sra/SRR10537645
Cj_18A3020F1	2018	SRR10537612	PRJNA591966	Chicken	Australia	Campylobacter jejuni	9432	ST-353 complex	https://www.ncbi.nlm.nih.gov/sra/SRR10537612
Cj_18A3021F1	2018	SRR10537601	PRJNA591966	Chicken	Australia	Campylobacter jejuni	50	ST-21 complex	https://www.ncbi.nlm.nih.gov/sra/SRR10537601
Cj_18A3023F1	2018	SRR10537567	PRJNA591966	Chicken	Australia	Campylobacter jejuni	21	ST-21 complex	https://www.ncbi.nlm.nih.gov/sra/SRR10537567
Cj_18A3024F1	2018	SRR10537556	PRJNA591966	Chicken	Australia	Campylobacter jejuni	21	ST-21 complex	https://www.ncbi.nlm.nih.gov/sra/SRR10537556
Cj_18A3025F1	2018	SRR10537545	PRJNA591966	Chicken	Australia	Campylobacter jejuni	50	ST-21 complex	https://www.ncbi.nlm.nih.gov/sra/SRR10537545
Cj_18A3026F1	2018	SRR10537534	PRJNA591966	Chicken	Australia	Campylobacter jejuni	9432	ST-353 complex	https://www.ncbi.nlm.nih.gov/sra/SRR10537534
Cj_18A3028F1	2018	SRR10537512	PRJNA591966	Chicken	Australia	Campylobacter jejuni	567	ST-22 complex	https://www.ncbi.nlm.nih.gov/sra/SRR10537512
Cj_18A3029F1	2018	SRR10537501	PRJNA591966	Chicken	Australia	Campylobacter jejuni	53	ST-21 complex	https://www.ncbi.nlm.nih.gov/sra/SRR10537501
Cj_18A3030F1	2018	SRR10537490	PRJNA591966	Chicken	Australia	Campylobacter jejuni	53	ST-21 complex	https://www.ncbi.nlm.nih.gov/sra/SRR10537490
Cj_18A3035F1	2018	SRR10537468	PRJNA591966	Chicken	Australia	Campylobacter jejuni	21	ST-21 complex	https://www.ncbi.nlm.nih.gov/sra/SRR10537468
Cj_18A3044F1	2018	SRR10537456	PRJNA591966	Chicken	Australia	Campylobacter jejuni	137	ST-45 complex	https://www.ncbi.nlm.nih.gov/sra/SRR10537456
Cj_18A3045F1	2018	SRR10538062	PRJNA591966	Chicken	Australia	Campylobacter jejuni	10136	ST-45 complex	https://www.ncbi.nlm.nih.gov/sra/SRR10538062
Cj_18A3054F1	2018	SRR10537962	PRJNA591966	Chicken	Australia	Campylobacter jejuni	51	ST-443 complex	https://www.ncbi.nlm.nih.gov/sra/SRR10537962
Cj_18A3056F1	2018	SRR10537940	PRJNA591966	Chicken	Australia	Campylobacter jejuni	996		https://www.ncbi.nlm.nih.gov/sra/SRR10537940
Cj_18A3061F1	2018	SRR10537885	PRJNA591966	Chicken	Australia	Campylobacter jejuni	9432	ST-353 complex	https://www.ncbi.nlm.nih.gov/sra/SRR10537885
Cj_18A3066F1	2018	SRR10537830	PRJNA591966	Chicken	Australia	Campylobacter jejuni	10134	ST-45 complex	https://www.ncbi.nlm.nih.gov/sra/SRR10537830
Cj_18A3068F1	2018	SRR10537823	PRJNA591966	Chicken	Australia	Campylobacter jejuni	21	ST-21 complex	https://www.ncbi.nlm.nih.gov/sra/SRR10537823
Cj_18A3069F1	2018	SRR10537822	PRJNA591966	Chicken	Australia	Campylobacter jejuni	4896	ST-353 complex	https://www.ncbi.nlm.nih.gov/sra/SRR10537822
Cj_18A3070F1	2018	SRR10537821	PRJNA591966	Chicken	Australia	Campylobacter jejuni	9432	ST-353 complex	https://www.ncbi.nlm.nih.gov/sra/SRR10537821
Cj_18N3004F1	2018	SRR10537716	PRJNA591966	Chicken	Australia	Campylobacter jejuni	7323		https://www.ncbi.nlm.nih.gov/sra/SRR10537716
Cj_18N3031F1	2018	SRR10537693	PRJNA591966	Chicken	Australia	Campylobacter jejuni	257	ST-257 complex	https://www.ncbi.nlm.nih.gov/sra/SRR10537693
Cj_18N3050F1	2018	SRR10537683	PRJNA591966	Chicken	Australia	Campylobacter jejuni	50	ST-21 complex	https://www.ncbi.nlm.nih.gov/sra/SRR10537683
Cj_18N3054F1	2018	SRR10537680	PRJNA591966	Chicken	Australia	Campylobacter jejuni	45	ST-45 complex	https://www.ncbi.nlm.nih.gov/sra/SRR10537680
Cj_18N3066F1	2018	SRR10537669	PRJNA591966	Chicken	Australia	Campylobacter jejuni	528	ST-354 complex	https://www.ncbi.nlm.nih.gov/sra/SRR10537669
Cj_18N3069F1	2018	SRR10537665	PRJNA591966	Chicken	Australia	Campylobacter jejuni	42	ST-42 complex	https://www.ncbi.nlm.nih.gov/sra/SRR10537665
Cj_18N3070F1	2018	SRR10537664	PRJNA591966	Chicken	Australia	Campylobacter jejuni	2083		https://www.ncbi.nlm.nih.gov/sra/SRR10537664
Cj_18N3073F1	2018	SRR10537661	PRJNA591966	Chicken	Australia	Campylobacter jejuni	696	ST-1332 complex	https://www.ncbi.nlm.nih.gov/sra/SRR10537661
Cj_18N3076F1	2018	SRR10537658	PRJNA591966	Chicken	Australia	Campylobacter jejuni	692	ST-692 complex	https://www.ncbi.nlm.nih.gov/sra/SRR10537658
Cj_18N3077F1	2018	SRR10537657	PRJNA591966	Chicken	Australia	Campylobacter jejuni	2083		https://www.ncbi.nlm.nih.gov/sra/SRR10537657
Cj_18N3078F1	2018	SRR10537655	PRJNA591966	Chicken	Australia	Campylobacter jejuni	2083		https://www.ncbi.nlm.nih.gov/sra/SRR10537655
Cj_18N3091F1	2018	SRR10537641	PRJNA591966	Chicken	Australia	Campylobacter jejuni	9432	ST-353 complex	https://www.ncbi.nlm.nih.gov/sra/SRR10537641
Cj_18N3094F1	2018	SRR10537638	PRJNA591966	Chicken	Australia	Campylobacter jejuni	51	ST-443 complex	https://www.ncbi.nlm.nih.gov/sra/SRR10537638
Cj_18N3153F1	2018	SRR10537627	PRJNA591966	Chicken	Australia	Campylobacter jejuni	46	ST-206 complex	https://www.ncbi.nlm.nih.gov/sra/SRR10537627
Cj_18Q3012F1	2018	SRR10538035	PRJNA591966	Chicken	Australia	Campylobacter jejuni	50	ST-21 complex	https://www.ncbi.nlm.nih.gov/sra/SRR10538035
Cj_18Q3013F1	2018	SRR10538034	PRJNA591966	Chicken	Australia	Campylobacter jejuni	10139	ST-45 complex	https://www.ncbi.nlm.nih.gov/sra/SRR10538034
Cj_18Q3015F1	2018	SRR10538032	PRJNA591966	Chicken	Australia	Campylobacter jejuni	583	ST-45 complex	https://www.ncbi.nlm.nih.gov/sra/SRR10538032
Cj_18Q3020F1	2018	SRR10538026	PRJNA591966	Chicken	Australia	Campylobacter jejuni	50	ST-21 complex	https://www.ncbi.nlm.nih.gov/sra/SRR10538026
Cj_18Q3025F1	2018	SRR10538022	PRJNA591966	Chicken	Australia	Campylobacter jejuni	10130	ST-52 complex	https://www.ncbi.nlm.nih.gov/sra/SRR10538022
Cj_18Q3027F1	2018	SRR10538020	PRJNA591966	Chicken	Australia	Campylobacter jejuni	567	ST-22 complex	https://www.ncbi.nlm.nih.gov/sra/SRR10538020
Cj_18Q3030F1	2018	SRR10538016	PRJNA591966	Chicken	Australia	Campylobacter jejuni	50	ST-21 complex	https://www.ncbi.nlm.nih.gov/sra/SRR10538016
Cj_18Q3031F1	2018	SRR10538015	PRJNA591966	Chicken	Australia	Campylobacter jejuni	449	ST-581 complex	https://www.ncbi.nlm.nih.gov/sra/SRR10538015
Cj_18Q3032F1	2018	SRR10538014	PRJNA591966	Chicken	Australia	Campylobacter jejuni	992		https://www.ncbi.nlm.nih.gov/sra/SRR10538014
Cj_18Q3034F1	2018	SRR10538012	PRJNA591966	Chicken	Australia	Campylobacter jejuni	50	ST-21 complex	https://www.ncbi.nlm.nih.gov/sra/SRR10538012
Cj_18Q3035F1	2018	SRR10538011	PRJNA591966	Chicken	Australia	Campylobacter jejuni	4896	ST-353 complex	https://www.ncbi.nlm.nih.gov/sra/SRR10538011
Cj_18Q3037F1	2018	SRR10538009	PRJNA591966	Chicken	Australia	Campylobacter jejuni	449	ST-581 complex	https://www.ncbi.nlm.nih.gov/sra/SRR10538009
Cj_18Q3038F1	2018	SRR10538008	PRJNA591966	Chicken	Australia	Campylobacter jejuni	449	ST-581 complex	https://www.ncbi.nlm.nih.gov/sra/SRR10538008
Cj_18Q3039F1	2018	SRR10538006	PRJNA591966	Chicken	Australia	Campylobacter jejuni	4896	ST-353 complex	https://www.ncbi.nlm.nih.gov/sra/SRR10538006
Cj_18V3004F1	2018	SRR10537930	PRJNA591966	Chicken	Australia	Campylobacter jejuni	132	ST-508 complex	https://www.ncbi.nlm.nih.gov/sra/SRR10537930
Cj_18V3008F1	2018	SRR10537926	PRJNA591966	Chicken	Australia	Campylobacter jejuni	45	ST-45 complex	https://www.ncbi.nlm.nih.gov/sra/SRR10537926
Cj_18V3011F1	2018	SRR10537923	PRJNA591966	Chicken	Australia	Campylobacter jejuni	4896	ST-353 complex	https://www.ncbi.nlm.nih.gov/sra/SRR10537923
Cj_18V3014F1	2018	SRR10537921	PRJNA591966	Chicken	Australia	Campylobacter jejuni	528	ST-354 complex	https://www.ncbi.nlm.nih.gov/sra/SRR10537921
Cj_18V3020F1	2018	SRR10537913	PRJNA591966	Chicken	Australia	Campylobacter jejuni	9429	ST-581 complex	https://www.ncbi.nlm.nih.gov/sra/SRR10537913
Cj_18V3021F1	2018	SRR10537912	PRJNA591966	Chicken	Australia	Campylobacter jejuni	4896	ST-353 complex	https://www.ncbi.nlm.nih.gov/sra/SRR10537912
Cj_18V3024F1	2018	SRR10537909	PRJNA591966	Chicken	Australia	Campylobacter jejuni	53	ST-21 complex	https://www.ncbi.nlm.nih.gov/sra/SRR10537909
Cj_18V3025F1	2018	SRR10537908	PRJNA591966	Chicken	Australia	Campylobacter jejuni	50	ST-21 complex	https://www.ncbi.nlm.nih.gov/sra/SRR10537908
Cj_18V3028F1	2018	SRR10537904	PRJNA591966	Chicken	Australia	Campylobacter jejuni	538	ST-45 complex	https://www.ncbi.nlm.nih.gov/sra/SRR10537904
Cj_18V3032F1	2018	SRR10537900	PRJNA591966	Chicken	Australia	Campylobacter jejuni	1911		https://www.ncbi.nlm.nih.gov/sra/SRR10537900
Cj_18V3036F2	2018	SRR10537895	PRJNA591966	Chicken	Australia	Campylobacter jejuni	137	ST-45 complex	https://www.ncbi.nlm.nih.gov/sra/SRR10537895
Cj_18V3038F1	2018	SRR10537893	PRJNA591966	Chicken	Australia	Campylobacter jejuni	50	ST-21 complex	https://www.ncbi.nlm.nih.gov/sra/SRR10537893
Cj_18V3040F1	2018	SRR10537890	PRJNA591966	Chicken	Australia	Campylobacter jejuni	9429	ST-581 complex	https://www.ncbi.nlm.nih.gov/sra/SRR10537890
Cj_18V3043F1	2018	SRR10537887	PRJNA591966	Chicken	Australia	Campylobacter jejuni	227	ST-206 complex	https://www.ncbi.nlm.nih.gov/sra/SRR10537887
Cj_18V3046F1	2018	SRR10537883	PRJNA591966	Chicken	Australia	Campylobacter jejuni	50	ST-21 complex	https://www.ncbi.nlm.nih.gov/sra/SRR10537883
Cj_18V3047F1	2018	SRR10537882	PRJNA591966	Chicken	Australia	Campylobacter jejuni	583	ST-45 complex	https://www.ncbi.nlm.nih.gov/sra/SRR10537882
Cj_18V3048F1	2018	SRR10537881	PRJNA591966	Chicken	Australia	Campylobacter jejuni	538	ST-45 complex	https://www.ncbi.nlm.nih.gov/sra/SRR10537881
Cj_18V3050F1	2018	SRR10537879	PRJNA591966	Chicken	Australia	Campylobacter jejuni	190	ST-21 complex	https://www.ncbi.nlm.nih.gov/sra/SRR10537879
Cj_18V3051F1	2018	SRR10537878	PRJNA591966	Chicken	Australia	Campylobacter jejuni	9429	ST-581 complex	https://www.ncbi.nlm.nih.gov/sra/SRR10537878

Isolate no.	Year	Accession no.	BioProject no.	Source	Country	Species	Sequence type	Clonal complex	SRA hyperlink
Cj_18V3052F1	2018	SRR10537877	PRJNA591966	Chicken	Australia	Campylobacter jejuni	9432	ST-353 complex	https://www.ncbi.nlm.nih.gov/sra/SRR10537877
Cj_18V3054F1	2018	SRR10537875	PRJNA591966	Chicken	Australia	Campylobacter jejuni	535	ST-460 complex	https://www.ncbi.nlm.nih.gov/sra/SRR10537875
Cj_18V3055F1	2018	SRR10537873	PRJNA591966	Chicken	Australia	Campylobacter jejuni	9429	ST-581 complex	https://www.ncbi.nlm.nih.gov/sra/SRR10537873
Cj_18V3059F1	2018	SRR10537869	PRJNA591966	Chicken	Australia	Campylobacter jejuni	9429	ST-581 complex	https://www.ncbi.nlm.nih.gov/sra/SRR10537869
Cj_18V3061F1	2018	SRR10537867	PRJNA591966	Chicken	Australia	Campylobacter jejuni	132	ST-508 complex	https://www.ncbi.nlm.nih.gov/sra/SRR10537867
Cj_18V3063F1	2018	SRR10537865	PRJNA591966	Chicken	Australia	Campylobacter jejuni	535	ST-460 complex	https://www.ncbi.nlm.nih.gov/sra/SRR10537865
Cj_18V3066F2	2018	SRR10537861	PRJNA591966	Chicken	Australia	Campylobacter jejuni	128	ST-45 complex	https://www.ncbi.nlm.nih.gov/sra/SRR10537861
Cj_18V3068F2	2018	SRR10537858	PRJNA591966	Chicken	Australia	Campylobacter jejuni	528	ST-354 complex	https://www.ncbi.nlm.nih.gov/sra/SRR10537858
Cj_18V3069F1	2018	SRR10537857	PRJNA591966	Chicken	Australia	Campylobacter jejuni	7323		https://www.ncbi.nlm.nih.gov/sra/SRR10537857
Cj_18V3070F1	2018	SRR10537856	PRJNA591966	Chicken	Australia	Campylobacter jejuni	45	ST-45 complex	https://www.ncbi.nlm.nih.gov/sra/SRR10537856
Cj_18V3071F1	2018	SRR10537855	PRJNA591966	Chicken	Australia	Campylobacter jejuni	42	ST-42 complex	https://www.ncbi.nlm.nih.gov/sra/SRR10537855
Cj_18V3073F1	2018	SRR10537850	PRJNA591966	Chicken	Australia	Campylobacter jejuni	7323		https://www.ncbi.nlm.nih.gov/sra/SRR10537850
Cj_18V3074F1	2018	SRR10537848	PRJNA591966	Chicken	Australia	Campylobacter jejuni	991	ST-692 complex	https://www.ncbi.nlm.nih.gov/sra/SRR10537848
Cj_18V3078F1	2018	SRR10537844	PRJNA591966	Chicken	Australia	Campylobacter jejuni	48	ST-48 complex	https://www.ncbi.nlm.nih.gov/sra/SRR10537844
Cj_18V3079F1	2018	SRR10537843	PRJNA591966	Chicken	Australia	Campylobacter jejuni	528	ST-354 complex	https://www.ncbi.nlm.nih.gov/sra/SRR10537843
Cj_18V3084F1	2018	SRR10537838	PRJNA591966	Chicken	Australia	Campylobacter jejuni	137	ST-45 complex	https://www.ncbi.nlm.nih.gov/sra/SRR10537838
Cj_18V3085F2	2018	SRR10537836	PRJNA591966	Chicken	Australia	Campylobacter jejuni	46	ST-206 complex	https://www.ncbi.nlm.nih.gov/sra/SRR10537836
Cj_18V4087F2	2018	SRR10537834	PRJNA591966	Chicken	Australia	Campylobacter jejuni	535	ST-460 complex	https://www.ncbi.nlm.nih.gov/sra/SRR10537834
Cj_18V5088F3	2018	SRR10537833	PRJNA591966	Chicken	Australia	Campylobacter jejuni	2083		https://www.ncbi.nlm.nih.gov/sra/SRR10537833
Cj_19N3023F1	2019	SRR10537621	PRJNA591966	Chicken	Australia	Campylobacter jejuni	528	ST-354 complex	https://www.ncbi.nlm.nih.gov/sra/SRR10537621
Cj_19N3083F1	2019	SRR10537616	PRJNA591966	Chicken	Australia	Campylobacter jejuni	46	ST-206 complex	https://www.ncbi.nlm.nih.gov/sra/SRR10537616
Cj_19N3103F1	2019	SRR10537614	PRJNA591966	Chicken	Australia	Campylobacter jejuni	50	ST-21 complex	https://www.ncbi.nlm.nih.gov/sra/SRR10537614
Cj_19N3112F1	2019	SRR10537606	PRJNA591966	Chicken	Australia	Campylobacter jejuni	9432	ST-353 complex	https://www.ncbi.nlm.nih.gov/sra/SRR10537606
Cj_19N3143F1	2019	SRR10537598	PRJNA591966	Chicken	Australia	Campylobacter jejuni	10134	ST-45 complex	https://www.ncbi.nlm.nih.gov/sra/SRR10537598
Cj_19N3185F1	2019	SRR10537595	PRJNA591966	Chicken	Australia	Campylobacter jejuni	53	ST-21 complex	https://www.ncbi.nlm.nih.gov/sra/SRR10537595
Cj_19N3191F1	2019	SRR10537592	PRJNA591966	Chicken	Australia	Campylobacter jejuni	52	ST-52 complex	https://www.ncbi.nlm.nih.gov/sra/SRR10537592
Cj_19N3192F1	2019	SRR10537591	PRJNA591966	Chicken	Australia	Campylobacter jejuni	7323		https://www.ncbi.nlm.nih.gov/sra/SRR10537591
Dc_17V1003H1	2017	SRR25376678	PRJNA592186	Human	Australia	Campylobacter coli	1181	ST-828 complex	https://www.ncbi.nlm.nih.gov/sra/SRR25376678
Dc_17V1004H1	2017	SRR25376679	PRJNA592186	Human	Australia	Campylobacter coli	10149		https://www.ncbi.nlm.nih.gov/sra/SRR25376679
Dc_17V1007H1	2017	SRR25376810	PRJNA592186	Human	Australia	Campylobacter coli	10165		https://www.ncbi.nlm.nih.gov/sra/SRR25376810
Dc_17V1008H1	2017	SRR25376811	PRJNA592186	Human	Australia	Campylobacter coli	827	ST-828 complex	https://www.ncbi.nlm.nih.gov/sra/SRR25376811
Dc_17V1012H1	2017	SRR25376817	PRJNA592186	Human	Australia	Campylobacter coli	825	ST-828 complex	https://www.ncbi.nlm.nih.gov/sra/SRR25376817
Dc_17V1015H1	2017	SRR25376819	PRJNA592186	Human	Australia	Campylobacter coli	825	ST-828 complex	https://www.ncbi.nlm.nih.gov/sra/SRR25376819
Dc_17V1025H1	2017	SRR25376828	PRJNA592186	Human	Australia	Campylobacter coli	825	ST-828 complex	https://www.ncbi.nlm.nih.gov/sra/SRR25376828
Dc_17V1027H1	2017	SRR25376830	PRJNA592186	Human	Australia	Campylobacter coli	825	ST-828 complex	https://www.ncbi.nlm.nih.gov/sra/SRR25376830
Dc_17V1040H1	2017	SRR25376835	PRJNA592186	Human	Australia	Campylobacter coli	1181	ST-828 complex	https://www.ncbi.nlm.nih.gov/sra/SRR25376835
Dc_18A1001H1	2018	SRR25377063	PRJNA592186	Human	Australia	Campylobacter coli	1181	ST-828 complex	https://www.ncbi.nlm.nih.gov/sra/SRR25377063
Dc_18A1009H1	2018	SRR25377048	PRJNA592186	Human	Australia	Campylobacter coli	860	ST-828 complex	https://www.ncbi.nlm.nih.gov/sra/SRR25377048
Dc_18A1014H1	2018	SRR25376852	PRJNA592186	Human	Australia	Campylobacter coli	860	ST-828 complex	https://www.ncbi.nlm.nih.gov/sra/SRR25376852
Dc_18N1001H1	2018	SRR25376731	PRJNA592186	Human	Australia	Campylobacter coli	827	ST-828 complex	https://www.ncbi.nlm.nih.gov/sra/SRR25376731
Dc_18N1003H1	2018	SRR25377027	PRJNA592186	Human	Australia	Campylobacter coli	10168		https://www.ncbi.nlm.nih.gov/sra/SRR25377027
Dc_18N1004H1	2018	SRR25377030	PRJNA592186	Human	Australia	Campylobacter coli	10153		https://www.ncbi.nlm.nih.gov/sra/SRR25377030
Dc_18N1020H1	2018	SRR25377046	PRJNA592186	Human	Australia	Campylobacter coli	860	ST-828 complex	https://www.ncbi.nlm.nih.gov/sra/SRR25377046
Dc_18N1031H1	2018	SRR25376622	PRJNA592186	Human	Australia	Campylobacter coli	10169	ST-828 complex	https://www.ncbi.nlm.nih.gov/sra/SRR25376622
Dc_18N1033H1	2018	SRR25376624	PRJNA592186	Human	Australia	Campylobacter coli	4175	ST-828 complex	https://www.ncbi.nlm.nih.gov/sra/SRR25376624
Dc_18N1036H1	2018	SRR25376627	PRJNA592186	Human	Australia	Campylobacter coli	1181	ST-828 complex	https://www.ncbi.nlm.nih.gov/sra/SRR25376627
Dc_18N1041H1	2018	SRR25376630	PRJNA592186	Human	Australia	Campylobacter coli	827	ST-828 complex	https://www.ncbi.nlm.nih.gov/sra/SRR25376630
Dc_18N1045H1	2018	SRR25376634	PRJNA592186	Human	Australia	Campylobacter coli	1055	ST-828 complex	https://www.ncbi.nlm.nih.gov/sra/SRR25376634
Dc_18N1053H1	2018	SRR25376636	PRJNA592186	Human	Australia	Campylobacter coli	1181	ST-828 complex	https://www.ncbi.nlm.nih.gov/sra/SRR25376636
Dc_18N1054H1	2018	SRR25376637	PRJNA592186	Human	Australia	Campylobacter coli	1181	ST-828 complex	https://www.ncbi.nlm.nih.gov/sra/SRR25376637
Dc_18N1055H1	2018	SRR25376638	PRJNA592186	Human	Australia	Campylobacter coli	827	ST-828 complex	https://www.ncbi.nlm.nih.gov/sra/SRR25376638
Dc_18N1089H1	2018	SRR25376855	PRJNA592186	Human	Australia	Campylobacter coli	6110		https://www.ncbi.nlm.nih.gov/sra/SRR25376855
Dc_18N1091H1	2018	SRR25376857	PRJNA592186	Human	Australia	Campylobacter coli	9435	ST-828 complex	https://www.ncbi.nlm.nih.gov/sra/SRR25376857
Dc_18N1094H1	2018	SRR25376860	PRJNA592186	Human	Australia	Campylobacter coli	1181	ST-828 complex	https://www.ncbi.nlm.nih.gov/sra/SRR25376860
Dc_18N1099H1	2018	SRR25376863	PRJNA592186	Human	Australia	Campylobacter coli	1181	ST-828 complex	https://www.ncbi.nlm.nih.gov/sra/SRR25376863
Dc_18N1100H1	2018	SRR25376866	PRJNA592186	Human	Australia	Campylobacter coli	9436		https://www.ncbi.nlm.nih.gov/sra/SRR25376866
Dc_18Q1006H1	2018	SRR25376980	PRJNA592186	Human	Australia	Campylobacter coli	825	ST-828 complex	https://www.ncbi.nlm.nih.gov/sra/SRR25376980
Dc_18Q1008H1	2018	SRR25376982	PRJNA592186	Human	Australia	Campylobacter coli	827	ST-828 complex	https://www.ncbi.nlm.nih.gov/sra/SRR25376982
Dc_18Q1020H1	2018	SRR25376998	PRJNA592186	Human	Australia	Campylobacter coli	827	ST-828 complex	https://www.ncbi.nlm.nih.gov/sra/SRR25376998
Dc_18Q1026H1	2018	SRR25377000	PRJNA592186	Human	Australia	Campylobacter coli	1181	ST-828 complex	https://www.ncbi.nlm.nih.gov/sra/SRR25377000
Dc_18Q1027H1	2018	SRR25377123	PRJNA592186	Human	Australia	Campylobacter coli	10172	ST-828 complex	https://www.ncbi.nlm.nih.gov/sra/SRR25377123
Dc_18Q1037H1	2018	SRR25376603	PRJNA592186	Human	Australia	Campylobacter coli	9419		https://www.ncbi.nlm.nih.gov/sra/SRR25376603
Dc_18Q1058H1	2018	SRR11318663	PRJNA592186	Human	Australia	Campylobacter coli	1181	ST-828 complex	https://www.ncbi.nlm.nih.gov/sra/SRR11318663
Dc_18Q1059H1	2018	SRR11318662	PRJNA592186	Human	Australia	Campylobacter coli	1181	ST-828 complex	https://www.ncbi.nlm.nih.gov/sra/SRR11318662
Dc_18Q1060H1	2018	SRR11318661	PRJNA592186	Human	Australia	Campylobacter coli	9436		https://www.ncbi.nlm.nih.gov/sra/SRR11318661
Dc_18Q1063H1	2018	SRR11318660	PRJNA592186	Human	Australia	Campylobacter coli	9436		https://www.ncbi.nlm.nih.gov/sra/SRR11318660
Dc_18Q1067H1	2018	SRR25376910	PRJNA592186	Human	Australia	Campylobacter coli	4175	ST-828 complex	https://www.ncbi.nlm.nih.gov/sra/SRR25376910
Dc_18Q1084H1	2018	SRR25376931	PRJNA592186	Human	Australia	Campylobacter coli	10157		https://www.ncbi.nlm.nih.gov/sra/SRR25376931
Dc_18Q1089H1	2018	SRR25377003	PRJNA592186	Human	Australia	Campylobacter coli	10173		https://www.ncbi.nlm.nih.gov/sra/SRR25377003
Dc_18Q1125H1	2018	SRR25376944	PRJNA592186	Human	Australia	Campylobacter coli	1181	ST-828 complex	https://www.ncbi.nlm.nih.gov/sra/SRR25376944
Dc_18Q1128H1	2018	SRR25376945	PRJNA592186	Human	Australia	Campylobacter coli	1181	ST-828 complex	https://www.ncbi.nlm.nih.gov/sra/SRR25376945
Dc_18Q1130H1	2018	SRR25376947	PRJNA592186	Human	Australia	Campylobacter coli	1181	ST-828 complex	https://www.ncbi.nlm.nih.gov/sra/SRR25376947
Dc_18Q1133H1	2018	SRR25376951	PRJNA592186	Human	Australia	Campylobacter coli	9420		https://www.ncbi.nlm.nih.gov/sra/SRR25376951
Dc_18Q1136H1	2018	SRR25376954	PRJNA592186	Human	Australia	Campylobacter coli	825	ST-828 complex	https://www.ncbi.nlm.nih.gov/sra/SRR25376954
Dc_18Q1139H1	2018	SRR25376956	PRJNA592186	Human	Australia	Campylobacter coli	1181	ST-828 complex	https://www.ncbi.nlm.nih.gov/sra/SRR25376956
Dc_18Q1143H1	2018	SRR25376959	PRJNA592186	Human	Australia	Campylobacter coli	2179	ST-828 complex	https://www.ncbi.nlm.nih.gov/sra/SRR25376959
Dc_18Q1147H1	2018	SRR25376966	PRJNA592186	Human	Australia	Campylobacter coli	825	ST-828 complex	https://www.ncbi.nlm.nih.gov/sra/SRR25376966
Dc_18Q1149H1	2018	SRR25377022	PRJNA592186	Human	Australia	Campylobacter coli	827	ST-828 complex	https://www.ncbi.nlm.nih.gov/sra/SRR25377022
Dc_18Q1153H1	2018	SRR25376631	PRJNA592186	Human	Australia	Campylobacter coli	825	ST-828 complex	https://www.ncbi.nlm.nih.gov/sra/SRR25376631
Dc_18Q1166H1	2018	SRR25376748	PRJNA592186	Human	Australia	Campylobacter coli	9436		https://www.ncbi.nlm.nih.gov/sra/SRR25376748
Dc_18Q1169H1	2018	SRR25376752	PRJNA592186	Human	Australia	Campylobacter coli	1181	ST-828 complex	https://www.ncbi.nlm.nih.gov/sra/SRR25376752
Dc_18Q1171H1	2018	SRR25376754	PRJNA592186	Human	Australia	Campylobacter coli	1181	ST-828 complex	https://www.ncbi.nlm.nih.gov/sra/SRR25376754
Dc_18Q1172H1	2018	SRR25376755	PRJNA592186	Human	Australia	Campylobacter coli	9436		https://www.ncbi.nlm.nih.gov/sra/SRR25376755
Dc_18Q1173H1	2018	SRR25376756	PRJNA592186	Human	Australia	Campylobacter coli	1181	ST-828 complex	https://www.ncbi.nlm.nih.gov/sra/SRR25376756
Dc_18Q1178H1	2018	SRR25376761	PRJNA592186	Human	Australia	Campylobacter coli	9420		https://www.ncbi.nlm.nih.gov/sra/SRR25376761
Dc_18Q1179H1	2018	SRR25376763	PRJNA592186	Human	Australia	Campylobacter coli	10158	ST-828 complex	https://www.ncbi.nlm.nih.gov/sra/SRR25376763
Dc_18Q1184H1	2018	SRR25376768	PRJNA592186	Human	Australia	Campylobacter coli	831	ST-828 complex	https://www.ncbi.nlm.nih.gov/sra/SRR25376768

Isolate no.	Year	Accession no.	BioProject no.	Source	Country	Species	Sequence type	Clonal complex	SRA hyperlink
Dc_18Q1185H1	2018	SRR25376770	PRJNA592186	Human	Australia	Campylobacter coli	831	ST-828 complex	https://www.ncbi.nlm.nih.gov/sra/SRR25376770
Dc_18Q1191H1	2018	SRR25376777	PRJNA592186	Human	Australia	Campylobacter coli	825	ST-828 complex	https://www.ncbi.nlm.nih.gov/sra/SRR25376777
Dc_18Q1199H1	2018	SRR25376781	PRJNA592186	Human	Australia	Campylobacter coli	9436		https://www.ncbi.nlm.nih.gov/sra/SRR25376781
Dc_18Q1218H1	2018	SRR25377098	PRJNA592186	Human	Australia	Campylobacter coli	9436		https://www.ncbi.nlm.nih.gov/sra/SRR25377098
Dc_18Q1221H1	2018	SRR25376983	PRJNA592186	Human	Australia	Campylobacter coli	825	ST-828 complex	https://www.ncbi.nlm.nih.gov/sra/SRR25376983
Dc_18Q1226H1	2018	SRR25376751	PRJNA592186	Human	Australia	Campylobacter coli	825	ST-828 complex	https://www.ncbi.nlm.nih.gov/sra/SRR25376751
Dc_18Q1240H1	2018	SRR25376842	PRJNA592186	Human	Australia	Campylobacter coli	1181	ST-828 complex	https://www.ncbi.nlm.nih.gov/sra/SRR25376842
Dc_18Q1265H1	2018	SRR25376794	PRJNA592186	Human	Australia	Campylobacter coli	825	ST-828 complex	https://www.ncbi.nlm.nih.gov/sra/SRR25376794
Dc_18Q1297H1	2018	SRR25376670	PRJNA592186	Human	Australia	Campylobacter coli	1181	ST-828 complex	https://www.ncbi.nlm.nih.gov/sra/SRR25376670
Dc_18Q1298H1	2018	SRR25376671	PRJNA592186	Human	Australia	Campylobacter coli	860	ST-828 complex	https://www.ncbi.nlm.nih.gov/sra/SRR25376671
Dc_18V1005H1	2018	SRR25376839	PRJNA592186	Human	Australia	Campylobacter coli	872	ST-828 complex	https://www.ncbi.nlm.nih.gov/sra/SRR25376839
Dc_18V1015H1	2018	SRR25376683	PRJNA592186	Human	Australia	Campylobacter coli	825	ST-828 complex	https://www.ncbi.nlm.nih.gov/sra/SRR25376683
Dc_18V1020H1	2018	SRR25376686	PRJNA592186	Human	Australia	Campylobacter coli	827	ST-828 complex	https://www.ncbi.nlm.nih.gov/sra/SRR25376686
Dc_18V1028H1	2018	SRR25377081	PRJNA592186	Human	Australia	Campylobacter coli	829	ST-828 complex	https://www.ncbi.nlm.nih.gov/sra/SRR25377081
Dc_18V1036H1	2018	SRR25377085	PRJNA592186	Human	Australia	Campylobacter coli	825	ST-828 complex	https://www.ncbi.nlm.nih.gov/sra/SRR25377085
Dc_18V1052H1	2018	SRR25376726	PRJNA592186	Human	Australia	Campylobacter coli	nC01		https://www.ncbi.nlm.nih.gov/sra/SRR25376726
Dc_18V1065H1	2018	SRR25376738	PRJNA592186	Human	Australia	Campylobacter coli	10161	ST-828 complex	https://www.ncbi.nlm.nih.gov/sra/SRR25376738
Dc_18V1067H1	2018	SRR25376740	PRJNA592186	Human	Australia	Campylobacter coli	1181	ST-828 complex	https://www.ncbi.nlm.nih.gov/sra/SRR25376740
Dc_18V1069H1	2018	SRR25376742	PRJNA592186	Human	Australia	Campylobacter coli	nC10		https://www.ncbi.nlm.nih.gov/sra/SRR25376742
Dc_18V1072H1	2018	SRR25376744	PRJNA592186	Human	Australia	Campylobacter coli	9419		https://www.ncbi.nlm.nih.gov/sra/SRR25376744
Dc_18V1075H1	2018	SRR25376845	PRJNA592186	Human	Australia	Campylobacter coli	1181	ST-828 complex	https://www.ncbi.nlm.nih.gov/sra/SRR25376845
Dc_19N1007H1	2019	SRR25376693	PRJNA592186	Human	Australia	Campylobacter coli	829	ST-828 complex	https://www.ncbi.nlm.nih.gov/sra/SRR25376693
Dc_19N1025H1	2019	SRR25376696	PRJNA592186	Human	Australia	Campylobacter coli	825	ST-828 complex	https://www.ncbi.nlm.nih.gov/sra/SRR25376696
Dc_19N1065H1	2019	SRR25377058	PRJNA592186	Human	Australia	Campylobacter coli	860	ST-828 complex	https://www.ncbi.nlm.nih.gov/sra/SRR25377058
Dc_19N1073H1	2019	SRR25377061	PRJNA592186	Human	Australia	Campylobacter coli	860	ST-828 complex	https://www.ncbi.nlm.nih.gov/sra/SRR25377061
Dc_19N1077H1	2019	SRR25377089	PRJNA592186	Human	Australia	Campylobacter coli	1181	ST-828 complex	https://www.ncbi.nlm.nih.gov/sra/SRR25377089
Dc_19N1078H1	2019	SRR25377091	PRJNA592186	Human	Australia	Campylobacter coli	9419		https://www.ncbi.nlm.nih.gov/sra/SRR25377091
Dc_19N1079H1	2019	SRR25377092	PRJNA592186	Human	Australia	Campylobacter coli	9436		https://www.ncbi.nlm.nih.gov/sra/SRR25377092
Dc_19N1080H1	2019	SRR11318658	PRJNA592186	Human	Australia	Campylobacter coli	827	ST-828 complex	https://www.ncbi.nlm.nih.gov/sra/SRR11318658
Dc_19N1088H1	2019	SRR25377100	PRJNA592186	Human	Australia	Campylobacter coli	1181	ST-828 complex	https://www.ncbi.nlm.nih.gov/sra/SRR25377100
Dc_19N1091H1	2019	SRR25377103	PRJNA592186	Human	Australia	Campylobacter coli	825	ST-828 complex	https://www.ncbi.nlm.nih.gov/sra/SRR25377103
Dc_19N1093H1	2019	SRR25377111	PRJNA592186	Human	Australia	Campylobacter coli	827	ST-828 complex	https://www.ncbi.nlm.nih.gov/sra/SRR25377111
Dc_19N1107H1	2019	SRR25377115	PRJNA592186	Human	Australia	Campylobacter coli	827	ST-828 complex	https://www.ncbi.nlm.nih.gov/sra/SRR25377115
Dc_19N1191H1	2019	SRR11318657	PRJNA592186	Human	Australia	Campylobacter coli	829	ST-828 complex	https://www.ncbi.nlm.nih.gov/sra/SRR11318657
Dj_17V1001H1	2017	SRR25376675	PRJNA592186	Human	Australia	Campylobacter jejuni	48	ST-48 complex	https://www.ncbi.nlm.nih.gov/sra/SRR25376675
Dj_17V1002H1	2017	SRR25376677	PRJNA592186	Human	Australia	Campylobacter jejuni	10133	ST-21 complex	https://www.ncbi.nlm.nih.gov/sra/SRR25376677
Dj_17V1005H1	2017	SRR25376680	PRJNA592186	Human	Australia	Campylobacter jejuni	128	ST-45 complex	https://www.ncbi.nlm.nih.gov/sra/SRR25376680
Dj_17V1006H1	2017	SRR25376681	PRJNA592186	Human	Australia	Campylobacter jejuni	49	ST-49 complex	https://www.ncbi.nlm.nih.gov/sra/SRR25376681
Dj_17V1009H1	2017	SRR25376813	PRJNA592186	Human	Australia	Campylobacter jejuni	658	ST-658 complex	https://www.ncbi.nlm.nih.gov/sra/SRR25376813
Dj_17V1010H1	2017	SRR25376814	PRJNA592186	Human	Australia	Campylobacter jejuni	10134	ST-45 complex	https://www.ncbi.nlm.nih.gov/sra/SRR25376814
Dj_17V1011H1	2017	SRR25376816	PRJNA592186	Human	Australia	Campylobacter jejuni	658	ST-658 complex	https://www.ncbi.nlm.nih.gov/sra/SRR25376816
Dj_17V1013H1	2017	SRR25376818	PRJNA592186	Human	Australia	Campylobacter jejuni	1972		https://www.ncbi.nlm.nih.gov/sra/SRR25376818
Dj_17V1016H1	2017	SRR25376820	PRJNA592186	Human	Australia	Campylobacter jejuni	48	ST-48 complex	https://www.ncbi.nlm.nih.gov/sra/SRR25376820
Dj_17V1019H1	2017	SRR25376821	PRJNA592186	Human	Australia	Campylobacter jejuni	583	ST-45 complex	https://www.ncbi.nlm.nih.gov/sra/SRR25376821
Dj_17V1022H1	2017	SRR25376822	PRJNA592186	Human	Australia	Campylobacter jejuni	61	ST-61 complex	https://www.ncbi.nlm.nih.gov/sra/SRR25376822
Dj_17V1023H1	2017	SRR25376823	PRJNA592186	Human	Australia	Campylobacter jejuni	583	ST-45 complex	https://www.ncbi.nlm.nih.gov/sra/SRR25376823
Dj_17V1024H1	2017	SRR25376825	PRJNA592186	Human	Australia	Campylobacter jejuni	5	ST-353 complex	https://www.ncbi.nlm.nih.gov/sra/SRR25376825
Dj_17V1026H1	2017	SRR25376829	PRJNA592186	Human	Australia	Campylobacter jejuni	61	ST-61 complex	https://www.ncbi.nlm.nih.gov/sra/SRR25376829
Dj_17V1028H1	2017	SRR25376831	PRJNA592186	Human	Australia	Campylobacter jejuni	10133	ST-21 complex	https://www.ncbi.nlm.nih.gov/sra/SRR25376831
Dj_17V1030H1	2017	SRR25376832	PRJNA592186	Human	Australia	Campylobacter jejuni	50	ST-21 complex	https://www.ncbi.nlm.nih.gov/sra/SRR25376832
Dj_17V1031H1	2017	SRR25376833	PRJNA592186	Human	Australia	Campylobacter jejuni	51	ST-443 complex	https://www.ncbi.nlm.nih.gov/sra/SRR25376833
Dj_17V1036H1	2017	SRR25376834	PRJNA592186	Human	Australia	Campylobacter jejuni	528	ST-354 complex	https://www.ncbi.nlm.nih.gov/sra/SRR25376834
Dj_18A1002H1	2018	SRR25377064	PRJNA592186	Human	Australia	Campylobacter jejuni	2343	ST-48 complex	https://www.ncbi.nlm.nih.gov/sra/SRR25377064
Dj_18A1004H1	2018	SRR25376990	PRJNA592186	Human	Australia	Campylobacter jejuni	190	ST-21 complex	https://www.ncbi.nlm.nih.gov/sra/SRR25376990
Dj_18A1005H1	2018	SRR25376960	PRJNA592186	Human	Australia	Campylobacter jejuni	7323		https://www.ncbi.nlm.nih.gov/sra/SRR25376960
Dj_18A1006H1	2018	SRR25376883	PRJNA592186	Human	Australia	Campylobacter jejuni	51	ST-443 complex	https://www.ncbi.nlm.nih.gov/sra/SRR25376883
Dj_18A1007H1	2018	SRR25376718	PRJNA592186	Human	Australia	Campylobacter jejuni	48	ST-48 complex	https://www.ncbi.nlm.nih.gov/sra/SRR25376718
Dj_18A1008H1	2018	SRR25377036	PRJNA592186	Human	Australia	Campylobacter jejuni	567	ST-22 complex	https://www.ncbi.nlm.nih.gov/sra/SRR25377036
Dj_18A1010H1	2018	SRR25376625	PRJNA592186	Human	Australia	Campylobacter jejuni	50	ST-21 complex	https://www.ncbi.nlm.nih.gov/sra/SRR25376625
Dj_18A1011H1	2018	SRR25376641	PRJNA592186	Human	Australia	Campylobacter jejuni	982	ST-21 complex	https://www.ncbi.nlm.nih.gov/sra/SRR25376641
Dj_18A1013H1	2018	SRR25377065	PRJNA592186	Human	Australia	Campylobacter jejuni	48	ST-48 complex	https://www.ncbi.nlm.nih.gov/sra/SRR25377065
Dj_18A1015H1	2018	SRR25376865	PRJNA592186	Human	Australia	Campylobacter jejuni	567	ST-22 complex	https://www.ncbi.nlm.nih.gov/sra/SRR25376865
Dj_18A1016H1	2018	SRR25376691	PRJNA592186	Human	Australia	Campylobacter jejuni	696	ST-1332 complex	https://www.ncbi.nlm.nih.gov/sra/SRR25376691
Dj_18A1017H1	2018	SRR25376703	PRJNA592186	Human	Australia	Campylobacter jejuni	50	ST-21 complex	https://www.ncbi.nlm.nih.gov/sra/SRR25376703
Dj_18A1018H1	2018	SRR25377055	PRJNA592186	Human	Australia	Campylobacter jejuni	21	ST-21 complex	https://www.ncbi.nlm.nih.gov/sra/SRR25377055
Dj_18A1024H1	2018	SRR25377102	PRJNA592186	Human	Australia	Campylobacter jejuni	48	ST-48 complex	https://www.ncbi.nlm.nih.gov/sra/SRR25377102
Dj_18A1025H1	2018	SRR25377114	PRJNA592186	Human	Australia	Campylobacter jejuni	50	ST-21 complex	https://www.ncbi.nlm.nih.gov/sra/SRR25377114
Dj_18A1026H1	2018	SRR25376978	PRJNA592186	Human	Australia	Campylobacter jejuni	21	ST-21 complex	https://www.ncbi.nlm.nih.gov/sra/SRR25376978
Dj_18A1029H1	2018	SRR25376991	PRJNA592186	Human	Australia	Campylobacter jejuni	50	ST-21 complex	https://www.ncbi.nlm.nih.gov/sra/SRR25376991
Dj_18A1031H1	2018	SRR25377120	PRJNA592186	Human	Australia	Campylobacter jejuni	21	ST-21 complex	https://www.ncbi.nlm.nih.gov/sra/SRR25377120
Dj_18A1032H1	2018	SRR25376602	PRJNA592186	Human	Australia	Campylobacter jejuni	42	ST-42 complex	https://www.ncbi.nlm.nih.gov/sra/SRR25376602
Dj_18A1033H1	2018	SRR25376894	PRJNA592186	Human	Australia	Campylobacter jejuni	2083		https://www.ncbi.nlm.nih.gov/sra/SRR25376894
Dj_18A1034H1	2018	SRR25376906	PRJNA592186	Human	Australia	Campylobacter jejuni	1728	ST-21 complex	https://www.ncbi.nlm.nih.gov/sra/SRR25376906
Dj_18A1035H1	2018	SRR25376918	PRJNA592186	Human	Australia	Campylobacter jejuni	567	ST-22 complex	https://www.ncbi.nlm.nih.gov/sra/SRR25376918
Dj_18A1036H1	2018	SRR25376930	PRJNA592186	Human	Australia	Campylobacter jejuni	50	ST-21 complex	https://www.ncbi.nlm.nih.gov/sra/SRR25376930
Dj_18N1002H1	2018	SRR25376743	PRJNA592186	Human	Australia	Campylobacter jejuni	10139	ST-45 complex	https://www.ncbi.nlm.nih.gov/sra/SRR25376743
Dj_18N1005H1	2018	SRR25377025	PRJNA592186	Human	Australia	Campylobacter jejuni	583	ST-45 complex	https://www.ncbi.nlm.nih.gov/sra/SRR25377025
Dj_18N1006H1	2018	SRR25377031	PRJNA592186	Human	Australia	Campylobacter jejuni	567	ST-22 complex	https://www.ncbi.nlm.nih.gov/sra/SRR25377031
Dj_18N1007H1	2018	SRR25377032	PRJNA592186	Human	Australia	Campylobacter jejuni	508	ST-508 complex	https://www.ncbi.nlm.nih.gov/sra/SRR25377032
Dj_18N1008H1	2018	SRR25377034	PRJNA592186	Human	Australia	Campylobacter jejuni	42	ST-42 complex	https://www.ncbi.nlm.nih.gov/sra/SRR25377034
Dj_18N1009H1	2018	SRR25377035	PRJNA592186	Human	Australia	Campylobacter jejuni	583	ST-45 complex	https://www.ncbi.nlm.nih.gov/sra/SRR25377035
Dj_18N1010H1	2018	SRR25377037	PRJNA592186	Human	Australia	Campylobacter jejuni	52	ST-52 complex	https://www.ncbi.nlm.nih.gov/sra/SRR25377037
Dj_18N1011H1	2018	SRR25377038	PRJNA592186	Human	Australia	Campylobacter jejuni	61	ST-61 complex	https://www.ncbi.nlm.nih.gov/sra/SRR25377038
Dj_18N1012H1	2018	SRR25377039	PRJNA592186	Human	Australia	Campylobacter jejuni	190	ST-21 complex	https://www.ncbi.nlm.nih.gov/sra/SRR25377039
Dj_18N1014H1	2018	SRR25377040	PRJNA592186	Human	Australia	Campylobacter jejuni	61	ST-61 complex	https://www.ncbi.nlm.nih.gov/sra/SRR25377040
Dj_18N1015H1	2018	SRR25377041	PRJNA592186	Human	Australia	Campylobacter jejuni	7323		https://www.ncbi.nlm.nih.gov/sra/SRR25377041
Dj_18N1017H1	2018	SRR25377042	PRJNA592186	Human	Australia	Campylobacter jejuni	190	ST-21 complex	https://www.ncbi.nlm.nih.gov/sra/SRR25377042

Isolate no.	Year	Accession no.	BioProject no.	Source	Country	Species	Sequence type	Clonal complex	SRA hyperlink
Dj_18N1018H1	2018	SRR25377043	PRJNA592186	Human	Australia	Campylobacter jejuni	50	ST-21 complex	https://www.ncbi.nlm.nih.gov/sra/SRR25377043
Dj_18N1019H1	2018	SRR25377045	PRJNA592186	Human	Australia	Campylobacter jejuni	227	ST-206 complex	https://www.ncbi.nlm.nih.gov/sra/SRR25377045
Dj_18N1021H1	2018	SRR25377047	PRJNA592186	Human	Australia	Campylobacter jejuni	50	ST-21 complex	https://www.ncbi.nlm.nih.gov/sra/SRR25377047
Dj_18N1022H1	2018	SRR25377049	PRJNA592186	Human	Australia	Campylobacter jejuni	2343	ST-48 complex	https://www.ncbi.nlm.nih.gov/sra/SRR25377049
Dj_18N1023H1	2018	SRR25377050	PRJNA592186	Human	Australia	Campylobacter jejuni	48	ST-48 complex	https://www.ncbi.nlm.nih.gov/sra/SRR25377050
Dj_18N1025H1	2018	SRR25377051	PRJNA592186	Human	Australia	Campylobacter jejuni	658	ST-658 complex	https://www.ncbi.nlm.nih.gov/sra/SRR25377051
Dj_18N1026H1	2018	SRR25377052	PRJNA592186	Human	Australia	Campylobacter jejuni	583	ST-45 complex	https://www.ncbi.nlm.nih.gov/sra/SRR25377052
Dj_18N1027H1	2018	SRR25377053	PRJNA592186	Human	Australia	Campylobacter jejuni	49	ST-49 complex	https://www.ncbi.nlm.nih.gov/sra/SRR25377053
Dj_18N1029H1	2018	SRR25376618	PRJNA592186	Human	Australia	Campylobacter jejuni	520	ST-21 complex	https://www.ncbi.nlm.nih.gov/sra/SRR25376618
Dj_18N1030H1	2018	SRR25376621	PRJNA592186	Human	Australia	Campylobacter jejuni	50	ST-21 complex	https://www.ncbi.nlm.nih.gov/sra/SRR25376621
Dj_18N1032H1	2018	SRR25376623	PRJNA592186	Human	Australia	Campylobacter jejuni	583	ST-45 complex	https://www.ncbi.nlm.nih.gov/sra/SRR25376623
Dj_18N1034H1	2018	SRR25376626	PRJNA592186	Human	Australia	Campylobacter jejuni	50	ST-21 complex	https://www.ncbi.nlm.nih.gov/sra/SRR25376626
Dj_18N1037H1	2018	SRR25376628	PRJNA592186	Human	Australia	Campylobacter jejuni	658	ST-658 complex	https://www.ncbi.nlm.nih.gov/sra/SRR25376628
Dj_18N1039H1	2018	SRR25376629	PRJNA592186	Human	Australia	Campylobacter jejuni	5687	ST-21 complex	https://www.ncbi.nlm.nih.gov/sra/SRR25376629
Dj_18N1042H1	2018	SRR25376632	PRJNA592186	Human	Australia	Campylobacter jejuni	42	ST-42 complex	https://www.ncbi.nlm.nih.gov/sra/SRR25376632
Dj_18N1043H1	2018	SRR25376633	PRJNA592186	Human	Australia	Campylobacter jejuni	50	ST-21 complex	https://www.ncbi.nlm.nih.gov/sra/SRR25376633
Dj_18N1049H1	2018	SRR25376635	PRJNA592186	Human	Australia	Campylobacter jejuni	2343	ST-48 complex	https://www.ncbi.nlm.nih.gov/sra/SRR25376635
Dj_18N1050H1	2018	SRR25376640	PRJNA592186	Human	Australia	Campylobacter jejuni	53	ST-21 complex	https://www.ncbi.nlm.nih.gov/sra/SRR25376640
Dj_18N1051H1	2018	SRR25376643	PRJNA592186	Human	Australia	Campylobacter jejuni	9432	ST-353 complex	https://www.ncbi.nlm.nih.gov/sra/SRR25376643
Dj_18N1057H1	2018	SRR25376639	PRJNA592186	Human	Australia	Campylobacter jejuni	567	ST-22 complex	https://www.ncbi.nlm.nih.gov/sra/SRR25376639
Dj_18N1062H1	2018	SRR25376644	PRJNA592186	Human	Australia	Campylobacter jejuni	50	ST-21 complex	https://www.ncbi.nlm.nih.gov/sra/SRR25376644
Dj_18N1064H1	2018	SRR25376968	PRJNA592186	Human	Australia	Campylobacter jejuni	50	ST-21 complex	https://www.ncbi.nlm.nih.gov/sra/SRR25376968
Dj_18N1065H1	2018	SRR25376969	PRJNA592186	Human	Australia	Campylobacter jejuni	567	ST-22 complex	https://www.ncbi.nlm.nih.gov/sra/SRR25376969
Dj_18N1066H1	2018	SRR25377023	PRJNA592186	Human	Australia	Campylobacter jejuni	137	ST-45 complex	https://www.ncbi.nlm.nih.gov/sra/SRR25377023
Dj_18N1070H1	2018	SRR25377024	PRJNA592186	Human	Australia	Campylobacter jejuni	190	ST-21 complex	https://www.ncbi.nlm.nih.gov/sra/SRR25377024
Dj_18N1071H1	2018	SRR25377066	PRJNA592186	Human	Australia	Campylobacter jejuni	528	ST-354 complex	https://www.ncbi.nlm.nih.gov/sra/SRR25377066
Dj_18N1072H1	2018	SRR25377067	PRJNA592186	Human	Australia	Campylobacter jejuni	8436	ST-42 complex	https://www.ncbi.nlm.nih.gov/sra/SRR25377067
Dj_18N1076H1	2018	SRR25377068	PRJNA592186	Human	Australia	Campylobacter jejuni	8436	ST-42 complex	https://www.ncbi.nlm.nih.gov/sra/SRR25377068
Dj_18N1078H1	2018	SRR25377069	PRJNA592186	Human	Australia	Campylobacter jejuni	61	ST-61 complex	https://www.ncbi.nlm.nih.gov/sra/SRR25377069
Dj_18N1079H1	2018	SRR25377070	PRJNA592186	Human	Australia	Campylobacter jejuni	49	ST-49 complex	https://www.ncbi.nlm.nih.gov/sra/SRR25377070
Dj_18N1080H1	2018	SRR25377074	PRJNA592186	Human	Australia	Campylobacter jejuni	161	ST-52 complex	https://www.ncbi.nlm.nih.gov/sra/SRR25377074
Dj_18N1081H1	2018	SRR25377075	PRJNA592186	Human	Australia	Campylobacter jejuni	42	ST-42 complex	https://www.ncbi.nlm.nih.gov/sra/SRR25377075
Dj_18N1082H1	2018	SRR25377076	PRJNA592186	Human	Australia	Campylobacter jejuni	21	ST-21 complex	https://www.ncbi.nlm.nih.gov/sra/SRR25377076
Dj_18N1086H1	2018	SRR25377077	PRJNA592186	Human	Australia	Campylobacter jejuni	46	ST-206 complex	https://www.ncbi.nlm.nih.gov/sra/SRR25377077
Dj_18N1087H1	2018	SRR25376851	PRJNA592186	Human	Australia	Campylobacter jejuni	46	ST-206 complex	https://www.ncbi.nlm.nih.gov/sra/SRR25376851
Dj_18N1088H1	2018	SRR25376854	PRJNA592186	Human	Australia	Campylobacter jejuni	528	ST-354 complex	https://www.ncbi.nlm.nih.gov/sra/SRR25376854
Dj_18N1089H2	2018	SRR25376856	PRJNA592186	Human	Australia	Campylobacter jejuni	528	ST-354 complex	https://www.ncbi.nlm.nih.gov/sra/SRR25376856
Dj_18N1092H1	2018	SRR25376858	PRJNA592186	Human	Australia	Campylobacter jejuni	53	ST-21 complex	https://www.ncbi.nlm.nih.gov/sra/SRR25376858
Dj_18N1093H1	2018	SRR25376859	PRJNA592186	Human	Australia	Campylobacter jejuni	567	ST-22 complex	https://www.ncbi.nlm.nih.gov/sra/SRR25376859
Dj_18N1097H1	2018	SRR25376861	PRJNA592186	Human	Australia	Campylobacter jejuni	567	ST-22 complex	https://www.ncbi.nlm.nih.gov/sra/SRR25376861
Dj_18N1098H1	2018	SRR25376862	PRJNA592186	Human	Australia	Campylobacter jejuni	50	ST-21 complex	https://www.ncbi.nlm.nih.gov/sra/SRR25376862
Dj_18N1101H1	2018	SRR25376867	PRJNA592186	Human	Australia	Campylobacter jejuni	21	ST-21 complex	https://www.ncbi.nlm.nih.gov/sra/SRR25376867
Dj_18N1102H1	2018	SRR25376868	PRJNA592186	Human	Australia	Campylobacter jejuni	508	ST-508 complex	https://www.ncbi.nlm.nih.gov/sra/SRR25376868
Dj_18N1103H1	2018	SRR25376869	PRJNA592186	Human	Australia	Campylobacter jejuni	534		https://www.ncbi.nlm.nih.gov/sra/SRR25376869
Dj_18N1107H1	2018	SRR25376870	PRJNA592186	Human	Australia	Campylobacter jejuni	42	ST-42 complex	https://www.ncbi.nlm.nih.gov/sra/SRR25376870
Dj_18N1119H1	2018	SRR25376871	PRJNA592186	Human	Australia	Campylobacter jejuni	4080		https://www.ncbi.nlm.nih.gov/sra/SRR25376871
Dj_18N1120H1	2018	SRR25376872	PRJNA592186	Human	Australia	Campylobacter jejuni	2083		https://www.ncbi.nlm.nih.gov/sra/SRR25376872
Dj_18N1121H1	2018	SRR25376687	PRJNA592186	Human	Australia	Campylobacter jejuni	2343	ST-48 complex	https://www.ncbi.nlm.nih.gov/sra/SRR25376687
Dj_18Q1001H1	2018	SRR25376975	PRJNA592186	Human	Australia	Campylobacter jejuni	49	ST-49 complex	https://www.ncbi.nlm.nih.gov/sra/SRR25376975
Dj_18Q1002H1	2018	SRR25376976	PRJNA592186	Human	Australia	Campylobacter jejuni	567	ST-22 complex	https://www.ncbi.nlm.nih.gov/sra/SRR25376976
Dj_18Q1003H1	2018	SRR11318650	PRJNA592186	Human	Australia	Campylobacter jejuni	137	ST-45 complex	https://www.ncbi.nlm.nih.gov/sra/SRR11318650
Dj_18Q1004H1	2018	SRR25376977	PRJNA592186	Human	Australia	Campylobacter jejuni	658	ST-658 complex	https://www.ncbi.nlm.nih.gov/sra/SRR25376977
Dj_18Q1005H1	2018	SRR25376979	PRJNA592186	Human	Australia	Campylobacter jejuni	50	ST-21 complex	https://www.ncbi.nlm.nih.gov/sra/SRR25376979
Dj_18Q1007H1	2018	SRR25376981	PRJNA592186	Human	Australia	Campylobacter jejuni	525	ST-607 complex	https://www.ncbi.nlm.nih.gov/sra/SRR25376981
Dj_18Q1009H1	2018	SRR25376984	PRJNA592186	Human	Australia	Campylobacter jejuni	61	ST-61 complex	https://www.ncbi.nlm.nih.gov/sra/SRR25376984
Dj_18Q1010H1	2018	SRR25376985	PRJNA592186	Human	Australia	Campylobacter jejuni	46	ST-206 complex	https://www.ncbi.nlm.nih.gov/sra/SRR25376985
Dj_18Q1011H1	2018	SRR25376986	PRJNA592186	Human	Australia	Campylobacter jejuni	21	ST-21 complex	https://www.ncbi.nlm.nih.gov/sra/SRR25376986
Dj_18Q1012H1	2018	SRR25376987	PRJNA592186	Human	Australia	Campylobacter jejuni	658	ST-658 complex	https://www.ncbi.nlm.nih.gov/sra/SRR25376987
Dj_18Q1013H1	2018	SRR25376988	PRJNA592186	Human	Australia	Campylobacter jejuni	49	ST-49 complex	https://www.ncbi.nlm.nih.gov/sra/SRR25376988
Dj_18Q1014H1	2018	SRR25376989	PRJNA592186	Human	Australia	Campylobacter jejuni	535	ST-460 complex	https://www.ncbi.nlm.nih.gov/sra/SRR25376989
Dj_18Q1015H1	2018	SRR25376992	PRJNA592186	Human	Australia	Campylobacter jejuni	567	ST-22 complex	https://www.ncbi.nlm.nih.gov/sra/SRR25376992
Dj_18Q1016H1	2018	SRR25376993	PRJNA592186	Human	Australia	Campylobacter jejuni	50	ST-21 complex	https://www.ncbi.nlm.nih.gov/sra/SRR25376993
Dj_18Q1017H1	2018	SRR25376995	PRJNA592186	Human	Australia	Campylobacter jejuni	658	ST-658 complex	https://www.ncbi.nlm.nih.gov/sra/SRR25376995
Dj_18Q1018H1	2018	SRR25376996	PRJNA592186	Human	Australia	Campylobacter jejuni	2083		https://www.ncbi.nlm.nih.gov/sra/SRR25376996
Dj_18Q1019H1	2018	SRR25376997	PRJNA592186	Human	Australia	Campylobacter jejuni	538	ST-45 complex	https://www.ncbi.nlm.nih.gov/sra/SRR25376997
Dj_18Q1021H1	2018	SRR25376999	PRJNA592186	Human	Australia	Campylobacter jejuni	10130	ST-52 complex	https://www.ncbi.nlm.nih.gov/sra/SRR25376999
Dj_18Q1022H1	2018	SRR25377001	PRJNA592186	Human	Australia	Campylobacter jejuni	658	ST-658 complex	https://www.ncbi.nlm.nih.gov/sra/SRR25377001
Dj_18Q1023H1	2018	SRR25377118	PRJNA592186	Human	Australia	Campylobacter jejuni	21	ST-21 complex	https://www.ncbi.nlm.nih.gov/sra/SRR25377118
Dj_18Q1024H1	2018	SRR25377119	PRJNA592186	Human	Australia	Campylobacter jejuni	257	ST-257 complex	https://www.ncbi.nlm.nih.gov/sra/SRR25377119
Dj_18Q1025H1	2018	SRR25377122	PRJNA592186	Human	Australia	Campylobacter jejuni	567	ST-22 complex	https://www.ncbi.nlm.nih.gov/sra/SRR25377122
Dj_18Q1028H1	2018	SRR25377124	PRJNA592186	Human	Australia	Campylobacter jejuni	10130	ST-52 complex	https://www.ncbi.nlm.nih.gov/sra/SRR25377124
Dj_18Q1029H1	2018	SRR11318669	PRJNA592186	Human	Australia	Campylobacter jejuni	50	ST-21 complex	https://www.ncbi.nlm.nih.gov/sra/SRR11318669
Dj_18Q1030H1	2018	SRR25377125	PRJNA592186	Human	Australia	Campylobacter jejuni	9432	ST-353 complex	https://www.ncbi.nlm.nih.gov/sra/SRR25377125
Dj_18Q1031H1	2018	SRR25377126	PRJNA592186	Human	Australia	Campylobacter jejuni	45	ST-45 complex	https://www.ncbi.nlm.nih.gov/sra/SRR25377126
Dj_18Q1032H1	2018	SRR25377127	PRJNA592186	Human	Australia	Campylobacter jejuni	61	ST-61 complex	https://www.ncbi.nlm.nih.gov/sra/SRR25377127
Dj_18Q1033H1	2018	SRR25377128	PRJNA592186	Human	Australia	Campylobacter jejuni	7323		https://www.ncbi.nlm.nih.gov/sra/SRR25377128
Dj_18Q1034H1	2018	SRR11318668	PRJNA592186	Human	Australia	Campylobacter jejuni	50	ST-21 complex	https://www.ncbi.nlm.nih.gov/sra/SRR11318668
Dj_18Q1035H1	2018	SRR25377129	PRJNA592186	Human	Australia	Campylobacter jejuni	50	ST-21 complex	https://www.ncbi.nlm.nih.gov/sra/SRR25377129
Dj_18Q1036H1	2018	SRR25377130	PRJNA592186	Human	Australia	Campylobacter jejuni	528	ST-354 complex	https://www.ncbi.nlm.nih.gov/sra/SRR25377130
Dj_18Q1038H1	2018	SRR25376604	PRJNA592186	Human	Australia	Campylobacter jejuni	53	ST-21 complex	https://www.ncbi.nlm.nih.gov/sra/SRR25376604
Dj_18Q1039H1	2018	SRR25376605	PRJNA592186	Human	Australia	Campylobacter jejuni	50	ST-21 complex	https://www.ncbi.nlm.nih.gov/sra/SRR25376605
Dj_18Q1040H1	2018	SRR25376606	PRJNA592186	Human	Australia	Campylobacter jejuni	46	ST-206 complex	https://www.ncbi.nlm.nih.gov/sra/SRR25376606
Dj_18Q1041H1	2018	SRR25376887	PRJNA592186	Human	Australia	Campylobacter jejuni	46	ST-206 complex	https://www.ncbi.nlm.nih.gov/sra/SRR25376887
Dj_18Q1042H1	2018	SRR25376888	PRJNA592186	Human	Australia	Campylobacter jejuni	50	ST-21 complex	https://www.ncbi.nlm.nih.gov/sra/SRR25376888
Dj_18Q1043H1	2018	SRR25376889	PRJNA592186	Human	Australia	Campylobacter jejuni	50	ST-21 complex	https://www.ncbi.nlm.nih.gov/sra/SRR25376889
Dj_18Q1044H1	2018	SRR25376890	PRJNA592186	Human	Australia	Campylobacter jejuni	61	ST-61 complex	https://www.ncbi.nlm.nih.gov/sra/SRR25376890
Dj_18Q1045H1	2018	SRR25376892	PRJNA592186	Human	Australia	Campylobacter jejuni	525	ST-607 complex	https://www.ncbi.nlm.nih.gov/sra/SRR25376892

Isolate no.	Year	Accession no.	BioProject no.	Source	Country	Species	Sequence type	Clonal complex	SRA hyperlink
Dj_18Q1047H1	2018	SRR25376895	PRJNA592186	Human	Australia	Campylobacter jejuni	52	ST-52 complex	https://www.ncbi.nlm.nih.gov/sra/SRR25376895
Dj_18Q1048H1	2018	SRR25376896	PRJNA592186	Human	Australia	Campylobacter jejuni	567	ST-22 complex	https://www.ncbi.nlm.nih.gov/sra/SRR25376896
Dj_18Q1049H1	2018	SRR25376897	PRJNA592186	Human	Australia	Campylobacter jejuni	50	ST-21 complex	https://www.ncbi.nlm.nih.gov/sra/SRR25376897
Dj_18Q1054H1	2018	SRR11318667	PRJNA592186	Human	Australia	Campylobacter jejuni	61	ST-61 complex	https://www.ncbi.nlm.nih.gov/sra/SRR11318667
Dj_18Q1055H1	2018	SRR25376903	PRJNA592186	Human	Australia	Campylobacter jejuni	53	ST-21 complex	https://www.ncbi.nlm.nih.gov/sra/SRR25376903
Dj_18Q1056H1	2018	SRR11318666	PRJNA592186	Human	Australia	Campylobacter jejuni	528	ST-354 complex	https://www.ncbi.nlm.nih.gov/sra/SRR11318666
Dj_18Q1057H1	2018	SRR11318665	PRJNA592186	Human	Australia	Campylobacter jejuni	508	ST-508 complex	https://www.ncbi.nlm.nih.gov/sra/SRR11318665
Dj_18Q1068H1	2018	SRR25376911	PRJNA592186	Human	Australia	Campylobacter jejuni	658	ST-658 complex	https://www.ncbi.nlm.nih.gov/sra/SRR25376911
Dj_18Q1069H1	2018	SRR25376912	PRJNA592186	Human	Australia	Campylobacter jejuni	50	ST-21 complex	https://www.ncbi.nlm.nih.gov/sra/SRR25376912
Dj_18Q1070H1	2018	SRR25376914	PRJNA592186	Human	Australia	Campylobacter jejuni	257	ST-257 complex	https://www.ncbi.nlm.nih.gov/sra/SRR25376914
Dj_18Q1071H1	2018	SRR25376915	PRJNA592186	Human	Australia	Campylobacter jejuni	525	ST-607 complex	https://www.ncbi.nlm.nih.gov/sra/SRR25376915
Dj_18Q1072H1	2018	SRR25376916	PRJNA592186	Human	Australia	Campylobacter jejuni	525	ST-607 complex	https://www.ncbi.nlm.nih.gov/sra/SRR25376916
Dj_18Q1073H1	2018	SRR25376917	PRJNA592186	Human	Australia	Campylobacter jejuni	4896	ST-353 complex	https://www.ncbi.nlm.nih.gov/sra/SRR25376917
Dj_18Q1074H1	2018	SRR25376919	PRJNA592186	Human	Australia	Campylobacter jejuni	61	ST-61 complex	https://www.ncbi.nlm.nih.gov/sra/SRR25376919
Dj_18Q1075H1	2018	SRR25376920	PRJNA592186	Human	Australia	Campylobacter jejuni	2398		https://www.ncbi.nlm.nih.gov/sra/SRR25376920
Dj_18Q1076H1	2018	SRR25376921	PRJNA592186	Human	Australia	Campylobacter jejuni	4896	ST-353 complex	https://www.ncbi.nlm.nih.gov/sra/SRR25376921
Dj_18Q1077H1	2018	SRR25376922	PRJNA592186	Human	Australia	Campylobacter jejuni	50	ST-21 complex	https://www.ncbi.nlm.nih.gov/sra/SRR25376922
Dj_18Q1078H1	2018	SRR25376923	PRJNA592186	Human	Australia	Campylobacter jejuni	525	ST-607 complex	https://www.ncbi.nlm.nih.gov/sra/SRR25376923
Dj_18Q1079H1	2018	SRR25376925	PRJNA592186	Human	Australia	Campylobacter jejuni	257	ST-257 complex	https://www.ncbi.nlm.nih.gov/sra/SRR25376925
Dj_18Q1080H1	2018	SRR25376926	PRJNA592186	Human	Australia	Campylobacter jejuni	48	ST-48 complex	https://www.ncbi.nlm.nih.gov/sra/SRR25376926
Dj_18Q1081H1	2018	SRR25376927	PRJNA592186	Human	Australia	Campylobacter jejuni	525	ST-607 complex	https://www.ncbi.nlm.nih.gov/sra/SRR25376927
Dj_18Q1082H1	2018	SRR25376928	PRJNA592186	Human	Australia	Campylobacter jejuni	46	ST-206 complex	https://www.ncbi.nlm.nih.gov/sra/SRR25376928
Dj_18Q1083H1	2018	SRR25376929	PRJNA592186	Human	Australia	Campylobacter jejuni	525	ST-607 complex	https://www.ncbi.nlm.nih.gov/sra/SRR25376929
Dj_18Q1085H1	2018	SRR25376932	PRJNA592186	Human	Australia	Campylobacter jejuni	50	ST-21 complex	https://www.ncbi.nlm.nih.gov/sra/SRR25376932
Dj_18Q1086H1	2018	SRR25376933	PRJNA592186	Human	Australia	Campylobacter jejuni	2398		https://www.ncbi.nlm.nih.gov/sra/SRR25376933
Dj_18Q1087H1	2018	SRR25376934	PRJNA592186	Human	Australia	Campylobacter jejuni	535	ST-460 complex	https://www.ncbi.nlm.nih.gov/sra/SRR25376934
Dj_18Q1088H1	2018	SRR25377002	PRJNA592186	Human	Australia	Campylobacter jejuni	520	ST-21 complex	https://www.ncbi.nlm.nih.gov/sra/SRR25377002
Dj_18Q1090H1	2018	SRR25377004	PRJNA592186	Human	Australia	Campylobacter jejuni	10127	ST-257 complex	https://www.ncbi.nlm.nih.gov/sra/SRR25377004
Dj_18Q1094H1	2018	SRR25377006	PRJNA592186	Human	Australia	Campylobacter jejuni	53	ST-21 complex	https://www.ncbi.nlm.nih.gov/sra/SRR25377006
Dj_18Q1099H1	2018	SRR25377010	PRJNA592186	Human	Australia	Campylobacter jejuni	53	ST-21 complex	https://www.ncbi.nlm.nih.gov/sra/SRR25377010
Dj_18Q1100H1	2018	SRR25377011	PRJNA592186	Human	Australia	Campylobacter jejuni	50	ST-21 complex	https://www.ncbi.nlm.nih.gov/sra/SRR25377011
Dj_18Q1103H1	2018	SRR25377014	PRJNA592186	Human	Australia	Campylobacter jejuni	61	ST-61 complex	https://www.ncbi.nlm.nih.gov/sra/SRR25377014
Dj_18Q1106H1	2018	SRR25377015	PRJNA592186	Human	Australia	Campylobacter jejuni	61	ST-61 complex	https://www.ncbi.nlm.nih.gov/sra/SRR25377015
Dj_18Q1108H1	2018	SRR25377016	PRJNA592186	Human	Australia	Campylobacter jejuni	9432	ST-353 complex	https://www.ncbi.nlm.nih.gov/sra/SRR25377016
Dj_18Q1115H1	2018	SRR25377019	PRJNA592186	Human	Australia	Campylobacter jejuni	50	ST-21 complex	https://www.ncbi.nlm.nih.gov/sra/SRR25377019
Dj_18Q1117H1	2018	SRR25376938	PRJNA592186	Human	Australia	Campylobacter jejuni	42	ST-42 complex	https://www.ncbi.nlm.nih.gov/sra/SRR25376938
Dj_18Q1118H1	2018	SRR25376940	PRJNA592186	Human	Australia	Campylobacter jejuni	9432	ST-353 complex	https://www.ncbi.nlm.nih.gov/sra/SRR25376940
Dj_18Q1119H1	2018	SRR25376941	PRJNA592186	Human	Australia	Campylobacter jejuni	10143	ST-353 complex	https://www.ncbi.nlm.nih.gov/sra/SRR25376941
Dj_18Q1120H1	2018	SRR25376942	PRJNA592186	Human	Australia	Campylobacter jejuni	50	ST-21 complex	https://www.ncbi.nlm.nih.gov/sra/SRR25376942
Dj_18Q1121H1	2018	SRR25376943	PRJNA592186	Human	Australia	Campylobacter jejuni	50	ST-21 complex	https://www.ncbi.nlm.nih.gov/sra/SRR25376943
Dj_18Q1129H1	2018	SRR25376946	PRJNA592186	Human	Australia	Campylobacter jejuni	50	ST-21 complex	https://www.ncbi.nlm.nih.gov/sra/SRR25376946
Dj_18Q1132H1	2018	SRR25376949	PRJNA592186	Human	Australia	Campylobacter jejuni	528	ST-354 complex	https://www.ncbi.nlm.nih.gov/sra/SRR25376949
Dj_18Q1134H1	2018	SRR25376952	PRJNA592186	Human	Australia	Campylobacter jejuni	61	ST-61 complex	https://www.ncbi.nlm.nih.gov/sra/SRR25376952
Dj_18Q1135H1	2018	SRR25376953	PRJNA592186	Human	Australia	Campylobacter jejuni	42	ST-42 complex	https://www.ncbi.nlm.nih.gov/sra/SRR25376953
Dj_18Q1137H1	2018	SRR25376955	PRJNA592186	Human	Australia	Campylobacter jejuni	9432	ST-353 complex	https://www.ncbi.nlm.nih.gov/sra/SRR25376955
Dj_18Q1141H1	2018	SRR25376957	PRJNA592186	Human	Australia	Campylobacter jejuni	2343	ST-48 complex	https://www.ncbi.nlm.nih.gov/sra/SRR25376957
Dj_18Q1142H1	2018	SRR25376958	PRJNA592186	Human	Australia	Campylobacter jejuni	48	ST-48 complex	https://www.ncbi.nlm.nih.gov/sra/SRR25376958
Dj_18Q1144H1	2018	SRR25376963	PRJNA592186	Human	Australia	Campylobacter jejuni	50	ST-21 complex	https://www.ncbi.nlm.nih.gov/sra/SRR25376963
Dj_18Q1145H1	2018	SRR25376964	PRJNA592186	Human	Australia	Campylobacter jejuni	50	ST-21 complex	https://www.ncbi.nlm.nih.gov/sra/SRR25376964
Dj_18Q1146H1	2018	SRR25376965	PRJNA592186	Human	Australia	Campylobacter jejuni	2083		https://www.ncbi.nlm.nih.gov/sra/SRR25376965
Dj_18Q1148H1	2018	SRR25376967	PRJNA592186	Human	Australia	Campylobacter jejuni	508	ST-508 complex	https://www.ncbi.nlm.nih.gov/sra/SRR25376967
Dj_18Q1150H1	2018	SRR25377033	PRJNA592186	Human	Australia	Campylobacter jejuni	538	ST-45 complex	https://www.ncbi.nlm.nih.gov/sra/SRR25377033
Dj_18Q1151H1	2018	SRR25377044	PRJNA592186	Human	Australia	Campylobacter jejuni	48	ST-48 complex	https://www.ncbi.nlm.nih.gov/sra/SRR25377044
Dj_18Q1152H1	2018	SRR25376619	PRJNA592186	Human	Australia	Campylobacter jejuni	10146		https://www.ncbi.nlm.nih.gov/sra/SRR25376619
Dj_18Q1154H1	2018	SRR25377131	PRJNA592186	Human	Australia	Campylobacter jejuni	449	ST-581 complex	https://www.ncbi.nlm.nih.gov/sra/SRR25377131
Dj_18Q1156H1	2018	SRR25376614	PRJNA592186	Human	Australia	Campylobacter jejuni	48	ST-48 complex	https://www.ncbi.nlm.nih.gov/sra/SRR25376614
Dj_18Q1157H1	2018	SRR25376607	PRJNA592186	Human	Australia	Campylobacter jejuni	42	ST-42 complex	https://www.ncbi.nlm.nih.gov/sra/SRR25376607
Dj_18Q1158H1	2018	SRR25376608	PRJNA592186	Human	Australia	Campylobacter jejuni	52	ST-52 complex	https://www.ncbi.nlm.nih.gov/sra/SRR25376608
Dj_18Q1159H1	2018	SRR25376609	PRJNA592186	Human	Australia	Campylobacter jejuni	567	ST-22 complex	https://www.ncbi.nlm.nih.gov/sra/SRR25376609
Dj_18Q1160H1	2018	SRR25376610	PRJNA592186	Human	Australia	Campylobacter jejuni	567	ST-22 complex	https://www.ncbi.nlm.nih.gov/sra/SRR25376610
Dj_18Q1161H1	2018	SRR25376611	PRJNA592186	Human	Australia	Campylobacter jejuni	50	ST-21 complex	https://www.ncbi.nlm.nih.gov/sra/SRR25376611
Dj_18Q1162H1	2018	SRR25376615	PRJNA592186	Human	Australia	Campylobacter jejuni	2083		https://www.ncbi.nlm.nih.gov/sra/SRR25376615
Dj_18Q1163H1	2018	SRR25376616	PRJNA592186	Human	Australia	Campylobacter jejuni	10132		https://www.ncbi.nlm.nih.gov/sra/SRR25376616
Dj_18Q1164H1	2018	SRR25376746	PRJNA592186	Human	Australia	Campylobacter jejuni	48	ST-48 complex	https://www.ncbi.nlm.nih.gov/sra/SRR25376746
Dj_18Q1165H1	2018	SRR25376747	PRJNA592186	Human	Australia	Campylobacter jejuni	2398		https://www.ncbi.nlm.nih.gov/sra/SRR25376747
Dj_18Q1167H1	2018	SRR25376749	PRJNA592186	Human	Australia	Campylobacter jejuni	508	ST-508 complex	https://www.ncbi.nlm.nih.gov/sra/SRR25376749
Dj_18Q1168H1	2018	SRR25376750	PRJNA592186	Human	Australia	Campylobacter jejuni	45	ST-45 complex	https://www.ncbi.nlm.nih.gov/sra/SRR25376750
Dj_18Q1170H1	2018	SRR25376753	PRJNA592186	Human	Australia	Campylobacter jejuni	696	ST-1332 complex	https://www.ncbi.nlm.nih.gov/sra/SRR25376753
Dj_18Q1175H1	2018	SRR25376758	PRJNA592186	Human	Australia	Campylobacter jejuni	52	ST-52 complex	https://www.ncbi.nlm.nih.gov/sra/SRR25376758
Dj_18Q1176H1	2018	SRR25376759	PRJNA592186	Human	Australia	Campylobacter jejuni	525	ST-607 complex	https://www.ncbi.nlm.nih.gov/sra/SRR25376759
Dj_18Q1177H1	2018	SRR25376760	PRJNA592186	Human	Australia	Campylobacter jejuni	137	ST-45 complex	https://www.ncbi.nlm.nih.gov/sra/SRR25376760
Dj_18Q1180H1	2018	SRR25376764	PRJNA592186	Human	Australia	Campylobacter jejuni	567	ST-22 complex	https://www.ncbi.nlm.nih.gov/sra/SRR25376764
Dj_18Q1181H1	2018	SRR25376765	PRJNA592186	Human	Australia	Campylobacter jejuni	9425	ST-353 complex	https://www.ncbi.nlm.nih.gov/sra/SRR25376765
Dj_18Q1182H1	2018	SRR25376766	PRJNA592186	Human	Australia	Campylobacter jejuni	9592	ST-177 complex	https://www.ncbi.nlm.nih.gov/sra/SRR25376766
Dj_18Q1183H1	2018	SRR25376767	PRJNA592186	Human	Australia	Campylobacter jejuni	42	ST-42 complex	https://www.ncbi.nlm.nih.gov/sra/SRR25376767
Dj_18Q1186H1	2018	SRR25376771	PRJNA592186	Human	Australia	Campylobacter jejuni	38	ST-48 complex	https://www.ncbi.nlm.nih.gov/sra/SRR25376771
Dj_18Q1187H1	2018	SRR25376772	PRJNA592186	Human	Australia	Campylobacter jejuni	2398		https://www.ncbi.nlm.nih.gov/sra/SRR25376772
Dj_18Q1188H1	2018	SRR25376774	PRJNA592186	Human	Australia	Campylobacter jejuni	48	ST-48 complex	https://www.ncbi.nlm.nih.gov/sra/SRR25376774
Dj_18Q1189H1	2018	SRR25376775	PRJNA592186	Human	Australia	Campylobacter jejuni	508	ST-508 complex	https://www.ncbi.nlm.nih.gov/sra/SRR25376775
Dj_18Q1190H1	2018	SRR25376776	PRJNA592186	Human	Australia	Campylobacter jejuni	48	ST-48 complex	https://www.ncbi.nlm.nih.gov/sra/SRR25376776
Dj_18Q1192H1	2018	SRR25376874	PRJNA592186	Human	Australia	Campylobacter jejuni	658	ST-658 complex	https://www.ncbi.nlm.nih.gov/sra/SRR25376874
Dj_18Q1193H1	2018	SRR25376875	PRJNA592186	Human	Australia	Campylobacter jejuni	10130	ST-52 complex	https://www.ncbi.nlm.nih.gov/sra/SRR25376875
Dj_18Q1194H1	2018	SRR25376876	PRJNA592186	Human	Australia	Campylobacter jejuni	137	ST-45 complex	https://www.ncbi.nlm.nih.gov/sra/SRR25376876
Dj_18Q1195H1	2018	SRR25376878	PRJNA592186	Human	Australia	Campylobacter jejuni	50	ST-21 complex	https://www.ncbi.nlm.nih.gov/sra/SRR25376878
Dj_18Q1196H1	2018	SRR25376879	PRJNA592186	Human	Australia	Campylobacter jejuni	48	ST-48 complex	https://www.ncbi.nlm.nih.gov/sra/SRR25376879
Dj_18Q1197H1	2018	SRR25376885	PRJNA592186	Human	Australia	Campylobacter jejuni	7323		https://www.ncbi.nlm.nih.gov/sra/SRR25376885
Dj_18Q1198H1	2018	SRR25376886	PRJNA592186	Human	Australia	Campylobacter jejuni	567	ST-22 complex	https://www.ncbi.nlm.nih.gov/sra/SRR25376886

Isolate no.	Year	Accession no.	BioProject no.	Source	Country	Species	Sequence type	Clonal complex	SRA hyperlink
Dj_18Q1200H1	2018	SRR25376792	PRJNA592186	Human	Australia	Campylobacter jejuni	658	ST-658 complex	https://www.ncbi.nlm.nih.gov/sra/SRR25376792
Dj_18Q1201H1	2018	SRR25376803	PRJNA592186	Human	Australia	Campylobacter jejuni	51	ST-443 complex	https://www.ncbi.nlm.nih.gov/sra/SRR25376803
Dj_18Q1202H1	2018	SRR25376654	PRJNA592186	Human	Australia	Campylobacter jejuni	21	ST-21 complex	https://www.ncbi.nlm.nih.gov/sra/SRR25376654
Dj_18Q1203H1	2018	SRR25376665	PRJNA592186	Human	Australia	Campylobacter jejuni	2947	ST-257 complex	https://www.ncbi.nlm.nih.gov/sra/SRR25376665
Dj_18Q1204H1	2018	SRR25376676	PRJNA592186	Human	Australia	Campylobacter jejuni	21	ST-21 complex	https://www.ncbi.nlm.nih.gov/sra/SRR25376676
Dj_18Q1205H1	2018	SRR25376826	PRJNA592186	Human	Australia	Campylobacter jejuni	42	ST-42 complex	https://www.ncbi.nlm.nih.gov/sra/SRR25376826
Dj_18Q1206H1	2018	SRR25376838	PRJNA592186	Human	Australia	Campylobacter jejuni	50	ST-21 complex	https://www.ncbi.nlm.nih.gov/sra/SRR25376838
Dj_18Q1207H1	2018	SRR25376689	PRJNA592186	Human	Australia	Campylobacter jejuni	508	ST-508 complex	https://www.ncbi.nlm.nih.gov/sra/SRR25376689
Dj_18Q1208H1	2018	SRR25376700	PRJNA592186	Human	Australia	Campylobacter jejuni	128	ST-45 complex	https://www.ncbi.nlm.nih.gov/sra/SRR25376700
Dj_18Q1209H1	2018	SRR25376711	PRJNA592186	Human	Australia	Campylobacter jejuni	48	ST-48 complex	https://www.ncbi.nlm.nih.gov/sra/SRR25376711
Dj_18Q1210H1	2018	SRR25377062	PRJNA592186	Human	Australia	Campylobacter jejuni	4896	ST-353 complex	https://www.ncbi.nlm.nih.gov/sra/SRR25377062
Dj_18Q1211H1	2018	SRR25377073	PRJNA592186	Human	Australia	Campylobacter jejuni	46	ST-206 complex	https://www.ncbi.nlm.nih.gov/sra/SRR25377073
Dj_18Q1212H1	2018	SRR25377084	PRJNA592186	Human	Australia	Campylobacter jejuni	50	ST-21 complex	https://www.ncbi.nlm.nih.gov/sra/SRR25377084
Dj_18Q1213H1	2018	SRR25376723	PRJNA592186	Human	Australia	Campylobacter jejuni	4896	ST-353 complex	https://www.ncbi.nlm.nih.gov/sra/SRR25376723
Dj_18Q1214H1	2018	SRR25376734	PRJNA592186	Human	Australia	Campylobacter jejuni	46	ST-206 complex	https://www.ncbi.nlm.nih.gov/sra/SRR25376734
Dj_18Q1215H1	2018	SRR25376853	PRJNA592186	Human	Australia	Campylobacter jejuni	10130	ST-52 complex	https://www.ncbi.nlm.nih.gov/sra/SRR25376853
Dj_18Q1216H1	2018	SRR25376864	PRJNA592186	Human	Australia	Campylobacter jejuni	508	ST-508 complex	https://www.ncbi.nlm.nih.gov/sra/SRR25376864
Dj_18Q1217H1	2018	SRR25377087	PRJNA592186	Human	Australia	Campylobacter jejuni	2398		https://www.ncbi.nlm.nih.gov/sra/SRR25377087
Dj_18Q1219H1	2018	SRR25377109	PRJNA592186	Human	Australia	Campylobacter jejuni	53	ST-21 complex	https://www.ncbi.nlm.nih.gov/sra/SRR25377109
Dj_18Q1220H1	2018	SRR25376972	PRJNA592186	Human	Australia	Campylobacter jejuni	38	ST-48 complex	https://www.ncbi.nlm.nih.gov/sra/SRR25376972
Dj_18Q1223H1	2018	SRR25376994	PRJNA592186	Human	Australia	Campylobacter jejuni	48	ST-48 complex	https://www.ncbi.nlm.nih.gov/sra/SRR25376994
Dj_18Q1224H1	2018	SRR25377121	PRJNA592186	Human	Australia	Campylobacter jejuni	10132		https://www.ncbi.nlm.nih.gov/sra/SRR25377121
Dj_18Q1225H1	2018	SRR25377132	PRJNA592186	Human	Australia	Campylobacter jejuni	46	ST-206 complex	https://www.ncbi.nlm.nih.gov/sra/SRR25377132
Dj_18Q1227H1	2018	SRR25376762	PRJNA592186	Human	Australia	Campylobacter jejuni	933	ST-403 complex	https://www.ncbi.nlm.nih.gov/sra/SRR25376762
Dj_18Q1228H1	2018	SRR25376773	PRJNA592186	Human	Australia	Campylobacter jejuni	50	ST-21 complex	https://www.ncbi.nlm.nih.gov/sra/SRR25376773
Dj_18Q1229H1	2018	SRR25376880	PRJNA592186	Human	Australia	Campylobacter jejuni	10130	ST-52 complex	https://www.ncbi.nlm.nih.gov/sra/SRR25376880
Dj_18Q1230H1	2018	SRR25376891	PRJNA592186	Human	Australia	Campylobacter jejuni	2083		https://www.ncbi.nlm.nih.gov/sra/SRR25376891
Dj_18Q1231H1	2018	SRR25376902	PRJNA592186	Human	Australia	Campylobacter jejuni	45	ST-45 complex	https://www.ncbi.nlm.nih.gov/sra/SRR25376902
Dj_18Q1232H1	2018	SRR25376913	PRJNA592186	Human	Australia	Campylobacter jejuni	2083		https://www.ncbi.nlm.nih.gov/sra/SRR25376913
Dj_18Q1233H1	2018	SRR25376924	PRJNA592186	Human	Australia	Campylobacter jejuni	46	ST-206 complex	https://www.ncbi.nlm.nih.gov/sra/SRR25376924
Dj_18Q1234H1	2018	SRR25376935	PRJNA592186	Human	Australia	Campylobacter jejuni	567	ST-22 complex	https://www.ncbi.nlm.nih.gov/sra/SRR25376935
Dj_18Q1236H1	2018	SRR25376950	PRJNA592186	Human	Australia	Campylobacter jejuni	50	ST-21 complex	https://www.ncbi.nlm.nih.gov/sra/SRR25376950
Dj_18Q1237H1	2018	SRR25376961	PRJNA592186	Human	Australia	Campylobacter jejuni	50	ST-21 complex	https://www.ncbi.nlm.nih.gov/sra/SRR25376961
Dj_18Q1238H1	2018	SRR25376620	PRJNA592186	Human	Australia	Campylobacter jejuni	10132		https://www.ncbi.nlm.nih.gov/sra/SRR25376620
Dj_18Q1242H1	2018	SRR25376936	PRJNA592186	Human	Australia	Campylobacter jejuni	658	ST-658 complex	https://www.ncbi.nlm.nih.gov/sra/SRR25376936
Dj_18Q1243H1	2018	SRR25376937	PRJNA592186	Human	Australia	Campylobacter jejuni	528	ST-354 complex	https://www.ncbi.nlm.nih.gov/sra/SRR25376937
Dj_18Q1245H1	2018	SRR25376882	PRJNA592186	Human	Australia	Campylobacter jejuni	61	ST-61 complex	https://www.ncbi.nlm.nih.gov/sra/SRR25376882
Dj_18Q1246H1	2018	SRR25376645	PRJNA592186	Human	Australia	Campylobacter jejuni	2083		https://www.ncbi.nlm.nih.gov/sra/SRR25376645
Dj_18Q1249H1	2018	SRR25376648	PRJNA592186	Human	Australia	Campylobacter jejuni	2398		https://www.ncbi.nlm.nih.gov/sra/SRR25376648
Dj_18Q1251H1	2018	SRR25376778	PRJNA592186	Human	Australia	Campylobacter jejuni	10127	ST-257 complex	https://www.ncbi.nlm.nih.gov/sra/SRR25376778
Dj_18Q1252H1	2018	SRR25376779	PRJNA592186	Human	Australia	Campylobacter jejuni	658	ST-658 complex	https://www.ncbi.nlm.nih.gov/sra/SRR25376779
Dj_18Q1254H1	2018	SRR25376782	PRJNA592186	Human	Australia	Campylobacter jejuni	354	ST-354 complex	https://www.ncbi.nlm.nih.gov/sra/SRR25376782
Dj_18Q1255H1	2018	SRR25376783	PRJNA592186	Human	Australia	Campylobacter jejuni	10143	ST-353 complex	https://www.ncbi.nlm.nih.gov/sra/SRR25376783
Dj_18Q1257H1	2018	SRR25376786	PRJNA592186	Human	Australia	Campylobacter jejuni	2083		https://www.ncbi.nlm.nih.gov/sra/SRR25376786
Dj_18Q1261H1	2018	SRR25376789	PRJNA592186	Human	Australia	Campylobacter jejuni	50	ST-21 complex	https://www.ncbi.nlm.nih.gov/sra/SRR25376789
Dj_18Q1262H1	2018	SRR25376790	PRJNA592186	Human	Australia	Campylobacter jejuni	449	ST-581 complex	https://www.ncbi.nlm.nih.gov/sra/SRR25376790
Dj_18Q1263H1	2018	SRR25376791	PRJNA592186	Human	Australia	Campylobacter jejuni	50	ST-21 complex	https://www.ncbi.nlm.nih.gov/sra/SRR25376791
Dj_18Q1266H1	2018	SRR25376795	PRJNA592186	Human	Australia	Campylobacter jejuni	45	ST-45 complex	https://www.ncbi.nlm.nih.gov/sra/SRR25376795
Dj_18Q1267H1	2018	SRR25376797	PRJNA592186	Human	Australia	Campylobacter jejuni	7323		https://www.ncbi.nlm.nih.gov/sra/SRR25376797
Dj_18Q1269H1	2018	SRR25376799	PRJNA592186	Human	Australia	Campylobacter jejuni	53	ST-21 complex	https://www.ncbi.nlm.nih.gov/sra/SRR25376799
Dj_18Q1270H1	2018	SRR25376800	PRJNA592186	Human	Australia	Campylobacter jejuni	10138	ST-21 complex	https://www.ncbi.nlm.nih.gov/sra/SRR25376800
Dj_18Q1271H1	2018	SRR25376801	PRJNA592186	Human	Australia	Campylobacter jejuni	2083		https://www.ncbi.nlm.nih.gov/sra/SRR25376801
Dj_18Q1272H1	2018	SRR25376802	PRJNA592186	Human	Australia	Campylobacter jejuni	46	ST-206 complex	https://www.ncbi.nlm.nih.gov/sra/SRR25376802
Dj_18Q1274H1	2018	SRR25376805	PRJNA592186	Human	Australia	Campylobacter jejuni	4896	ST-353 complex	https://www.ncbi.nlm.nih.gov/sra/SRR25376805
Dj_18Q1277H1	2018	SRR25376809	PRJNA592186	Human	Australia	Campylobacter jejuni	525	ST-607 complex	https://www.ncbi.nlm.nih.gov/sra/SRR25376809
Dj_18Q1280H1	2018	SRR11318664	PRJNA592186	Human	Australia	Campylobacter jejuni	528	ST-354 complex	https://www.ncbi.nlm.nih.gov/sra/SRR11318664
Dj_18Q1281H1	2018	SRR25376651	PRJNA592186	Human	Australia	Campylobacter jejuni	50	ST-21 complex	https://www.ncbi.nlm.nih.gov/sra/SRR25376651
Dj_18Q1282H1	2018	SRR25376652	PRJNA592186	Human	Australia	Campylobacter jejuni	2398		https://www.ncbi.nlm.nih.gov/sra/SRR25376652
Dj_18Q1283H1	2018	SRR25376653	PRJNA592186	Human	Australia	Campylobacter jejuni	534		https://www.ncbi.nlm.nih.gov/sra/SRR25376653
Dj_18Q1284H1	2018	SRR25376655	PRJNA592186	Human	Australia	Campylobacter jejuni	567	ST-22 complex	https://www.ncbi.nlm.nih.gov/sra/SRR25376655
Dj_18Q1285H1	2018	SRR25376656	PRJNA592186	Human	Australia	Campylobacter jejuni	45	ST-45 complex	https://www.ncbi.nlm.nih.gov/sra/SRR25376656
Dj_18Q1286H1	2018	SRR25376657	PRJNA592186	Human	Australia	Campylobacter jejuni	50	ST-21 complex	https://www.ncbi.nlm.nih.gov/sra/SRR25376657
Dj_18Q1287H1	2018	SRR25376658	PRJNA592186	Human	Australia	Campylobacter jejuni	50	ST-21 complex	https://www.ncbi.nlm.nih.gov/sra/SRR25376658
Dj_18Q1288H1	2018	SRR25376659	PRJNA592186	Human	Australia	Campylobacter jejuni	696	ST-1332 complex	https://www.ncbi.nlm.nih.gov/sra/SRR25376659
Dj_18Q1289H1	2018	SRR25376661	PRJNA592186	Human	Australia	Campylobacter jejuni	2083		https://www.ncbi.nlm.nih.gov/sra/SRR25376661
Dj_18Q1290H1	2018	SRR25376662	PRJNA592186	Human	Australia	Campylobacter jejuni	51	ST-443 complex	https://www.ncbi.nlm.nih.gov/sra/SRR25376662
Dj_18Q1291H1	2018	SRR25376663	PRJNA592186	Human	Australia	Campylobacter jejuni	10152		https://www.ncbi.nlm.nih.gov/sra/SRR25376663
Dj_18Q1292H1	2018	SRR25376664	PRJNA592186	Human	Australia	Campylobacter jejuni	46	ST-206 complex	https://www.ncbi.nlm.nih.gov/sra/SRR25376664
Dj_18Q1293H1	2018	SRR25376666	PRJNA592186	Human	Australia	Campylobacter jejuni	21	ST-21 complex	https://www.ncbi.nlm.nih.gov/sra/SRR25376666
Dj_18Q1294H1	2018	SRR25376667	PRJNA592186	Human	Australia	Campylobacter jejuni	50	ST-21 complex	https://www.ncbi.nlm.nih.gov/sra/SRR25376667
Dj_18Q1295H1	2018	SRR25376668	PRJNA592186	Human	Australia	Campylobacter jejuni	4577	ST-179 complex	https://www.ncbi.nlm.nih.gov/sra/SRR25376668
Dj_18Q1296H1	2018	SRR25376669	PRJNA592186	Human	Australia	Campylobacter jejuni	50	ST-21 complex	https://www.ncbi.nlm.nih.gov/sra/SRR25376669
Dj_18Q1299H1	2018	SRR25376673	PRJNA592186	Human	Australia	Campylobacter jejuni	528	ST-354 complex	https://www.ncbi.nlm.nih.gov/sra/SRR25376673
Dj_18Q1300H1	2018	SRR25376674	PRJNA592186	Human	Australia	Campylobacter jejuni	2398		https://www.ncbi.nlm.nih.gov/sra/SRR25376674
Dj_18V1004H1	2018	SRR25376836	PRJNA592186	Human	Australia	Campylobacter jejuni	137	ST-45 complex	https://www.ncbi.nlm.nih.gov/sra/SRR25376836
Dj_18V1006H1	2018	SRR25376840	PRJNA592186	Human	Australia	Campylobacter jejuni	50	ST-21 complex	https://www.ncbi.nlm.nih.gov/sra/SRR25376840
Dj_18V1008H1	2018	SRR25376841	PRJNA592186	Human	Australia	Campylobacter jejuni	567	ST-22 complex	https://www.ncbi.nlm.nih.gov/sra/SRR25376841
Dj_18V1014H1	2018	SRR25376682	PRJNA592186	Human	Australia	Campylobacter jejuni	50	ST-21 complex	https://www.ncbi.nlm.nih.gov/sra/SRR25376682
Dj_18V1017H1	2018	SRR25376684	PRJNA592186	Human	Australia	Campylobacter jejuni	42	ST-42 complex	https://www.ncbi.nlm.nih.gov/sra/SRR25376684
Dj_18V1018H1	2018	SRR25376685	PRJNA592186	Human	Australia	Campylobacter jejuni	49	ST-49 complex	https://www.ncbi.nlm.nih.gov/sra/SRR25376685
Dj_18V1023H1	2018	SRR25377071	PRJNA592186	Human	Australia	Campylobacter jejuni	190	ST-21 complex	https://www.ncbi.nlm.nih.gov/sra/SRR25377071
Dj_18V1025H1	2018	SRR25377072	PRJNA592186	Human	Australia	Campylobacter jejuni	50	ST-21 complex	https://www.ncbi.nlm.nih.gov/sra/SRR25377072
Dj_18V1026H1	2018	SRR25377079	PRJNA592186	Human	Australia	Campylobacter jejuni	8516		https://www.ncbi.nlm.nih.gov/sra/SRR25377079
Dj_18V1027H1	2018	SRR25377080	PRJNA592186	Human	Australia	Campylobacter jejuni	61	ST-61 complex	https://www.ncbi.nlm.nih.gov/sra/SRR25377080
Dj_18V1030H1	2018	SRR25377082	PRJNA592186	Human	Australia	Campylobacter jejuni	2580	ST-42 complex	https://www.ncbi.nlm.nih.gov/sra/SRR25377082
Dj_18V1032H1	2018	SRR25377083	PRJNA592186	Human	Australia	Campylobacter jejuni	7323		https://www.ncbi.nlm.nih.gov/sra/SRR25377083
Dj_18V1037H1	2018	SRR25376714	PRJNA592186	Human	Australia	Campylobacter jejuni	2083		https://www.ncbi.nlm.nih.gov/sra/SRR25376714

Isolate no.	Year	Accession no.	BioProject no.	Source	Country	Species	Sequence type	Clonal complex	SRA hyperlink
Dj_18V1040H1	2018	SRR25376715	PRJNA592186	Human	Australia	Campylobacter jejuni	21	ST-21 complex	https://www.ncbi.nlm.nih.gov/sra/SRR25376715
Dj_18V1041H1	2018	SRR25376716	PRJNA592186	Human	Australia	Campylobacter jejuni	21	ST-21 complex	https://www.ncbi.nlm.nih.gov/sra/SRR25376716
Dj_18V1042H1	2018	SRR25376717	PRJNA592186	Human	Australia	Campylobacter jejuni	4896	ST-353 complex	https://www.ncbi.nlm.nih.gov/sra/SRR25376717
Dj_18V1043H1	2018	SRR25376720	PRJNA592186	Human	Australia	Campylobacter jejuni	51	ST-443 complex	https://www.ncbi.nlm.nih.gov/sra/SRR25376720
Dj_18V1044H1	2018	SRR25376721	PRJNA592186	Human	Australia	Campylobacter jejuni	10155		https://www.ncbi.nlm.nih.gov/sra/SRR25376721
Dj_18V1045H1	2018	SRR25376722	PRJNA592186	Human	Australia	Campylobacter jejuni	2349		https://www.ncbi.nlm.nih.gov/sra/SRR25376722
Dj_18V1046H1	2018	SRR25376724	PRJNA592186	Human	Australia	Campylobacter jejuni	190	ST-21 complex	https://www.ncbi.nlm.nih.gov/sra/SRR25376724
Dj_18V1051H1	2018	SRR25376725	PRJNA592186	Human	Australia	Campylobacter jejuni	51	ST-443 complex	https://www.ncbi.nlm.nih.gov/sra/SRR25376725
Dj_18V1053H1	2018	SRR25376727	PRJNA592186	Human	Australia	Campylobacter jejuni	528	ST-354 complex	https://www.ncbi.nlm.nih.gov/sra/SRR25376727
Dj_18V1054H1	2018	SRR25376728	PRJNA592186	Human	Australia	Campylobacter jejuni	51	ST-443 complex	https://www.ncbi.nlm.nih.gov/sra/SRR25376728
Dj_18V1055H1	2018	SRR25376729	PRJNA592186	Human	Australia	Campylobacter jejuni	50	ST-21 complex	https://www.ncbi.nlm.nih.gov/sra/SRR25376729
Dj_18V1056H1	2018	SRR25376730	PRJNA592186	Human	Australia	Campylobacter jejuni	46	ST-206 complex	https://www.ncbi.nlm.nih.gov/sra/SRR25376730
Dj_18V1057H1	2018	SRR25376732	PRJNA592186	Human	Australia	Campylobacter jejuni	49	ST-49 complex	https://www.ncbi.nlm.nih.gov/sra/SRR25376732
Dj_18V1060H1	2018	SRR25376733	PRJNA592186	Human	Australia	Campylobacter jejuni	696	ST-1332 complex	https://www.ncbi.nlm.nih.gov/sra/SRR25376733
Dj_18V1062H1	2018	SRR25376735	PRJNA592186	Human	Australia	Campylobacter jejuni	21	ST-21 complex	https://www.ncbi.nlm.nih.gov/sra/SRR25376735
Dj_18V1063H1	2018	SRR25376736	PRJNA592186	Human	Australia	Campylobacter jejuni	10155		https://www.ncbi.nlm.nih.gov/sra/SRR25376736
Dj_18V1064H1	2018	SRR25376737	PRJNA592186	Human	Australia	Campylobacter jejuni	22	ST-22 complex	https://www.ncbi.nlm.nih.gov/sra/SRR25376737
Dj_18V1066H1	2018	SRR25376739	PRJNA592186	Human	Australia	Campylobacter jejuni	696	ST-1332 complex	https://www.ncbi.nlm.nih.gov/sra/SRR25376739
Dj_18V1068H1	2018	SRR25376741	PRJNA592186	Human	Australia	Campylobacter jejuni	257	ST-257 complex	https://www.ncbi.nlm.nih.gov/sra/SRR25376741
Dj_18V1073H1	2018	SRR25376843	PRJNA592186	Human	Australia	Campylobacter jejuni	583	ST-45 complex	https://www.ncbi.nlm.nih.gov/sra/SRR25376843
Dj_18V1074H1	2018	SRR25376844	PRJNA592186	Human	Australia	Campylobacter jejuni	190	ST-21 complex	https://www.ncbi.nlm.nih.gov/sra/SRR25376844
Dj_18V1076H1	2018	SRR25376846	PRJNA592186	Human	Australia	Campylobacter jejuni	2083		https://www.ncbi.nlm.nih.gov/sra/SRR25376846
Dj_18V1097H1	2018	SRR25376847	PRJNA592186	Human	Australia	Campylobacter jejuni	567	ST-22 complex	https://www.ncbi.nlm.nih.gov/sra/SRR25376847
Dj_18V1098H1	2018	SRR25376848	PRJNA592186	Human	Australia	Campylobacter jejuni	128	ST-45 complex	https://www.ncbi.nlm.nih.gov/sra/SRR25376848
Dj_18V1099H1	2018	SRR25376849	PRJNA592186	Human	Australia	Campylobacter jejuni	45	ST-45 complex	https://www.ncbi.nlm.nih.gov/sra/SRR25376849
Dj_19A1006H1	2019	SRR25377008	PRJNA592186	Human	Australia	Campylobacter jejuni	48	ST-48 complex	https://www.ncbi.nlm.nih.gov/sra/SRR25377008
Dj_19A1007H1	2019	SRR25377020	PRJNA592186	Human	Australia	Campylobacter jejuni	508	ST-508 complex	https://www.ncbi.nlm.nih.gov/sra/SRR25377020
Dj_19A1008H1	2019	SRR25376948	PRJNA592186	Human	Australia	Campylobacter jejuni	2083		https://www.ncbi.nlm.nih.gov/sra/SRR25376948
Dj_19A1009H1	2019	SRR25376962	PRJNA592186	Human	Australia	Campylobacter jejuni	48	ST-48 complex	https://www.ncbi.nlm.nih.gov/sra/SRR25376962
Dj_19A1012H1	2019	SRR25376642	PRJNA592186	Human	Australia	Campylobacter jejuni	50	ST-21 complex	https://www.ncbi.nlm.nih.gov/sra/SRR25376642
Dj_19A1013H1	2019	SRR25376617	PRJNA592186	Human	Australia	Campylobacter jejuni	567	ST-22 complex	https://www.ncbi.nlm.nih.gov/sra/SRR25376617
Dj_19A1014H1	2019	SRR25376757	PRJNA592186	Human	Australia	Campylobacter jejuni	46	ST-206 complex	https://www.ncbi.nlm.nih.gov/sra/SRR25376757
Dj_19A1015H1	2019	SRR25376769	PRJNA592186	Human	Australia	Campylobacter jejuni	50	ST-21 complex	https://www.ncbi.nlm.nih.gov/sra/SRR25376769
Dj_19A1016H1	2019	SRR25376877	PRJNA592186	Human	Australia	Campylobacter jejuni	46	ST-206 complex	https://www.ncbi.nlm.nih.gov/sra/SRR25376877
Dj_19A1017H1	2019	SRR25376815	PRJNA592186	Human	Australia	Campylobacter jejuni	50	ST-21 complex	https://www.ncbi.nlm.nih.gov/sra/SRR25376815
Dj_19A1018H1	2019	SRR25376745	PRJNA592186	Human	Australia	Campylobacter jejuni	50	ST-21 complex	https://www.ncbi.nlm.nih.gov/sra/SRR25376745
Dj_19A1019H1	2019	SRR25376612	PRJNA592186	Human	Australia	Campylobacter jejuni	538	ST-45 complex	https://www.ncbi.nlm.nih.gov/sra/SRR25376612
Dj_19A1020H1	2019	SRR25376939	PRJNA592186	Human	Australia	Campylobacter jejuni	257	ST-257 complex	https://www.ncbi.nlm.nih.gov/sra/SRR25376939
Dj_19A1021H1	2019	SRR25376884	PRJNA592186	Human	Australia	Campylobacter jejuni	520	ST-21 complex	https://www.ncbi.nlm.nih.gov/sra/SRR25376884
Dj_19A1022H1	2019	SRR25376784	PRJNA592186	Human	Australia	Campylobacter jejuni	538	ST-45 complex	https://www.ncbi.nlm.nih.gov/sra/SRR25376784
Dj_19A1023H1	2019	SRR25376796	PRJNA592186	Human	Australia	Campylobacter jejuni	21	ST-21 complex	https://www.ncbi.nlm.nih.gov/sra/SRR25376796
Dj_19A1024H1	2019	SRR25376808	PRJNA592186	Human	Australia	Campylobacter jejuni	52	ST-52 complex	https://www.ncbi.nlm.nih.gov/sra/SRR25376808
Dj_19A1025H1	2019	SRR25376660	PRJNA592186	Human	Australia	Campylobacter jejuni	46	ST-206 complex	https://www.ncbi.nlm.nih.gov/sra/SRR25376660
Dj_19A1026H1	2019	SRR25376672	PRJNA592186	Human	Australia	Campylobacter jejuni	2083		https://www.ncbi.nlm.nih.gov/sra/SRR25376672
Dj_19A1027H1	2019	SRR25376812	PRJNA592186	Human	Australia	Campylobacter jejuni	6891	ST-21 complex	https://www.ncbi.nlm.nih.gov/sra/SRR25376812
Dj_19A1028H1	2019	SRR25376824	PRJNA592186	Human	Australia	Campylobacter jejuni	46	ST-206 complex	https://www.ncbi.nlm.nih.gov/sra/SRR25376824
Dj_19A1029H1	2019	SRR25376837	PRJNA592186	Human	Australia	Campylobacter jejuni	257	ST-257 complex	https://www.ncbi.nlm.nih.gov/sra/SRR25376837
Dj_19A1030H1	2019	SRR25377078	PRJNA592186	Human	Australia	Campylobacter jejuni	2083		https://www.ncbi.nlm.nih.gov/sra/SRR25377078
Dj_19A1031H1	2019	SRR25376719	PRJNA592186	Human	Australia	Campylobacter jejuni	50	ST-21 complex	https://www.ncbi.nlm.nih.gov/sra/SRR25376719
Dj_19N1002H1	2019	SRR25376688	PRJNA592186	Human	Australia	Campylobacter jejuni	2398		https://www.ncbi.nlm.nih.gov/sra/SRR25376688
Dj_19N1004H1	2019	SRR25376690	PRJNA592186	Human	Australia	Campylobacter jejuni	10132		https://www.ncbi.nlm.nih.gov/sra/SRR25376690
Dj_19N1005H1	2019	SRR25376692	PRJNA592186	Human	Australia	Campylobacter jejuni	50	ST-21 complex	https://www.ncbi.nlm.nih.gov/sra/SRR25376692
Dj_19N1008H1	2019	SRR25376694	PRJNA592186	Human	Australia	Campylobacter jejuni	658	ST-658 complex	https://www.ncbi.nlm.nih.gov/sra/SRR25376694
Dj_19N1016H1	2019	SRR25376695	PRJNA592186	Human	Australia	Campylobacter jejuni	10132		https://www.ncbi.nlm.nih.gov/sra/SRR25376695
Dj_19N1026H1	2019	SRR25376697	PRJNA592186	Human	Australia	Campylobacter jejuni	538	ST-45 complex	https://www.ncbi.nlm.nih.gov/sra/SRR25376697
Dj_19N1027H1	2019	SRR25376698	PRJNA592186	Human	Australia	Campylobacter jejuni	567	ST-22 complex	https://www.ncbi.nlm.nih.gov/sra/SRR25376698
Dj_19N1028H1	2019	SRR25376699	PRJNA592186	Human	Australia	Campylobacter jejuni	48	ST-48 complex	https://www.ncbi.nlm.nih.gov/sra/SRR25376699
Dj_19N1029H1	2019	SRR25376701	PRJNA592186	Human	Australia	Campylobacter jejuni	50	ST-21 complex	https://www.ncbi.nlm.nih.gov/sra/SRR25376701
Dj_19N1035H1	2019	SRR25376702	PRJNA592186	Human	Australia	Campylobacter jejuni	257	ST-257 complex	https://www.ncbi.nlm.nih.gov/sra/SRR25376702
Dj_19N1036H1	2019	SRR25376704	PRJNA592186	Human	Australia	Campylobacter jejuni	48	ST-48 complex	https://www.ncbi.nlm.nih.gov/sra/SRR25376704
Dj_19N1038H1	2019	SRR25376705	PRJNA592186	Human	Australia	Campylobacter jejuni	257	ST-257 complex	https://www.ncbi.nlm.nih.gov/sra/SRR25376705
Dj_19N1039H1	2019	SRR25376706	PRJNA592186	Human	Australia	Campylobacter jejuni	50	ST-21 complex	https://www.ncbi.nlm.nih.gov/sra/SRR25376706
Dj_19N1040H1	2019	SRR25376707	PRJNA592186	Human	Australia	Campylobacter jejuni	50	ST-21 complex	https://www.ncbi.nlm.nih.gov/sra/SRR25376707
Dj_19N1042H1	2019	SRR25376708	PRJNA592186	Human	Australia	Campylobacter jejuni	52	ST-52 complex	https://www.ncbi.nlm.nih.gov/sra/SRR25376708
Dj_19N1044H1	2019	SRR25376709	PRJNA592186	Human	Australia	Campylobacter jejuni	45	ST-45 complex	https://www.ncbi.nlm.nih.gov/sra/SRR25376709
Dj_19N1048H1	2019	SRR25376710	PRJNA592186	Human	Australia	Campylobacter jejuni	696	ST-1332 complex	https://www.ncbi.nlm.nih.gov/sra/SRR25376710
Dj_19N1050H1	2019	SRR25376712	PRJNA592186	Human	Australia	Campylobacter jejuni	50	ST-21 complex	https://www.ncbi.nlm.nih.gov/sra/SRR25376712
Dj_19N1055H1	2019	SRR25376713	PRJNA592186	Human	Australia	Campylobacter jejuni	50	ST-21 complex	https://www.ncbi.nlm.nih.gov/sra/SRR25376713
Dj_19N1056H1	2019	SRR25377054	PRJNA592186	Human	Australia	Campylobacter jejuni	10132		https://www.ncbi.nlm.nih.gov/sra/SRR25377054
Dj_19N1060H1	2019	SRR25377056	PRJNA592186	Human	Australia	Campylobacter jejuni	42	ST-42 complex	https://www.ncbi.nlm.nih.gov/sra/SRR25377056
Dj_19N1063H1	2019	SRR25377057	PRJNA592186	Human	Australia	Campylobacter jejuni	46	ST-206 complex	https://www.ncbi.nlm.nih.gov/sra/SRR25377057
Dj_19N1069H1	2019	SRR25377059	PRJNA592186	Human	Australia	Campylobacter jejuni	583	ST-45 complex	https://www.ncbi.nlm.nih.gov/sra/SRR25377059
Dj_19N1071H1	2019	SRR25377060	PRJNA592186	Human	Australia	Campylobacter jejuni	50	ST-21 complex	https://www.ncbi.nlm.nih.gov/sra/SRR25377060
Dj_19N1072H1	2019	SRR11318670	PRJNA592186	Human	Australia	Campylobacter jejuni	45	ST-45 complex	https://www.ncbi.nlm.nih.gov/sra/SRR11318670
Dj_19N1074H1	2019	SRR25376873	PRJNA592186	Human	Australia	Campylobacter jejuni	46	ST-206 complex	https://www.ncbi.nlm.nih.gov/sra/SRR25376873
Dj_19N1075H1	2019	SRR25377086	PRJNA592186	Human	Australia	Campylobacter jejuni	190	ST-21 complex	https://www.ncbi.nlm.nih.gov/sra/SRR25377086
Dj_19N1076H1	2019	SRR25377088	PRJNA592186	Human	Australia	Campylobacter jejuni	1525		https://www.ncbi.nlm.nih.gov/sra/SRR25377088
Dj_19N1081H1	2019	SRR25377093	PRJNA592186	Human	Australia	Campylobacter jejuni	2343	ST-48 complex	https://www.ncbi.nlm.nih.gov/sra/SRR25377093
Dj_19N1082H1	2019	SRR25377094	PRJNA592186	Human	Australia	Campylobacter jejuni	534		https://www.ncbi.nlm.nih.gov/sra/SRR25377094
Dj_19N1083H1	2019	SRR25377095	PRJNA592186	Human	Australia	Campylobacter jejuni	50	ST-21 complex	https://www.ncbi.nlm.nih.gov/sra/SRR25377095
Dj_19N1084H1	2019	SRR25377096	PRJNA592186	Human	Australia	Campylobacter jejuni	10132		https://www.ncbi.nlm.nih.gov/sra/SRR25377096
Dj_19N1085H1	2019	SRR25377097	PRJNA592186	Human	Australia	Campylobacter jejuni	46	ST-206 complex	https://www.ncbi.nlm.nih.gov/sra/SRR25377097
Dj_19N1087H1	2019	SRR25377099	PRJNA592186	Human	Australia	Campylobacter jejuni	528	ST-354 complex	https://www.ncbi.nlm.nih.gov/sra/SRR25377099
Dj_19N1090H1	2019	SRR25377101	PRJNA592186	Human	Australia	Campylobacter jejuni	50	ST-21 complex	https://www.ncbi.nlm.nih.gov/sra/SRR25377101
Dj_19N1094H1	2019	SRR25377104	PRJNA592186	Human	Australia	Campylobacter jejuni	2398		https://www.ncbi.nlm.nih.gov/sra/SRR25377104
Dj_19N1096H1	2019	SRR25377105	PRJNA592186	Human	Australia	Campylobacter jejuni	696	ST-1332 complex	https://www.ncbi.nlm.nih.gov/sra/SRR25377105
Dj_19N1098H1	2019	SRR25377106	PRJNA592186	Human	Australia	Campylobacter jejuni	528	ST-354 complex	https://www.ncbi.nlm.nih.gov/sra/SRR25377106

Isolate no.	Year	Accession no.	BioProject no.	Source	Country	Species	Sequence type	Clonal complex	SRA hyperlink
Dj_19N1099H1	2019	SRR25377107	PRJNA592186	Human	Australia	Campylobacter jejuni	538	ST-45 complex	https://www.ncbi.nlm.nih.gov/sra/SRR25377107
Dj_19N1100H1	2019	SRR11318659	PRJNA592186	Human	Australia	Campylobacter jejuni	508	ST-508 complex	https://www.ncbi.nlm.nih.gov/sra/SRR11318659
Dj_19N1101H1	2019	SRR25377108	PRJNA592186	Human	Australia	Campylobacter jejuni	21	ST-21 complex	https://www.ncbi.nlm.nih.gov/sra/SRR25377108
Dj_19N1102H1	2019	SRR25377110	PRJNA592186	Human	Australia	Campylobacter jejuni	1911		https://www.ncbi.nlm.nih.gov/sra/SRR25377110
Dj_19N1103H1	2019	SRR25377112	PRJNA592186	Human	Australia	Campylobacter jejuni	53	ST-21 complex	https://www.ncbi.nlm.nih.gov/sra/SRR25377112
Dj_19N1104H1	2019	SRR25377113	PRJNA592186	Human	Australia	Campylobacter jejuni	2398		https://www.ncbi.nlm.nih.gov/sra/SRR25377113
Dj_19N1109H1	2019	SRR25377116	PRJNA592186	Human	Australia	Campylobacter jejuni	61	ST-61 complex	https://www.ncbi.nlm.nih.gov/sra/SRR25377116
Dj_19N1111H1	2019	SRR25377117	PRJNA592186	Human	Australia	Campylobacter jejuni	538	ST-45 complex	https://www.ncbi.nlm.nih.gov/sra/SRR25377117
Dj_19N1112H1	2019	SRR25376970	PRJNA592186	Human	Australia	Campylobacter jejuni	50	ST-21 complex	https://www.ncbi.nlm.nih.gov/sra/SRR25376970
Dj_19N1113H1	2019	SRR25376971	PRJNA592186	Human	Australia	Campylobacter jejuni	42	ST-42 complex	https://www.ncbi.nlm.nih.gov/sra/SRR25376971
Dj_19N1114H1	2019	SRR25376973	PRJNA592186	Human	Australia	Campylobacter jejuni	50	ST-21 complex	https://www.ncbi.nlm.nih.gov/sra/SRR25376973
Dj_19N1115H1	2019	SRR25376974	PRJNA592186	Human	Australia	Campylobacter jejuni	528	ST-354 complex	https://www.ncbi.nlm.nih.gov/sra/SRR25376974
Dj_19N1131H1	2019	SRR11318671	PRJNA592186	Human	Australia	Campylobacter jejuni	52	ST-52 complex	https://www.ncbi.nlm.nih.gov/sra/SRR11318671
Dj_19N1186H1	2019	SRR11318656	PRJNA592186	Human	Australia	Campylobacter jejuni	45	ST-45 complex	https://www.ncbi.nlm.nih.gov/sra/SRR11318656
Dj_19N1187H1	2019	SRR11318655	PRJNA592186	Human	Australia	Campylobacter jejuni	820	ST-61 complex	https://www.ncbi.nlm.nih.gov/sra/SRR11318655
Dj_19N1188H1	2019	SRR11318654	PRJNA592186	Human	Australia	Campylobacter jejuni	10132		https://www.ncbi.nlm.nih.gov/sra/SRR11318654
Dj_19N1190H1	2019	SRR11318653	PRJNA592186	Human	Australia	Campylobacter jejuni	50	ST-21 complex	https://www.ncbi.nlm.nih.gov/sra/SRR11318653
Dj_19N1192H1	2019	SRR11318652	PRJNA592186	Human	Australia	Campylobacter jejuni	50	ST-21 complex	https://www.ncbi.nlm.nih.gov/sra/SRR11318652
Dj_19N1193H1	2019	SRR11318651	PRJNA592186	Human	Australia	Campylobacter jejuni	50	ST-21 complex	https://www.ncbi.nlm.nih.gov/sra/SRR11318651
Dj_19V1001H1	2019	SRR25376850	PRJNA592186	Human	Australia	Campylobacter jejuni	21	ST-21 complex	https://www.ncbi.nlm.nih.gov/sra/SRR25376850
Dj_19V1002H1	2019	SRR25377026	PRJNA592186	Human	Australia	Campylobacter jejuni	2398		https://www.ncbi.nlm.nih.gov/sra/SRR25377026
Dj_19V1004H1	2019	SRR25377028	PRJNA592186	Human	Australia	Campylobacter jejuni	567	ST-22 complex	https://www.ncbi.nlm.nih.gov/sra/SRR25377028
Dj_19V1005H1	2019	SRR25377029	PRJNA592186	Human	Australia	Campylobacter jejuni	52	ST-52 complex	https://www.ncbi.nlm.nih.gov/sra/SRR25377029
Ec_SC0098	2018	SRR17926691	PRJNA675916	Human	New Zealand	Campylobacter coli	2256	ST-828 complex	https://www.ncbi.nlm.nih.gov/sra/SRR17926691
Ec_SC0111	2018	SRR17899275	PRJNA675916	Chicken	New Zealand	Campylobacter coli	825	ST-828 complex	https://www.ncbi.nlm.nih.gov/sra/SRR17899275
Ec_SC0176	2018	SRR17899206	PRJNA675916	Human	New Zealand	Campylobacter coli	4009	ST-828 complex	https://www.ncbi.nlm.nih.gov/sra/SRR17899206
Ec_SC0187	2018	SRR17899194	PRJNA675916	Chicken	New Zealand	Campylobacter coli	2256	ST-828 complex	https://www.ncbi.nlm.nih.gov/sra/SRR17899194
Ec_SC0194	2018	SRR17899186	PRJNA675916	Human	New Zealand	Campylobacter coli	825	ST-828 complex	https://www.ncbi.nlm.nih.gov/sra/SRR17899186
Ec_SC0231	2018	SRR17900365	PRJNA675916	Human	New Zealand	Campylobacter coli	4009	ST-828 complex	https://www.ncbi.nlm.nih.gov/sra/SRR17900365
Ec_SC0280	2018	SRR17900313	PRJNA675916	Human	New Zealand	Campylobacter coli	2256	ST-828 complex	https://www.ncbi.nlm.nih.gov/sra/SRR17900313
Ec_SC0286	2018	SRR17900306	PRJNA675916	Human	New Zealand	Campylobacter coli	8926	ST-828 complex	https://www.ncbi.nlm.nih.gov/sra/SRR17900306
Ec_SC0352	2018	SRR17900464	PRJNA675916	Human	New Zealand	Campylobacter coli	12248		https://www.ncbi.nlm.nih.gov/sra/SRR17900464
Ec_SC0361	2018	SRR17900455	PRJNA675916	Human	New Zealand	Campylobacter coli	827	ST-828 complex	https://www.ncbi.nlm.nih.gov/sra/SRR17900455
Ec_SC0391	2018	SRR17900359	PRJNA675916	Human	New Zealand	Campylobacter coli	4009	ST-828 complex	https://www.ncbi.nlm.nih.gov/sra/SRR17900359
Ec_SC0396	2018	SRR17900353	PRJNA675916	Human	New Zealand	Campylobacter coli	825	ST-828 complex	https://www.ncbi.nlm.nih.gov/sra/SRR17900353
Ec_SC0408	2018	SRR17900339	PRJNA675916	Chicken	New Zealand	Campylobacter coli	2256	ST-828 complex	https://www.ncbi.nlm.nih.gov/sra/SRR17900339
Ec_SC0453	2018	SRR17900577	PRJNA675916	Chicken	New Zealand	Campylobacter coli	2256	ST-828 complex	https://www.ncbi.nlm.nih.gov/sra/SRR17900577
Ec_SC0454	2018	SRR17900576	PRJNA675916	Chicken	New Zealand	Campylobacter coli	2256	ST-828 complex	https://www.ncbi.nlm.nih.gov/sra/SRR17900576
Ec_SC0475	2018	SRR17900393	PRJNA675916	Chicken	New Zealand	Campylobacter coli	8712	ST-828 complex	https://www.ncbi.nlm.nih.gov/sra/SRR17900393
Ec_SC0476	2018	SRR17900392	PRJNA675916	Chicken	New Zealand	Campylobacter coli	2256	ST-828 complex	https://www.ncbi.nlm.nih.gov/sra/SRR17900392
Ec_SC0494	2018	SRR17900373	PRJNA675916	Human	New Zealand	Campylobacter coli	8926	ST-828 complex	https://www.ncbi.nlm.nih.gov/sra/SRR17900373
Ec_SC0504	2018	SRR17925266	PRJNA675916	Chicken	New Zealand	Campylobacter coli	8926	ST-828 complex	https://www.ncbi.nlm.nih.gov/sra/SRR17925266
Ec_SC0540	2018	SRR17925434	PRJNA675916	Human	New Zealand	Campylobacter coli	3072	ST-828 complex	https://www.ncbi.nlm.nih.gov/sra/SRR17925434
Ec_SC0594	2018	SRR17925118	PRJNA675916	Human	New Zealand	Campylobacter coli	8926	ST-828 complex	https://www.ncbi.nlm.nih.gov/sra/SRR17925118
Ec_SC0613	2018	SRR17925473	PRJNA675916	Chicken	New Zealand	Campylobacter coli	828	ST-828 complex	https://www.ncbi.nlm.nih.gov/sra/SRR17925473
Ec_SC0618	2018	SRR17925468	PRJNA675916	Chicken	New Zealand	Campylobacter coli	8926	ST-828 complex	https://www.ncbi.nlm.nih.gov/sra/SRR17925468
Ec_SC0632	2018	SRR17925356	PRJNA675916	Human	New Zealand	Campylobacter coli	8926	ST-828 complex	https://www.ncbi.nlm.nih.gov/sra/SRR17925356
Ec_SC0670	2018	SRR17925412	PRJNA675916	Chicken	New Zealand	Campylobacter coli	1581		https://www.ncbi.nlm.nih.gov/sra/SRR17925412
Ec_SC0674	2018	SRR17925407	PRJNA675916	Chicken	New Zealand	Campylobacter coli	890	ST-828 complex	https://www.ncbi.nlm.nih.gov/sra/SRR17925407
Ec_SC0677	2018	SRR17925404	PRJNA675916	Chicken	New Zealand	Campylobacter coli	8926	ST-828 complex	https://www.ncbi.nlm.nih.gov/sra/SRR17925404
Ec_SC0682	2018	SRR17925398	PRJNA675916	Human	New Zealand	Campylobacter coli	830	ST-828 complex	https://www.ncbi.nlm.nih.gov/sra/SRR17925398
Ec_SC0697	2018	SRR17925382	PRJNA675916	Chicken	New Zealand	Campylobacter coli	1581		https://www.ncbi.nlm.nih.gov/sra/SRR17925382
Ec_SC0725	2018	SRR17925256	PRJNA675916	Human	New Zealand	Campylobacter coli	nC07		https://www.ncbi.nlm.nih.gov/sra/SRR17925256
Ec_SC0737	2018	SRR17925243	PRJNA675916	Human	New Zealand	Campylobacter coli	825	ST-828 complex	https://www.ncbi.nlm.nih.gov/sra/SRR17925243
Ec_SC0742	2018	SRR17925238	PRJNA675916	Chicken	New Zealand	Campylobacter coli	8926	ST-828 complex	https://www.ncbi.nlm.nih.gov/sra/SRR17925238
Ec_SC0745	2018	SRR17925234	PRJNA675916	Human	New Zealand	Campylobacter coli	12072		https://www.ncbi.nlm.nih.gov/sra/SRR17925234
Ec_SC0760	2018	SRR17925314	PRJNA675916	Human	New Zealand	Campylobacter coli	854	ST-828 complex	https://www.ncbi.nlm.nih.gov/sra/SRR17925314
Ec_SC0761	2018	SRR17925313	PRJNA675916	Chicken	New Zealand	Campylobacter coli	8712	ST-828 complex	https://www.ncbi.nlm.nih.gov/sra/SRR17925313
Ec_SC0776	2018	SRR17925296	PRJNA675916	Human	New Zealand	Campylobacter coli	8926	ST-828 complex	https://www.ncbi.nlm.nih.gov/sra/SRR17925296
Ec_SC0840	2018	SRR17925138	PRJNA675916	Human	New Zealand	Campylobacter coli	1581		https://www.ncbi.nlm.nih.gov/sra/SRR17925138
Ec_SC0909	2018	SRR17929510	PRJNA675916	Human	New Zealand	Campylobacter coli	nC09		https://www.ncbi.nlm.nih.gov/sra/SRR17929510
Ec_SC0941	2018	SRR17929557	PRJNA675916	Chicken	New Zealand	Campylobacter coli	900		https://www.ncbi.nlm.nih.gov/sra/SRR17929557
Ec_SC0947	2018	SRR17929550	PRJNA675916	Chicken	New Zealand	Campylobacter coli	5734	ST-828 complex	https://www.ncbi.nlm.nih.gov/sra/SRR17929550
Ec_SC0948	2018	SRR17929549	PRJNA675916	Chicken	New Zealand	Campylobacter coli	2256	ST-828 complex	https://www.ncbi.nlm.nih.gov/sra/SRR17929549
Ec_SC0949	2018	SRR17929548	PRJNA675916	Chicken	New Zealand	Campylobacter coli	2256	ST-828 complex	https://www.ncbi.nlm.nih.gov/sra/SRR17929548
Ec_SC0987	2018	SRR17929518	PRJNA675916	Chicken	New Zealand	Campylobacter coli	900		https://www.ncbi.nlm.nih.gov/sra/SRR17929518
Ec_SC1010	2018	SRR17929595	PRJNA675916	Human	New Zealand	Campylobacter coli	nC09		https://www.ncbi.nlm.nih.gov/sra/SRR17929595
Ec_SC1073	2018	SRR17929610	PRJNA675916	Human	New Zealand	Campylobacter coli	8926	ST-828 complex	https://www.ncbi.nlm.nih.gov/sra/SRR17929610
Ec_SC1319	2019	SRR17965024	PRJNA675916	Chicken	New Zealand	Campylobacter coli	4009	ST-828 complex	https://www.ncbi.nlm.nih.gov/sra/SRR17965024
Ec_SC1325	2019	SRR17965018	PRJNA675916	Chicken	New Zealand	Campylobacter coli	1581		https://www.ncbi.nlm.nih.gov/sra/SRR17965018
Ec_SC1380	2019	SRR17964945	PRJNA675916	Human	New Zealand	Campylobacter coli	8926	ST-828 complex	https://www.ncbi.nlm.nih.gov/sra/SRR17964945
Ec_SC1512	2019	SRR17974180	PRJNA675916	Human	New Zealand	Campylobacter coli	1581		https://www.ncbi.nlm.nih.gov/sra/SRR17974180
Ec_SC1558	2019	SRR17974355	PRJNA675916	Human	New Zealand	Campylobacter coli	8926	ST-828 complex	https://www.ncbi.nlm.nih.gov/sra/SRR17974355
Ec_SC1694	Data missing	SRR17974146	PRJNA675916	Chicken	New Zealand	Campylobacter coli	2256	ST-828 complex	https://www.ncbi.nlm.nih.gov/sra/SRR17974146
Ec_SC1697	Data missing	SRR17974143	PRJNA675916	Chicken	New Zealand	Campylobacter coli	2256	ST-828 complex	https://www.ncbi.nlm.nih.gov/sra/SRR17974143
Ec_SC1703	Data missing	SRR17974135	PRJNA675916	Chicken	New Zealand	Campylobacter coli	854	ST-828 complex	https://www.ncbi.nlm.nih.gov/sra/SRR17974135
Ec_SC1705	Data missing	SRR17974133	PRJNA675916	Chicken	New Zealand	Campylobacter coli	2256	ST-828 complex	https://www.ncbi.nlm.nih.gov/sra/SRR17974133
Ec_SC1706	Data missing	SRR17974132	PRJNA675916	Chicken	New Zealand	Campylobacter coli	2256	ST-828 complex	https://www.ncbi.nlm.nih.gov/sra/SRR17974132
Ec_SC1707	Data missing	SRR17974131	PRJNA675916	Chicken	New Zealand	Campylobacter coli	2256	ST-828 complex	https://www.ncbi.nlm.nih.gov/sra/SRR17974131
Ec_SC1708	Data missing	SRR17974129	PRJNA675916	Chicken	New Zealand	Campylobacter coli	2256	ST-828 complex	https://www.ncbi.nlm.nih.gov/sra/SRR17974129
Ec_SC1716	Data missing	SRR17974217	PRJNA675916	Chicken	New Zealand	Campylobacter coli	2256	ST-828 complex	https://www.ncbi.nlm.nih.gov/sra/SRR17974217
Ec_SC1717	Data missing	SRR17974216	PRJNA675916	Chicken	New Zealand	Campylobacter coli	2256	ST-828 complex	https://www.ncbi.nlm.nih.gov/sra/SRR17974216
Ec_SC1718	Data missing	SRR17974214	PRJNA675916	Chicken	New Zealand	Campylobacter coli	3230	ST-828 complex	https://www.ncbi.nlm.nih.gov/sra/SRR17974214
Ec_SC1725	Data missing	SRR17974207	PRJNA675916	Chicken	New Zealand	Campylobacter coli	2256	ST-828 complex	https://www.ncbi.nlm.nih.gov/sra/SRR17974207
Ej_SC0016	2018	SRR17898870	PRJNA675916	Human	New Zealand	Campylobacter jejuni	45	ST-45 complex	https://www.ncbi.nlm.nih.gov/sra/SRR17898870
Ej_SC0017	2018	SRR17898869	PRJNA675916	Human	New Zealand	Campylobacter jejuni	508	ST-508 complex	https://www.ncbi.nlm.nih.gov/sra/SRR17898869
Ej_SC0018	2018	SRR17898868	PRJNA675916	Human	New Zealand	Campylobacter jejuni	45	ST-45 complex	https://www.ncbi.nlm.nih.gov/sra/SRR17898868

Isolate no.	Year	Accession no.	BioProject no.	Source	Country	Species	Sequence type	Clonal complex	SRA hyperlink
Ej_SC0019	2018	SRR17898867	PRJNA675916	Human	New Zealand	Campylobacter jejuni	45	ST-45 complex	https://www.ncbi.nlm.nih.gov/sra/SRR17898867
Ej_SC0024	2018	SRR17898900	PRJNA675916	Human	New Zealand	Campylobacter jejuni	474	ST-48 complex	https://www.ncbi.nlm.nih.gov/sra/SRR17898900
Ej_SC0026	2018	SRR17898898	PRJNA675916	Human	New Zealand	Campylobacter jejuni	6964	ST-354 complex	https://www.ncbi.nlm.nih.gov/sra/SRR17898898
Ej_SC0028	2018	SRR17898896	PRJNA675916	Human	New Zealand	Campylobacter jejuni	12749	ST-353 complex	https://www.ncbi.nlm.nih.gov/sra/SRR17898896
Ej_SC0031	2018	SRR17898892	PRJNA675916	Human	New Zealand	Campylobacter jejuni	227	ST-206 complex	https://www.ncbi.nlm.nih.gov/sra/SRR17898892
Ej_SC0037	2018	SRR17898886	PRJNA675916	Human	New Zealand	Campylobacter jejuni	354	ST-354 complex	https://www.ncbi.nlm.nih.gov/sra/SRR17898886
Ej_SC0038	2018	SRR17898885	PRJNA675916	Human	New Zealand	Campylobacter jejuni	6964	ST-354 complex	https://www.ncbi.nlm.nih.gov/sra/SRR17898885
Ej_SC0041	2018	SRR17898881	PRJNA675916	Human	New Zealand	Campylobacter jejuni	48	ST-48 complex	https://www.ncbi.nlm.nih.gov/sra/SRR17898881
Ej_SC0042	2018	SRR17898880	PRJNA675916	Human	New Zealand	Campylobacter jejuni	538	ST-45 complex	https://www.ncbi.nlm.nih.gov/sra/SRR17898880
Ej_SC0045	2018	SRR17898877	PRJNA675916	Human	New Zealand	Campylobacter jejuni	45	ST-45 complex	https://www.ncbi.nlm.nih.gov/sra/SRR17898877
Ej_SC0046	2018	SRR17898876	PRJNA675916	Human	New Zealand	Campylobacter jejuni	50	ST-21 complex	https://www.ncbi.nlm.nih.gov/sra/SRR17898876
Ej_SC0047	2018	SRR17898875	PRJNA675916	Human	New Zealand	Campylobacter jejuni	21	ST-21 complex	https://www.ncbi.nlm.nih.gov/sra/SRR17898875
Ej_SC0048	2018	SRR17898874	PRJNA675916	Human	New Zealand	Campylobacter jejuni	486	ST-21 complex	https://www.ncbi.nlm.nih.gov/sra/SRR17898874
Ej_SC0054	2018	SRR17926709	PRJNA675916	Human	New Zealand	Campylobacter jejuni	50	ST-21 complex	https://www.ncbi.nlm.nih.gov/sra/SRR17926709
Ej_SC0058	2018	SRR17926686	PRJNA675916	Human	New Zealand	Campylobacter jejuni	677	ST-677 complex	https://www.ncbi.nlm.nih.gov/sra/SRR17926686
Ej_SC0062	2018	SRR17926729	PRJNA675916	Human	New Zealand	Campylobacter jejuni	6964	ST-354 complex	https://www.ncbi.nlm.nih.gov/sra/SRR17926729
Ej_SC0064	2018	SRR17926727	PRJNA675916	Human	New Zealand	Campylobacter jejuni	50	ST-21 complex	https://www.ncbi.nlm.nih.gov/sra/SRR17926727
Ej_SC0066	2018	SRR17926725	PRJNA675916	Human	New Zealand	Campylobacter jejuni	2345	ST-206 complex	https://www.ncbi.nlm.nih.gov/sra/SRR17926725
Ej_SC0069	2018	SRR17926723	PRJNA675916	Human	New Zealand	Campylobacter jejuni	12752		https://www.ncbi.nlm.nih.gov/sra/SRR17926723
Ej_SC0072	2018	SRR17926719	PRJNA675916	Human	New Zealand	Campylobacter jejuni	53	ST-21 complex	https://www.ncbi.nlm.nih.gov/sra/SRR17926719
Ej_SC0074	2018	SRR17926717	PRJNA675916	Human	New Zealand	Campylobacter jejuni	474	ST-48 complex	https://www.ncbi.nlm.nih.gov/sra/SRR17926717
Ej_SC0078	2018	SRR17926713	PRJNA675916	Chicken	New Zealand	Campylobacter jejuni	53	ST-21 complex	https://www.ncbi.nlm.nih.gov/sra/SRR17926713
Ej_SC0079	2018	SRR17926712	PRJNA675916	Chicken	New Zealand	Campylobacter jejuni	3721	ST-354 complex	https://www.ncbi.nlm.nih.gov/sra/SRR17926712
Ej_SC0080	2018	SRR17926711	PRJNA675916	Chicken	New Zealand	Campylobacter jejuni	6964	ST-354 complex	https://www.ncbi.nlm.nih.gov/sra/SRR17926711
Ej_SC0081	2018	SRR17926710	PRJNA675916	Chicken	New Zealand	Campylobacter jejuni	6964	ST-354 complex	https://www.ncbi.nlm.nih.gov/sra/SRR17926710
Ej_SC0082	2018	SRR17926708	PRJNA675916	Chicken	New Zealand	Campylobacter jejuni	48	ST-48 complex	https://www.ncbi.nlm.nih.gov/sra/SRR17926708
Ej_SC0096	2018	SRR17926693	PRJNA675916	Human	New Zealand	Campylobacter jejuni	53	ST-21 complex	https://www.ncbi.nlm.nih.gov/sra/SRR17926693
Ej_SC0097	2018	SRR17926692	PRJNA675916	Human	New Zealand	Campylobacter jejuni	190	ST-21 complex	https://www.ncbi.nlm.nih.gov/sra/SRR17926692
Ej_SC0102	2018	SRR17899276	PRJNA675916	Human	New Zealand	Campylobacter jejuni	2347		https://www.ncbi.nlm.nih.gov/sra/SRR17899276
Ej_SC0103	2018	SRR17899233	PRJNA675916	Human	New Zealand	Campylobacter jejuni	61	ST-61 complex	https://www.ncbi.nlm.nih.gov/sra/SRR17899233
Ej_SC0105	2018	SRR17899211	PRJNA675916	Human	New Zealand	Campylobacter jejuni	190	ST-21 complex	https://www.ncbi.nlm.nih.gov/sra/SRR17899211
Ej_SC0107	2018	SRR17899253	PRJNA675916	Human	New Zealand	Campylobacter jejuni	3610	ST-21 complex	https://www.ncbi.nlm.nih.gov/sra/SRR17899253
Ej_SC0109	2018	SRR17899199	PRJNA675916	Chicken	New Zealand	Campylobacter jejuni	257	ST-257 complex	https://www.ncbi.nlm.nih.gov/sra/SRR17899199
Ej_SC0110	2018	SRR17899188	PRJNA675916	Chicken	New Zealand	Campylobacter jejuni	474	ST-48 complex	https://www.ncbi.nlm.nih.gov/sra/SRR17899188
Ej_SC0113	2018	SRR17899274	PRJNA675916	Chicken	New Zealand	Campylobacter jejuni	474	ST-48 complex	https://www.ncbi.nlm.nih.gov/sra/SRR17899274
Ej_SC0114	2018	SRR17899241	PRJNA675916	Chicken	New Zealand	Campylobacter jejuni	474	ST-48 complex	https://www.ncbi.nlm.nih.gov/sra/SRR17899241
Ej_SC0115	2018	SRR17899240	PRJNA675916	Chicken	New Zealand	Campylobacter jejuni	474	ST-48 complex	https://www.ncbi.nlm.nih.gov/sra/SRR17899240
Ej_SC0116	2018	SRR17899239	PRJNA675916	Chicken	New Zealand	Campylobacter jejuni	45	ST-45 complex	https://www.ncbi.nlm.nih.gov/sra/SRR17899239
Ej_SC0117	2018	SRR17899238	PRJNA675916	Chicken	New Zealand	Campylobacter jejuni	48	ST-48 complex	https://www.ncbi.nlm.nih.gov/sra/SRR17899238
Ej_SC0134	2018	SRR17899220	PRJNA675916	Human	New Zealand	Campylobacter jejuni	535	ST-460 complex	https://www.ncbi.nlm.nih.gov/sra/SRR17899220
Ej_SC0136	2018	SRR17899218	PRJNA675916	Human	New Zealand	Campylobacter jejuni	8185	ST-21 complex	https://www.ncbi.nlm.nih.gov/sra/SRR17899218
Ej_SC0137	2018	SRR17899217	PRJNA675916	Human	New Zealand	Campylobacter jejuni	48	ST-48 complex	https://www.ncbi.nlm.nih.gov/sra/SRR17899217
Ej_SC0138	2018	SRR17899216	PRJNA675916	Human	New Zealand	Campylobacter jejuni	48	ST-48 complex	https://www.ncbi.nlm.nih.gov/sra/SRR17899216
Ej_SC0139	2018	SRR17899215	PRJNA675916	Human	New Zealand	Campylobacter jejuni	2347		https://www.ncbi.nlm.nih.gov/sra/SRR17899215
Ej_SC0141	2018	SRR17899213	PRJNA675916	Human	New Zealand	Campylobacter jejuni	474	ST-48 complex	https://www.ncbi.nlm.nih.gov/sra/SRR17899213
Ej_SC0143	2018	SRR17899210	PRJNA675916	Human	New Zealand	Campylobacter jejuni	50	ST-21 complex	https://www.ncbi.nlm.nih.gov/sra/SRR17899210
Ej_SC0144	2018	SRR17899273	PRJNA675916	Human	New Zealand	Campylobacter jejuni	8065	ST-353 complex	https://www.ncbi.nlm.nih.gov/sra/SRR17899273
Ej_SC0145	2018	SRR17899272	PRJNA675916	Human	New Zealand	Campylobacter jejuni	257	ST-257 complex	https://www.ncbi.nlm.nih.gov/sra/SRR17899272
Ej_SC0147	2018	SRR17899270	PRJNA675916	Human	New Zealand	Campylobacter jejuni	257	ST-257 complex	https://www.ncbi.nlm.nih.gov/sra/SRR17899270
Ej_SC0149	2018	SRR17899268	PRJNA675916	Human	New Zealand	Campylobacter jejuni	486	ST-21 complex	https://www.ncbi.nlm.nih.gov/sra/SRR17899268
Ej_SC0153	2018	SRR17899263	PRJNA675916	Human	New Zealand	Campylobacter jejuni	3538		https://www.ncbi.nlm.nih.gov/sra/SRR17899263
Ej_SC0154	2018	SRR17899262	PRJNA675916	Human	New Zealand	Campylobacter jejuni	3538		https://www.ncbi.nlm.nih.gov/sra/SRR17899262
Ej_SC0155	2018	SRR17899261	PRJNA675916	Human	New Zealand	Campylobacter jejuni	474	ST-48 complex	https://www.ncbi.nlm.nih.gov/sra/SRR17899261
Ej_SC0159	2018	SRR17899257	PRJNA675916	Human	New Zealand	Campylobacter jejuni	48	ST-48 complex	https://www.ncbi.nlm.nih.gov/sra/SRR17899257
Ej_SC0160	2018	SRR17899256	PRJNA675916	Human	New Zealand	Campylobacter jejuni	6964	ST-354 complex	https://www.ncbi.nlm.nih.gov/sra/SRR17899256
Ej_SC0161	2018	SRR17899255	PRJNA675916	Chicken	New Zealand	Campylobacter jejuni	6964	ST-354 complex	https://www.ncbi.nlm.nih.gov/sra/SRR17899255
Ej_SC0162	2018	SRR17899254	PRJNA675916	Chicken	New Zealand	Campylobacter jejuni	2345	ST-206 complex	https://www.ncbi.nlm.nih.gov/sra/SRR17899254
Ej_SC0163	2018	SRR17899252	PRJNA675916	Chicken	New Zealand	Campylobacter jejuni	6964	ST-354 complex	https://www.ncbi.nlm.nih.gov/sra/SRR17899252
Ej_SC0164	2018	SRR17899251	PRJNA675916	Chicken	New Zealand	Campylobacter jejuni	45	ST-45 complex	https://www.ncbi.nlm.nih.gov/sra/SRR17899251
Ej_SC0165	2018	SRR17899250	PRJNA675916	Human	New Zealand	Campylobacter jejuni	190	ST-21 complex	https://www.ncbi.nlm.nih.gov/sra/SRR17899250
Ej_SC0166	2018	SRR17899249	PRJNA675916	Human	New Zealand	Campylobacter jejuni	474	ST-48 complex	https://www.ncbi.nlm.nih.gov/sra/SRR17899249
Ej_SC0167	2018	SRR17899248	PRJNA675916	Human	New Zealand	Campylobacter jejuni	50	ST-21 complex	https://www.ncbi.nlm.nih.gov/sra/SRR17899248
Ej_SC0168	2018	SRR17899247	PRJNA675916	Human	New Zealand	Campylobacter jejuni	520	ST-21 complex	https://www.ncbi.nlm.nih.gov/sra/SRR17899247
Ej_SC0169	2018	SRR17899246	PRJNA675916	Human	New Zealand	Campylobacter jejuni	436		https://www.ncbi.nlm.nih.gov/sra/SRR17899246
Ej_SC0170	2018	SRR17899245	PRJNA675916	Human	New Zealand	Campylobacter jejuni	6964	ST-354 complex	https://www.ncbi.nlm.nih.gov/sra/SRR17899245
Ej_SC0172	2018	SRR17899243	PRJNA675916	Human	New Zealand	Campylobacter jejuni	486	ST-21 complex	https://www.ncbi.nlm.nih.gov/sra/SRR17899243
Ej_SC0173	2018	SRR17899209	PRJNA675916	Human	New Zealand	Campylobacter jejuni	61	ST-61 complex	https://www.ncbi.nlm.nih.gov/sra/SRR17899209
Ej_SC0174	2018	SRR17899208	PRJNA675916	Human	New Zealand	Campylobacter jejuni	53	ST-21 complex	https://www.ncbi.nlm.nih.gov/sra/SRR17899208
Ej_SC0180	2018	SRR17899202	PRJNA675916	Human	New Zealand	Campylobacter jejuni	61	ST-61 complex	https://www.ncbi.nlm.nih.gov/sra/SRR17899202
Ej_SC0181	2018	SRR17899201	PRJNA675916	Human	New Zealand	Campylobacter jejuni	53	ST-21 complex	https://www.ncbi.nlm.nih.gov/sra/SRR17899201
Ej_SC0182	2018	SRR17899200	PRJNA675916	Human	New Zealand	Campylobacter jejuni	474	ST-48 complex	https://www.ncbi.nlm.nih.gov/sra/SRR17899200
Ej_SC0183	2018	SRR17899198	PRJNA675916	Human	New Zealand	Campylobacter jejuni	190	ST-21 complex	https://www.ncbi.nlm.nih.gov/sra/SRR17899198
Ej_SC0184	2018	SRR17899197	PRJNA675916	Chicken	New Zealand	Campylobacter jejuni	50	ST-21 complex	https://www.ncbi.nlm.nih.gov/sra/SRR17899197
Ej_SC0185	2018	SRR17899196	PRJNA675916	Chicken	New Zealand	Campylobacter jejuni	658	ST-658 complex	https://www.ncbi.nlm.nih.gov/sra/SRR17899196
Ej_SC0186	2018	SRR17899195	PRJNA675916	Chicken	New Zealand	Campylobacter jejuni	6964	ST-354 complex	https://www.ncbi.nlm.nih.gov/sra/SRR17899195
Ej_SC0188	2018	SRR17899193	PRJNA675916	Chicken	New Zealand	Campylobacter jejuni	6964	ST-354 complex	https://www.ncbi.nlm.nih.gov/sra/SRR17899193
Ej_SC0189	2018	SRR17899192	PRJNA675916	Chicken	New Zealand	Campylobacter jejuni	6964	ST-354 complex	https://www.ncbi.nlm.nih.gov/sra/SRR17899192
Ej_SC0190	2018	SRR17899191	PRJNA675916	Human	New Zealand	Campylobacter jejuni	474	ST-48 complex	https://www.ncbi.nlm.nih.gov/sra/SRR17899191
Ej_SC0191	2018	SRR17899190	PRJNA675916	Human	New Zealand	Campylobacter jejuni	50	ST-21 complex	https://www.ncbi.nlm.nih.gov/sra/SRR17899190
Ej_SC0195	2018	SRR17899185	PRJNA675916	Human	New Zealand	Campylobacter jejuni	422	ST-21 complex	https://www.ncbi.nlm.nih.gov/sra/SRR17899185
Ej_SC0196	2018	SRR17899184	PRJNA675916	Human	New Zealand	Campylobacter jejuni	53	ST-21 complex	https://www.ncbi.nlm.nih.gov/sra/SRR17899184
Ej_SC0214	2018	SRR17900487	PRJNA675916	Human	New Zealand	Campylobacter jejuni	2026	ST-403 complex	https://www.ncbi.nlm.nih.gov/sra/SRR17900487
Ej_SC0217	2018	SRR17900454	PRJNA675916	Human	New Zealand	Campylobacter jejuni	520	ST-21 complex	https://www.ncbi.nlm.nih.gov/sra/SRR17900454
Ej_SC0218	2018	SRR17900443	PRJNA675916	Human	New Zealand	Campylobacter jejuni	2345	ST-206 complex	https://www.ncbi.nlm.nih.gov/sra/SRR17900443
Ej_SC0219	2018	SRR17900432	PRJNA675916	Human	New Zealand	Campylobacter jejuni	38	ST-48 complex	https://www.ncbi.nlm.nih.gov/sra/SRR17900432
Ej_SC0220	2018	SRR17900357	PRJNA675916	Human	New Zealand	Campylobacter jejuni	520	ST-21 complex	https://www.ncbi.nlm.nih.gov/sra/SRR17900357
Ej_SC0222	2018	SRR17900334	PRJNA675916	Human	New Zealand	Campylobacter jejuni	520	ST-21 complex	https://www.ncbi.nlm.nih.gov/sra/SRR17900334

Isolate no.	Year	Accession no.	BioProject no.	Source	Country	Species	Sequence type	Clonal complex	SRA hyperlink
Ej_SC0223	2018	SRR17900419	PRJNA675916	Chicken	New Zealand	Campylobacter jejuni	50	ST-21 complex	https://www.ncbi.nlm.nih.gov/sra/SRR17900419
Ej_SC0224	2018	SRR17900408	PRJNA675916	Chicken	New Zealand	Campylobacter jejuni	45	ST-45 complex	https://www.ncbi.nlm.nih.gov/sra/SRR17900408
Ej_SC0225	2018	SRR17900397	PRJNA675916	Chicken	New Zealand	Campylobacter jejuni	48	ST-48 complex	https://www.ncbi.nlm.nih.gov/sra/SRR17900397
Ej_SC0226	2018	SRR17900578	PRJNA675916	Chicken	New Zealand	Campylobacter jejuni	190	ST-21 complex	https://www.ncbi.nlm.nih.gov/sra/SRR17900578
Ej_SC0227	2018	SRR17900567	PRJNA675916	Chicken	New Zealand	Campylobacter jejuni	190	ST-21 complex	https://www.ncbi.nlm.nih.gov/sra/SRR17900567
Ej_SC0228	2018	SRR17900556	PRJNA675916	Chicken	New Zealand	Campylobacter jejuni	6964	ST-354 complex	https://www.ncbi.nlm.nih.gov/sra/SRR17900556
Ej_SC0230	2018	SRR17900374	PRJNA675916	Human	New Zealand	Campylobacter jejuni	50	ST-21 complex	https://www.ncbi.nlm.nih.gov/sra/SRR17900374
Ej_SC0235	2018	SRR17900553	PRJNA675916	Human	New Zealand	Campylobacter jejuni	2081		https://www.ncbi.nlm.nih.gov/sra/SRR17900553
Ej_SC0236	2018	SRR17900552	PRJNA675916	Human	New Zealand	Campylobacter jejuni	45	ST-45 complex	https://www.ncbi.nlm.nih.gov/sra/SRR17900552
Ej_SC0237	2018	SRR17900551	PRJNA675916	Human	New Zealand	Campylobacter jejuni	2392	ST-52 complex	https://www.ncbi.nlm.nih.gov/sra/SRR17900551
Ej_SC0238	2018	SRR17900550	PRJNA675916	Human	New Zealand	Campylobacter jejuni	520	ST-21 complex	https://www.ncbi.nlm.nih.gov/sra/SRR17900550
Ej_SC0240	2018	SRR17900548	PRJNA675916	Human	New Zealand	Campylobacter jejuni	42	ST-42 complex	https://www.ncbi.nlm.nih.gov/sra/SRR17900548
Ej_SC0241	2018	SRR17900546	PRJNA675916	Human	New Zealand	Campylobacter jejuni	50	ST-21 complex	https://www.ncbi.nlm.nih.gov/sra/SRR17900546
Ej_SC0259	2018	SRR17900528	PRJNA675916	Human	New Zealand	Campylobacter jejuni	583	ST-45 complex	https://www.ncbi.nlm.nih.gov/sra/SRR17900528
Ej_SC0260	2018	SRR17900527	PRJNA675916	Human	New Zealand	Campylobacter jejuni	6964	ST-354 complex	https://www.ncbi.nlm.nih.gov/sra/SRR17900527
Ej_SC0261	2018	SRR17900526	PRJNA675916	Human	New Zealand	Campylobacter jejuni	530		https://www.ncbi.nlm.nih.gov/sra/SRR17900526
Ej_SC0266	2018	SRR17900328	PRJNA675916	Human	New Zealand	Campylobacter jejuni	38	ST-48 complex	https://www.ncbi.nlm.nih.gov/sra/SRR17900328
Ej_SC0267	2018	SRR17900327	PRJNA675916	Human	New Zealand	Campylobacter jejuni	486	ST-21 complex	https://www.ncbi.nlm.nih.gov/sra/SRR17900327
Ej_SC0268	2018	SRR17900326	PRJNA675916	Human	New Zealand	Campylobacter jejuni	474	ST-48 complex	https://www.ncbi.nlm.nih.gov/sra/SRR17900326
Ej_SC0270	2018	SRR17900324	PRJNA675916	Chicken	New Zealand	Campylobacter jejuni	6964	ST-354 complex	https://www.ncbi.nlm.nih.gov/sra/SRR17900324
Ej_SC0271	2018	SRR17900323	PRJNA675916	Human	New Zealand	Campylobacter jejuni	50	ST-21 complex	https://www.ncbi.nlm.nih.gov/sra/SRR17900323
Ej_SC0272	2018	SRR17900321	PRJNA675916	Human	New Zealand	Campylobacter jejuni	6964	ST-354 complex	https://www.ncbi.nlm.nih.gov/sra/SRR17900321
Ej_SC0273	2018	SRR17900320	PRJNA675916	Human	New Zealand	Campylobacter jejuni	508	ST-508 complex	https://www.ncbi.nlm.nih.gov/sra/SRR17900320
Ej_SC0275	2018	SRR17900318	PRJNA675916	Human	New Zealand	Campylobacter jejuni	227	ST-206 complex	https://www.ncbi.nlm.nih.gov/sra/SRR17900318
Ej_SC0278	2018	SRR17900315	PRJNA675916	Human	New Zealand	Campylobacter jejuni	474	ST-48 complex	https://www.ncbi.nlm.nih.gov/sra/SRR17900315
Ej_SC0279	2018	SRR17900314	PRJNA675916	Human	New Zealand	Campylobacter jejuni	508	ST-508 complex	https://www.ncbi.nlm.nih.gov/sra/SRR17900314
Ej_SC0282	2018	SRR17900310	PRJNA675916	Human	New Zealand	Campylobacter jejuni	474	ST-48 complex	https://www.ncbi.nlm.nih.gov/sra/SRR17900310
Ej_SC0283	2018	SRR17900309	PRJNA675916	Human	New Zealand	Campylobacter jejuni	45	ST-45 complex	https://www.ncbi.nlm.nih.gov/sra/SRR17900309
Ej_SC0284	2018	SRR17900308	PRJNA675916	Human	New Zealand	Campylobacter jejuni	50	ST-21 complex	https://www.ncbi.nlm.nih.gov/sra/SRR17900308
Ej_SC0285	2018	SRR17900307	PRJNA675916	Human	New Zealand	Campylobacter jejuni	61	ST-61 complex	https://www.ncbi.nlm.nih.gov/sra/SRR17900307
Ej_SC0287	2018	SRR17900305	PRJNA675916	Chicken	New Zealand	Campylobacter jejuni	4337	ST-21 complex	https://www.ncbi.nlm.nih.gov/sra/SRR17900305
Ej_SC0288	2018	SRR17900304	PRJNA675916	Chicken	New Zealand	Campylobacter jejuni	45	ST-45 complex	https://www.ncbi.nlm.nih.gov/sra/SRR17900304
Ej_SC0289	2018	SRR17900303	PRJNA675916	Chicken	New Zealand	Campylobacter jejuni	6964	ST-354 complex	https://www.ncbi.nlm.nih.gov/sra/SRR17900303
Ej_SC0293	2018	SRR17900298	PRJNA675916	Human	New Zealand	Campylobacter jejuni	50	ST-21 complex	https://www.ncbi.nlm.nih.gov/sra/SRR17900298
Ej_SC0295	2018	SRR17900296	PRJNA675916	Human	New Zealand	Campylobacter jejuni	486	ST-21 complex	https://www.ncbi.nlm.nih.gov/sra/SRR17900296
Ej_SC0296	2018	SRR17900295	PRJNA675916	Human	New Zealand	Campylobacter jejuni	48	ST-48 complex	https://www.ncbi.nlm.nih.gov/sra/SRR17900295
Ej_SC0297	2018	SRR17900294	PRJNA675916	Human	New Zealand	Campylobacter jejuni	6964	ST-354 complex	https://www.ncbi.nlm.nih.gov/sra/SRR17900294
Ej_SC0298	2018	SRR17900293	PRJNA675916	Human	New Zealand	Campylobacter jejuni	190	ST-21 complex	https://www.ncbi.nlm.nih.gov/sra/SRR17900293
Ej_SC0299	2018	SRR17900292	PRJNA675916	Human	New Zealand	Campylobacter jejuni	61	ST-61 complex	https://www.ncbi.nlm.nih.gov/sra/SRR17900292
Ej_SC0303	2018	SRR17900518	PRJNA675916	Human	New Zealand	Campylobacter jejuni	50	ST-21 complex	https://www.ncbi.nlm.nih.gov/sra/SRR17900518
Ej_SC0305	2018	SRR17900516	PRJNA675916	Human	New Zealand	Campylobacter jejuni	1517	ST-354 complex	https://www.ncbi.nlm.nih.gov/sra/SRR17900516
Ej_SC0306	2018	SRR17900515	PRJNA675916	Chicken	New Zealand	Campylobacter jejuni	5655	ST-21 complex	https://www.ncbi.nlm.nih.gov/sra/SRR17900515
Ej_SC0307	2018	SRR17900514	PRJNA675916	Chicken	New Zealand	Campylobacter jejuni	45	ST-45 complex	https://www.ncbi.nlm.nih.gov/sra/SRR17900514
Ej_SC0308	2018	SRR17900513	PRJNA675916	Chicken	New Zealand	Campylobacter jejuni	6964	ST-354 complex	https://www.ncbi.nlm.nih.gov/sra/SRR17900513
Ej_SC0309	2018	SRR17900512	PRJNA675916	Chicken	New Zealand	Campylobacter jejuni	48	ST-48 complex	https://www.ncbi.nlm.nih.gov/sra/SRR17900512
Ej_SC0310	2018	SRR17900511	PRJNA675916	Chicken	New Zealand	Campylobacter jejuni	45	ST-45 complex	https://www.ncbi.nlm.nih.gov/sra/SRR17900511
Ej_SC0311	2018	SRR17900510	PRJNA675916	Chicken	New Zealand	Campylobacter jejuni	4337	ST-21 complex	https://www.ncbi.nlm.nih.gov/sra/SRR17900510
Ej_SC0312	2018	SRR17900508	PRJNA675916	Chicken	New Zealand	Campylobacter jejuni	486	ST-21 complex	https://www.ncbi.nlm.nih.gov/sra/SRR17900508
Ej_SC0314	2018	SRR17900506	PRJNA675916	Human	New Zealand	Campylobacter jejuni	6964	ST-354 complex	https://www.ncbi.nlm.nih.gov/sra/SRR17900506
Ej_SC0316	2018	SRR17900504	PRJNA675916	Human	New Zealand	Campylobacter jejuni	25	ST-45 complex	https://www.ncbi.nlm.nih.gov/sra/SRR17900504
Ej_SC0317	2018	SRR17900503	PRJNA675916	Human	New Zealand	Campylobacter jejuni	474	ST-48 complex	https://www.ncbi.nlm.nih.gov/sra/SRR17900503
Ej_SC0319	2018	SRR17900501	PRJNA675916	Human	New Zealand	Campylobacter jejuni	227	ST-206 complex	https://www.ncbi.nlm.nih.gov/sra/SRR17900501
Ej_SC0320	2018	SRR17900500	PRJNA675916	Human	New Zealand	Campylobacter jejuni	227	ST-206 complex	https://www.ncbi.nlm.nih.gov/sra/SRR17900500
Ej_SC0321	2018	SRR17900499	PRJNA675916	Human	New Zealand	Campylobacter jejuni	42	ST-42 complex	https://www.ncbi.nlm.nih.gov/sra/SRR17900499
Ej_SC0323	2018	SRR17900496	PRJNA675916	Human	New Zealand	Campylobacter jejuni	6964	ST-354 complex	https://www.ncbi.nlm.nih.gov/sra/SRR17900496
Ej_SC0324	2018	SRR17900495	PRJNA675916	Human	New Zealand	Campylobacter jejuni	2350	ST-48 complex	https://www.ncbi.nlm.nih.gov/sra/SRR17900495
Ej_SC0349	2018	SRR17900468	PRJNA675916	Chicken	New Zealand	Campylobacter jejuni	6964	ST-354 complex	https://www.ncbi.nlm.nih.gov/sra/SRR17900468
Ej_SC0350	2018	SRR17900467	PRJNA675916	Chicken	New Zealand	Campylobacter jejuni	6964	ST-354 complex	https://www.ncbi.nlm.nih.gov/sra/SRR17900467
Ej_SC0351	2018	SRR17900466	PRJNA675916	Chicken	New Zealand	Campylobacter jejuni	583	ST-45 complex	https://www.ncbi.nlm.nih.gov/sra/SRR17900466
Ej_SC0353	2018	SRR17900463	PRJNA675916	Human	New Zealand	Campylobacter jejuni	508	ST-508 complex	https://www.ncbi.nlm.nih.gov/sra/SRR17900463
Ej_SC0354	2018	SRR17900462	PRJNA675916	Human	New Zealand	Campylobacter jejuni	22	ST-22 complex	https://www.ncbi.nlm.nih.gov/sra/SRR17900462
Ej_SC0356	2018	SRR17900460	PRJNA675916	Human	New Zealand	Campylobacter jejuni	227	ST-206 complex	https://www.ncbi.nlm.nih.gov/sra/SRR17900460
Ej_SC0357	2018	SRR17900459	PRJNA675916	Human	New Zealand	Campylobacter jejuni	22	ST-22 complex	https://www.ncbi.nlm.nih.gov/sra/SRR17900459
Ej_SC0358	2018	SRR17900458	PRJNA675916	Human	New Zealand	Campylobacter jejuni	6964	ST-354 complex	https://www.ncbi.nlm.nih.gov/sra/SRR17900458
Ej_SC0359	2018	SRR17900457	PRJNA675916	Human	New Zealand	Campylobacter jejuni	6964	ST-354 complex	https://www.ncbi.nlm.nih.gov/sra/SRR17900457
Ej_SC0360	2018	SRR17900456	PRJNA675916	Human	New Zealand	Campylobacter jejuni	48	ST-48 complex	https://www.ncbi.nlm.nih.gov/sra/SRR17900456
Ej_SC0362	2018	SRR17900453	PRJNA675916	Human	New Zealand	Campylobacter jejuni	50	ST-21 complex	https://www.ncbi.nlm.nih.gov/sra/SRR17900453
Ej_SC0363	2018	SRR17900452	PRJNA675916	Human	New Zealand	Campylobacter jejuni	45	ST-45 complex	https://www.ncbi.nlm.nih.gov/sra/SRR17900452
Ej_SC0364	2018	SRR17900451	PRJNA675916	Human	New Zealand	Campylobacter jejuni	227	ST-206 complex	https://www.ncbi.nlm.nih.gov/sra/SRR17900451
Ej_SC0366	2018	SRR17900449	PRJNA675916	Human	New Zealand	Campylobacter jejuni	2026	ST-403 complex	https://www.ncbi.nlm.nih.gov/sra/SRR17900449
Ej_SC0367	2018	SRR17900448	PRJNA675916	Human	New Zealand	Campylobacter jejuni	3676	ST-42 complex	https://www.ncbi.nlm.nih.gov/sra/SRR17900448
Ej_SC0368	2018	SRR17900447	PRJNA675916	Human	New Zealand	Campylobacter jejuni	53	ST-21 complex	https://www.ncbi.nlm.nih.gov/sra/SRR17900447
Ej_SC0369	2018	SRR17900446	PRJNA675916	Human	New Zealand	Campylobacter jejuni	50	ST-21 complex	https://www.ncbi.nlm.nih.gov/sra/SRR17900446
Ej_SC0370	2018	SRR17900445	PRJNA675916	Human	New Zealand	Campylobacter jejuni	6964	ST-354 complex	https://www.ncbi.nlm.nih.gov/sra/SRR17900445
Ej_SC0371	2018	SRR17900444	PRJNA675916	Human	New Zealand	Campylobacter jejuni	4337	ST-21 complex	https://www.ncbi.nlm.nih.gov/sra/SRR17900444
Ej_SC0389	2018	SRR17900360	PRJNA675916	Chicken	New Zealand	Campylobacter jejuni	6964	ST-354 complex	https://www.ncbi.nlm.nih.gov/sra/SRR17900360
Ej_SC0392	2018	SRR17900358	PRJNA675916	Human	New Zealand	Campylobacter jejuni	48	ST-48 complex	https://www.ncbi.nlm.nih.gov/sra/SRR17900358
Ej_SC0393	2018	SRR17900356	PRJNA675916	Human	New Zealand	Campylobacter jejuni	45	ST-45 complex	https://www.ncbi.nlm.nih.gov/sra/SRR17900356
Ej_SC0394	2018	SRR17900355	PRJNA675916	Human	New Zealand	Campylobacter jejuni	53	ST-21 complex	https://www.ncbi.nlm.nih.gov/sra/SRR17900355
Ej_SC0397	2018	SRR17900352	PRJNA675916	Human	New Zealand	Campylobacter jejuni	50	ST-21 complex	https://www.ncbi.nlm.nih.gov/sra/SRR17900352
Ej_SC0398	2018	SRR17900351	PRJNA675916	Human	New Zealand	Campylobacter jejuni	2026	ST-403 complex	https://www.ncbi.nlm.nih.gov/sra/SRR17900351
Ej_SC0399	2018	SRR17900350	PRJNA675916	Human	New Zealand	Campylobacter jejuni	61	ST-61 complex	https://www.ncbi.nlm.nih.gov/sra/SRR17900350
Ej_SC0400	2018	SRR17900349	PRJNA675916	Human	New Zealand	Campylobacter jejuni	50	ST-21 complex	https://www.ncbi.nlm.nih.gov/sra/SRR17900349
Ej_SC0401	2018	SRR17900348	PRJNA675916	Human	New Zealand	Campylobacter jejuni	227	ST-206 complex	https://www.ncbi.nlm.nih.gov/sra/SRR17900348
Ej_SC0402	2018	SRR17900347	PRJNA675916	Human	New Zealand	Campylobacter jejuni	618	ST-61 complex	https://www.ncbi.nlm.nih.gov/sra/SRR17900347
Ej_SC0403	2018	SRR17900344	PRJNA675916	Human	New Zealand	Campylobacter jejuni	61	ST-61 complex	https://www.ncbi.nlm.nih.gov/sra/SRR17900344
Ej_SC0404	2018	SRR17900343	PRJNA675916	Human	New Zealand	Campylobacter jejuni	422	ST-21 complex	https://www.ncbi.nlm.nih.gov/sra/SRR17900343

Isolate no.	Year	Accession no.	BioProject no.	Source	Country	Species	Sequence type	Clonal complex	SRA hyperlink
Ej_SC0405	2018	SRR17900342	PRJNA675916	Chicken	New Zealand	Campylobacter jejuni	48	ST-48 complex	https://www.ncbi.nlm.nih.gov/sra/SRR17900342
Ej_SC0406	2018	SRR17900341	PRJNA675916	Chicken	New Zealand	Campylobacter jejuni	696	ST-1332 complex	https://www.ncbi.nlm.nih.gov/sra/SRR17900341
Ej_SC0407	2018	SRR17900340	PRJNA675916	Chicken	New Zealand	Campylobacter jejuni	190	ST-21 complex	https://www.ncbi.nlm.nih.gov/sra/SRR17900340
Ej_SC0411	2018	SRR17900336	PRJNA675916	Human	New Zealand	Campylobacter jejuni	48	ST-48 complex	https://www.ncbi.nlm.nih.gov/sra/SRR17900336
Ej_SC0413	2018	SRR17900333	PRJNA675916	Human	New Zealand	Campylobacter jejuni	5655	ST-21 complex	https://www.ncbi.nlm.nih.gov/sra/SRR17900333
Ej_SC0415	2018	SRR17900331	PRJNA675916	Human	New Zealand	Campylobacter jejuni	4337	ST-21 complex	https://www.ncbi.nlm.nih.gov/sra/SRR17900331
Ej_SC0417	2018	SRR17900425	PRJNA675916	Human	New Zealand	Campylobacter jejuni	48	ST-48 complex	https://www.ncbi.nlm.nih.gov/sra/SRR17900425
Ej_SC0420	2018	SRR17900422	PRJNA675916	Human	New Zealand	Campylobacter jejuni	6964	ST-354 complex	https://www.ncbi.nlm.nih.gov/sra/SRR17900422
Ej_SC0421	2018	SRR17900421	PRJNA675916	Human	New Zealand	Campylobacter jejuni	48	ST-48 complex	https://www.ncbi.nlm.nih.gov/sra/SRR17900421
Ej_SC0423	2018	SRR17900418	PRJNA675916	Human	New Zealand	Campylobacter jejuni	53	ST-21 complex	https://www.ncbi.nlm.nih.gov/sra/SRR17900418
Ej_SC0424	2018	SRR17900417	PRJNA675916	Human	New Zealand	Campylobacter jejuni	42	ST-42 complex	https://www.ncbi.nlm.nih.gov/sra/SRR17900417
Ej_SC0425	2018	SRR17900416	PRJNA675916	Human	New Zealand	Campylobacter jejuni	190	ST-21 complex	https://www.ncbi.nlm.nih.gov/sra/SRR17900416
Ej_SC0426	2018	SRR17900415	PRJNA675916	Human	New Zealand	Campylobacter jejuni	474	ST-48 complex	https://www.ncbi.nlm.nih.gov/sra/SRR17900415
Ej_SC0427	2018	SRR17900414	PRJNA675916	Human	New Zealand	Campylobacter jejuni	2535		https://www.ncbi.nlm.nih.gov/sra/SRR17900414
Ej_SC0428	2018	SRR17900413	PRJNA675916	Human	New Zealand	Campylobacter jejuni	474	ST-48 complex	https://www.ncbi.nlm.nih.gov/sra/SRR17900413
Ej_SC0431	2018	SRR17900410	PRJNA675916	Human	New Zealand	Campylobacter jejuni	50	ST-21 complex	https://www.ncbi.nlm.nih.gov/sra/SRR17900410
Ej_SC0432	2018	SRR17900409	PRJNA675916	Human	New Zealand	Campylobacter jejuni	1517	ST-354 complex	https://www.ncbi.nlm.nih.gov/sra/SRR17900409
Ej_SC0433	2018	SRR17900407	PRJNA675916	Chicken	New Zealand	Campylobacter jejuni	53	ST-21 complex	https://www.ncbi.nlm.nih.gov/sra/SRR17900407
Ej_SC0434	2018	SRR17900406	PRJNA675916	Chicken	New Zealand	Campylobacter jejuni	48	ST-48 complex	https://www.ncbi.nlm.nih.gov/sra/SRR17900406
Ej_SC0445	2018	SRR17900586	PRJNA675916	Human	New Zealand	Campylobacter jejuni	48	ST-48 complex	https://www.ncbi.nlm.nih.gov/sra/SRR17900586
Ej_SC0449	2018	SRR17900582	PRJNA675916	Human	New Zealand	Campylobacter jejuni	50	ST-21 complex	https://www.ncbi.nlm.nih.gov/sra/SRR17900582
Ej_SC0450	2018	SRR17900581	PRJNA675916	Human	New Zealand	Campylobacter jejuni	508	ST-508 complex	https://www.ncbi.nlm.nih.gov/sra/SRR17900581
Ej_SC0451	2018	SRR17900580	PRJNA675916	Human	New Zealand	Campylobacter jejuni	227	ST-206 complex	https://www.ncbi.nlm.nih.gov/sra/SRR17900580
Ej_SC0452	2018	SRR17900579	PRJNA675916	Chicken	New Zealand	Campylobacter jejuni	50	ST-21 complex	https://www.ncbi.nlm.nih.gov/sra/SRR17900579
Ej_SC0455	2018	SRR17900575	PRJNA675916	Chicken	New Zealand	Campylobacter jejuni	50	ST-21 complex	https://www.ncbi.nlm.nih.gov/sra/SRR17900575
Ej_SC0461	2018	SRR17900569	PRJNA675916	Human	New Zealand	Campylobacter jejuni	6964	ST-354 complex	https://www.ncbi.nlm.nih.gov/sra/SRR17900569
Ej_SC0462	2018	SRR17900568	PRJNA675916	Human	New Zealand	Campylobacter jejuni	50	ST-21 complex	https://www.ncbi.nlm.nih.gov/sra/SRR17900568
Ej_SC0464	2018	SRR17900565	PRJNA675916	Human	New Zealand	Campylobacter jejuni	538	ST-45 complex	https://www.ncbi.nlm.nih.gov/sra/SRR17900565
Ej_SC0465	2018	SRR17900564	PRJNA675916	Human	New Zealand	Campylobacter jejuni	257	ST-257 complex	https://www.ncbi.nlm.nih.gov/sra/SRR17900564
Ej_SC0467	2018	SRR17900562	PRJNA675916	Human	New Zealand	Campylobacter jejuni	48	ST-48 complex	https://www.ncbi.nlm.nih.gov/sra/SRR17900562
Ej_SC0469	2018	SRR17900560	PRJNA675916	Human	New Zealand	Campylobacter jejuni	53	ST-21 complex	https://www.ncbi.nlm.nih.gov/sra/SRR17900560
Ej_SC0470	2018	SRR17900559	PRJNA675916	Human	New Zealand	Campylobacter jejuni	50	ST-21 complex	https://www.ncbi.nlm.nih.gov/sra/SRR17900559
Ej_SC0472	2018	SRR17900557	PRJNA675916	Chicken	New Zealand	Campylobacter jejuni	6964	ST-354 complex	https://www.ncbi.nlm.nih.gov/sra/SRR17900557
Ej_SC0473	2018	SRR17900555	PRJNA675916	Chicken	New Zealand	Campylobacter jejuni	48	ST-48 complex	https://www.ncbi.nlm.nih.gov/sra/SRR17900555
Ej_SC0474	2018	SRR17900394	PRJNA675916	Chicken	New Zealand	Campylobacter jejuni	422	ST-21 complex	https://www.ncbi.nlm.nih.gov/sra/SRR17900394
Ej_SC0487	2018	SRR17900380	PRJNA675916	Human	New Zealand	Campylobacter jejuni	474	ST-48 complex	https://www.ncbi.nlm.nih.gov/sra/SRR17900380
Ej_SC0488	2018	SRR17900379	PRJNA675916	Human	New Zealand	Campylobacter jejuni	48	ST-48 complex	https://www.ncbi.nlm.nih.gov/sra/SRR17900379
Ej_SC0490	2018	SRR17900378	PRJNA675916	Human	New Zealand	Campylobacter jejuni	190	ST-21 complex	https://www.ncbi.nlm.nih.gov/sra/SRR17900378
Ej_SC0491	2018	SRR17900377	PRJNA675916	Human	New Zealand	Campylobacter jejuni	48	ST-48 complex	https://www.ncbi.nlm.nih.gov/sra/SRR17900377
Ej_SC0492	2018	SRR17900376	PRJNA675916	Human	New Zealand	Campylobacter jejuni	45	ST-45 complex	https://www.ncbi.nlm.nih.gov/sra/SRR17900376
Ej_SC0495	2018	SRR17900372	PRJNA675916	Human	New Zealand	Campylobacter jejuni	6964	ST-354 complex	https://www.ncbi.nlm.nih.gov/sra/SRR17900372
Ej_SC0498	2018	SRR17900369	PRJNA675916	Chicken	New Zealand	Campylobacter jejuni	6964	ST-354 complex	https://www.ncbi.nlm.nih.gov/sra/SRR17900369
Ej_SC0499	2018	SRR17900368	PRJNA675916	Chicken	New Zealand	Campylobacter jejuni	6964	ST-354 complex	https://www.ncbi.nlm.nih.gov/sra/SRR17900368
Ej_SC0500	2018	SRR17900367	PRJNA675916	Chicken	New Zealand	Campylobacter jejuni	6964	ST-354 complex	https://www.ncbi.nlm.nih.gov/sra/SRR17900367
Ej_SC0501	2018	SRR17925489	PRJNA675916	Chicken	New Zealand	Campylobacter jejuni	6964	ST-354 complex	https://www.ncbi.nlm.nih.gov/sra/SRR17925489
Ej_SC0502	2018	SRR17925488	PRJNA675916	Human	New Zealand	Campylobacter jejuni	1956	ST-1034 complex	https://www.ncbi.nlm.nih.gov/sra/SRR17925488
Ej_SC0503	2018	SRR17925377	PRJNA675916	Chicken	New Zealand	Campylobacter jejuni	6964	ST-354 complex	https://www.ncbi.nlm.nih.gov/sra/SRR17925377
Ej_SC0508	2018	SRR17925176	PRJNA675916	Human	New Zealand	Campylobacter jejuni	3711	ST-257 complex	https://www.ncbi.nlm.nih.gov/sra/SRR17925176
Ej_SC0514	2018	SRR17925358	PRJNA675916	Human	New Zealand	Campylobacter jejuni	6964	ST-354 complex	https://www.ncbi.nlm.nih.gov/sra/SRR17925358
Ej_SC0515	2018	SRR17925347	PRJNA675916	Chicken	New Zealand	Campylobacter jejuni	6964	ST-354 complex	https://www.ncbi.nlm.nih.gov/sra/SRR17925347
Ej_SC0516	2018	SRR17925336	PRJNA675916	Chicken	New Zealand	Campylobacter jejuni	50	ST-21 complex	https://www.ncbi.nlm.nih.gov/sra/SRR17925336
Ej_SC0517	2018	SRR17925421	PRJNA675916	Chicken	New Zealand	Campylobacter jejuni	583	ST-45 complex	https://www.ncbi.nlm.nih.gov/sra/SRR17925421
Ej_SC0518	2018	SRR17925410	PRJNA675916	Chicken	New Zealand	Campylobacter jejuni	4500		https://www.ncbi.nlm.nih.gov/sra/SRR17925410
Ej_SC0519	2018	SRR17925399	PRJNA675916	Chicken	New Zealand	Campylobacter jejuni	6964	ST-354 complex	https://www.ncbi.nlm.nih.gov/sra/SRR17925399
Ej_SC0520	2018	SRR17925388	PRJNA675916	Chicken	New Zealand	Campylobacter jejuni	45	ST-45 complex	https://www.ncbi.nlm.nih.gov/sra/SRR17925388
Ej_SC0521	2018	SRR17925376	PRJNA675916	Chicken	New Zealand	Campylobacter jejuni	2345	ST-206 complex	https://www.ncbi.nlm.nih.gov/sra/SRR17925376
Ej_SC0522	2018	SRR17925365	PRJNA675916	Chicken	New Zealand	Campylobacter jejuni	4337	ST-21 complex	https://www.ncbi.nlm.nih.gov/sra/SRR17925365
Ej_SC0523	2018	SRR17925258	PRJNA675916	Chicken	New Zealand	Campylobacter jejuni	48	ST-48 complex	https://www.ncbi.nlm.nih.gov/sra/SRR17925258
Ej_SC0524	2018	SRR17925247	PRJNA675916	Chicken	New Zealand	Campylobacter jejuni	48	ST-48 complex	https://www.ncbi.nlm.nih.gov/sra/SRR17925247
Ej_SC0525	2018	SRR17925236	PRJNA675916	Chicken	New Zealand	Campylobacter jejuni	48	ST-48 complex	https://www.ncbi.nlm.nih.gov/sra/SRR17925236
Ej_SC0526	2018	SRR17925321	PRJNA675916	Chicken	New Zealand	Campylobacter jejuni	227	ST-206 complex	https://www.ncbi.nlm.nih.gov/sra/SRR17925321
Ej_SC0528	2018	SRR17925299	PRJNA675916	Human	New Zealand	Campylobacter jejuni	6964	ST-354 complex	https://www.ncbi.nlm.nih.gov/sra/SRR17925299
Ej_SC0529	2018	SRR17925288	PRJNA675916	Human	New Zealand	Campylobacter jejuni	53	ST-21 complex	https://www.ncbi.nlm.nih.gov/sra/SRR17925288
Ej_SC0530	2018	SRR17925277	PRJNA675916	Human	New Zealand	Campylobacter jejuni	6964	ST-354 complex	https://www.ncbi.nlm.nih.gov/sra/SRR17925277
Ej_SC0531	2018	SRR17925169	PRJNA675916	Human	New Zealand	Campylobacter jejuni	508	ST-508 complex	https://www.ncbi.nlm.nih.gov/sra/SRR17925169
Ej_SC0536	2018	SRR17925210	PRJNA675916	Human	New Zealand	Campylobacter jejuni	2026	ST-403 complex	https://www.ncbi.nlm.nih.gov/sra/SRR17925210
Ej_SC0537	2018	SRR17925455	PRJNA675916	Human	New Zealand	Campylobacter jejuni	50	ST-21 complex	https://www.ncbi.nlm.nih.gov/sra/SRR17925455
Ej_SC0538	2018	SRR17925444	PRJNA675916	Human	New Zealand	Campylobacter jejuni	190	ST-21 complex	https://www.ncbi.nlm.nih.gov/sra/SRR17925444
Ej_SC0539	2018	SRR17925435	PRJNA675916	Human	New Zealand	Campylobacter jejuni	45	ST-45 complex	https://www.ncbi.nlm.nih.gov/sra/SRR17925435
Ej_SC0553	2018	SRR17925195	PRJNA675916	Human	New Zealand	Campylobacter jejuni	486	ST-21 complex	https://www.ncbi.nlm.nih.gov/sra/SRR17925195
Ej_SC0554	2018	SRR17925194	PRJNA675916	Human	New Zealand	Campylobacter jejuni	42	ST-42 complex	https://www.ncbi.nlm.nih.gov/sra/SRR17925194
Ej_SC0555	2018	SRR17925193	PRJNA675916	Human	New Zealand	Campylobacter jejuni	583	ST-45 complex	https://www.ncbi.nlm.nih.gov/sra/SRR17925193
Ej_SC0556	2018	SRR17925192	PRJNA675916	Human	New Zealand	Campylobacter jejuni	45	ST-45 complex	https://www.ncbi.nlm.nih.gov/sra/SRR17925192
Ej_SC0557	2018	SRR17925191	PRJNA675916	Human	New Zealand	Campylobacter jejuni	45	ST-45 complex	https://www.ncbi.nlm.nih.gov/sra/SRR17925191
Ej_SC0558	2018	SRR17925190	PRJNA675916	Human	New Zealand	Campylobacter jejuni	227	ST-206 complex	https://www.ncbi.nlm.nih.gov/sra/SRR17925190
Ej_SC0560	2018	SRR17925188	PRJNA675916	Human	New Zealand	Campylobacter jejuni	61	ST-61 complex	https://www.ncbi.nlm.nih.gov/sra/SRR17925188
Ej_SC0567	2018	SRR17925180	PRJNA675916	Human	New Zealand	Campylobacter jejuni	48	ST-48 complex	https://www.ncbi.nlm.nih.gov/sra/SRR17925180
Ej_SC0586	2018	SRR17925127	PRJNA675916	Human	New Zealand	Campylobacter jejuni	4337	ST-21 complex	https://www.ncbi.nlm.nih.gov/sra/SRR17925127
Ej_SC0588	2018	SRR17925125	PRJNA675916	Human	New Zealand	Campylobacter jejuni	583	ST-45 complex	https://www.ncbi.nlm.nih.gov/sra/SRR17925125
Ej_SC0589	2018	SRR17925124	PRJNA675916	Human	New Zealand	Campylobacter jejuni	6964	ST-354 complex	https://www.ncbi.nlm.nih.gov/sra/SRR17925124
Ej_SC0592	2018	SRR17925120	PRJNA675916	Human	New Zealand	Campylobacter jejuni	12762	ST-61 complex	https://www.ncbi.nlm.nih.gov/sra/SRR17925120
Ej_SC0595	2018	SRR17925117	PRJNA675916	Human	New Zealand	Campylobacter jejuni	48	ST-48 complex	https://www.ncbi.nlm.nih.gov/sra/SRR17925117
Ej_SC0596	2018	SRR17925116	PRJNA675916	Human	New Zealand	Campylobacter jejuni	48	ST-48 complex	https://www.ncbi.nlm.nih.gov/sra/SRR17925116
Ej_SC0597	2018	SRR17925115	PRJNA675916	Human	New Zealand	Campylobacter jejuni	2345	ST-206 complex	https://www.ncbi.nlm.nih.gov/sra/SRR17925115
Ej_SC0600	2018	SRR17925112	PRJNA675916	Human	New Zealand	Campylobacter jejuni	422	ST-21 complex	https://www.ncbi.nlm.nih.gov/sra/SRR17925112
Ej_SC0601	2018	SRR17925486	PRJNA675916	Chicken	New Zealand	Campylobacter jejuni	583	ST-45 complex	https://www.ncbi.nlm.nih.gov/sra/SRR17925486
Ej_SC0603	2018	SRR17925484	PRJNA675916	Chicken	New Zealand	Campylobacter jejuni	50	ST-21 complex	https://www.ncbi.nlm.nih.gov/sra/SRR17925484

Isolate no.	Year	Accession no.	BioProject no.	Source	Country	Species	Sequence type	Clonal complex	SRA hyperlink
Ej_SC0604	2018	SRR17925483	PRJNA675916	Chicken	New Zealand	Campylobacter jejuni	583	ST-45 complex	https://www.ncbi.nlm.nih.gov/sra/SRR17925483
Ej_SC0605	2018	SRR17925482	PRJNA675916	Chicken	New Zealand	Campylobacter jejuni	583	ST-45 complex	https://www.ncbi.nlm.nih.gov/sra/SRR17925482
Ej_SC0606	2018	SRR17925481	PRJNA675916	Chicken	New Zealand	Campylobacter jejuni	6964	ST-354 complex	https://www.ncbi.nlm.nih.gov/sra/SRR17925481
Ej_SC0607	2018	SRR17925480	PRJNA675916	Chicken	New Zealand	Campylobacter jejuni	227	ST-206 complex	https://www.ncbi.nlm.nih.gov/sra/SRR17925480
Ej_SC0608	2018	SRR17925479	PRJNA675916	Chicken	New Zealand	Campylobacter jejuni	48	ST-48 complex	https://www.ncbi.nlm.nih.gov/sra/SRR17925479
Ej_SC0609	2018	SRR17925478	PRJNA675916	Chicken	New Zealand	Campylobacter jejuni	48	ST-48 complex	https://www.ncbi.nlm.nih.gov/sra/SRR17925478
Ej_SC0610	2018	SRR17925477	PRJNA675916	Chicken	New Zealand	Campylobacter jejuni	1517	ST-354 complex	https://www.ncbi.nlm.nih.gov/sra/SRR17925477
Ej_SC0611	2018	SRR17925475	PRJNA675916	Chicken	New Zealand	Campylobacter jejuni	48	ST-48 complex	https://www.ncbi.nlm.nih.gov/sra/SRR17925475
Ej_SC0612	2018	SRR17925474	PRJNA675916	Chicken	New Zealand	Campylobacter jejuni	6964	ST-354 complex	https://www.ncbi.nlm.nih.gov/sra/SRR17925474
Ej_SC0614	2018	SRR17925472	PRJNA675916	Chicken	New Zealand	Campylobacter jejuni	6964	ST-354 complex	https://www.ncbi.nlm.nih.gov/sra/SRR17925472
Ej_SC0615	2018	SRR17925471	PRJNA675916	Chicken	New Zealand	Campylobacter jejuni	4337	ST-21 complex	https://www.ncbi.nlm.nih.gov/sra/SRR17925471
Ej_SC0616	2018	SRR17925470	PRJNA675916	Chicken	New Zealand	Campylobacter jejuni	6964	ST-354 complex	https://www.ncbi.nlm.nih.gov/sra/SRR17925470
Ej_SC0617	2018	SRR17925469	PRJNA675916	Chicken	New Zealand	Campylobacter jejuni	48	ST-48 complex	https://www.ncbi.nlm.nih.gov/sra/SRR17925469
Ej_SC0619	2018	SRR17925467	PRJNA675916	Chicken	New Zealand	Campylobacter jejuni	6964	ST-354 complex	https://www.ncbi.nlm.nih.gov/sra/SRR17925467
Ej_SC0620	2018	SRR17925466	PRJNA675916	Chicken	New Zealand	Campylobacter jejuni	6964	ST-354 complex	https://www.ncbi.nlm.nih.gov/sra/SRR17925466
Ej_SC0621	2018	SRR17925464	PRJNA675916	Chicken	New Zealand	Campylobacter jejuni	6964	ST-354 complex	https://www.ncbi.nlm.nih.gov/sra/SRR17925464
Ej_SC0624	2018	SRR17925461	PRJNA675916	Human	New Zealand	Campylobacter jejuni	6964	ST-354 complex	https://www.ncbi.nlm.nih.gov/sra/SRR17925461
Ej_SC0626	2018	SRR17925459	PRJNA675916	Human	New Zealand	Campylobacter jejuni	583	ST-45 complex	https://www.ncbi.nlm.nih.gov/sra/SRR17925459
Ej_SC0628	2018	SRR17925361	PRJNA675916	Human	New Zealand	Campylobacter jejuni	2081		https://www.ncbi.nlm.nih.gov/sra/SRR17925361
Ej_SC0629	2018	SRR17925360	PRJNA675916	Human	New Zealand	Campylobacter jejuni	520	ST-21 complex	https://www.ncbi.nlm.nih.gov/sra/SRR17925360
Ej_SC0635	2018	SRR17925353	PRJNA675916	Human	New Zealand	Campylobacter jejuni	6964	ST-354 complex	https://www.ncbi.nlm.nih.gov/sra/SRR17925353
Ej_SC0637	2018	SRR17925351	PRJNA675916	Human	New Zealand	Campylobacter jejuni	696	ST-1332 complex	https://www.ncbi.nlm.nih.gov/sra/SRR17925351
Ej_SC0638	2018	SRR17925350	PRJNA675916	Human	New Zealand	Campylobacter jejuni	422	ST-21 complex	https://www.ncbi.nlm.nih.gov/sra/SRR17925350
Ej_SC0639	2018	SRR17925349	PRJNA675916	Human	New Zealand	Campylobacter jejuni	12761		https://www.ncbi.nlm.nih.gov/sra/SRR17925349
Ej_SC0644	2018	SRR17925343	PRJNA675916	Chicken	New Zealand	Campylobacter jejuni	190	ST-21 complex	https://www.ncbi.nlm.nih.gov/sra/SRR17925343
Ej_SC0645	2018	SRR17925342	PRJNA675916	Chicken	New Zealand	Campylobacter jejuni	227	ST-206 complex	https://www.ncbi.nlm.nih.gov/sra/SRR17925342
Ej_SC0646	2018	SRR17925341	PRJNA675916	Chicken	New Zealand	Campylobacter jejuni	6964	ST-354 complex	https://www.ncbi.nlm.nih.gov/sra/SRR17925341
Ej_SC0647	2018	SRR17925340	PRJNA675916	Chicken	New Zealand	Campylobacter jejuni	45	ST-45 complex	https://www.ncbi.nlm.nih.gov/sra/SRR17925340
Ej_SC0648	2018	SRR17925339	PRJNA675916	Chicken	New Zealand	Campylobacter jejuni	nJ08		https://www.ncbi.nlm.nih.gov/sra/SRR17925339
Ej_SC0649	2018	SRR17925338	PRJNA675916	Human	New Zealand	Campylobacter jejuni	227	ST-206 complex	https://www.ncbi.nlm.nih.gov/sra/SRR17925338
Ej_SC0650	2018	SRR17925337	PRJNA675916	Human	New Zealand	Campylobacter jejuni	6964	ST-354 complex	https://www.ncbi.nlm.nih.gov/sra/SRR17925337
Ej_SC0651	2018	SRR17925335	PRJNA675916	Human	New Zealand	Campylobacter jejuni	1517	ST-354 complex	https://www.ncbi.nlm.nih.gov/sra/SRR17925335
Ej_SC0656	2018	SRR17925330	PRJNA675916	Human	New Zealand	Campylobacter jejuni	227	ST-206 complex	https://www.ncbi.nlm.nih.gov/sra/SRR17925330
Ej_SC0657	2018	SRR17925425	PRJNA675916	Human	New Zealand	Campylobacter jejuni	6964	ST-354 complex	https://www.ncbi.nlm.nih.gov/sra/SRR17925425
Ej_SC0658	2018	SRR17925424	PRJNA675916	Human	New Zealand	Campylobacter jejuni	6964	ST-354 complex	https://www.ncbi.nlm.nih.gov/sra/SRR17925424
Ej_SC0660	2018	SRR17925422	PRJNA675916	Human	New Zealand	Campylobacter jejuni	45	ST-45 complex	https://www.ncbi.nlm.nih.gov/sra/SRR17925422
Ej_SC0661	2018	SRR17925420	PRJNA675916	Human	New Zealand	Campylobacter jejuni	53	ST-21 complex	https://www.ncbi.nlm.nih.gov/sra/SRR17925420
Ej_SC0663	2018	SRR17925418	PRJNA675916	Human	New Zealand	Campylobacter jejuni	422	ST-21 complex	https://www.ncbi.nlm.nih.gov/sra/SRR17925418
Ej_SC0665	2018	SRR17925416	PRJNA675916	Human	New Zealand	Campylobacter jejuni	53	ST-21 complex	https://www.ncbi.nlm.nih.gov/sra/SRR17925416
Ej_SC0666	2018	SRR17925415	PRJNA675916	Human	New Zealand	Campylobacter jejuni	21	ST-21 complex	https://www.ncbi.nlm.nih.gov/sra/SRR17925415
Ej_SC0668	2018	SRR17925414	PRJNA675916	Human	New Zealand	Campylobacter jejuni	2345	ST-206 complex	https://www.ncbi.nlm.nih.gov/sra/SRR17925414
Ej_SC0671	2018	SRR17925411	PRJNA675916	Chicken	New Zealand	Campylobacter jejuni	2345	ST-206 complex	https://www.ncbi.nlm.nih.gov/sra/SRR17925411
Ej_SC0672	2018	SRR17925409	PRJNA675916	Chicken	New Zealand	Campylobacter jejuni	583	ST-45 complex	https://www.ncbi.nlm.nih.gov/sra/SRR17925409
Ej_SC0673	2018	SRR17925408	PRJNA675916	Chicken	New Zealand	Campylobacter jejuni	52	ST-52 complex	https://www.ncbi.nlm.nih.gov/sra/SRR17925408
Ej_SC0675	2018	SRR17925406	PRJNA675916	Chicken	New Zealand	Campylobacter jejuni	8185	ST-21 complex	https://www.ncbi.nlm.nih.gov/sra/SRR17925406
Ej_SC0676	2018	SRR17925405	PRJNA675916	Chicken	New Zealand	Campylobacter jejuni	190	ST-21 complex	https://www.ncbi.nlm.nih.gov/sra/SRR17925405
Ej_SC0678	2018	SRR17925403	PRJNA675916	Chicken	New Zealand	Campylobacter jejuni	48	ST-48 complex	https://www.ncbi.nlm.nih.gov/sra/SRR17925403
Ej_SC0679	2018	SRR17925402	PRJNA675916	Chicken	New Zealand	Campylobacter jejuni	48	ST-48 complex	https://www.ncbi.nlm.nih.gov/sra/SRR17925402
Ej_SC0680	2018	SRR17925401	PRJNA675916	Chicken	New Zealand	Campylobacter jejuni	583	ST-45 complex	https://www.ncbi.nlm.nih.gov/sra/SRR17925401
Ej_SC0681	2018	SRR17925400	PRJNA675916	Chicken	New Zealand	Campylobacter jejuni	6964	ST-354 complex	https://www.ncbi.nlm.nih.gov/sra/SRR17925400
Ej_SC0684	2018	SRR17925396	PRJNA675916	Human	New Zealand	Campylobacter jejuni	677	ST-677 complex	https://www.ncbi.nlm.nih.gov/sra/SRR17925396
Ej_SC0685	2018	SRR17925395	PRJNA675916	Human	New Zealand	Campylobacter jejuni	25	ST-45 complex	https://www.ncbi.nlm.nih.gov/sra/SRR17925395
Ej_SC0687	2018	SRR17925393	PRJNA675916	Human	New Zealand	Campylobacter jejuni	45	ST-45 complex	https://www.ncbi.nlm.nih.gov/sra/SRR17925393
Ej_SC0688	2018	SRR17925392	PRJNA675916	Human	New Zealand	Campylobacter jejuni	45	ST-45 complex	https://www.ncbi.nlm.nih.gov/sra/SRR17925392
Ej_SC0689	2018	SRR17925391	PRJNA675916	Human	New Zealand	Campylobacter jejuni	48	ST-48 complex	https://www.ncbi.nlm.nih.gov/sra/SRR17925391
Ej_SC0690	2018	SRR17925390	PRJNA675916	Human	New Zealand	Campylobacter jejuni	48	ST-48 complex	https://www.ncbi.nlm.nih.gov/sra/SRR17925390
Ej_SC0691	2018	SRR17925389	PRJNA675916	Human	New Zealand	Campylobacter jejuni	696	ST-1332 complex	https://www.ncbi.nlm.nih.gov/sra/SRR17925389
Ej_SC0692	2018	SRR17925387	PRJNA675916	Human	New Zealand	Campylobacter jejuni	6964	ST-354 complex	https://www.ncbi.nlm.nih.gov/sra/SRR17925387
Ej_SC0698	2018	SRR17925381	PRJNA675916	Human	New Zealand	Campylobacter jejuni	48	ST-48 complex	https://www.ncbi.nlm.nih.gov/sra/SRR17925381
Ej_SC0700	2018	SRR17925379	PRJNA675916	Human	New Zealand	Campylobacter jejuni	45	ST-45 complex	https://www.ncbi.nlm.nih.gov/sra/SRR17925379
Ej_SC0702	2018	SRR17925375	PRJNA675916	Human	New Zealand	Campylobacter jejuni	48	ST-48 complex	https://www.ncbi.nlm.nih.gov/sra/SRR17925375
Ej_SC0703	2018	SRR17925374	PRJNA675916	Human	New Zealand	Campylobacter jejuni	45	ST-45 complex	https://www.ncbi.nlm.nih.gov/sra/SRR17925374
Ej_SC0705	2018	SRR17925372	PRJNA675916	Human	New Zealand	Campylobacter jejuni	227	ST-206 complex	https://www.ncbi.nlm.nih.gov/sra/SRR17925372
Ej_SC0721	2018	SRR17925261	PRJNA675916	Human	New Zealand	Campylobacter jejuni	227	ST-206 complex	https://www.ncbi.nlm.nih.gov/sra/SRR17925261
Ej_SC0722	2018	SRR17925260	PRJNA675916	Human	New Zealand	Campylobacter jejuni	50	ST-21 complex	https://www.ncbi.nlm.nih.gov/sra/SRR17925260
Ej_SC0723	2018	SRR17925259	PRJNA675916	Human	New Zealand	Campylobacter jejuni	6964	ST-354 complex	https://www.ncbi.nlm.nih.gov/sra/SRR17925259
Ej_SC0724	2018	SRR17925257	PRJNA675916	Human	New Zealand	Campylobacter jejuni	45	ST-45 complex	https://www.ncbi.nlm.nih.gov/sra/SRR17925257
Ej_SC0727	2018	SRR17925254	PRJNA675916	Human	New Zealand	Campylobacter jejuni	48	ST-48 complex	https://www.ncbi.nlm.nih.gov/sra/SRR17925254
Ej_SC0730	2018	SRR17925251	PRJNA675916	Human	New Zealand	Campylobacter jejuni	6964	ST-354 complex	https://www.ncbi.nlm.nih.gov/sra/SRR17925251
Ej_SC0732	2018	SRR17925249	PRJNA675916	Human	New Zealand	Campylobacter jejuni	474	ST-48 complex	https://www.ncbi.nlm.nih.gov/sra/SRR17925249
Ej_SC0733	2018	SRR17925248	PRJNA675916	Human	New Zealand	Campylobacter jejuni	42	ST-42 complex	https://www.ncbi.nlm.nih.gov/sra/SRR17925248
Ej_SC0734	2018	SRR17925246	PRJNA675916	Human	New Zealand	Campylobacter jejuni	50	ST-21 complex	https://www.ncbi.nlm.nih.gov/sra/SRR17925246
Ej_SC0735	2018	SRR17925245	PRJNA675916	Human	New Zealand	Campylobacter jejuni	4337	ST-21 complex	https://www.ncbi.nlm.nih.gov/sra/SRR17925245
Ej_SC0736	2018	SRR17925244	PRJNA675916	Human	New Zealand	Campylobacter jejuni	21	ST-21 complex	https://www.ncbi.nlm.nih.gov/sra/SRR17925244
Ej_SC0741	2018	SRR17925239	PRJNA675916	Human	New Zealand	Campylobacter jejuni	48	ST-48 complex	https://www.ncbi.nlm.nih.gov/sra/SRR17925239
Ej_SC0744	2018	SRR17925235	PRJNA675916	Human	New Zealand	Campylobacter jejuni	227	ST-206 complex	https://www.ncbi.nlm.nih.gov/sra/SRR17925235
Ej_SC0747	2018	SRR17925328	PRJNA675916	Human	New Zealand	Campylobacter jejuni	508	ST-508 complex	https://www.ncbi.nlm.nih.gov/sra/SRR17925328
Ej_SC0748	2018	SRR17925327	PRJNA675916	Human	New Zealand	Campylobacter jejuni	6964	ST-354 complex	https://www.ncbi.nlm.nih.gov/sra/SRR17925327
Ej_SC0750	2018	SRR17925325	PRJNA675916	Human	New Zealand	Campylobacter jejuni	137	ST-45 complex	https://www.ncbi.nlm.nih.gov/sra/SRR17925325
Ej_SC0751	2018	SRR17925324	PRJNA675916	Human	New Zealand	Campylobacter jejuni	2026	ST-403 complex	https://www.ncbi.nlm.nih.gov/sra/SRR17925324
Ej_SC0753	2018	SRR17925322	PRJNA675916	Human	New Zealand	Campylobacter jejuni	48	ST-48 complex	https://www.ncbi.nlm.nih.gov/sra/SRR17925322
Ej_SC0754	2018	SRR17925320	PRJNA675916	Human	New Zealand	Campylobacter jejuni	6964	ST-354 complex	https://www.ncbi.nlm.nih.gov/sra/SRR17925320
Ej_SC0756	2018	SRR17925318	PRJNA675916	Human	New Zealand	Campylobacter jejuni	190	ST-21 complex	https://www.ncbi.nlm.nih.gov/sra/SRR17925318
Ej_SC0758	2018	SRR17925316	PRJNA675916	Human	New Zealand	Campylobacter jejuni	45	ST-45 complex	https://www.ncbi.nlm.nih.gov/sra/SRR17925316
Ej_SC0762	2018	SRR17925312	PRJNA675916	Chicken	New Zealand	Campylobacter jejuni	6964	ST-354 complex	https://www.ncbi.nlm.nih.gov/sra/SRR17925312
Ej_SC0763	2018	SRR17925311	PRJNA675916	Chicken	New Zealand	Campylobacter jejuni	6964	ST-354 complex	https://www.ncbi.nlm.nih.gov/sra/SRR17925311
Ej_SC0764	2018	SRR17925309	PRJNA675916	Chicken	New Zealand	Campylobacter jejuni	4337	ST-21 complex	https://www.ncbi.nlm.nih.gov/sra/SRR17925309

Isolate no.	Year	Accession no.	BioProject no.	Source	Country	Species	Sequence type	Clonal complex	SRA hyperlink
Ej_SC0765	2018	SRR17925308	PRJNA675916	Chicken	New Zealand	Campylobacter jejuni	6964	ST-354 complex	https://www.ncbi.nlm.nih.gov/sra/SRR17925308
Ej_SC0770	2018	SRR17925303	PRJNA675916	Human	New Zealand	Campylobacter jejuni	51	ST-443 complex	https://www.ncbi.nlm.nih.gov/sra/SRR17925303
Ej_SC0771	2018	SRR17925302	PRJNA675916	Human	New Zealand	Campylobacter jejuni	6964	ST-354 complex	https://www.ncbi.nlm.nih.gov/sra/SRR17925302
Ej_SC0773	2018	SRR17925300	PRJNA675916	Human	New Zealand	Campylobacter jejuni	486	ST-21 complex	https://www.ncbi.nlm.nih.gov/sra/SRR17925300
Ej_SC0777	2018	SRR17925295	PRJNA675916	Human	New Zealand	Campylobacter jejuni	486	ST-21 complex	https://www.ncbi.nlm.nih.gov/sra/SRR17925295
Ej_SC0780	2018	SRR17925292	PRJNA675916	Human	New Zealand	Campylobacter jejuni	486	ST-21 complex	https://www.ncbi.nlm.nih.gov/sra/SRR17925292
Ej_SC0781	2018	SRR17925291	PRJNA675916	Human	New Zealand	Campylobacter jejuni	48	ST-48 complex	https://www.ncbi.nlm.nih.gov/sra/SRR17925291
Ej_SC0783	2018	SRR17925289	PRJNA675916	Human	New Zealand	Campylobacter jejuni	190	ST-21 complex	https://www.ncbi.nlm.nih.gov/sra/SRR17925289
Ej_SC0784	2018	SRR17925287	PRJNA675916	Chicken	New Zealand	Campylobacter jejuni	583	ST-45 complex	https://www.ncbi.nlm.nih.gov/sra/SRR17925287
Ej_SC0785	2018	SRR17925286	PRJNA675916	Chicken	New Zealand	Campylobacter jejuni	583	ST-45 complex	https://www.ncbi.nlm.nih.gov/sra/SRR17925286
Ej_SC0786	2018	SRR17925285	PRJNA675916	Chicken	New Zealand	Campylobacter jejuni	486	ST-21 complex	https://www.ncbi.nlm.nih.gov/sra/SRR17925285
Ej_SC0787	2018	SRR17925284	PRJNA675916	Chicken	New Zealand	Campylobacter jejuni	6964	ST-354 complex	https://www.ncbi.nlm.nih.gov/sra/SRR17925284
Ej_SC0788	2018	SRR17925283	PRJNA675916	Chicken	New Zealand	Campylobacter jejuni	48	ST-48 complex	https://www.ncbi.nlm.nih.gov/sra/SRR17925283
Ej_SC0789	2018	SRR17925282	PRJNA675916	Chicken	New Zealand	Campylobacter jejuni	2345	ST-206 complex	https://www.ncbi.nlm.nih.gov/sra/SRR17925282
Ej_SC0791	2018	SRR17925280	PRJNA675916	Human	New Zealand	Campylobacter jejuni	227	ST-206 complex	https://www.ncbi.nlm.nih.gov/sra/SRR17925280
Ej_SC0792	2018	SRR17925279	PRJNA675916	Human	New Zealand	Campylobacter jejuni	677	ST-677 complex	https://www.ncbi.nlm.nih.gov/sra/SRR17925279
Ej_SC0795	2018	SRR17925275	PRJNA675916	Human	New Zealand	Campylobacter jejuni	257	ST-257 complex	https://www.ncbi.nlm.nih.gov/sra/SRR17925275
Ej_SC0796	2018	SRR17925274	PRJNA675916	Human	New Zealand	Campylobacter jejuni	50	ST-21 complex	https://www.ncbi.nlm.nih.gov/sra/SRR17925274
Ej_SC0797	2018	SRR17925273	PRJNA675916	Human	New Zealand	Campylobacter jejuni	4337	ST-21 complex	https://www.ncbi.nlm.nih.gov/sra/SRR17925273
Ej_SC0799	2018	SRR17925271	PRJNA675916	Human	New Zealand	Campylobacter jejuni	227	ST-206 complex	https://www.ncbi.nlm.nih.gov/sra/SRR17925271
Ej_SC0801	2018	SRR17925269	PRJNA675916	Human	New Zealand	Campylobacter jejuni	nJ07		https://www.ncbi.nlm.nih.gov/sra/SRR17925269
Ej_SC0803	2018	SRR17925267	PRJNA675916	Human	New Zealand	Campylobacter jejuni	520	ST-21 complex	https://www.ncbi.nlm.nih.gov/sra/SRR17925267
Ej_SC0805	2018	SRR17925167	PRJNA675916	Human	New Zealand	Campylobacter jejuni	6964	ST-354 complex	https://www.ncbi.nlm.nih.gov/sra/SRR17925167
Ej_SC0807	2018	SRR17925165	PRJNA675916	Human	New Zealand	Campylobacter jejuni	227	ST-206 complex	https://www.ncbi.nlm.nih.gov/sra/SRR17925165
Ej_SC0808	2018	SRR17925164	PRJNA675916	Human	New Zealand	Campylobacter jejuni	45	ST-45 complex	https://www.ncbi.nlm.nih.gov/sra/SRR17925164
Ej_SC0809	2018	SRR17925163	PRJNA675916	Human	New Zealand	Campylobacter jejuni	190	ST-21 complex	https://www.ncbi.nlm.nih.gov/sra/SRR17925163
Ej_SC0811	2018	SRR17925161	PRJNA675916	Human	New Zealand	Campylobacter jejuni	21	ST-21 complex	https://www.ncbi.nlm.nih.gov/sra/SRR17925161
Ej_SC0812	2018	SRR17925160	PRJNA675916	Human	New Zealand	Campylobacter jejuni	48	ST-48 complex	https://www.ncbi.nlm.nih.gov/sra/SRR17925160
Ej_SC0821	2018	SRR17925150	PRJNA675916	Chicken	New Zealand	Campylobacter jejuni	6964	ST-354 complex	https://www.ncbi.nlm.nih.gov/sra/SRR17925150
Ej_SC0823	2018	SRR17925149	PRJNA675916	Human	New Zealand	Campylobacter jejuni	48	ST-48 complex	https://www.ncbi.nlm.nih.gov/sra/SRR17925149
Ej_SC0824	2018	SRR17925148	PRJNA675916	Human	New Zealand	Campylobacter jejuni	53	ST-21 complex	https://www.ncbi.nlm.nih.gov/sra/SRR17925148
Ej_SC0826	2018	SRR17925146	PRJNA675916	Human	New Zealand	Campylobacter jejuni	227	ST-206 complex	https://www.ncbi.nlm.nih.gov/sra/SRR17925146
Ej_SC0827	2018	SRR17925145	PRJNA675916	Human	New Zealand	Campylobacter jejuni	42	ST-42 complex	https://www.ncbi.nlm.nih.gov/sra/SRR17925145
Ej_SC0830	2018	SRR17925144	PRJNA675916	Human	New Zealand	Campylobacter jejuni	4337	ST-21 complex	https://www.ncbi.nlm.nih.gov/sra/SRR17925144
Ej_SC0832	2018	SRR17925143	PRJNA675916	Human	New Zealand	Campylobacter jejuni	48	ST-48 complex	https://www.ncbi.nlm.nih.gov/sra/SRR17925143
Ej_SC0834	2018	SRR17925142	PRJNA675916	Human	New Zealand	Campylobacter jejuni	190	ST-21 complex	https://www.ncbi.nlm.nih.gov/sra/SRR17925142
Ej_SC0836	2018	SRR17925141	PRJNA675916	Human	New Zealand	Campylobacter jejuni	48	ST-48 complex	https://www.ncbi.nlm.nih.gov/sra/SRR17925141
Ej_SC0837	2018	SRR17925140	PRJNA675916	Human	New Zealand	Campylobacter jejuni	53	ST-21 complex	https://www.ncbi.nlm.nih.gov/sra/SRR17925140
Ej_SC0838	2018	SRR17925139	PRJNA675916	Human	New Zealand	Campylobacter jejuni	2345	ST-206 complex	https://www.ncbi.nlm.nih.gov/sra/SRR17925139
Ej_SC0841	2018	SRR17925233	PRJNA675916	Human	New Zealand	Campylobacter jejuni	42	ST-42 complex	https://www.ncbi.nlm.nih.gov/sra/SRR17925233
Ej_SC0845	2018	SRR17925231	PRJNA675916	Human	New Zealand	Campylobacter jejuni	4337	ST-21 complex	https://www.ncbi.nlm.nih.gov/sra/SRR17925231
Ej_SC0846	2018	SRR17925230	PRJNA675916	Human	New Zealand	Campylobacter jejuni	6964	ST-354 complex	https://www.ncbi.nlm.nih.gov/sra/SRR17925230
Ej_SC0862	2018	SRR17925215	PRJNA675916	Chicken	New Zealand	Campylobacter jejuni	45	ST-45 complex	https://www.ncbi.nlm.nih.gov/sra/SRR17925215
Ej_SC0863	2018	SRR17925214	PRJNA675916	Human	New Zealand	Campylobacter jejuni	53	ST-21 complex	https://www.ncbi.nlm.nih.gov/sra/SRR17925214
Ej_SC0864	2018	SRR17925213	PRJNA675916	Chicken	New Zealand	Campylobacter jejuni	6964	ST-354 complex	https://www.ncbi.nlm.nih.gov/sra/SRR17925213
Ej_SC0865	2018	SRR17925212	PRJNA675916	Chicken	New Zealand	Campylobacter jejuni	4337	ST-21 complex	https://www.ncbi.nlm.nih.gov/sra/SRR17925212
Ej_SC0866	2018	SRR17925211	PRJNA675916	Chicken	New Zealand	Campylobacter jejuni	6964	ST-354 complex	https://www.ncbi.nlm.nih.gov/sra/SRR17925211
Ej_SC0867	2018	SRR17925209	PRJNA675916	Chicken	New Zealand	Campylobacter jejuni	6964	ST-354 complex	https://www.ncbi.nlm.nih.gov/sra/SRR17925209
Ej_SC0870	2018	SRR17925207	PRJNA675916	Human	New Zealand	Campylobacter jejuni	520	ST-21 complex	https://www.ncbi.nlm.nih.gov/sra/SRR17925207
Ej_SC0871	2018	SRR17925206	PRJNA675916	Human	New Zealand	Campylobacter jejuni	6964	ST-354 complex	https://www.ncbi.nlm.nih.gov/sra/SRR17925206
Ej_SC0873	2018	SRR17925205	PRJNA675916	Human	New Zealand	Campylobacter jejuni	45	ST-45 complex	https://www.ncbi.nlm.nih.gov/sra/SRR17925205
Ej_SC0874	2018	SRR17925204	PRJNA675916	Human	New Zealand	Campylobacter jejuni	45	ST-45 complex	https://www.ncbi.nlm.nih.gov/sra/SRR17925204
Ej_SC0876	2018	SRR17925203	PRJNA675916	Human	New Zealand	Campylobacter jejuni	1326	ST-45 complex	https://www.ncbi.nlm.nih.gov/sra/SRR17925203
Ej_SC0877	2018	SRR17925202	PRJNA675916	Human	New Zealand	Campylobacter jejuni	61	ST-61 complex	https://www.ncbi.nlm.nih.gov/sra/SRR17925202
Ej_SC0878	2018	SRR17925457	PRJNA675916	Human	New Zealand	Campylobacter jejuni	45	ST-45 complex	https://www.ncbi.nlm.nih.gov/sra/SRR17925457
Ej_SC0879	2018	SRR17925456	PRJNA675916	Human	New Zealand	Campylobacter jejuni	45	ST-45 complex	https://www.ncbi.nlm.nih.gov/sra/SRR17925456
Ej_SC0880	2018	SRR17925454	PRJNA675916	Human	New Zealand	Campylobacter jejuni	38	ST-48 complex	https://www.ncbi.nlm.nih.gov/sra/SRR17925454
Ej_SC0881	2018	SRR17925453	PRJNA675916	Human	New Zealand	Campylobacter jejuni	50	ST-21 complex	https://www.ncbi.nlm.nih.gov/sra/SRR17925453
Ej_SC0882	2018	SRR17925452	PRJNA675916	Human	New Zealand	Campylobacter jejuni	45	ST-45 complex	https://www.ncbi.nlm.nih.gov/sra/SRR17925452
Ej_SC0883	2018	SRR17925451	PRJNA675916	Human	New Zealand	Campylobacter jejuni	45	ST-45 complex	https://www.ncbi.nlm.nih.gov/sra/SRR17925451
Ej_SC0886	2018	SRR17925450	PRJNA675916	Human	New Zealand	Campylobacter jejuni	45	ST-45 complex	https://www.ncbi.nlm.nih.gov/sra/SRR17925450
Ej_SC0887	2018	SRR17925449	PRJNA675916	Human	New Zealand	Campylobacter jejuni	2026	ST-403 complex	https://www.ncbi.nlm.nih.gov/sra/SRR17925449
Ej_SC0888	2018	SRR17925448	PRJNA675916	Human	New Zealand	Campylobacter jejuni	3538		https://www.ncbi.nlm.nih.gov/sra/SRR17925448
Ej_SC0889	2018	SRR17925447	PRJNA675916	Human	New Zealand	Campylobacter jejuni	583	ST-45 complex	https://www.ncbi.nlm.nih.gov/sra/SRR17925447
Ej_SC0907	2018	SRR17929532	PRJNA675916	Human	New Zealand	Campylobacter jejuni	538	ST-45 complex	https://www.ncbi.nlm.nih.gov/sra/SRR17929532
Ej_SC0908	2018	SRR17929521	PRJNA675916	Human	New Zealand	Campylobacter jejuni	6964	ST-354 complex	https://www.ncbi.nlm.nih.gov/sra/SRR17929521
Ej_SC0910	2018	SRR17929591	PRJNA675916	Human	New Zealand	Campylobacter jejuni	45	ST-45 complex	https://www.ncbi.nlm.nih.gov/sra/SRR17929591
Ej_SC0911	2018	SRR17929580	PRJNA675916	Human	New Zealand	Campylobacter jejuni	48	ST-48 complex	https://www.ncbi.nlm.nih.gov/sra/SRR17929580
Ej_SC0914	2018	SRR17929631	PRJNA675916	Human	New Zealand	Campylobacter jejuni	61	ST-61 complex	https://www.ncbi.nlm.nih.gov/sra/SRR17929631
Ej_SC0918	2018	SRR17929620	PRJNA675916	Human	New Zealand	Campylobacter jejuni	583	ST-45 complex	https://www.ncbi.nlm.nih.gov/sra/SRR17929620
Ej_SC0920	2018	SRR17929609	PRJNA675916	Human	New Zealand	Campylobacter jejuni	4337	ST-21 complex	https://www.ncbi.nlm.nih.gov/sra/SRR17929609
Ej_SC0923	2018	SRR17929600	PRJNA675916	Human	New Zealand	Campylobacter jejuni	4337	ST-21 complex	https://www.ncbi.nlm.nih.gov/sra/SRR17929600
Ej_SC0925	2018	SRR17929599	PRJNA675916	Human	New Zealand	Campylobacter jejuni	422	ST-21 complex	https://www.ncbi.nlm.nih.gov/sra/SRR17929599
Ej_SC0926	2018	SRR17929598	PRJNA675916	Human	New Zealand	Campylobacter jejuni	45	ST-45 complex	https://www.ncbi.nlm.nih.gov/sra/SRR17929598
Ej_SC0927	2018	SRR17929569	PRJNA675916	Human	New Zealand	Campylobacter jejuni	227	ST-206 complex	https://www.ncbi.nlm.nih.gov/sra/SRR17929569
Ej_SC0928	2018	SRR17929568	PRJNA675916	Human	New Zealand	Campylobacter jejuni	508	ST-508 complex	https://www.ncbi.nlm.nih.gov/sra/SRR17929568
Ej_SC0929	2018	SRR17929567	PRJNA675916	Human	New Zealand	Campylobacter jejuni	677	ST-677 complex	https://www.ncbi.nlm.nih.gov/sra/SRR17929567
Ej_SC0930	2018	SRR17929566	PRJNA675916	Human	New Zealand	Campylobacter jejuni	508	ST-508 complex	https://www.ncbi.nlm.nih.gov/sra/SRR17929566
Ej_SC0932	2018	SRR17929564	PRJNA675916	Human	New Zealand	Campylobacter jejuni	583	ST-45 complex	https://www.ncbi.nlm.nih.gov/sra/SRR17929564
Ej_SC0933	2018	SRR17929563	PRJNA675916	Human	New Zealand	Campylobacter jejuni	50	ST-21 complex	https://www.ncbi.nlm.nih.gov/sra/SRR17929563
Ej_SC0934	2018	SRR17929562	PRJNA675916	Human	New Zealand	Campylobacter jejuni	53	ST-21 complex	https://www.ncbi.nlm.nih.gov/sra/SRR17929562
Ej_SC0936	2018	SRR17929561	PRJNA675916	Human	New Zealand	Campylobacter jejuni	5655	ST-21 complex	https://www.ncbi.nlm.nih.gov/sra/SRR17929561
Ej_SC0937	2018	SRR17929560	PRJNA675916	Human	New Zealand	Campylobacter jejuni	227	ST-206 complex	https://www.ncbi.nlm.nih.gov/sra/SRR17929560
Ej_SC0938	2018	SRR17929559	PRJNA675916	Human	New Zealand	Campylobacter jejuni	227	ST-206 complex	https://www.ncbi.nlm.nih.gov/sra/SRR17929559
Ej_SC0939	2018	SRR17929558	PRJNA675916	Human	New Zealand	Campylobacter jejuni	2345	ST-206 complex	https://www.ncbi.nlm.nih.gov/sra/SRR17929558
Ej_SC0942	2018	SRR17929556	PRJNA675916	Chicken	New Zealand	Campylobacter jejuni	4337	ST-21 complex	https://www.ncbi.nlm.nih.gov/sra/SRR17929556
Ej_SC0943	2018	SRR17929555	PRJNA675916	Chicken	New Zealand	Campylobacter jejuni	535	ST-460 complex	https://www.ncbi.nlm.nih.gov/sra/SRR17929555

Isolate no.	Year	Accession no.	BioProject no.	Source	Country	Species	Sequence type	Clonal complex	SRA hyperlink
Ej_SC0944	2018	SRR17929553	PRJNA675916	Chicken	New Zealand	Campylobacter jejuni	2345	ST-206 complex	https://www.ncbi.nlm.nih.gov/sra/SRR17929553
Ej_SC0945	2018	SRR17929552	PRJNA675916	Chicken	New Zealand	Campylobacter jejuni	nJ05		https://www.ncbi.nlm.nih.gov/sra/SRR17929552
Ej_SC0946	2018	SRR17929551	PRJNA675916	Chicken	New Zealand	Campylobacter jejuni	45	ST-45 complex	https://www.ncbi.nlm.nih.gov/sra/SRR17929551
Ej_SC0950	2018	SRR17929547	PRJNA675916	Chicken	New Zealand	Campylobacter jejuni	53	ST-21 complex	https://www.ncbi.nlm.nih.gov/sra/SRR17929547
Ej_SC0964	2018	SRR17929531	PRJNA675916	Human	New Zealand	Campylobacter jejuni	3538		https://www.ncbi.nlm.nih.gov/sra/SRR17929531
Ej_SC0966	2018	SRR17929530	PRJNA675916	Human	New Zealand	Campylobacter jejuni	2535		https://www.ncbi.nlm.nih.gov/sra/SRR17929530
Ej_SC0968	2018	SRR17929529	PRJNA675916	Human	New Zealand	Campylobacter jejuni	45	ST-45 complex	https://www.ncbi.nlm.nih.gov/sra/SRR17929529
Ej_SC0971	2018	SRR17929528	PRJNA675916	Human	New Zealand	Campylobacter jejuni	48	ST-48 complex	https://www.ncbi.nlm.nih.gov/sra/SRR17929528
Ej_SC0973	2018	SRR17929527	PRJNA675916	Human	New Zealand	Campylobacter jejuni	45	ST-45 complex	https://www.ncbi.nlm.nih.gov/sra/SRR17929527
Ej_SC0975	2018	SRR17929526	PRJNA675916	Human	New Zealand	Campylobacter jejuni	45	ST-45 complex	https://www.ncbi.nlm.nih.gov/sra/SRR17929526
Ej_SC0976	2018	SRR17929525	PRJNA675916	Human	New Zealand	Campylobacter jejuni	53	ST-21 complex	https://www.ncbi.nlm.nih.gov/sra/SRR17929525
Ej_SC0977	2018	SRR17929524	PRJNA675916	Human	New Zealand	Campylobacter jejuni	45	ST-45 complex	https://www.ncbi.nlm.nih.gov/sra/SRR17929524
Ej_SC0978	2018	SRR17929523	PRJNA675916	Human	New Zealand	Campylobacter jejuni	45	ST-45 complex	https://www.ncbi.nlm.nih.gov/sra/SRR17929523
Ej_SC0980	2018	SRR17929522	PRJNA675916	Human	New Zealand	Campylobacter jejuni	45	ST-45 complex	https://www.ncbi.nlm.nih.gov/sra/SRR17929522
Ej_SC0981	2018	SRR17929520	PRJNA675916	Human	New Zealand	Campylobacter jejuni	45	ST-45 complex	https://www.ncbi.nlm.nih.gov/sra/SRR17929520
Ej_SC0986	2018	SRR17929519	PRJNA675916	Human	New Zealand	Campylobacter jejuni	583	ST-45 complex	https://www.ncbi.nlm.nih.gov/sra/SRR17929519
Ej_SC0988	2018	SRR17929517	PRJNA675916	Chicken	New Zealand	Campylobacter jejuni	6964	ST-354 complex	https://www.ncbi.nlm.nih.gov/sra/SRR17929517
Ej_SC0989	2018	SRR17929516	PRJNA675916	Chicken	New Zealand	Campylobacter jejuni	6964	ST-354 complex	https://www.ncbi.nlm.nih.gov/sra/SRR17929516
Ej_SC0993	2018	SRR17929515	PRJNA675916	Human	New Zealand	Campylobacter jejuni	45	ST-45 complex	https://www.ncbi.nlm.nih.gov/sra/SRR17929515
Ej_SC0994	2018	SRR17929514	PRJNA675916	Human	New Zealand	Campylobacter jejuni	53	ST-21 complex	https://www.ncbi.nlm.nih.gov/sra/SRR17929514
Ej_SC0995	2018	SRR17929513	PRJNA675916	Human	New Zealand	Campylobacter jejuni	48	ST-48 complex	https://www.ncbi.nlm.nih.gov/sra/SRR17929513
Ej_SC0997	2018	SRR17929512	PRJNA675916	Human	New Zealand	Campylobacter jejuni	50	ST-21 complex	https://www.ncbi.nlm.nih.gov/sra/SRR17929512
Ej_SC0999	2018	SRR17929511	PRJNA675916	Human	New Zealand	Campylobacter jejuni	677	ST-677 complex	https://www.ncbi.nlm.nih.gov/sra/SRR17929511
Ej_SC1001	2018	SRR17929509	PRJNA675916	Human	New Zealand	Campylobacter jejuni	2345	ST-206 complex	https://www.ncbi.nlm.nih.gov/sra/SRR17929509
Ej_SC1003	2018	SRR17929508	PRJNA675916	Human	New Zealand	Campylobacter jejuni	354	ST-354 complex	https://www.ncbi.nlm.nih.gov/sra/SRR17929508
Ej_SC1005	2018	SRR17929507	PRJNA675916	Human	New Zealand	Campylobacter jejuni	45	ST-45 complex	https://www.ncbi.nlm.nih.gov/sra/SRR17929507
Ej_SC1006	2018	SRR17929506	PRJNA675916	Human	New Zealand	Campylobacter jejuni	45	ST-45 complex	https://www.ncbi.nlm.nih.gov/sra/SRR17929506
Ej_SC1007	2018	SRR17929597	PRJNA675916	Human	New Zealand	Campylobacter jejuni	583	ST-45 complex	https://www.ncbi.nlm.nih.gov/sra/SRR17929597
Ej_SC1009	2018	SRR17929596	PRJNA675916	Human	New Zealand	Campylobacter jejuni	474	ST-48 complex	https://www.ncbi.nlm.nih.gov/sra/SRR17929596
Ej_SC1011	2018	SRR17929594	PRJNA675916	Chicken	New Zealand	Campylobacter jejuni	2345	ST-206 complex	https://www.ncbi.nlm.nih.gov/sra/SRR17929594
Ej_SC1012	2018	SRR17929593	PRJNA675916	Chicken	New Zealand	Campylobacter jejuni	53	ST-21 complex	https://www.ncbi.nlm.nih.gov/sra/SRR17929593
Ej_SC1013	2018	SRR17929592	PRJNA675916	Chicken	New Zealand	Campylobacter jejuni	422	ST-21 complex	https://www.ncbi.nlm.nih.gov/sra/SRR17929592
Ej_SC1015	2018	SRR17929590	PRJNA675916	Human	New Zealand	Campylobacter jejuni	45	ST-45 complex	https://www.ncbi.nlm.nih.gov/sra/SRR17929590
Ej_SC1016	2018	SRR17929589	PRJNA675916	Human	New Zealand	Campylobacter jejuni	12755		https://www.ncbi.nlm.nih.gov/sra/SRR17929589
Ej_SC1017	2018	SRR17929588	PRJNA675916	Human	New Zealand	Campylobacter jejuni	61	ST-61 complex	https://www.ncbi.nlm.nih.gov/sra/SRR17929588
Ej_SC1019	2018	SRR17929587	PRJNA675916	Human	New Zealand	Campylobacter jejuni	677	ST-677 complex	https://www.ncbi.nlm.nih.gov/sra/SRR17929587
Ej_SC1021	2018	SRR17929586	PRJNA675916	Human	New Zealand	Campylobacter jejuni	583	ST-45 complex	https://www.ncbi.nlm.nih.gov/sra/SRR17929586
Ej_SC1022	2018	SRR17929585	PRJNA675916	Human	New Zealand	Campylobacter jejuni	699	ST-692 complex	https://www.ncbi.nlm.nih.gov/sra/SRR17929585
Ej_SC1026	2018	SRR17929584	PRJNA675916	Human	New Zealand	Campylobacter jejuni	583	ST-45 complex	https://www.ncbi.nlm.nih.gov/sra/SRR17929584
Ej_SC1028	2018	SRR17929583	PRJNA675916	Human	New Zealand	Campylobacter jejuni	4337	ST-21 complex	https://www.ncbi.nlm.nih.gov/sra/SRR17929583
Ej_SC1029	2018	SRR17929582	PRJNA675916	Human	New Zealand	Campylobacter jejuni	nJ06		https://www.ncbi.nlm.nih.gov/sra/SRR17929582
Ej_SC1030	2018	SRR17929581	PRJNA675916	Human	New Zealand	Campylobacter jejuni	nJ04		https://www.ncbi.nlm.nih.gov/sra/SRR17929581
Ej_SC1031	2018	SRR17929579	PRJNA675916	Human	New Zealand	Campylobacter jejuni	227	ST-206 complex	https://www.ncbi.nlm.nih.gov/sra/SRR17929579
Ej_SC1032	2018	SRR17929578	PRJNA675916	Human	New Zealand	Campylobacter jejuni	25	ST-45 complex	https://www.ncbi.nlm.nih.gov/sra/SRR17929578
Ej_SC1033	2018	SRR17929577	PRJNA675916	Human	New Zealand	Campylobacter jejuni	4337	ST-21 complex	https://www.ncbi.nlm.nih.gov/sra/SRR17929577
Ej_SC1034	2018	SRR17929576	PRJNA675916	Human	New Zealand	Campylobacter jejuni	8658	ST-464 complex	https://www.ncbi.nlm.nih.gov/sra/SRR17929576
Ej_SC1035	2018	SRR17929575	PRJNA675916	Human	New Zealand	Campylobacter jejuni	48	ST-48 complex	https://www.ncbi.nlm.nih.gov/sra/SRR17929575
Ej_SC1036	2018	SRR17929574	PRJNA675916	Human	New Zealand	Campylobacter jejuni	4337	ST-21 complex	https://www.ncbi.nlm.nih.gov/sra/SRR17929574
Ej_SC1037	2018	SRR17929573	PRJNA675916	Human	New Zealand	Campylobacter jejuni	45	ST-45 complex	https://www.ncbi.nlm.nih.gov/sra/SRR17929573
Ej_SC1038	2018	SRR17929572	PRJNA675916	Human	New Zealand	Campylobacter jejuni	45	ST-45 complex	https://www.ncbi.nlm.nih.gov/sra/SRR17929572
Ej_SC1039	2018	SRR17929571	PRJNA675916	Human	New Zealand	Campylobacter jejuni	4337	ST-21 complex	https://www.ncbi.nlm.nih.gov/sra/SRR17929571
Ej_SC1041	2018	SRR17929570	PRJNA675916	Human	New Zealand	Campylobacter jejuni	677	ST-677 complex	https://www.ncbi.nlm.nih.gov/sra/SRR17929570
Ej_SC1042	2018	SRR17929630	PRJNA675916	Human	New Zealand	Campylobacter jejuni	48	ST-48 complex	https://www.ncbi.nlm.nih.gov/sra/SRR17929630
Ej_SC1043	2018	SRR17929629	PRJNA675916	Human	New Zealand	Campylobacter jejuni	190	ST-21 complex	https://www.ncbi.nlm.nih.gov/sra/SRR17929629
Ej_SC1044	2018	SRR17929628	PRJNA675916	Human	New Zealand	Campylobacter jejuni	474	ST-48 complex	https://www.ncbi.nlm.nih.gov/sra/SRR17929628
Ej_SC1046	2018	SRR17929627	PRJNA675916	Human	New Zealand	Campylobacter jejuni	583	ST-45 complex	https://www.ncbi.nlm.nih.gov/sra/SRR17929627
Ej_SC1047	2018	SRR17929626	PRJNA675916	Human	New Zealand	Campylobacter jejuni	nJ03		https://www.ncbi.nlm.nih.gov/sra/SRR17929626
Ej_SC1056	2018	SRR17929625	PRJNA675916	Human	New Zealand	Campylobacter jejuni	25	ST-45 complex	https://www.ncbi.nlm.nih.gov/sra/SRR17929625
Ej_SC1057	2018	SRR17929624	PRJNA675916	Human	New Zealand	Campylobacter jejuni	4337	ST-21 complex	https://www.ncbi.nlm.nih.gov/sra/SRR17929624
Ej_SC1058	2018	SRR17929623	PRJNA675916	Human	New Zealand	Campylobacter jejuni	48	ST-48 complex	https://www.ncbi.nlm.nih.gov/sra/SRR17929623
Ej_SC1066	2018	SRR17929615	PRJNA675916	Human	New Zealand	Campylobacter jejuni	25	ST-45 complex	https://www.ncbi.nlm.nih.gov/sra/SRR17929615
Ej_SC1067	2018	SRR17929614	PRJNA675916	Human	New Zealand	Campylobacter jejuni	6964	ST-354 complex	https://www.ncbi.nlm.nih.gov/sra/SRR17929614
Ej_SC1069	2018	SRR17929613	PRJNA675916	Human	New Zealand	Campylobacter jejuni	45	ST-45 complex	https://www.ncbi.nlm.nih.gov/sra/SRR17929613
Ej_SC1071	2018	SRR17929612	PRJNA675916	Human	New Zealand	Campylobacter jejuni	45	ST-45 complex	https://www.ncbi.nlm.nih.gov/sra/SRR17929612
Ej_SC1072	2018	SRR17929611	PRJNA675916	Human	New Zealand	Campylobacter jejuni	677	ST-677 complex	https://www.ncbi.nlm.nih.gov/sra/SRR17929611
Ej_SC1076	2018	SRR17929608	PRJNA675916	Human	New Zealand	Campylobacter jejuni	677	ST-677 complex	https://www.ncbi.nlm.nih.gov/sra/SRR17929608
Ej_SC1079	2018	SRR17929607	PRJNA675916	Human	New Zealand	Campylobacter jejuni	45	ST-45 complex	https://www.ncbi.nlm.nih.gov/sra/SRR17929607
Ej_SC1081	2018	SRR17929606	PRJNA675916	Human	New Zealand	Campylobacter jejuni	45	ST-45 complex	https://www.ncbi.nlm.nih.gov/sra/SRR17929606
Ej_SC1082	2018	SRR17929605	PRJNA675916	Human	New Zealand	Campylobacter jejuni	583	ST-45 complex	https://www.ncbi.nlm.nih.gov/sra/SRR17929605
Ej_SC1086	2018	SRR17929604	PRJNA675916	Human	New Zealand	Campylobacter jejuni	2345	ST-206 complex	https://www.ncbi.nlm.nih.gov/sra/SRR17929604
Ej_SC1087	2018	SRR17929603	PRJNA675916	Human	New Zealand	Campylobacter jejuni	45	ST-45 complex	https://www.ncbi.nlm.nih.gov/sra/SRR17929603
Ej_SC1088	2018	SRR17929602	PRJNA675916	Human	New Zealand	Campylobacter jejuni	45	ST-45 complex	https://www.ncbi.nlm.nih.gov/sra/SRR17929602
Ej_SC1089	2018	SRR17929601	PRJNA675916	Human	New Zealand	Campylobacter jejuni	53	ST-21 complex	https://www.ncbi.nlm.nih.gov/sra/SRR17929601
Ej_SC1090	2018	SRR17965094	PRJNA675916	Human	New Zealand	Campylobacter jejuni	45	ST-45 complex	https://www.ncbi.nlm.nih.gov/sra/SRR17965094
Ej_SC1091	2018	SRR17965093	PRJNA675916	Human	New Zealand	Campylobacter jejuni	45	ST-45 complex	https://www.ncbi.nlm.nih.gov/sra/SRR17965093
Ej_SC1092	2018	SRR17964950	PRJNA675916	Human	New Zealand	Campylobacter jejuni	2345	ST-206 complex	https://www.ncbi.nlm.nih.gov/sra/SRR17964950
Ej_SC1094	2018	SRR17964912	PRJNA675916	Human	New Zealand	Campylobacter jejuni	45	ST-45 complex	https://www.ncbi.nlm.nih.gov/sra/SRR17964912
Ej_SC1098	2018	SRR17964900	PRJNA675916	Human	New Zealand	Campylobacter jejuni	53	ST-21 complex	https://www.ncbi.nlm.nih.gov/sra/SRR17964900
Ej_SC1099	2018	SRR17964889	PRJNA675916	Human	New Zealand	Campylobacter jejuni	45	ST-45 complex	https://www.ncbi.nlm.nih.gov/sra/SRR17964889
Ej_SC1100	2018	SRR17964878	PRJNA675916	Chicken	New Zealand	Campylobacter jejuni	677	ST-677 complex	https://www.ncbi.nlm.nih.gov/sra/SRR17964878
Ej_SC1101	2018	SRR17965092	PRJNA675916	Chicken	New Zealand	Campylobacter jejuni	48	ST-48 complex	https://www.ncbi.nlm.nih.gov/sra/SRR17965092
Ej_SC1102	2018	SRR17965081	PRJNA675916	Chicken	New Zealand	Campylobacter jejuni	45	ST-45 complex	https://www.ncbi.nlm.nih.gov/sra/SRR17965081
Ej_SC1103	2018	SRR17965070	PRJNA675916	Human	New Zealand	Campylobacter jejuni	45	ST-45 complex	https://www.ncbi.nlm.nih.gov/sra/SRR17965070
Ej_SC1104	2018	SRR17965059	PRJNA675916	Human	New Zealand	Campylobacter jejuni	2535		https://www.ncbi.nlm.nih.gov/sra/SRR17965059
Ej_SC1108	2018	SRR17965048	PRJNA675916	Human	New Zealand	Campylobacter jejuni	48	ST-48 complex	https://www.ncbi.nlm.nih.gov/sra/SRR17965048
Ej_SC1110	2018	SRR17965037	PRJNA675916	Human	New Zealand	Campylobacter jejuni	45	ST-45 complex	https://www.ncbi.nlm.nih.gov/sra/SRR17965037
Ej_SC1111	2018	SRR17965026	PRJNA675916	Human	New Zealand	Campylobacter jejuni	25	ST-45 complex	https://www.ncbi.nlm.nih.gov/sra/SRR17965026

Isolate no.	Year	Accession no.	BioProject no.	Source	Country	Species	Sequence type	Clonal complex	SRA hyperlink
Ej_SC1113	2018	SRR17965015	PRJNA675916	Human	New Zealand	Campylobacter jejuni	45	ST-45 complex	https://www.ncbi.nlm.nih.gov/sra/SRR17965015
Ej_SC1114	2018	SRR17965004	PRJNA675916	Human	New Zealand	Campylobacter jejuni	45	ST-45 complex	https://www.ncbi.nlm.nih.gov/sra/SRR17965004
Ej_SC1115	2018	SRR17964961	PRJNA675916	Human	New Zealand	Campylobacter jejuni	45	ST-45 complex	https://www.ncbi.nlm.nih.gov/sra/SRR17964961
Ej_SC1117	2018	SRR17964949	PRJNA675916	Human	New Zealand	Campylobacter jejuni	583	ST-45 complex	https://www.ncbi.nlm.nih.gov/sra/SRR17964949
Ej_SC1118	2018	SRR17964938	PRJNA675916	Human	New Zealand	Campylobacter jejuni	45	ST-45 complex	https://www.ncbi.nlm.nih.gov/sra/SRR17964938
Ej_SC1119	2018	SRR17964927	PRJNA675916	Human	New Zealand	Campylobacter jejuni	2345	ST-206 complex	https://www.ncbi.nlm.nih.gov/sra/SRR17964927
Ej_SC1121	2018	SRR17964919	PRJNA675916	Human	New Zealand	Campylobacter jejuni	257	ST-257 complex	https://www.ncbi.nlm.nih.gov/sra/SRR17964919
Ej_SC1122	2018	SRR17964918	PRJNA675916	Human	New Zealand	Campylobacter jejuni	137	ST-45 complex	https://www.ncbi.nlm.nih.gov/sra/SRR17964918
Ej_SC1123	2018	SRR17964917	PRJNA675916	Human	New Zealand	Campylobacter jejuni	583	ST-45 complex	https://www.ncbi.nlm.nih.gov/sra/SRR17964917
Ej_SC1125	2018	SRR17964916	PRJNA675916	Human	New Zealand	Campylobacter jejuni	583	ST-45 complex	https://www.ncbi.nlm.nih.gov/sra/SRR17964916
Ej_SC1126	2018	SRR17964915	PRJNA675916	Human	New Zealand	Campylobacter jejuni	677	ST-677 complex	https://www.ncbi.nlm.nih.gov/sra/SRR17964915
Ej_SC1127	2018	SRR17964914	PRJNA675916	Human	New Zealand	Campylobacter jejuni	nJ10		https://www.ncbi.nlm.nih.gov/sra/SRR17964914
Ej_SC1128	2018	SRR17964913	PRJNA675916	Human	New Zealand	Campylobacter jejuni	45	ST-45 complex	https://www.ncbi.nlm.nih.gov/sra/SRR17964913
Ej_SC1129	2018	SRR17964911	PRJNA675916	Human	New Zealand	Campylobacter jejuni	177	ST-177 complex	https://www.ncbi.nlm.nih.gov/sra/SRR17964911
Ej_SC1146	2018	SRR17964993	PRJNA675916	Human	New Zealand	Campylobacter jejuni	677	ST-677 complex	https://www.ncbi.nlm.nih.gov/sra/SRR17964993
Ej_SC1147	2018	SRR17964992	PRJNA675916	Human	New Zealand	Campylobacter jejuni	2345	ST-206 complex	https://www.ncbi.nlm.nih.gov/sra/SRR17964992
Ej_SC1148	2018	SRR17964991	PRJNA675916	Human	New Zealand	Campylobacter jejuni	6964	ST-354 complex	https://www.ncbi.nlm.nih.gov/sra/SRR17964991
Ej_SC1149	2018	SRR17964990	PRJNA675916	Human	New Zealand	Campylobacter jejuni	45	ST-45 complex	https://www.ncbi.nlm.nih.gov/sra/SRR17964990
Ej_SC1150	2018	SRR17964989	PRJNA675916	Human	New Zealand	Campylobacter jejuni	583	ST-45 complex	https://www.ncbi.nlm.nih.gov/sra/SRR17964989
Ej_SC1151	2018	SRR17964988	PRJNA675916	Human	New Zealand	Campylobacter jejuni	583	ST-45 complex	https://www.ncbi.nlm.nih.gov/sra/SRR17964988
Ej_SC1152	2018	SRR17964987	PRJNA675916	Human	New Zealand	Campylobacter jejuni	677	ST-677 complex	https://www.ncbi.nlm.nih.gov/sra/SRR17964987
Ej_SC1153	2018	SRR17964985	PRJNA675916	Human	New Zealand	Campylobacter jejuni	583	ST-45 complex	https://www.ncbi.nlm.nih.gov/sra/SRR17964985
Ej_SC1155	2018	SRR17964984	PRJNA675916	Human	New Zealand	Campylobacter jejuni	45	ST-45 complex	https://www.ncbi.nlm.nih.gov/sra/SRR17964984
Ej_SC1156	2018	SRR17964983	PRJNA675916	Human	New Zealand	Campylobacter jejuni	677	ST-677 complex	https://www.ncbi.nlm.nih.gov/sra/SRR17964983
Ej_SC1157	2018	SRR17964982	PRJNA675916	Human	New Zealand	Campylobacter jejuni	27	ST-362 complex	https://www.ncbi.nlm.nih.gov/sra/SRR17964982
Ej_SC1160	2018	SRR17964981	PRJNA675916	Human	New Zealand	Campylobacter jejuni	583	ST-45 complex	https://www.ncbi.nlm.nih.gov/sra/SRR17964981
Ej_SC1161	2018	SRR17964980	PRJNA675916	Human	New Zealand	Campylobacter jejuni	4492	ST-61 complex	https://www.ncbi.nlm.nih.gov/sra/SRR17964980
Ej_SC1163	2018	SRR17964978	PRJNA675916	Human	New Zealand	Campylobacter jejuni	45	ST-45 complex	https://www.ncbi.nlm.nih.gov/sra/SRR17964978
Ej_SC1164	2018	SRR17964977	PRJNA675916	Human	New Zealand	Campylobacter jejuni	45	ST-45 complex	https://www.ncbi.nlm.nih.gov/sra/SRR17964977
Ej_SC1165	2018	SRR17964976	PRJNA675916	Human	New Zealand	Campylobacter jejuni	2535		https://www.ncbi.nlm.nih.gov/sra/SRR17964976
Ej_SC1166	2018	SRR17964974	PRJNA675916	Human	New Zealand	Campylobacter jejuni	3712	ST-362 complex	https://www.ncbi.nlm.nih.gov/sra/SRR17964974
Ej_SC1169	2018	SRR17964973	PRJNA675916	Human	New Zealand	Campylobacter jejuni	583	ST-45 complex	https://www.ncbi.nlm.nih.gov/sra/SRR17964973
Ej_SC1170	2018	SRR17964972	PRJNA675916	Human	New Zealand	Campylobacter jejuni	422	ST-21 complex	https://www.ncbi.nlm.nih.gov/sra/SRR17964972
Ej_SC1173	2018	SRR17964971	PRJNA675916	Human	New Zealand	Campylobacter jejuni	45	ST-45 complex	https://www.ncbi.nlm.nih.gov/sra/SRR17964971
Ej_SC1176	2018	SRR17964906	PRJNA675916	Human	New Zealand	Campylobacter jejuni	583	ST-45 complex	https://www.ncbi.nlm.nih.gov/sra/SRR17964906
Ej_SC1180	2018	SRR17964905	PRJNA675916	Human	New Zealand	Campylobacter jejuni	45	ST-45 complex	https://www.ncbi.nlm.nih.gov/sra/SRR17964905
Ej_SC1181	2018	SRR17964904	PRJNA675916	Chicken	New Zealand	Campylobacter jejuni	50	ST-21 complex	https://www.ncbi.nlm.nih.gov/sra/SRR17964904
Ej_SC1182	2018	SRR17964903	PRJNA675916	Chicken	New Zealand	Campylobacter jejuni	53	ST-21 complex	https://www.ncbi.nlm.nih.gov/sra/SRR17964903
Ej_SC1183	2018	SRR17964902	PRJNA675916	Human	New Zealand	Campylobacter jejuni	22	ST-22 complex	https://www.ncbi.nlm.nih.gov/sra/SRR17964902
Ej_SC1185	2018	SRR17964901	PRJNA675916	Human	New Zealand	Campylobacter jejuni	45	ST-45 complex	https://www.ncbi.nlm.nih.gov/sra/SRR17964901
Ej_SC1188	2018	SRR17964899	PRJNA675916	Human	New Zealand	Campylobacter jejuni	53	ST-21 complex	https://www.ncbi.nlm.nih.gov/sra/SRR17964899
Ej_SC1189	2018	SRR17964898	PRJNA675916	Human	New Zealand	Campylobacter jejuni	677	ST-677 complex	https://www.ncbi.nlm.nih.gov/sra/SRR17964898
Ej_SC1190	2018	SRR17964897	PRJNA675916	Human	New Zealand	Campylobacter jejuni	45	ST-45 complex	https://www.ncbi.nlm.nih.gov/sra/SRR17964897
Ej_SC1191	2018	SRR17964896	PRJNA675916	Human	New Zealand	Campylobacter jejuni	51	ST-443 complex	https://www.ncbi.nlm.nih.gov/sra/SRR17964896
Ej_SC1194	2018	SRR17964894	PRJNA675916	Human	New Zealand	Campylobacter jejuni	2345	ST-206 complex	https://www.ncbi.nlm.nih.gov/sra/SRR17964894
Ej_SC1196	2018	SRR17964893	PRJNA675916	Human	New Zealand	Campylobacter jejuni	677	ST-677 complex	https://www.ncbi.nlm.nih.gov/sra/SRR17964893
Ej_SC1197	2018	SRR17964892	PRJNA675916	Human	New Zealand	Campylobacter jejuni	677	ST-677 complex	https://www.ncbi.nlm.nih.gov/sra/SRR17964892
Ej_SC1213	2018	SRR17964876	PRJNA675916	Human	New Zealand	Campylobacter jejuni	45	ST-45 complex	https://www.ncbi.nlm.nih.gov/sra/SRR17964876
Ej_SC1215	2018	SRR17964875	PRJNA675916	Human	New Zealand	Campylobacter jejuni	45	ST-45 complex	https://www.ncbi.nlm.nih.gov/sra/SRR17964875
Ej_SC1218	2018	SRR17964874	PRJNA675916	Human	New Zealand	Campylobacter jejuni	45	ST-45 complex	https://www.ncbi.nlm.nih.gov/sra/SRR17964874
Ej_SC1219	2018	SRR17964873	PRJNA675916	Human	New Zealand	Campylobacter jejuni	508	ST-508 complex	https://www.ncbi.nlm.nih.gov/sra/SRR17964873
Ej_SC1222	2018	SRR17964872	PRJNA675916	Human	New Zealand	Campylobacter jejuni	583	ST-45 complex	https://www.ncbi.nlm.nih.gov/sra/SRR17964872
Ej_SC1223	2018	SRR17964871	PRJNA675916	Human	New Zealand	Campylobacter jejuni	677	ST-677 complex	https://www.ncbi.nlm.nih.gov/sra/SRR17964871
Ej_SC1225	2018	SRR17964870	PRJNA675916	Human	New Zealand	Campylobacter jejuni	538	ST-45 complex	https://www.ncbi.nlm.nih.gov/sra/SRR17964870
Ej_SC1226	2018	SRR17964869	PRJNA675916	Human	New Zealand	Campylobacter jejuni	45	ST-45 complex	https://www.ncbi.nlm.nih.gov/sra/SRR17964869
Ej_SC1227	2018	SRR17964868	PRJNA675916	Human	New Zealand	Campylobacter jejuni	45	ST-45 complex	https://www.ncbi.nlm.nih.gov/sra/SRR17964868
Ej_SC1228	2018	SRR17965091	PRJNA675916	Human	New Zealand	Campylobacter jejuni	436		https://www.ncbi.nlm.nih.gov/sra/SRR17965091
Ej_SC1230	2018	SRR17965090	PRJNA675916	Human	New Zealand	Campylobacter jejuni	583	ST-45 complex	https://www.ncbi.nlm.nih.gov/sra/SRR17965090
Ej_SC1231	2018	SRR17965089	PRJNA675916	Human	New Zealand	Campylobacter jejuni	583	ST-45 complex	https://www.ncbi.nlm.nih.gov/sra/SRR17965089
Ej_SC1233	2018	SRR17965088	PRJNA675916	Human	New Zealand	Campylobacter jejuni	45	ST-45 complex	https://www.ncbi.nlm.nih.gov/sra/SRR17965088
Ej_SC1234	2018	SRR17965087	PRJNA675916	Human	New Zealand	Campylobacter jejuni	45	ST-45 complex	https://www.ncbi.nlm.nih.gov/sra/SRR17965087
Ej_SC1235	2018	SRR17965086	PRJNA675916	Human	New Zealand	Campylobacter jejuni	45	ST-45 complex	https://www.ncbi.nlm.nih.gov/sra/SRR17965086
Ej_SC1236	2018	SRR17965085	PRJNA675916	Chicken	New Zealand	Campylobacter jejuni	190	ST-21 complex	https://www.ncbi.nlm.nih.gov/sra/SRR17965085
Ej_SC1237	2018	SRR17965084	PRJNA675916	Chicken	New Zealand	Campylobacter jejuni	6964	ST-354 complex	https://www.ncbi.nlm.nih.gov/sra/SRR17965084
Ej_SC1238	2018	SRR17965083	PRJNA675916	Chicken	New Zealand	Campylobacter jejuni	53	ST-21 complex	https://www.ncbi.nlm.nih.gov/sra/SRR17965083
Ej_SC1239	2018	SRR17965082	PRJNA675916	Chicken	New Zealand	Campylobacter jejuni	48	ST-48 complex	https://www.ncbi.nlm.nih.gov/sra/SRR17965082
Ej_SC1240	2018	SRR17965080	PRJNA675916	Chicken	New Zealand	Campylobacter jejuni	45	ST-45 complex	https://www.ncbi.nlm.nih.gov/sra/SRR17965080
Ej_SC1241	2018	SRR17965079	PRJNA675916	Human	New Zealand	Campylobacter jejuni	45	ST-45 complex	https://www.ncbi.nlm.nih.gov/sra/SRR17965079
Ej_SC1242	2018	SRR17965078	PRJNA675916	Human	New Zealand	Campylobacter jejuni	677	ST-677 complex	https://www.ncbi.nlm.nih.gov/sra/SRR17965078
Ej_SC1243	2018	SRR17965077	PRJNA675916	Human	New Zealand	Campylobacter jejuni	583	ST-45 complex	https://www.ncbi.nlm.nih.gov/sra/SRR17965077
Ej_SC1244	2018	SRR17965076	PRJNA675916	Human	New Zealand	Campylobacter jejuni	45	ST-45 complex	https://www.ncbi.nlm.nih.gov/sra/SRR17965076
Ej_SC1245	2018	SRR17965075	PRJNA675916	Human	New Zealand	Campylobacter jejuni	677	ST-677 complex	https://www.ncbi.nlm.nih.gov/sra/SRR17965075
Ej_SC1247	2018	SRR17965074	PRJNA675916	Human	New Zealand	Campylobacter jejuni	45	ST-45 complex	https://www.ncbi.nlm.nih.gov/sra/SRR17965074
Ej_SC1248	2018	SRR17965073	PRJNA675916	Human	New Zealand	Campylobacter jejuni	451	ST-21 complex	https://www.ncbi.nlm.nih.gov/sra/SRR17965073
Ej_SC1249	2018	SRR17965072	PRJNA675916	Human	New Zealand	Campylobacter jejuni	45	ST-45 complex	https://www.ncbi.nlm.nih.gov/sra/SRR17965072
Ej_SC1250	2018	SRR17965071	PRJNA675916	Human	New Zealand	Campylobacter jejuni	45	ST-45 complex	https://www.ncbi.nlm.nih.gov/sra/SRR17965071
Ej_SC1251	2018	SRR17965069	PRJNA675916	Human	New Zealand	Campylobacter jejuni	4337	ST-21 complex	https://www.ncbi.nlm.nih.gov/sra/SRR17965069
Ej_SC1252	2018	SRR17965068	PRJNA675916	Human	New Zealand	Campylobacter jejuni	583	ST-45 complex	https://www.ncbi.nlm.nih.gov/sra/SRR17965068
Ej_SC1253	2018	SRR17965067	PRJNA675916	Human	New Zealand	Campylobacter jejuni	45	ST-45 complex	https://www.ncbi.nlm.nih.gov/sra/SRR17965067
Ej_SC1254	2018	SRR17965066	PRJNA675916	Human	New Zealand	Campylobacter jejuni	51	ST-443 complex	https://www.ncbi.nlm.nih.gov/sra/SRR17965066
Ej_SC1255	2018	SRR17965065	PRJNA675916	Human	New Zealand	Campylobacter jejuni	2345	ST-206 complex	https://www.ncbi.nlm.nih.gov/sra/SRR17965065
Ej_SC1256	2018	SRR17965064	PRJNA675916	Human	New Zealand	Campylobacter jejuni	45	ST-45 complex	https://www.ncbi.nlm.nih.gov/sra/SRR17965064
Ej_SC1257	2018	SRR17965063	PRJNA675916	Human	New Zealand	Campylobacter jejuni	4337	ST-21 complex	https://www.ncbi.nlm.nih.gov/sra/SRR17965063
Ej_SC1259	2018	SRR17965062	PRJNA675916	Human	New Zealand	Campylobacter jejuni	48	ST-48 complex	https://www.ncbi.nlm.nih.gov/sra/SRR17965062
Ej_SC1260	2018	SRR17965061	PRJNA675916	Human	New Zealand	Campylobacter jejuni	474	ST-48 complex	https://www.ncbi.nlm.nih.gov/sra/SRR17965061
Ej_SC1263	2018	SRR17965060	PRJNA675916	Human	New Zealand	Campylobacter jejuni	45	ST-45 complex	https://www.ncbi.nlm.nih.gov/sra/SRR17965060
Ej_SC1264	2018	SRR17965058	PRJNA675916	Human	New Zealand	Campylobacter jejuni	45	ST-45 complex	https://www.ncbi.nlm.nih.gov/sra/SRR17965058

Isolate no.	Year	Accession no.	BioProject no.	Source	Country	Species	Sequence type	Clonal complex	SRA hyperlink
Ej_SC1266	2018	SRR17965057	PRJNA675916	Human	New Zealand	Campylobacter jejuni	48	ST-48 complex	https://www.ncbi.nlm.nih.gov/sra/SRR17965057
Ej_SC1267	2018	SRR17965056	PRJNA675916	Human	New Zealand	Campylobacter jejuni	48	ST-48 complex	https://www.ncbi.nlm.nih.gov/sra/SRR17965056
Ej_SC1268	2018	SRR17965055	PRJNA675916	Human	New Zealand	Campylobacter jejuni	677	ST-677 complex	https://www.ncbi.nlm.nih.gov/sra/SRR17965055
Ej_SC1269	2018	SRR17965054	PRJNA675916	Human	New Zealand	Campylobacter jejuni	677	ST-677 complex	https://www.ncbi.nlm.nih.gov/sra/SRR17965054
Ej_SC1271	2018	SRR17965053	PRJNA675916	Human	New Zealand	Campylobacter jejuni	45	ST-45 complex	https://www.ncbi.nlm.nih.gov/sra/SRR17965053
Ej_SC1272	2018	SRR17965052	PRJNA675916	Human	New Zealand	Campylobacter jejuni	5647	ST-692 complex	https://www.ncbi.nlm.nih.gov/sra/SRR17965052
Ej_SC1273	2018	SRR17965051	PRJNA675916	Human	New Zealand	Campylobacter jejuni	583	ST-45 complex	https://www.ncbi.nlm.nih.gov/sra/SRR17965051
Ej_SC1274	2018	SRR17965050	PRJNA675916	Human	New Zealand	Campylobacter jejuni	583	ST-45 complex	https://www.ncbi.nlm.nih.gov/sra/SRR17965050
Ej_SC1275	2018	SRR17965049	PRJNA675916	Human	New Zealand	Campylobacter jejuni	53	ST-21 complex	https://www.ncbi.nlm.nih.gov/sra/SRR17965049
Ej_SC1276	2018	SRR17965047	PRJNA675916	Human	New Zealand	Campylobacter jejuni	45	ST-45 complex	https://www.ncbi.nlm.nih.gov/sra/SRR17965047
Ej_SC1278	2018	SRR17965046	PRJNA675916	Human	New Zealand	Campylobacter jejuni	538	ST-45 complex	https://www.ncbi.nlm.nih.gov/sra/SRR17965046
Ej_SC1279	2018	SRR17965045	PRJNA675916	Human	New Zealand	Campylobacter jejuni	45	ST-45 complex	https://www.ncbi.nlm.nih.gov/sra/SRR17965045
Ej_SC1280	2018	SRR17965044	PRJNA675916	Human	New Zealand	Campylobacter jejuni	45	ST-45 complex	https://www.ncbi.nlm.nih.gov/sra/SRR17965044
Ej_SC1281	2018	SRR17965043	PRJNA675916	Human	New Zealand	Campylobacter jejuni	38	ST-48 complex	https://www.ncbi.nlm.nih.gov/sra/SRR17965043
Ej_SC1286	2018	SRR17965041	PRJNA675916	Human	New Zealand	Campylobacter jejuni	45	ST-45 complex	https://www.ncbi.nlm.nih.gov/sra/SRR17965041
Ej_SC1288	2018	SRR17965040	PRJNA675916	Human	New Zealand	Campylobacter jejuni	583	ST-45 complex	https://www.ncbi.nlm.nih.gov/sra/SRR17965040
Ej_SC1289	2018	SRR17965039	PRJNA675916	Human	New Zealand	Campylobacter jejuni	nJ01		https://www.ncbi.nlm.nih.gov/sra/SRR17965039
Ej_SC1292	2018	SRR17965038	PRJNA675916	Human	New Zealand	Campylobacter jejuni	48	ST-48 complex	https://www.ncbi.nlm.nih.gov/sra/SRR17965038
Ej_SC1296	2018	SRR17965036	PRJNA675916	Human	New Zealand	Campylobacter jejuni	45	ST-45 complex	https://www.ncbi.nlm.nih.gov/sra/SRR17965036
Ej_SC1298	2018	SRR17965035	PRJNA675916	Human	New Zealand	Campylobacter jejuni	53	ST-21 complex	https://www.ncbi.nlm.nih.gov/sra/SRR17965035
Ej_SC1302	2018	SRR17965034	PRJNA675916	Human	New Zealand	Campylobacter jejuni	53	ST-21 complex	https://www.ncbi.nlm.nih.gov/sra/SRR17965034
Ej_SC1304	2018	SRR17965033	PRJNA675916	Human	New Zealand	Campylobacter jejuni	48	ST-48 complex	https://www.ncbi.nlm.nih.gov/sra/SRR17965033
Ej_SC1306	2018	SRR17965032	PRJNA675916	Human	New Zealand	Campylobacter jejuni	53	ST-21 complex	https://www.ncbi.nlm.nih.gov/sra/SRR17965032
Ej_SC1314	2018	SRR17965030	PRJNA675916	Chicken	New Zealand	Campylobacter jejuni	53	ST-21 complex	https://www.ncbi.nlm.nih.gov/sra/SRR17965030
Ej_SC1315	2018	SRR17965029	PRJNA675916	Chicken	New Zealand	Campylobacter jejuni	257	ST-257 complex	https://www.ncbi.nlm.nih.gov/sra/SRR17965029
Ej_SC1316	2018	SRR17965028	PRJNA675916	Chicken	New Zealand	Campylobacter jejuni	50	ST-21 complex	https://www.ncbi.nlm.nih.gov/sra/SRR17965028
Ej_SC1317	2019	SRR17965027	PRJNA675916	Chicken	New Zealand	Campylobacter jejuni	45	ST-45 complex	https://www.ncbi.nlm.nih.gov/sra/SRR17965027
Ej_SC1318	2019	SRR17965025	PRJNA675916	Chicken	New Zealand	Campylobacter jejuni	48	ST-48 complex	https://www.ncbi.nlm.nih.gov/sra/SRR17965025
Ej_SC1320	2019	SRR17965023	PRJNA675916	Chicken	New Zealand	Campylobacter jejuni	48	ST-48 complex	https://www.ncbi.nlm.nih.gov/sra/SRR17965023
Ej_SC1321	2019	SRR17965022	PRJNA675916	Chicken	New Zealand	Campylobacter jejuni	48	ST-48 complex	https://www.ncbi.nlm.nih.gov/sra/SRR17965022
Ej_SC1322	2019	SRR17965021	PRJNA675916	Chicken	New Zealand	Campylobacter jejuni	45	ST-45 complex	https://www.ncbi.nlm.nih.gov/sra/SRR17965021
Ej_SC1323	2019	SRR17965020	PRJNA675916	Chicken	New Zealand	Campylobacter jejuni	48	ST-48 complex	https://www.ncbi.nlm.nih.gov/sra/SRR17965020
Ej_SC1324	2019	SRR17965019	PRJNA675916	Chicken	New Zealand	Campylobacter jejuni	48	ST-48 complex	https://www.ncbi.nlm.nih.gov/sra/SRR17965019
Ej_SC1326	2019	SRR17965017	PRJNA675916	Chicken	New Zealand	Campylobacter jejuni	53	ST-21 complex	https://www.ncbi.nlm.nih.gov/sra/SRR17965017
Ej_SC1327	2019	SRR17965016	PRJNA675916	Chicken	New Zealand	Campylobacter jejuni	53	ST-21 complex	https://www.ncbi.nlm.nih.gov/sra/SRR17965016
Ej_SC1328	2019	SRR17965014	PRJNA675916	Chicken	New Zealand	Campylobacter jejuni	45	ST-45 complex	https://www.ncbi.nlm.nih.gov/sra/SRR17965014
Ej_SC1329	2019	SRR17965013	PRJNA675916	Chicken	New Zealand	Campylobacter jejuni	2345	ST-206 complex	https://www.ncbi.nlm.nih.gov/sra/SRR17965013
Ej_SC1330	2019	SRR17965012	PRJNA675916	Chicken	New Zealand	Campylobacter jejuni	4337	ST-21 complex	https://www.ncbi.nlm.nih.gov/sra/SRR17965012
Ej_SC1331	2019	SRR17965011	PRJNA675916	Human	New Zealand	Campylobacter jejuni	583	ST-45 complex	https://www.ncbi.nlm.nih.gov/sra/SRR17965011
Ej_SC1334	2019	SRR17965010	PRJNA675916	Human	New Zealand	Campylobacter jejuni	45	ST-45 complex	https://www.ncbi.nlm.nih.gov/sra/SRR17965010
Ej_SC1335	2019	SRR17965009	PRJNA675916	Human	New Zealand	Campylobacter jejuni	45	ST-45 complex	https://www.ncbi.nlm.nih.gov/sra/SRR17965009
Ej_SC1336	2019	SRR17965008	PRJNA675916	Human	New Zealand	Campylobacter jejuni	583	ST-45 complex	https://www.ncbi.nlm.nih.gov/sra/SRR17965008
Ej_SC1337	2019	SRR17965007	PRJNA675916	Human	New Zealand	Campylobacter jejuni	538	ST-45 complex	https://www.ncbi.nlm.nih.gov/sra/SRR17965007
Ej_SC1341	2019	SRR17965006	PRJNA675916	Human	New Zealand	Campylobacter jejuni	53	ST-21 complex	https://www.ncbi.nlm.nih.gov/sra/SRR17965006
Ej_SC1344	2019	SRR17965005	PRJNA675916	Human	New Zealand	Campylobacter jejuni	4337	ST-21 complex	https://www.ncbi.nlm.nih.gov/sra/SRR17965005
Ej_SC1345	2019	SRR17965003	PRJNA675916	Human	New Zealand	Campylobacter jejuni	583	ST-45 complex	https://www.ncbi.nlm.nih.gov/sra/SRR17965003
Ej_SC1346	2019	SRR17964970	PRJNA675916	Human	New Zealand	Campylobacter jejuni	2026	ST-403 complex	https://www.ncbi.nlm.nih.gov/sra/SRR17964970
Ej_SC1347	2019	SRR17964969	PRJNA675916	Human	New Zealand	Campylobacter jejuni	50	ST-21 complex	https://www.ncbi.nlm.nih.gov/sra/SRR17964969
Ej_SC1348	2019	SRR17964968	PRJNA675916	Human	New Zealand	Campylobacter jejuni	583	ST-45 complex	https://www.ncbi.nlm.nih.gov/sra/SRR17964968
Ej_SC1350	2019	SRR17964967	PRJNA675916	Human	New Zealand	Campylobacter jejuni	50	ST-21 complex	https://www.ncbi.nlm.nih.gov/sra/SRR17964967
Ej_SC1351	2019	SRR17964966	PRJNA675916	Human	New Zealand	Campylobacter jejuni	45	ST-45 complex	https://www.ncbi.nlm.nih.gov/sra/SRR17964966
Ej_SC1353	2019	SRR17964965	PRJNA675916	Human	New Zealand	Campylobacter jejuni	45	ST-45 complex	https://www.ncbi.nlm.nih.gov/sra/SRR17964965
Ej_SC1354	2019	SRR17964964	PRJNA675916	Human	New Zealand	Campylobacter jejuni	583	ST-45 complex	https://www.ncbi.nlm.nih.gov/sra/SRR17964964
Ej_SC1356	2019	SRR17964963	PRJNA675916	Human	New Zealand	Campylobacter jejuni	3712	ST-362 complex	https://www.ncbi.nlm.nih.gov/sra/SRR17964963
Ej_SC1357	2019	SRR17964962	PRJNA675916	Human	New Zealand	Campylobacter jejuni	6964	ST-354 complex	https://www.ncbi.nlm.nih.gov/sra/SRR17964962
Ej_SC1361	2019	SRR17964960	PRJNA675916	Human	New Zealand	Campylobacter jejuni	583	ST-45 complex	https://www.ncbi.nlm.nih.gov/sra/SRR17964960
Ej_SC1363	2019	SRR17964959	PRJNA675916	Human	New Zealand	Campylobacter jejuni	45	ST-45 complex	https://www.ncbi.nlm.nih.gov/sra/SRR17964959
Ej_SC1364	2019	SRR17964958	PRJNA675916	Human	New Zealand	Campylobacter jejuni	583	ST-45 complex	https://www.ncbi.nlm.nih.gov/sra/SRR17964958
Ej_SC1365	2019	SRR17964957	PRJNA675916	Human	New Zealand	Campylobacter jejuni	45	ST-45 complex	https://www.ncbi.nlm.nih.gov/sra/SRR17964957
Ej_SC1366	2019	SRR17964956	PRJNA675916	Human	New Zealand	Campylobacter jejuni	583	ST-45 complex	https://www.ncbi.nlm.nih.gov/sra/SRR17964956
Ej_SC1378	2019	SRR17964946	PRJNA675916	Human	New Zealand	Campylobacter jejuni	422	ST-21 complex	https://www.ncbi.nlm.nih.gov/sra/SRR17964946
Ej_SC1381	2019	SRR17964944	PRJNA675916	Chicken	New Zealand	Campylobacter jejuni	50	ST-21 complex	https://www.ncbi.nlm.nih.gov/sra/SRR17964944
Ej_SC1383	2019	SRR17964943	PRJNA675916	Human	New Zealand	Campylobacter jejuni	677	ST-677 complex	https://www.ncbi.nlm.nih.gov/sra/SRR17964943
Ej_SC1384	2019	SRR17964942	PRJNA675916	Human	New Zealand	Campylobacter jejuni	6139	ST-42 complex	https://www.ncbi.nlm.nih.gov/sra/SRR17964942
Ej_SC1385	2019	SRR17964941	PRJNA675916	Human	New Zealand	Campylobacter jejuni	486	ST-21 complex	https://www.ncbi.nlm.nih.gov/sra/SRR17964941
Ej_SC1386	2019	SRR17964940	PRJNA675916	Human	New Zealand	Campylobacter jejuni	190	ST-21 complex	https://www.ncbi.nlm.nih.gov/sra/SRR17964940
Ej_SC1389	2019	SRR17964939	PRJNA675916	Human	New Zealand	Campylobacter jejuni	45	ST-45 complex	https://www.ncbi.nlm.nih.gov/sra/SRR17964939
Ej_SC1390	2019	SRR17964937	PRJNA675916	Human	New Zealand	Campylobacter jejuni	53	ST-21 complex	https://www.ncbi.nlm.nih.gov/sra/SRR17964937
Ej_SC1391	2019	SRR17964936	PRJNA675916	Human	New Zealand	Campylobacter jejuni	45	ST-45 complex	https://www.ncbi.nlm.nih.gov/sra/SRR17964936
Ej_SC1393	2019	SRR17964935	PRJNA675916	Human	New Zealand	Campylobacter jejuni	45	ST-45 complex	https://www.ncbi.nlm.nih.gov/sra/SRR17964935
Ej_SC1394	2019	SRR17964934	PRJNA675916	Human	New Zealand	Campylobacter jejuni	45	ST-45 complex	https://www.ncbi.nlm.nih.gov/sra/SRR17964934
Ej_SC1396	2019	SRR17964933	PRJNA675916	Human	New Zealand	Campylobacter jejuni	21	ST-21 complex	https://www.ncbi.nlm.nih.gov/sra/SRR17964933
Ej_SC1397	2019	SRR17964932	PRJNA675916	Human	New Zealand	Campylobacter jejuni	583	ST-45 complex	https://www.ncbi.nlm.nih.gov/sra/SRR17964932
Ej_SC1399	2019	SRR17964931	PRJNA675916	Human	New Zealand	Campylobacter jejuni	45	ST-45 complex	https://www.ncbi.nlm.nih.gov/sra/SRR17964931
Ej_SC1400	2019	SRR17964930	PRJNA675916	Human	New Zealand	Campylobacter jejuni	45	ST-45 complex	https://www.ncbi.nlm.nih.gov/sra/SRR17964930
Ej_SC1401	2019	SRR17964929	PRJNA675916	Human	New Zealand	Campylobacter jejuni	61	ST-61 complex	https://www.ncbi.nlm.nih.gov/sra/SRR17964929
Ej_SC1403	2019	SRR17964928	PRJNA675916	Human	New Zealand	Campylobacter jejuni	12755		https://www.ncbi.nlm.nih.gov/sra/SRR17964928
Ej_SC1405	2019	SRR17964926	PRJNA675916	Human	New Zealand	Campylobacter jejuni	53	ST-21 complex	https://www.ncbi.nlm.nih.gov/sra/SRR17964926
Ej_SC1406	2019	SRR17964925	PRJNA675916	Human	New Zealand	Campylobacter jejuni	190	ST-21 complex	https://www.ncbi.nlm.nih.gov/sra/SRR17964925
Ej_SC1407	2019	SRR17964924	PRJNA675916	Human	New Zealand	Campylobacter jejuni	2345	ST-206 complex	https://www.ncbi.nlm.nih.gov/sra/SRR17964924
Ej_SC1420	2019	SRR17974245	PRJNA675916	Chicken	New Zealand	Campylobacter jejuni	51	ST-443 complex	https://www.ncbi.nlm.nih.gov/sra/SRR17974245
Ej_SC1426	2019	SRR17974161	PRJNA675916	Human	New Zealand	Campylobacter jejuni	583	ST-45 complex	https://www.ncbi.nlm.nih.gov/sra/SRR17974161
Ej_SC1427	2019	SRR17974356	PRJNA675916	Human	New Zealand	Campylobacter jejuni	45	ST-45 complex	https://www.ncbi.nlm.nih.gov/sra/SRR17974356
Ej_SC1428	2019	SRR17974345	PRJNA675916	Human	New Zealand	Campylobacter jejuni	53	ST-21 complex	https://www.ncbi.nlm.nih.gov/sra/SRR17974345
Ej_SC1429	2019	SRR17974334	PRJNA675916	Human	New Zealand	Campylobacter jejuni	583	ST-45 complex	https://www.ncbi.nlm.nih.gov/sra/SRR17974334
Ej_SC1432	2019	SRR17974323	PRJNA675916	Human	New Zealand	Campylobacter jejuni	48	ST-48 complex	https://www.ncbi.nlm.nih.gov/sra/SRR17974323
Ej_SC1433	2019	SRR17974312	PRJNA675916	Human	New Zealand	Campylobacter jejuni	45	ST-45 complex	https://www.ncbi.nlm.nih.gov/sra/SRR17974312

Isolate no.	Year	Accession no.	BioProject no.	Source	Country	Species	Sequence type	Clonal complex	SRA hyperlink
Ej_SC1435	2019	SRR17974301	PRJNA675916	Human	New Zealand	Campylobacter jejuni	21	ST-21 complex	https://www.ncbi.nlm.nih.gov/sra/SRR17974301
Ej_SC1460	2019	SRR17974258	PRJNA675916	Human	New Zealand	Campylobacter jejuni	53	ST-21 complex	https://www.ncbi.nlm.nih.gov/sra/SRR17974258
Ej_SC1464	2019	SRR17974257	PRJNA675916	Human	New Zealand	Campylobacter jejuni	45	ST-45 complex	https://www.ncbi.nlm.nih.gov/sra/SRR17974257
Ej_SC1465	2019	SRR17974255	PRJNA675916	Human	New Zealand	Campylobacter jejuni	3538		https://www.ncbi.nlm.nih.gov/sra/SRR17974255
Ej_SC1466	2019	SRR17974254	PRJNA675916	Human	New Zealand	Campylobacter jejuni	45	ST-45 complex	https://www.ncbi.nlm.nih.gov/sra/SRR17974254
Ej_SC1467	2019	SRR17974253	PRJNA675916	Human	New Zealand	Campylobacter jejuni	45	ST-45 complex	https://www.ncbi.nlm.nih.gov/sra/SRR17974253
Ej_SC1470	2019	SRR17974252	PRJNA675916	Human	New Zealand	Campylobacter jejuni	422	ST-21 complex	https://www.ncbi.nlm.nih.gov/sra/SRR17974252
Ej_SC1472	2019	SRR17974251	PRJNA675916	Human	New Zealand	Campylobacter jejuni	38	ST-48 complex	https://www.ncbi.nlm.nih.gov/sra/SRR17974251
Ej_SC1473	2019	SRR17974250	PRJNA675916	Human	New Zealand	Campylobacter jejuni	45	ST-45 complex	https://www.ncbi.nlm.nih.gov/sra/SRR17974250
Ej_SC1474	2019	SRR17974249	PRJNA675916	Human	New Zealand	Campylobacter jejuni	618	ST-61 complex	https://www.ncbi.nlm.nih.gov/sra/SRR17974249
Ej_SC1475	2019	SRR17974248	PRJNA675916	Human	New Zealand	Campylobacter jejuni	53	ST-21 complex	https://www.ncbi.nlm.nih.gov/sra/SRR17974248
Ej_SC1476	2019	SRR17974247	PRJNA675916	Human	New Zealand	Campylobacter jejuni	3351	ST-61 complex	https://www.ncbi.nlm.nih.gov/sra/SRR17974247
Ej_SC1481	2019	SRR17974246	PRJNA675916	Human	New Zealand	Campylobacter jejuni	53	ST-21 complex	https://www.ncbi.nlm.nih.gov/sra/SRR17974246
Ej_SC1485	2019	SRR17974244	PRJNA675916	Human	New Zealand	Campylobacter jejuni	53	ST-21 complex	https://www.ncbi.nlm.nih.gov/sra/SRR17974244
Ej_SC1486	2019	SRR17974243	PRJNA675916	Human	New Zealand	Campylobacter jejuni	38	ST-48 complex	https://www.ncbi.nlm.nih.gov/sra/SRR17974243
Ej_SC1487	2019	SRR17974242	PRJNA675916	Human	New Zealand	Campylobacter jejuni	677	ST-677 complex	https://www.ncbi.nlm.nih.gov/sra/SRR17974242
Ej_SC1488	2019	SRR17974241	PRJNA675916	Human	New Zealand	Campylobacter jejuni	45	ST-45 complex	https://www.ncbi.nlm.nih.gov/sra/SRR17974241
Ej_SC1489	2019	SRR17974240	PRJNA675916	Human	New Zealand	Campylobacter jejuni	583	ST-45 complex	https://www.ncbi.nlm.nih.gov/sra/SRR17974240
Ej_SC1490	2019	SRR17974239	PRJNA675916	Human	New Zealand	Campylobacter jejuni	677	ST-677 complex	https://www.ncbi.nlm.nih.gov/sra/SRR17974239
Ej_SC1503	2019	SRR17974185	PRJNA675916	Human	New Zealand	Campylobacter jejuni	48	ST-48 complex	https://www.ncbi.nlm.nih.gov/sra/SRR17974185
Ej_SC1504	2019	SRR17974184	PRJNA675916	Human	New Zealand	Campylobacter jejuni	45	ST-45 complex	https://www.ncbi.nlm.nih.gov/sra/SRR17974184
Ej_SC1508	2019	SRR17974182	PRJNA675916	Human	New Zealand	Campylobacter jejuni	45	ST-45 complex	https://www.ncbi.nlm.nih.gov/sra/SRR17974182
Ej_SC1510	2019	SRR17974181	PRJNA675916	Human	New Zealand	Campylobacter jejuni	45	ST-45 complex	https://www.ncbi.nlm.nih.gov/sra/SRR17974181
Ej_SC1513	2019	SRR17974179	PRJNA675916	Human	New Zealand	Campylobacter jejuni	1919	ST-52 complex	https://www.ncbi.nlm.nih.gov/sra/SRR17974179
Ej_SC1514	2019	SRR17974178	PRJNA675916	Human	New Zealand	Campylobacter jejuni	190	ST-21 complex	https://www.ncbi.nlm.nih.gov/sra/SRR17974178
Ej_SC1516	2019	SRR17974177	PRJNA675916	Human	New Zealand	Campylobacter jejuni	21	ST-21 complex	https://www.ncbi.nlm.nih.gov/sra/SRR17974177
Ej_SC1517	2019	SRR17974176	PRJNA675916	Human	New Zealand	Campylobacter jejuni	53	ST-21 complex	https://www.ncbi.nlm.nih.gov/sra/SRR17974176
Ej_SC1518	2019	SRR17974175	PRJNA675916	Human	New Zealand	Campylobacter jejuni	53	ST-21 complex	https://www.ncbi.nlm.nih.gov/sra/SRR17974175
Ej_SC1521	2019	SRR17974174	PRJNA675916	Human	New Zealand	Campylobacter jejuni	51	ST-443 complex	https://www.ncbi.nlm.nih.gov/sra/SRR17974174
Ej_SC1522	2019	SRR17974173	PRJNA675916	Human	New Zealand	Campylobacter jejuni	190	ST-21 complex	https://www.ncbi.nlm.nih.gov/sra/SRR17974173
Ej_SC1535	2019	SRR17974162	PRJNA675916	Human	New Zealand	Campylobacter jejuni	45	ST-45 complex	https://www.ncbi.nlm.nih.gov/sra/SRR17974162
Ej_SC1538	2019	SRR17974160	PRJNA675916	Human	New Zealand	Campylobacter jejuni	2345	ST-206 complex	https://www.ncbi.nlm.nih.gov/sra/SRR17974160
Ej_SC1540	2019	SRR17974159	PRJNA675916	Human	New Zealand	Campylobacter jejuni	53	ST-21 complex	https://www.ncbi.nlm.nih.gov/sra/SRR17974159
Ej_SC1542	2019	SRR17974230	PRJNA675916	Human	New Zealand	Campylobacter jejuni	451	ST-21 complex	https://www.ncbi.nlm.nih.gov/sra/SRR17974230
Ej_SC1543	2019	SRR17974229	PRJNA675916	Human	New Zealand	Campylobacter jejuni	2345	ST-206 complex	https://www.ncbi.nlm.nih.gov/sra/SRR17974229
Ej_SC1544	2019	SRR17974228	PRJNA675916	Human	New Zealand	Campylobacter jejuni	6964	ST-354 complex	https://www.ncbi.nlm.nih.gov/sra/SRR17974228
Ej_SC1546	2019	SRR17974227	PRJNA675916	Human	New Zealand	Campylobacter jejuni	257	ST-257 complex	https://www.ncbi.nlm.nih.gov/sra/SRR17974227
Ej_SC1548	2019	SRR17974226	PRJNA675916	Human	New Zealand	Campylobacter jejuni	257	ST-257 complex	https://www.ncbi.nlm.nih.gov/sra/SRR17974226
Ej_SC1551	2019	SRR17974225	PRJNA675916	Human	New Zealand	Campylobacter jejuni	50	ST-21 complex	https://www.ncbi.nlm.nih.gov/sra/SRR17974225
Ej_SC1552	2019	SRR17974224	PRJNA675916	Human	New Zealand	Campylobacter jejuni	52	ST-52 complex	https://www.ncbi.nlm.nih.gov/sra/SRR17974224
Ej_SC1556	2019	SRR17974223	PRJNA675916	Human	New Zealand	Campylobacter jejuni	486	ST-21 complex	https://www.ncbi.nlm.nih.gov/sra/SRR17974223
Ej_SC1559	2019	SRR17974354	PRJNA675916	Human	New Zealand	Campylobacter jejuni	45	ST-45 complex	https://www.ncbi.nlm.nih.gov/sra/SRR17974354
Ej_SC1560	2019	SRR17974353	PRJNA675916	Human	New Zealand	Campylobacter jejuni	257	ST-257 complex	https://www.ncbi.nlm.nih.gov/sra/SRR17974353
Ej_SC1561	2019	SRR17974352	PRJNA675916	Human	New Zealand	Campylobacter jejuni	61	ST-61 complex	https://www.ncbi.nlm.nih.gov/sra/SRR17974352
Ej_SC1562	2019	SRR17974351	PRJNA675916	Human	New Zealand	Campylobacter jejuni	190	ST-21 complex	https://www.ncbi.nlm.nih.gov/sra/SRR17974351
Ej_SC1563	2019	SRR17974350	PRJNA675916	Human	New Zealand	Campylobacter jejuni	474	ST-48 complex	https://www.ncbi.nlm.nih.gov/sra/SRR17974350
Ej_SC1564	2019	SRR17974349	PRJNA675916	Human	New Zealand	Campylobacter jejuni	227	ST-206 complex	https://www.ncbi.nlm.nih.gov/sra/SRR17974349
Ej_SC1571	2019	SRR17974344	PRJNA675916	Human	New Zealand	Campylobacter jejuni	677	ST-677 complex	https://www.ncbi.nlm.nih.gov/sra/SRR17974344
Ej_SC1574	2019	SRR17974343	PRJNA675916	Human	New Zealand	Campylobacter jejuni	422	ST-21 complex	https://www.ncbi.nlm.nih.gov/sra/SRR17974343
Ej_SC1575	2019	SRR17974342	PRJNA675916	Human	New Zealand	Campylobacter jejuni	2345	ST-206 complex	https://www.ncbi.nlm.nih.gov/sra/SRR17974342
Ej_SC1576	2019	SRR17974341	PRJNA675916	Human	New Zealand	Campylobacter jejuni	422	ST-21 complex	https://www.ncbi.nlm.nih.gov/sra/SRR17974341
Ej_SC1578	2019	SRR17974340	PRJNA675916	Human	New Zealand	Campylobacter jejuni	12751		https://www.ncbi.nlm.nih.gov/sra/SRR17974340
Ej_SC1579	2019	SRR17974339	PRJNA675916	Human	New Zealand	Campylobacter jejuni	4337	ST-21 complex	https://www.ncbi.nlm.nih.gov/sra/SRR17974339
Ej_SC1580	2019	SRR17974338	PRJNA675916	Human	New Zealand	Campylobacter jejuni	53	ST-21 complex	https://www.ncbi.nlm.nih.gov/sra/SRR17974338
Ej_SC1583	2019	SRR17974337	PRJNA675916	Human	New Zealand	Campylobacter jejuni	22	ST-22 complex	https://www.ncbi.nlm.nih.gov/sra/SRR17974337
Ej_SC1604	2019	SRR17974319	PRJNA675916	Human	New Zealand	Campylobacter jejuni	474	ST-48 complex	https://www.ncbi.nlm.nih.gov/sra/SRR17974319
Ej_SC1605	2019	SRR17974318	PRJNA675916	Human	New Zealand	Campylobacter jejuni	45	ST-45 complex	https://www.ncbi.nlm.nih.gov/sra/SRR17974318
Ej_SC1606	2019	SRR17974317	PRJNA675916	Human	New Zealand	Campylobacter jejuni	42	ST-42 complex	https://www.ncbi.nlm.nih.gov/sra/SRR17974317
Ej_SC1607	2019	SRR17974316	PRJNA675916	Human	New Zealand	Campylobacter jejuni	53	ST-21 complex	https://www.ncbi.nlm.nih.gov/sra/SRR17974316
Ej_SC1609	2019	SRR17974315	PRJNA675916	Human	New Zealand	Campylobacter jejuni	474	ST-48 complex	https://www.ncbi.nlm.nih.gov/sra/SRR17974315
Ej_SC1637	2019	SRR17974289	PRJNA675916	Human	New Zealand	Campylobacter jejuni	474	ST-48 complex	https://www.ncbi.nlm.nih.gov/sra/SRR17974289
Ej_SC1639	2019	SRR17974288	PRJNA675916	Human	New Zealand	Campylobacter jejuni	48	ST-48 complex	https://www.ncbi.nlm.nih.gov/sra/SRR17974288
Ej_SC1642	2019	SRR17974287	PRJNA675916	Human	New Zealand	Campylobacter jejuni	61	ST-61 complex	https://www.ncbi.nlm.nih.gov/sra/SRR17974287
Ej_SC1648	2019	SRR17974286	PRJNA675916	Human	New Zealand	Campylobacter jejuni	2026	ST-403 complex	https://www.ncbi.nlm.nih.gov/sra/SRR17974286
Ej_SC1650	2019	SRR17974285	PRJNA675916	Human	New Zealand	Campylobacter jejuni	486	ST-21 complex	https://www.ncbi.nlm.nih.gov/sra/SRR17974285
Ej_SC1651	2019	SRR17974284	PRJNA675916	Human	New Zealand	Campylobacter jejuni	45	ST-45 complex	https://www.ncbi.nlm.nih.gov/sra/SRR17974284
Ej_SC1654	2019	SRR17974283	PRJNA675916	Human	New Zealand	Campylobacter jejuni	190	ST-21 complex	https://www.ncbi.nlm.nih.gov/sra/SRR17974283
Ej_SC1655	2019	SRR17974282	PRJNA675916	Human	New Zealand	Campylobacter jejuni	6964	ST-354 complex	https://www.ncbi.nlm.nih.gov/sra/SRR17974282
Ej_SC1656	2019	SRR17974281	PRJNA675916	Human	New Zealand	Campylobacter jejuni	508	ST-508 complex	https://www.ncbi.nlm.nih.gov/sra/SRR17974281
Ej_SC1658	2019	SRR17974280	PRJNA675916	Human	New Zealand	Campylobacter jejuni	53	ST-21 complex	https://www.ncbi.nlm.nih.gov/sra/SRR17974280
Ej_SC1659	2019	SRR17974278	PRJNA675916	Human	New Zealand	Campylobacter jejuni	45	ST-45 complex	https://www.ncbi.nlm.nih.gov/sra/SRR17974278
Ej_SC1660	2019	SRR17974277	PRJNA675916	Human	New Zealand	Campylobacter jejuni	474	ST-48 complex	https://www.ncbi.nlm.nih.gov/sra/SRR17974277
Ej_SC1664	2019	SRR17974276	PRJNA675916	Human	New Zealand	Campylobacter jejuni	474	ST-48 complex	https://www.ncbi.nlm.nih.gov/sra/SRR17974276
Ej_SC1666	2019	SRR17974275	PRJNA675916	Human	New Zealand	Campylobacter jejuni	53	ST-21 complex	https://www.ncbi.nlm.nih.gov/sra/SRR17974275
Ej_SC1676	2019	SRR17974264	PRJNA675916	Human	New Zealand	Campylobacter jejuni	50	ST-21 complex	https://www.ncbi.nlm.nih.gov/sra/SRR17974264
Ej_SC1678	2019	SRR17974263	PRJNA675916	Human	New Zealand	Campylobacter jejuni	1517	ST-354 complex	https://www.ncbi.nlm.nih.gov/sra/SRR17974263
Ej_SC1679	2019	SRR17974158	PRJNA675916	Human	New Zealand	Campylobacter jejuni	658	ST-658 complex	https://www.ncbi.nlm.nih.gov/sra/SRR17974158
Ej_SC1681	2019	SRR17974157	PRJNA675916	Human	New Zealand	Campylobacter jejuni	2345	ST-206 complex	https://www.ncbi.nlm.nih.gov/sra/SRR17974157
Ej_SC1683	2019	SRR17974156	PRJNA675916	Human	New Zealand	Campylobacter jejuni	4337	ST-21 complex	https://www.ncbi.nlm.nih.gov/sra/SRR17974156
Ej_SC1684	2019	SRR17974155	PRJNA675916	Human	New Zealand	Campylobacter jejuni	45	ST-45 complex	https://www.ncbi.nlm.nih.gov/sra/SRR17974155
Ej_SC1690	Data missing	SRR17974150	PRJNA675916	Chicken	New Zealand	Campylobacter jejuni	48	ST-48 complex	https://www.ncbi.nlm.nih.gov/sra/SRR17974150
Ej_SC1691	Data missing	SRR17974149	PRJNA675916	Chicken	New Zealand	Campylobacter jejuni	2345	ST-206 complex	https://www.ncbi.nlm.nih.gov/sra/SRR17974149
Ej_SC1692	Data missing	SRR17974148	PRJNA675916	Chicken	New Zealand	Campylobacter jejuni	45	ST-45 complex	https://www.ncbi.nlm.nih.gov/sra/SRR17974148
Ej_SC1693	Data missing	SRR17974147	PRJNA675916	Chicken	New Zealand	Campylobacter jejuni	45	ST-45 complex	https://www.ncbi.nlm.nih.gov/sra/SRR17974147
Ej_SC1695	Data missing	SRR17974145	PRJNA675916	Chicken	New Zealand	Campylobacter jejuni	45	ST-45 complex	https://www.ncbi.nlm.nih.gov/sra/SRR17974145
Ej_SC1696	Data missing	SRR17974144	PRJNA675916	Chicken	New Zealand	Campylobacter jejuni	2345	ST-206 complex	https://www.ncbi.nlm.nih.gov/sra/SRR17974144
Ej_SC1698	Data missing	SRR17974140	PRJNA675916	Chicken	New Zealand	Campylobacter jejuni	227	ST-206 complex	https://www.ncbi.nlm.nih.gov/sra/SRR17974140

Isolate no.	Year	Accession no.	BioProject no.	Source	Country	Species	Sequence type	Clonal complex	SRA hyperlink
Ej_SC1699	Data missing	SRR17974139	PRJNA675916	Chicken	New Zealand	Campylobacter jejuni	6964	ST-354 complex	https://www.ncbi.nlm.nih.gov/sra/SRR17974139
Ej_SC1700	Data missing	SRR17974138	PRJNA675916	Chicken	New Zealand	Campylobacter jejuni	422	ST-21 complex	https://www.ncbi.nlm.nih.gov/sra/SRR17974138
Ej_SC1701	Data missing	SRR17974137	PRJNA675916	Chicken	New Zealand	Campylobacter jejuni	422	ST-21 complex	https://www.ncbi.nlm.nih.gov/sra/SRR17974137
Ej_SC1702	Data missing	SRR17974136	PRJNA675916	Chicken	New Zealand	Campylobacter jejuni	2345	ST-206 complex	https://www.ncbi.nlm.nih.gov/sra/SRR17974136
Ej_SC1704	Data missing	SRR17974134	PRJNA675916	Chicken	New Zealand	Campylobacter jejuni	45	ST-45 complex	https://www.ncbi.nlm.nih.gov/sra/SRR17974134
Ej_SC1709	Data missing	SRR17974128	PRJNA675916	Chicken	New Zealand	Campylobacter jejuni	45	ST-45 complex	https://www.ncbi.nlm.nih.gov/sra/SRR17974128
Ej_SC1710	Data missing	SRR17974127	PRJNA675916	Chicken	New Zealand	Campylobacter jejuni	6964	ST-354 complex	https://www.ncbi.nlm.nih.gov/sra/SRR17974127
Ej_SC1711	Data missing	SRR17974222	PRJNA675916	Chicken	New Zealand	Campylobacter jejuni	8187		https://www.ncbi.nlm.nih.gov/sra/SRR17974222
Ej_SC1712	Data missing	SRR17974221	PRJNA675916	Chicken	New Zealand	Campylobacter jejuni	53	ST-21 complex	https://www.ncbi.nlm.nih.gov/sra/SRR17974221
Ej_SC1713	Data missing	SRR17974220	PRJNA675916	Chicken	New Zealand	Campylobacter jejuni	45	ST-45 complex	https://www.ncbi.nlm.nih.gov/sra/SRR17974220
Ej_SC1714	Data missing	SRR17974219	PRJNA675916	Chicken	New Zealand	Campylobacter jejuni	45	ST-45 complex	https://www.ncbi.nlm.nih.gov/sra/SRR17974219
Ej_SC1715	Data missing	SRR17974218	PRJNA675916	Chicken	New Zealand	Campylobacter jejuni	53	ST-21 complex	https://www.ncbi.nlm.nih.gov/sra/SRR17974218
Ej_SC1719	Data missing	SRR17974213	PRJNA675916	Chicken	New Zealand	Campylobacter jejuni	6964	ST-354 complex	https://www.ncbi.nlm.nih.gov/sra/SRR17974213
Ej_SC1720	Data missing	SRR17974212	PRJNA675916	Chicken	New Zealand	Campylobacter jejuni	6964	ST-354 complex	https://www.ncbi.nlm.nih.gov/sra/SRR17974212
Ej_SC1721	Data missing	SRR17974211	PRJNA675916	Chicken	New Zealand	Campylobacter jejuni	48	ST-48 complex	https://www.ncbi.nlm.nih.gov/sra/SRR17974211
Ej_SC1722	Data missing	SRR17974210	PRJNA675916	Chicken	New Zealand	Campylobacter jejuni	583	ST-45 complex	https://www.ncbi.nlm.nih.gov/sra/SRR17974210
Ej_SC1723	Data missing	SRR17974209	PRJNA675916	Chicken	New Zealand	Campylobacter jejuni	3538		https://www.ncbi.nlm.nih.gov/sra/SRR17974209
Ej_SC1724	Data missing	SRR17974208	PRJNA675916	Chicken	New Zealand	Campylobacter jejuni	nJ09		https://www.ncbi.nlm.nih.gov/sra/SRR17974208
Ej_SC1726	Data missing	SRR17974206	PRJNA675916	Chicken	New Zealand	Campylobacter jejuni	583	ST-45 complex	https://www.ncbi.nlm.nih.gov/sra/SRR17974206
Ej_SC1727	Data missing	SRR17974205	PRJNA675916	Chicken	New Zealand	Campylobacter jejuni	50	ST-21 complex	https://www.ncbi.nlm.nih.gov/sra/SRR17974205
Ej_SC1728	Data missing	SRR17974203	PRJNA675916	Chicken	New Zealand	Campylobacter jejuni	45	ST-45 complex	https://www.ncbi.nlm.nih.gov/sra/SRR17974203
Ej_SC1729	Data missing	SRR17974202	PRJNA675916	Chicken	New Zealand	Campylobacter jejuni	45	ST-45 complex	https://www.ncbi.nlm.nih.gov/sra/SRR17974202

Table S2**.** Antimicrobial resistance genes and mutations used to infer phenotypic resistance in *Campylobacter* isolates.

**Drug class**	**Antimicrobial**	**Gene**	**Mutation**	**Reference**
**Aminoglycoside**	Gentamicin<br>Streptomycin	*aph(3')-IIIa*<br>*aadE-Cc*<br>*aad9*	None<br>None	Ramirez and Tolmasky (2010)
**Lincosamide**	Lincomycin	*lnu*(C)	None	Zhao et al. (2016)
**Beta-lactam**	Ampicillin	$bla_{OXA-184}$, $bla_{OXA-185}$<br>bla_{OXA-61}	None<br>bla_{OXA-61} G57T	Zeng et al. (2014); Whitehouse et al. (2018)
**Quinolone**	Ciprofloxacin	*gyrA*	GyrA T86I	Hakanen et al. (2002)
**Macrolide**	Erythromycin	*erm*(B)<br>23S rRNA	None<br>A2074T, A2075G	Qin et al. (2014)<br>Ladely et al. (2009)
**Tetracycline**	Tetracycline	*tet*(O)	None	Whitehouse et al. (2018)

Table S3. Summary of sequence types (STs) for *Campylobacter jejuni* and *Campylobacter coli* between Australia and New Zealand, chicken and human isolates, 2017–2019.

* Bold indicates sequence types that appear in both countries

Campylobacter jejuni										*Campylobacter coli*									
**AUS**				**NZ**				**All**		**AUS**				**NZ**				**All**	
ST	Chicken	Human	Total	ST	Chicken	Human	Total	Combined ST	Combined total	ST	Chicken	Human	Total	ST	Chicken	Human	Total	Combined ST	Combined total
**50**	23	96	119	**45**	24	118	142	**45**	162	1181	47	30	77	2256	16	2	18	1181	77
528	10	27	37	6964	46	40	86	**50**	160	**827**	46	12	58	**8926**	4	8	12	**827**	59
**48**	4	29	33	**48**	26	44	70	**48**	103	**825**	34	19	53	1581	3	2	5	**825**	57
567	3	29	32	**583**	11	42	53	6964	86	860	26	9	35	4009	1	3	4	860	35
46	5	22	27	**53**	12	39	51	**583**	68	9436	12	10	22	**825**	1	3	4	9419	22
2083	7	19	26	**50**	10	31	41	**53**	65	9419	15	7	22	nC09	0	2	2	9436	22
**21**	5	19	24	2345	10	18	28	**190**	39	832	16	0	16	854	1	1	2	2256	18
**61**	2	19	21	474	4	24	28	528	37	4175	12	3	15	8712	2	0	2	832	16
**45**	9	11	20	4337	8	19	27	**61**	35	3985	13	0	13	900	2	0	2	4175	15
**658**	2	18	20	**227**	4	23	27	567	32	829	7	4	11	12072	0	1	1	3985	13
4896	12	7	19	677	1	25	26	**21**	31	9420	4	2	6	12248	0	1	1	**8926**	13
7323	10	9	19	**190**	6	19	25	**227**	30	1243	5	0	5	3072	0	1	1	829	11
2398	0	18	18	422	4	11	15	2345	28	10144	4	0	4	**827**	0	1	1	9420	6
**257**	4	13	17	486	2	12	14	474	28	10164	4	0	4	830	0	1	1	1243	5
**42**	2	15	17	**61**	0	14	14	**257**	27	966	4	0	4	nC07	0	1	1	1581	5
**51**	7	9	16	**508**	0	11	11	4337	27	10145	3	0	3	3230	1	0	1	10144	4
9432	10	5	15	**257**	2	8	10	46	27	1427	3	0	3	5734	1	0	1	10164	4
**583**	5	10	15	2026	0	8	8	2083	26	**828**	3	0	3	**828**	1	0	1	4009	4
449	12	2	14	**42**	0	8	8	677	26	9789	3	0	3	890	1	0	1	**828**	4
**53**	5	9	14	**520**	0	8	8	**42**	25	831	0	2	2					966	4
**190**	1	13	14	**21**	0	7	7	**508**	22	2179	1	1	2	**Total isolates**			**61**	10145	3
**538**	3	10	13	3538	1	5	6	**658**	22	9435	1	1	2					1427	3
49	5	7	12	25	0	6	6	**51**	21	894	2	0	2					9789	3
**508**	0	11	11	**38**	0	6	6	4896	19	10149	0	1	1					2179	2
**52**	1	9	10	**538**	0	6	6	**538**	19	10153	0	1	1					831	2
**137**	3	6	9	1517	1	4	5	7323	19	10157	0	1	1					854	2
**696**	1	8	9	**51**	1	4	5	2398	18	10158	0	1	1					8712	2
525	0	9	9	**22**	0	4	4	422	15	1016	0	1	1					894	2
10130	3	5	8	2535	0	4	4	9432	15	10161	0	1	1					900	2
10132	0	8	8	5655	1	2	3	449	14	10165	0	1	1					9435	2
2343	0	8	8	**696**	1	2	3	486	14	10168	0	1	1					nC09	2
**535**	4	3	7	**52**	1	1	2	49	12	10169	0	1	1					10147	1
128	1	6	7	**535**	1	1	2	**52**	12	10172	0	1	1					10148	1
9429	6	0	6	**658**	1	1	2	**520**	12	10173	0	1	1					10149	1
10127	2	2	4	8185	1	1	2	**696**	12	1055	0	1	1					10150	1
**520**	0	4	4	12755	0	2	2	**137**	11	1666	0	1	1					10151	1
10134	2	1	3	**137**	0	2	2	525	9	6110	0	1	1					10153	1
**227**	2	1	3	2081	0	2	2	**535**	9	8323	0	1	1					10156	1
9425	2	1	3	2347	0	2	2	10130	8	872	0	1	1					10157	1
991	2	1	3	**354**	0	2	2	10132	8	**8926**	0	1	1					10158	1
534	0	3	3	3712	0	2	2	2026	8	9969	0	1	1					10159	1
10123	2	0	2	436	0	2	2	2343	8	nC01	0	1	1					1016	1

Campylobacter jejuni

AUS				NZ				All	
ST	Chicken	Human	Total	ST	Chicken	Human	Total	Combined ST	Combined total
132	2	0	2	451	0	2	2	**38**	8
10139	1	1	2	618	0	2	2	128	7
1911	1	1	2	3721	1	0	1	25	6
5687	1	1	2	4500	1	0	1	3538	6
6891	1	1	2	8187	1	0	1	9429	6
692	1	1	2	nJ05	1	0	1	1517	5
10133	0	2	2	nJ08	1	0	1	**22**	5
10143	0	2	2	nJ09	1	0	1	10127	4
10155	0	2	2	12749	0	1	1	2535	4
1078	0	2	2	12751	0	1	1	10134	3
2947	0	2	2	12752	0	1	1	**354**	3
305	0	2	2	12761	0	1	1	534	3
**38**	0	2	2	12762	0	1	1	5655	3
8436	0	2	2	1326	0	1	1	9425	3
933	0	2	2	177	0	1	1	991	3
10124	1	0	1	1919	0	1	1	10123	2
10126	1	0	1	1956	0	1	1	10133	2
10129	1	0	1	2350	0	1	1	10139	2
10136	1	0	1	2392	0	1	1	10143	2
**699**	1	0	1	27	0	1	1	10155	2
992	1	0	1	3351	0	1	1	1078	2
996	1	0	1	3610	0	1	1	12755	2
10138	0	1	1	3676	0	1	1	132	2
10146	0	1	1	3711	0	1	1	1911	2
10152	0	1	1	4492	0	1	1	2081	2
1525	0	1	1	530	0	1	1	2347	2
161	0	1	1	5647	0	1	1	2947	2
1728	0	1	1	6139	0	1	1	305	2
1972	0	1	1	**699**	0	1	1	3712	2
2107	0	1	1	8065	0	1	1	436	2
**22**	0	1	1	8658	0	1	1	451	2
2247	0	1	1	nJ01	0	1	1	5687	2
2349	0	1	1	nJ03	0	1	1	618	2
2580	0	1	1	nJ04	0	1	1	6891	2
**354**	0	1	1	nJ06	0	1	1	692	2
4010	0	1	1	nJ07	0	1	1	**699**	2
4080	0	1	1	nJ10	0	1	1	8185	2
4577	0	1	1					8436	2
464	0	1	1	**Total isolates**			**808**	933	2
4684	0	1	1					10124	1
5	0	1	1					10126	1
5221	0	1	1					10129	1
6788	0	1	1					10136	1
820	0	1	1					10138	1

Campylobacter coli

AUS				NZ				All	
ST	Chicken	Human	Total	ST	Chicken	Human	Total	Combined ST	Combined total
nC10	0	1	1					10161	1
10147	1	0	1					10162	1
10148	1	0	1					10163	1
10150	1	0	1					10165	1
10151	1	0	1					10166	1
10156	1	0	1					10168	1
10159	1	0	1					10169	1
10162	1	0	1					10170	1
10163	1	0	1					10172	1
10166	1	0	1					10173	1
10170	1	0	1					1055	1
1173	1	0	1					1173	1
1177	1	0	1					1177	1
1563	1	0	1					12072	1
1766	1	0	1					12248	1
4044	1	0	1					1563	1
6159	1	0	1					1666	1
9433	1	0	1					1766	1
9912	1	0	1					3072	1
nC02	1	0	1					3230	1
nC05	1	0	1					4044	1
nC06	1	0	1					5734	1
								6110	1
**Total isolates**			**406**					6159	1
								830	1
								8323	1
								872	1
								890	1
								9433	1
								9912	1
								9969	1
								nC01	1
								nC02	1
								nC05	1
								nC06	1
								nC07	1
								nC10	1
								**Total isolate**	**467**

Campylobacter jejuni									
AUS				NZ				All	
ST	Chicken	Human	Total	ST	Chicken	Human	Total	Combined ST	Combined total
8516	0	1	1					10146	1
859	0	1	1					10152	1
9592	0	1	1					12749	1
9608	0	1	1					12751	1
982	0	1	1					12752	1
9965	0	1	1					12761	1
9966	0	1	1					12762	1
9967	0	1	1					1326	1
9968	0	1	1					1525	1
								161	1
**Total isolates**			**747**					1728	1
								177	1
								1919	1
								1956	1
								1972	1
								2107	1
								2247	1
								2349	1
								2350	1
								2392	1
								2580	1
								27	1
								3351	1
								3610	1
								3676	1
								3711	1
								3721	1
								4010	1
								4080	1
								4492	1
								4500	1
								4577	1
								464	1
								4684	1
								5	1
								5221	1
								530	1
								5647	1
								6139	1
								6788	1
								8065	1
								8187	1
								820	1
								8516	1

Campylobacter coli									
AUS				NZ				All	
ST	Chicken	Human	Total	ST	Chicken	Human	Total	Combined ST	Combined total

Campylobacter jejuni										*Campylobacter coli*									
**AUS**				**NZ**				**All**		**AUS**				**NZ**				**All**	
ST	Chicken	Human	Total	ST	Chicken	Human	Total	Combined ST	Combined total	ST	Chicken	Human	Total	ST	Chicken	Human	Total	Combined ST	Combined total
								859	1										
								8658	1										
								9592	1										
								9608	1										
								982	1										
								992	1										
								996	1										
								9965	1										
								9966	1										
								9967	1										
								9968	1										
								nJ01	1										
								nJ03	1										
								nJ04	1										
								nJ05	1										
								nJ06	1										
								nJ07	1										
								nJ08	1										
								nJ09	1										
								nJ10	1										
								**Total isolates**	**1555**										

Table S4. *Campylobacter coli* isolate metadata for Fig S1.
Isolates from the United Kingdom are sourced from Sheppard et al., 2013, doi: 10.1111/mec.12162

**Isolate**	**Alias**	**ST**	**Country**	**Sub-clade**
15_BIGS0015_UK_ST887	15UK	887	UK	1a
17_BIGS0017_UK_ST867	17UK	867	UK	1a
19_BIGS0019_UK_ST8320	19UK	8320	UK	1a
2_BIGS0002_UK_ST867	2UK	867	UK	1a
21_BIGS0021_UK_ST828	21UK	828	UK	1a
24_BIGS0024_UK_ST828	24UK	828	UK	1a
5_BIGS0005_UK_ST2696	5UK	2696	UK	1a
Cc_18A3055F1	1AU	10151	Australia	1b
Dc_18Q1084H1	2AU	10157	Australia	1b
18_BIGS0018_UK_ST3667	18UK	3667	UK	1b
20_BIGS0020_UK_STnd	20UK	nd	UK	1b
Cc_18A3062F1	3AU	1243	Australia	1c
Cc_18A3063F1	4AU	1243	Australia	1c
Cc_18A3057F1	5AU	1243	Australia	1c
Cc_18N3082F1	6AU	1243	Australia	1c
Cc_18A3063F2	7AU	1243	Australia	1c
Cc_17Q3023F1	8AU	1766	Australia	1c
23_BIGS0023_UK_ST3311	23UK	3311	UK	1c
Cc_17Q3076F1	9AU	10147	Australia	2
Cc_17Q3084F2	10AU	10148	Australia	2
1_BIGS0001_UK_STnd	1UK	nd	UK	2
10_BIGS0010_UK_ST1572	10UK	1572	UK	2
11_BIGS0011_UK_ST2016	11UK	2016	UK	2
12_BIGS0012_UK_ST2326	12UK	2326	UK	2
Dc_17V1004H1	11AU	10149	Australia	3
Dc_18N1004H1	12AU	10153	Australia	3
Cc_17Q3129F1	13AU	10159	Australia	3
Cc_18V3029F1	14AU	10162	Australia	3
Cc_18V3034F1	15AU	10163	Australia	3
Dc_17V1007H1	16AU	10165	Australia	3
Dc_18N1003H1	17AU	10168	Australia	3
Cc_18N3027F1	18AU	10170	Australia	3
Dc_18Q1089H1	19AU	10173	Australia	3
Dc_18N1089H1	20AU	6110	Australia	3
Cc_17Q3073F1	21AU	6159	Australia	3
Cc_17Q3053F1	22AU	9433	Australia	3
Cc_17Q3099F1	23AU	9789	Australia	3
Cc_18Q3018F1	24AU	9789	Australia	3
Cc_18Q3019F1	25AU	9789	Australia	3
Cc_17V3023F1	26AU	9912	Australia	3
Dc_18V1052H1	27AU	nC01	Australia	3
Cc_18N3006F1	28AU	nC02	Australia	3
Cc_17N3018F1	29AU	nC06	Australia	3
3_BIGS0003_UK_ST1992	3UK	1992	UK	3
6_BIGS0006_UK_ST7243	6UK	7243	UK	3
7_BIGS0007_UK_ST1576	7UK	1576	UK	3
8_BIGS0008_UK_ST1670	8UK	1670	UK	3
9_BIGS0009_UK_ST1643	9UK	1643	UK	3

Table S5. Clusters by core genome single nucleotide polymorphism (SNP) thresholds for *Campylobacter jejuni* and *Campylobacter coli* sequence types with isolates from Australia and New Zealand.

* bold indicates sequence types with isolates from both Australia and New Zealand from at least <100 SNPs

Species	Sequence type	Total number of isolates	Australian count N (% human)	New Zealand count N (% human)	Number of clusters at SNP threshold N (N AU/NZ cluster)			
					<100 SNPs	<50 SNPs	<20 SNPs	<5 SNPs
C. coli	**827**	59	58 (20.7)	1 (100)	26 (1)	29 (1)	33 (0)	37 (0)
C. coli	**825**	57	53 (35.8)	4 (75.0)	11 (1)	14 (1)	19 (0)	26 (0)
C. coli	8926	13	1 (100)	12 (66.7)	2 (0)	2 (0)	2 (0)	2 (0)
C. coli	828	4	3 (0)	1 (0)	3 (0)	3 (0)	3 (0)	3 (0)
C. jejuni	**45**	162	20 (55)	142 (83.1)	47 (2)	54 (2)	70 (0)	106 (0)
C. jejuni	**50**	160	119 (80.1)	41 (75.6)	49 (1)	54 (1)	66 (0)	96 (0)
C. jejuni	48	103	33 (87.9)	70 (62.9)	10 (0)	12 (0)	15 (0)	26 (0)
C. jejuni	583	68	15 (66.7)	53 (79.2)	9 (0)	10 (0)	14 (0)	31 (0)
C. jejuni	53	65	14 (64.3)	51 (76.5)	27 (0)	30 (0)	37 (0)	50 (0)
C. jejuni	190	39	14 (92.9)	25 (76.0)	11 (0)	14 (0)	18 (0)	29 (0)
C. jejuni	61	35	21 (90.5)	14 (100)	31 (0)	32 (0)	33 (0)	34 (0)
C. jejuni	**21**	31	24 (79.2)	7 (100)	7 (1)	8 (0)	10 (0)	18 (0)
C. jejuni	227	30	3 (33.3)	27 (85.2)	4 (0)	4 (0)	5 (0)	10 (0)
C. jejuni	257	27	17 (76.5)	10 (80.0)	4 (0)	5 (0)	7 (0)	16 (0)
C. jejuni	42	25	17 (88.2)	8 (100)	14 (0)	14 (0)	17 (0)	23 (0)
C. jejuni	**508**	22	11 (100)	11 (100)	5 (1)	5 (1)	7 (2)	17 (0)
C. jejuni	658	22	20 (90.0)	2 (50.0)	2 (0)	2 (0)	3 (0)	9 (0)
C. jejuni	51	21	16 (56.3)	5 (80.0)	7 (0)	8 (0)	10 (0)	14 (0)
C. jejuni	**538**	19	13 (76.9)	6 (100)	1 (1)	1 (1)	4 (0)	11 (0)
C. jejuni	52	12	10 (90.0)	2 (50.0)	5 (0)	6 (0)	6 (0)	8 (0)
C. jejuni	520	12	4 (100)	8 (100)	6 (0)	8 (0)	8 (0)	12 (0)
C. jejuni	**696**	12	9 (88.9)	3 (66.7)	4 (1)	4 (1)	7 (0)	9 (0)
C. jejuni	**137**	11	9 (66.7)	2 (100)	5 (1)	6 (1)	6 (1)	9 (0)
C. jejuni	535	9	7 (42.9)	2 (50.0)	3 (0)	4 (0)	6 (0)	7 (0)
C. jejuni	**38**	8	2 (100)	6 (100)	2 (1)	2 (1)	5 (0)	8 (0)
C. jejuni	22	5	1 (100)	4 (100)	2 (0)	2 (0)	3 (0)	4 (0)
C. jejuni	354	3	1 (100)	2 (100)	3 (0)	3 (0)	3 (0)	3 (0)
C. jejuni	699	2	1 (0)	1 (100)	2 (0)	2 (0)	2 (0)	2 (0)

Table S6. Summary of genetic determinants of antimicrobial resistance (AMR) in *C.jejuni* and *C. coli* recovered from human and chicken samples in Australia and New Zealand, 2017 - 2019.

^ Values are presented at the percentage of isolates with the respective AMR gene or mutation per source; - gene not detected

			No. of antimicrobial resistance genes and mutations (%^)																		
			**Aminoglycoside**			**β-lactam**											**Lincosamide**	**Quinolone**	**Macrolide**			**Tetracyline**
**Species**	**Country**	**Source**	*aph(3')-IIIa*	*aad9*	*aadE*	*bla* $_{OXA-61}$	*bla* $_{OXA-61}$ G57T	*bla* $_{OXA-184}$	*bla* $_{OXA-185}$	*bla* $_{OXA-193}$	*bla* $_{OXA-449}$	*bla* $_{OXA-460}$	*bla* $_{OXA-466}$	*bla* $_{OXA-624}$	*bla* $_{OXA-625}$	*bla* $_{OXA-631}$	*lnu* (C)	*gyrA* T86I	*erm* (B)	23S rRNA A2074T	23S rRNA A2075G	*tet* (O)
C. jejuni (n = 1555)	Australia	Chicken (n=191)	0 (0.0)	0 (0.0)	0 (0.0)	95 (49.7)	15 (7.9)	4 (2.1)	1 (0.5)	90 (47.1)	2 (1.0)	11 (5.8)	7 (3.7)	1 (0.5)	2 (1.0)	1 (0.5)	-	20 (10.5)	0 (0.0)	0 (0.0)	0 (0.0)	32 (16.8)
		Human (n=556)	2 (0.4)	1 (0.2)	2 (0.4)	369 (66.4)	37 (6.7)	6 (1.1)	2 (0.4)	340 (61.2)	6 (1.1)	39 (7.0)	1 (0.2)	2 (0.4)	1 (0.2)	9 (1.6)	-	80 (14.4)	1 (0.2)	1 (0.2)	1 (0.2)	64 (11.5)
	New Zealand	Chicken (n=185)	0 (0.0)	0 (0.0)	0 (0.0)	115 (62.2)	4 (2.2)	6 (3.2)	0 (0.0)	89 (48.1)	2 (1.1)	49 (26.5)	0 (0.0)	0 (0.0)	2 (1.1)	1 (0.5)	-	46 (24.9)	0 (0.0)	0 (0.0)	0 (0.0)	51 (27.6)
		Human (n=623)	0 (0.0)	0 (0.0)	0 (0.0)	443 (71.1)	33 (5.3)	25 (4.0)	0 (0.0)	398 (63.9)	17 (2.7)	47 (7.5)	4 (0.6)	3 (0.5)	0 (0.0)	3 (0.5)	-	53 (8.5)	0 (0.0)	0 (0.0)	0 (0.0)	52 (8.3)
		Total count	2	1	2	1022	89	41	3	917	27	146	12	6	5	14		199	1	1	1	199
		Total percentage	0.1	0.1	0.1	65.7	5.7	2.6	0.2	59.0	1.7	9.4	0.8	0.4	0.3	0.9		12.8	0.1	0.1	0.1	12.8
C. coli (n=467)	Australia	Chicken (n=286)	0 (0.0)	0 (0.0)	0 (0.0)	155 (54.2)	0 (0.0)	-	-	157 (54.9)	-	5 (1.7)	-	-	-	-	0 (0.0)	0 (0.0)	0 (0.0)	-	1 (0.3)	37 (12.9)
		Human (n=120)	2 (1.7)	1 (0.8)	1 (0.8)	70 (58.3)	2 (1.7)	-	-	71 (59.2)	-	1 (0.8)	-	-	-	-	1 (0.8)	1 (0.8)	1 (0.8)	-	1 (0.8)	11 (9.2)
	New Zealand	Chicken (n=34)	0 (0.0)	0 (0.0)	0 (0.0)	33 (97.1)	0 (0.0)	-	-	33 (97.1)	-	1 (2.9)	-	-	-	-	0 (0.0)	0 (0.0)	0 (0.0)	-	0 (0.0)	0 (0.0)
		Human (n=27)	0 (0.0)	0 (0.0)	0 (0.0)	21 (77.8)	1 (3.7)	-	-	21 (77.8)	-	0 (0.0)	-	-	-	-	0 (0.0)	0 (0.0)	0 (0.0)	-	1 (3.7)	2 (7.4)
		Total count	2	1	1	279	3			282		7					1	1	1		3	50
		Total percentage	0.4	0.2	0.2	59.7	0.6			60.4		1.5					0.2	0.2	0.2		0.6	10.7

Table S7. Antimicrobial resistance genotype profile (from genes and mutations conferring resistance) for *C. jejuni* and *C. coli* isolates recovered from human and chicken samples in Australia and New Zealand, 2017 - 2019.
-' profile not detected

Resistance profile	*C. coli* n=467			*C. jejuni* n=1,555		
	**Australia**	**New Zealand**	**Total (%)**	**Australia**	**New Zealand**	**Total (%)**
Susceptible	354	59	413 (88.4)	555	644	1199 (77.1)
Aminoglycoside	1	-	1 (0.2)	-	-	-
Aminoglycoside + Beta-lactam + Macrolide + Tetracycline	1	-	1 (0.2)	-	-	-
Aminoglycoside + Beta-lactam + Quinolone + Macrolide	-	-	-	1	-	1 (0.1)
Aminoglycoside + Beta-lactam + Quinolone + Tetracycline	-	-	-	1	-	1 (0.1)
Aminoglycoside + Macrolide + Tetracycline	1	-	1 (0.2)	-	-	-
Beta-lactam	1	-	1 (0.2)	34	59	93 (6.0)
Beta-lactam + Quinolone	-	-	-	21	1	22 (1.4)
Beta-lactam + Quinolone + Tetracycline	-	-	-	4	7	11 (0.7)
Beta-lactam + Tetracycline	-	1	1 (0.2)	4	1	5 (0.3)
Lincosamide	1	-	1 (0.2)	-	-	-
Macrolide	1	-	1 (0.2)	-	-	-
Macrolide + Tetracycline	-	1	1 (0.2)	-	-	-
Quinolone	-	-	-	39	1	40 (2.6)
Quinolone + Macrolide	-	-	-	1	-	1 (0.1)
Quinolone + Macrolide + Tetracycline	-	-	-	1	-	1 (0.1)
Quinolone + Tetracycline	1	-	1 (0.2)	32	90	122 (7.8)
Tetracycline	45	-	45 (9.6)	54	5	59 (3.8)
